# Photoinduced Cleavage of Alkenyl Fluorides for Nucleophilic Acyl Substitution via In Situ Generated Acyl Fluorides

**DOI:** 10.1021/acs.orglett.5c04085

**Published:** 2025-11-25

**Authors:** Emma S. Gogarnoiu, Melissa S. Griffin, Ajay H. Bansode, Joshua M. Paolillo, Joseph M. Bergen, Marvin Parasram

**Affiliations:** Department of Chemistry, 5894 New York University, 24 Waverly Place, Third Floor, New York, New York 10003, United States

## Abstract

Acyl fluorides are valuable intermediates due to their stability and unique reactivity with amines, enabling selective acyl substitution. We report a mild one-pot method to access amides, esters, and thioesters from alkenyl fluorides. Visible-light photoexcitation of 4-nitrophthalonitrile promotes cleavage of the alkenyl fluoride, forming acyl fluorides in situ for nucleophilic acyl substitution. This protocol offers broad utility as demonstrated in the synthesis of linear peptide fragments.

## Graphical abstract

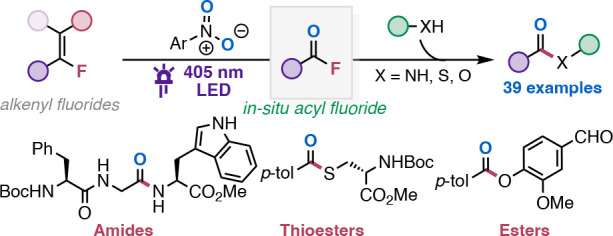

Among the broad library of carboxylic acid derivatives, acyl fluorides have emerged as valuable electrophiles for their unique stability, chemoselectivity for amine and anionic nucleophiles, and compatibility with sterically hindered coupling partners.^1–3^ Furthermore, acyl fluorides have greater stability than their highly sensitive chloride counterpart, making them more attractive for applications that demand broader functional group tolerance.^2,4^ As such, they have been sought as important reactants in transition metal catalysis for decarbonylative and acyl couplings, as well as a fluorine source.^5–10^ Of note, acyl fluorides have been utilized for peptide coupling and general amide bond formation because they result in reduced epimerization and side reactivity in solid phase peptide synthesis (SPPS).^2,11^ Conventional methods for the synthesis of acyl fluorides include deoxyfluorination of carboxylic acids with sulfur trifluoride reagents, transition metal-catalyzed fluorocarbonylation from organic halides with CO, and acyl exchange with carbonyl derivatives with transition metal catalysts and fluoride salts (Scheme 1A).^3,11^ Most procedures featuring acyl fluorides for the synthesis of amides are accomplished in a one-pot deoxyfluorination of carboxylic acids with a commercial or synthesized fluorination source in the presence of an amine nucleophile.^1,12–17^ However, these approaches often suffer from the employment of harsh reaction conditions, the use of costly reagents, and limited functional group tolerance.^18–20^ Broader scope and column-free purification have been achieved with the employment of toxic thionyl fluoride gas.^1,21–23^ Photochemical processes, which have been leveraged to construct complex bonds under mild, sustainable, and more selective conditions, have also been applied to the formation of acyl fluorides, including blue light-driven fluorocarbonylation^24,25^ and UV-light activated C­(sp^3^)–H abstraction.^26–28^ The variety of these creative approaches renders this space a promising alternative to traditional methods.

**Scheme 1:**
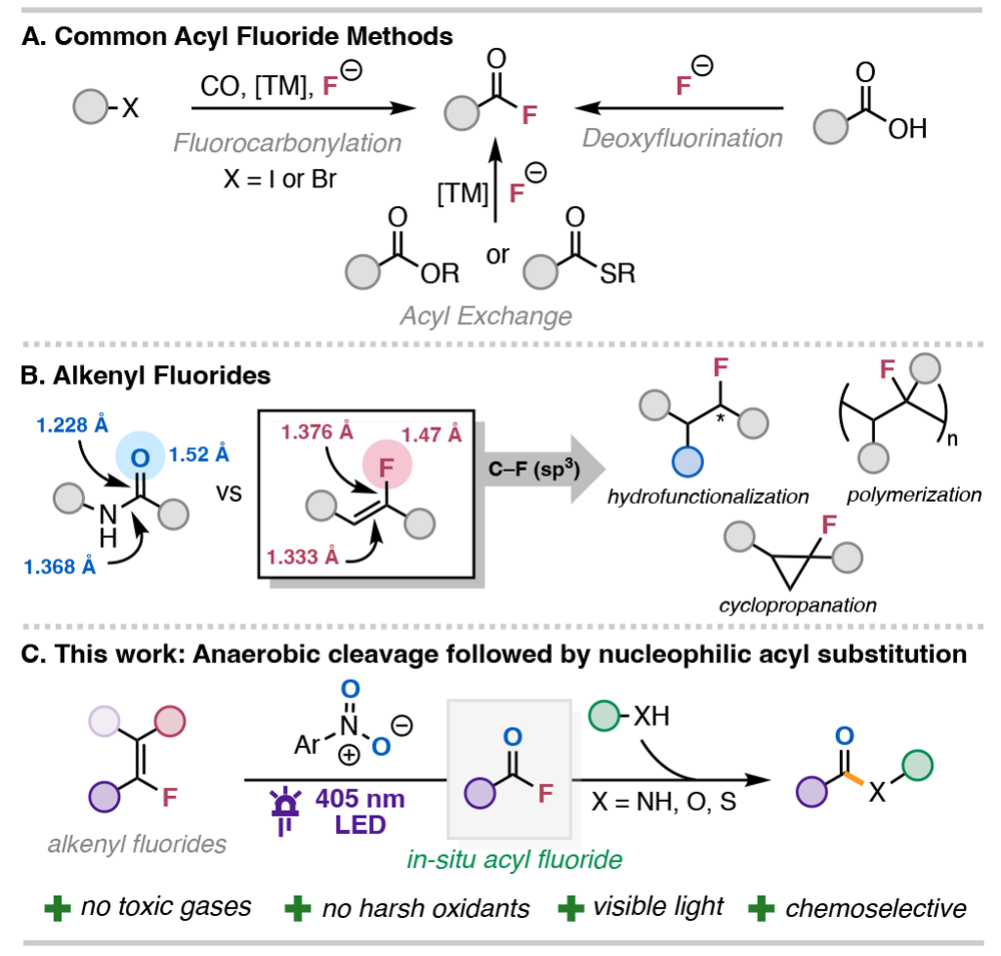
Acyl Fluoride Synthesis and Alkenyl Fluoride Utility

Alkenyl fluorides are privileged functional groups in medicinal chemistry because of their capacity to serve as amide bioisosteres and precursors to valuable C­(sp^3^)–F motifs (Scheme 1B).^29–32^ Due to their versatility, several approaches for accessing these powerful synthons have been reported.^33–35^ Seminal work by Kuczkowski and Gilles illustrated that alkenyl fluorides could serve as precursors to acyl fluorides via ozonolysis.^36–40^ However, their work was focused on mechanistic understanding and limited to the employment of lightweight alkenyl fluorides, and the formation of several cross-ozonide byproducts was observed.^38^ Moreover, the safety hazards associated with ozonolysis have deterred widespread implementation of this approach.^41^

Recently, photoexcited nitroarenes have been showcased as effective ozone surrogates to enable a safe and practical ozonolysis alternative.^42–46^ Hence, we envisioned that photoinduced cleavage of alkenyl fluorides could be leveraged to access acyl fluorides under mild and anaerobic conditions. The formed acyl fluorides can undergo *in situ* coupling with exogenous nucleophiles such as amines, alcohols, and thiols to furnish valuable amides, esters, and thioesters, respectively (Scheme 1C).^14,18^

To test this hypothesis, we studied the cleavage of 1-(1-fluorovinyl)-4-methylbenzene (**1a**) leading to acyl fluoride **1a′** under previously reported conditions (Table 1, entry 1). The desired product was obtained in a 31% NMR yield. A wavelength screening indicated that 405 nm light was most effective, resulting in 38% NMR yield of acyl fluoride **1a′** (Table 1, entry 2). After an extensive screening of nitroarenes, the use of 4-nitrophthalonitrile resulted in the highest yield of **1a′** in 76% (Table 1, entry 4). Notably, the hydrolytically stable acyl fluoride products can be directly isolated (*vide infra*). Control studies indicated that constant photoirradiation is required for transformation (Table 1, entry 5). Since acyl fluorides are often used as key reactive intermediates in synthetic reactions, we postulated that the acyl fluoride formed under our reaction conditions could undergo nucleophilic acyl substitution (NAS) with exogenous nucleophiles. It was found that the addition of hexylamine led to a 53% NMR yield of *N*-hexyl-4-methylbenzamide **2a** (Table 1, entry 7). We observed that a two-step, one-pot procedure was required, where the nucleophile was added after the cleavage event in the absence of light to avoid the formation of electron donor–acceptor (EDA) complexation and/or the quenching of the excited triplet state of the nitroarene.^47^ The one-pot protocol also allows for the efficient conversion of acyl fluoride without the loss of material due to stepwise purification. The remaining mass balance was attributed to the side reactivity of the carbonyl imine intermediate and/or reduced nitroarene after the cleavage event (see Supporting Information (SI)). To suppress the formation of these byproducts, we used a sacrificial aldehyde to react with the carbonyl imine and aniline, which led to an increased NMR yield of 71% (Table 1, entry 8) and an isolated yield of 50% for **2a** (Table 1).

**Table 1:**
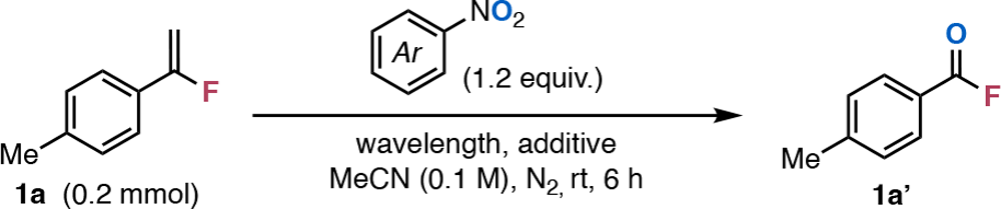
Optimization Studies

**entry**	**nitroarene**	**wavelength**	**additive**	**1a′** yield, %
1	4-CN	390 nm	none	31%
2	4-CN	405 nm	none	38%
3	4-CN	427 nm	none	29%
4	3,4-diCN	405 nm	none	76%
5	3,4-diCN	Dark	none	NR
6	none	405 nm	none	NR
7	3,4-diCN	405 nm	hexylamine^b^	0% (53%)^c^
**8**	**3,4-diCN**	**405 nm**	^***t***^ **BuCHO** ^d^ + **hexylamine** ^b^	**0% (71%)** ^c^

a ^19^F NMR yield using fluorobenzene as an internal standard.

b 1.5 equiv.

c ^1^H NMR yield of amide product (**2a**) using CH_2_Br_2_ as an internal standard.

d 0.5 equiv.

The scope was then investigated with styrenyl substrates (Table 2). We evaluated electron-rich *para*-substituted styrenyl fluorides and observed that *tert*-butyl and methoxy resulted in similar amide isolations of 46% and 53%, respectively (**2b**–**2c**). Electron neutral (**1d**) and poor (**1e**) substrates produced similar yields, demonstrating that there is a negligible electronic effect at the para-position. **1f** was subjected to photochemical conditions without amine addition, which resulted in the isolation of acyl fluoride **1′f** in 46% yield. After isolation of **1′f**, NAS with N-hexylamine led to a 40% yield over two steps, a slightly lower yield compared to our one-pot procedure (**2f**) (see SI). *Meta*-substituted styrenes resulted in amide products **2g** and **2h** in good yield. However, a lower yield was observed with *ortho*-substituents (**2i**) due to steric effects shutting down efficient starting material conversion. Tetra-substituted styrenes possessing sensitive carbonyl functionalities such as carboxylic acid and esters performed efficiently under the reaction conditions, resulting in 58% and 46% yields of **2k** and **2l**, respectively. Substrate **1m** containing sensitive benzylic alcohol generated **2m** in modest yield. Exposure of 3-fluoro-1*H*-indene to the reaction conditions resulted in isoquinolinone **2n** via intramolecular condensation from the formed amide and aldehyde.

**Table 2:**
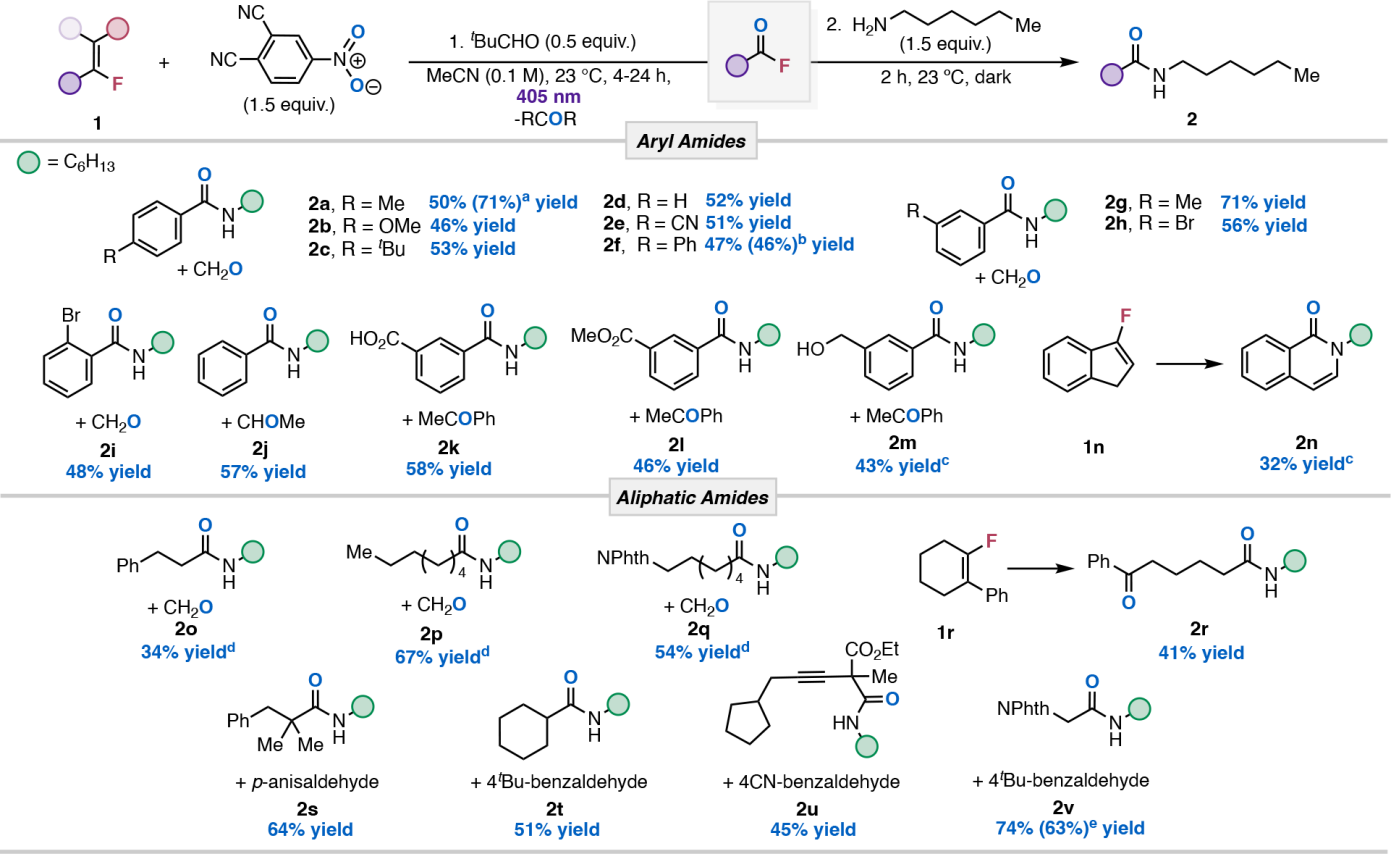
Reaction Scope of Tandem Cleavage and NAS of Alkenyl Fluorides

a Denotes ^1^H NMR yield using CH_2_Br_2_ as an internal standard.

b Denotes isolated yield of acyl fluoride **1′f**.

c Denotes modified protocol (see SI)

d Denotes conditions using 2.0 equiv of nitroarene, EtOAc (0.2 M), 390 nm, 48 h.

e Denotes 1.0 mmol scale.

Next, the scope of unactivated alkenyl fluorides was investigated under our protocol. Minor reoptimization was required, leading to the use of EtOAc (0.2 M) and 390 nm light. Monosubstituted alkenyl fluorides (**1o**–**1q**) produced the corresponding aliphatic amide **2o**–**2q** in good yield. Overall, unactivated substrates struggled with longer reaction times and poor conversion. To increase the efficiency for aliphatic amide formation, we hypothesized that appending an aromatic ring to the β-position of the aliphatic alkenyl fluoride (i.e., β-fluorostyrene) would provide shorter reaction times and higher yields in our transformation. Consequently, this strategy reduced the reaction time by 8-fold and resulted in increased yields of the aliphatic amide products. Cyclohexyl alkenyl fluoride (**1r**) underwent cleavage and successive NAS to yield keto amide **2r** in 41% yield. Unsurprisingly, attaching electron-rich aromatics to the acyclic aliphatic alkenyl fluorides **1s** and **1t** gave high yields of desired amide products (**2s**–**2t**). Markedly, internal alkyne in substrate **1u** did not undergo competitive reactivity,^32^ as amide **2u** was obtained chemoselectively in modest yield. While alkynes showed no reactivity under these conditions, styrenyl alkenes competed effectively with the alkenyl fluoride (Figure S1), and aliphatic alkenes, although less reactive, exhibited minor but noticeable conversion (Figure S5). We also tested the scalability of our photochemical setup by increasing the reaction scale 5-fold with **2v**. It was found that the isolated yield (64%) was comparable to that of our benchmark scale (74%).

Next, we tested a variety of different nucleophiles for our transformation with **1a** to access amide derivatives, esters, and thioesters (Table 3). Primary amines 1,2,3,4-tetrahydro-1-naphthylamine and *N*,*N*-dimethyl-*p*-phenylenediamine were tested and resulted in the corresponding amide products (**3a**–**3b**) in good yields. Expectedly, we found that primary and secondary amines coupled well with the acyl fluorides without the need for base additives, except when electron-deficient amines were used (**3c**, 18% yield). Subjecting *N*,*O*-dimethylhydroxylamine to the reaction conditions resulted in Weinreb amide **3d** in modest yield. Secondary amine diethanolamine showed complete selectivity for amide coupling (**3e**) over the glycol alcohol nucleophiles in 53% yield. Substrate **3f** resulted from the efficient coupling of desloratadine, a known antihistamine, with 4-methylbenzoyl fluoride under our transformation. All alcohol and thiol nucleophiles required base additives, such as TEA and DMAP, to drive the NAS reaction to completion. Vanillin underwent smooth coupling to give 40% of ester **3g**. The sterically hindered quinine coupling partner furnished **3h** in 28% yield, with the low yield being attributed to poor solubility. Due to polarity overlap with nitroarene byproducts, **3h** was obtained using a modified protocol in which the byproducts were precipitated with ether prior to the coupling step (see SI). Thiols, including hexanethiol and adamantathiol, reacted well upon the addition of both TEA (1.1 equiv) and DMAP (0.3 equiv), resulting in thioesters **3i** (40%) and **3j** (41%). Amino acid nucleophiles, such as *tert*-butyl leucine, serine, and cysteine, were tested to highlight the utility of our protocol in peptide coupling reactions. Coupling of *tert*-butyl leucine ester resulted in **3k** in 52% yield. Methyl ester *N*-Boc-serine required a longer reaction time and higher quantities of base additives for the NAS event; **3l** was obtained under the reaction conditions in 40% yield. Finally, the employment of the methyl ester *N*-Ac-cysteine as an amino acid nucleophile resulted in a 56% yield of the thioester **3m**.

**Table 3:**
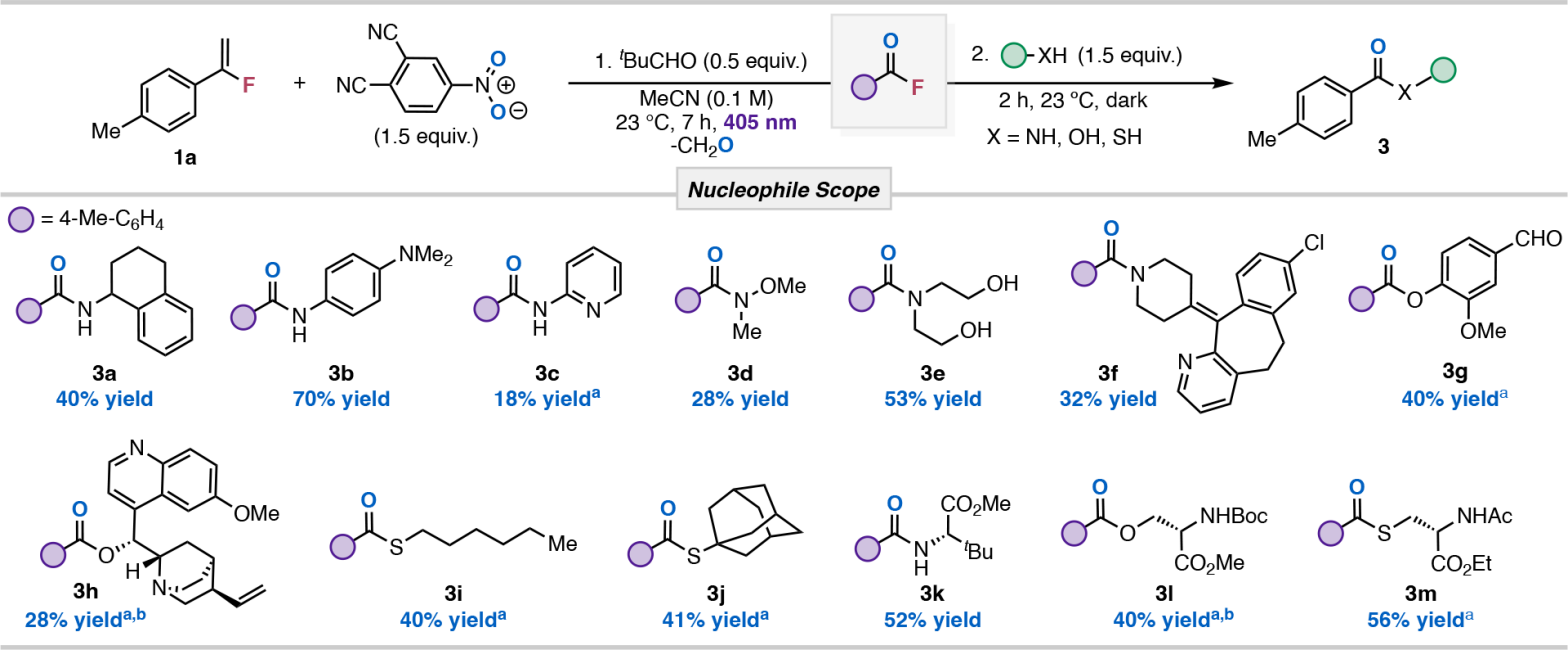
Nucleophile Scope

a Denotes base additive such as triethylamine (1.1 equiv) or DMAP (0.3 equiv).

b Denotes modified protocol (see SI).

With the success of coupling amino acid nucleophiles, we posited that linear peptides could be synthesized under our methodology. Existing protocols for NAS of peptides are often limited to carboxylate activating agents, bulky leaving groups, and base additives.^48,49,50^ The use of alkenyl fluorides as NAS precursors can allow for selective NAS for late-stage modification when other sensitive carboxylic acid derivatives are featured. Classical ozonolysis, however, is not often used in peptide synthesis since it is incompatible with peptide residues such as tyrosine, tryptophan, cysteine, serine, histidine, etc., which will undergo rapid oxidation.^51^ To illustrate the synthetic utility of our approach (Table 4), we subjected **1v** for successive cleavage and NAS with *tert*-butyl tyrosine methyl ester, which yielded the dipeptide NPhth-Gly-Tyr­(*t*-Bu)-OMe (**5a**) in 55% yield. Next, we synthesized a masked dipeptide **4b** that was coupled with a l-tryptophan methyl ester to furnish tripeptide NBoc-Phe-Gly-Trp-OMe (**5b**) in 54% yield. Notably, the Boc-protected phenylalanine did not undergo benzylic C–H oxidation under our reaction conditions.^52,53,49^ Our method was extended to a five-amino acid (**4c**) linear peptide sequence with a masked glycine fluoroalkene attached to the C-terminus. Heptamer **5c** was isolated in 38% yield, showcasing a mild method for complex substates featuring heterocycles (proline, tryptophan). The benefit of this method over the prior art is the ability to install the desired peptide bond with high regioselectivity and chemoselectivity at the alkenyl position.^54^ Acetylated β-d-Glucopyranose was successfully coupled with the aid of base additives to produce ester **5d** in 51% yield. Similar substructures of glucopyranosyl esters have been evaluated as opioid agonists.^55^

**Table 4:**
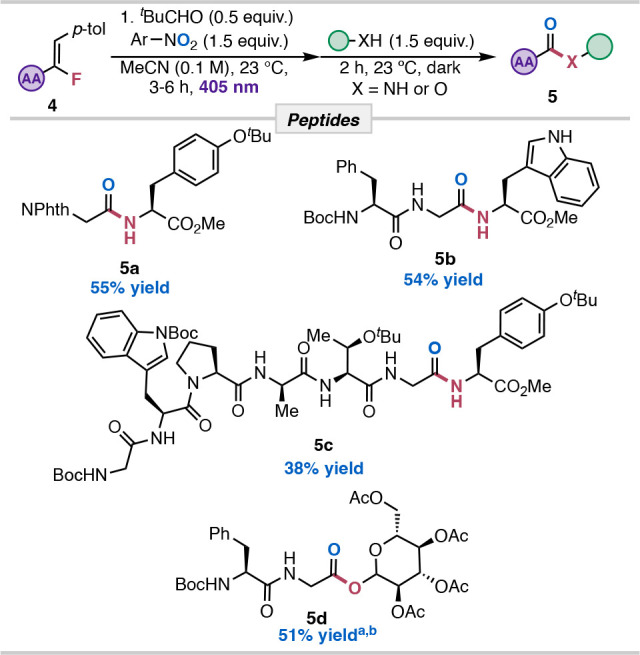
Chemical Biology Applications

a Denotes base additive used triethylamine (2.0 equiv) and DMAP (0.5 equiv).

b Denotes ^1^H NMR yield using CH_2_Br_2_ as an internal standard.

In conclusion, we have developed a mild, anaerobic method using photoexcited nitroarenes to cleave alkenyl fluorides to produce synthetically valuable acyl fluorides without the use of toxic or costly reagents.^56^ This approach tolerates sensitive functional groups and enables in situ nucleophilic substitution to access amides, esters, and thioesters. As the first practical route to acyl fluoride from alkenyl moieties, this method opens up new possibilities in medicinal chemistry and chemical biology.

## Supplementary Material



## Data Availability

The data underlying this study are available in the published article and its Supporting Information.

## Notes

Experimental details, optimization studies, characterization data, and NMR spectra (PDF)

## Abbreviations

NAS: nucleophilic acyl substitution
SPPS: solid-phase peptide synthesis
DMAP: 4-dimethylaminopyridine
TEA: triethylamine
NMR: nuclear magnetic resonance
UV: ultraviolet
