# Supplementary material for: Photoinduced Cleavage of Alkenyl Fluorides for Nucleophilic Acyl Substitution via In Situ Generated Acyl Fluorides
Source: Org Lett. 2025 Nov 25;27(48):13223–8. doi: 10.1021/acs.orglett.5c04085 (PMC12687311; doi:10.1021/acs.orglett.5c04085)
Supplement: Supplementary file 1 [file ol5c04085_si_001.pdf]

Photoinduced Cleavage of Alkenyl Fluorides for Nucleophilic Acyl Substitution via In Situ Generated Acyl Fluorides

Emma S. Gogarnoiu, Melissa S. Griffin, Ajay H. Bansode,[‡] Joshua M. Paolillo,[‡] Joseph M. Bergen, and Marvin Parasram*

Department of Chemistry, New York University, 24 Waverly Place, 3rd floor, New York, NY 10003

Supporting Information

General information	1
Optimization of the reaction parameters	2
General procedures	6
Substrate synthesis	7
Characterization of oxidative cleavage products	14
Mechanistic studies	26
Peptide substrate synthesis and cleavage products	29
NMR spectra of substrates	42
NMR spectra of products	69
References	110

General information

All reactions were carried out in oven-dried glassware under a nitrogen atmosphere, unless otherwise stated. Solvents were dried and deoxygenated by passing through alumina in a solvent purification system. $CDCl_3$, DMSO-d_6, CD3OD, and CD_3CN were purchased from Cambridge Isotope Laboratories.

All NMR spectra (^1H, ^{13}C, and ^{19}F) were recorded on Bruker 400 MHz, 500 MHz, 600 MHz, and 800Mz Avance spectrometer. Spectral data was acquired at 298 K. The chemical shifts (δ) are given in parts per million (ppm) and referenced to residual solvent peaks for ^1H and ^{13}C NMR spectra: $CDCl_3$, δ_H 7.26 ppm, δ_C 77.16 ppm; DMSO-d_6, δ_H 2.50 ppm, δ_C 39.52 ppm; CD3OD δ_H 4.87 ppm, δ_C 49.00 ppm; CD_3CN, δ_H 1.94 ppm, δ_C 1.32, 118.69 ppm. Coupling constants (*J*) are reported in Hertz (Hz) to the nearest 0.1 Hz. GC chromatograms were taken on an Agilent 8890 GC with 5977B MSD, and helium as the carrier gas. High-resolution mass spectra (HRMS) were obtained on a Waters ACQUITY/Xevo G3 LC-QToF MS which was acquired through the support of New York University. We utilized 34 W Kessil Lamps with varying wavelengths as well as 18 W EvoluChem 405 nm LEDs for our photochemical set ups.

Optimization of the reaction parameters

Table S1: Wavelength Screen[a]

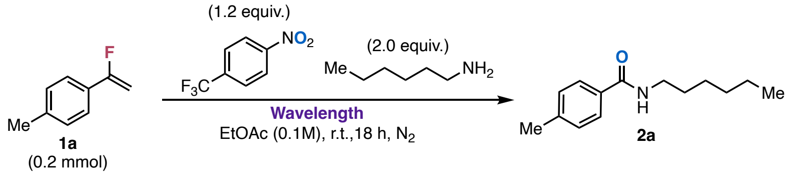

entry	Wavelength	Conversion, %	2a yield, %[b]
1	390 nm	90	41
2	405 nm	94	37
3	427 nm	20	27
4	440 nm	44	21
5	456 nm	10	3

[a]Reactions were conducted on a 0.20 mmol scale. [b]Yield determined by ^1H NMR using CH_2Br_2 as external standard.

Table S2: Optimization of Nitroarenes[a]

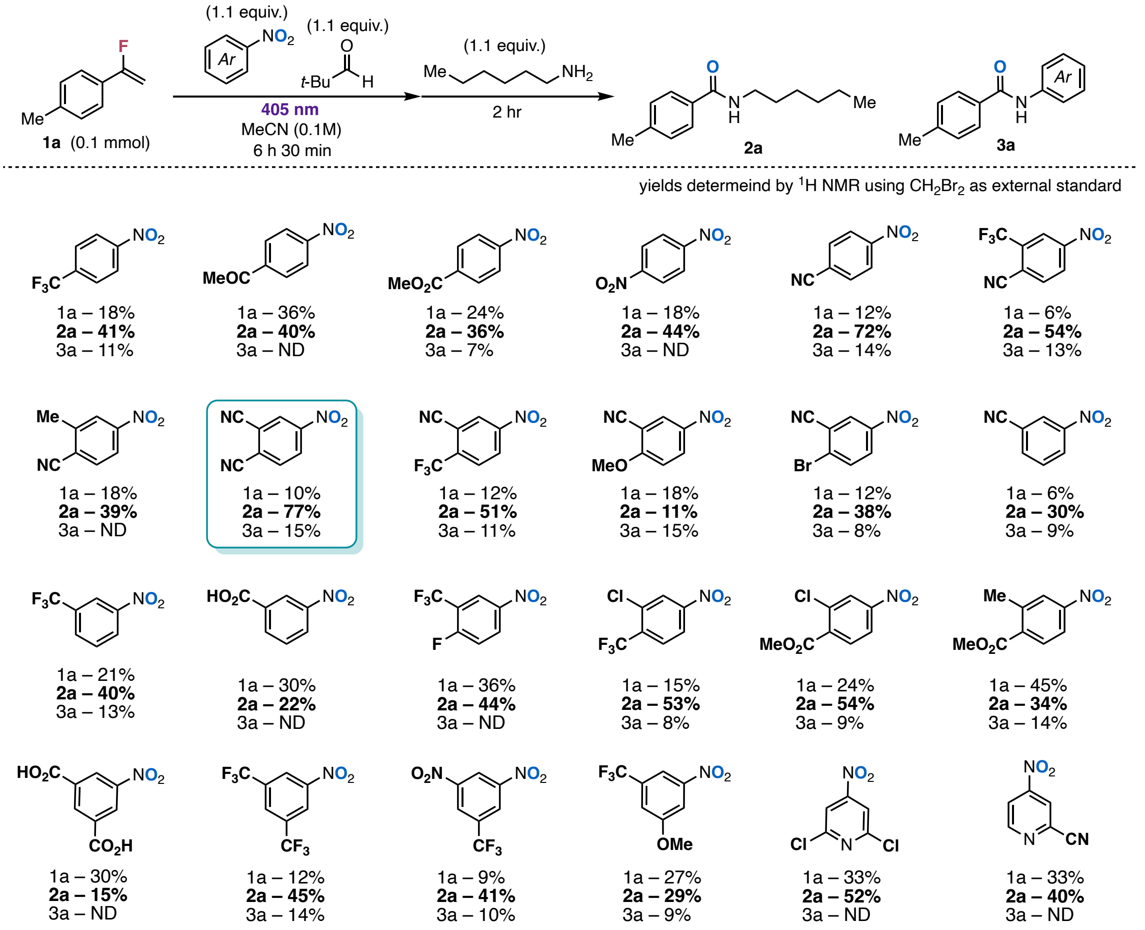

[a]Reactions were conducted on a 0.10 mmol scale.

Table S3: Time Course of Styrenyl Substrate[a]

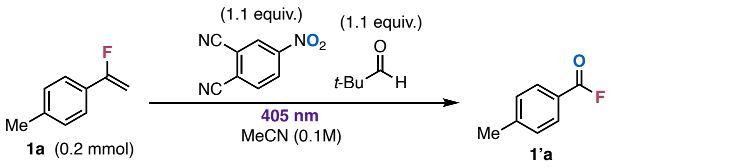

yields determeind by ^{19}F NMR using PhF as internal standard

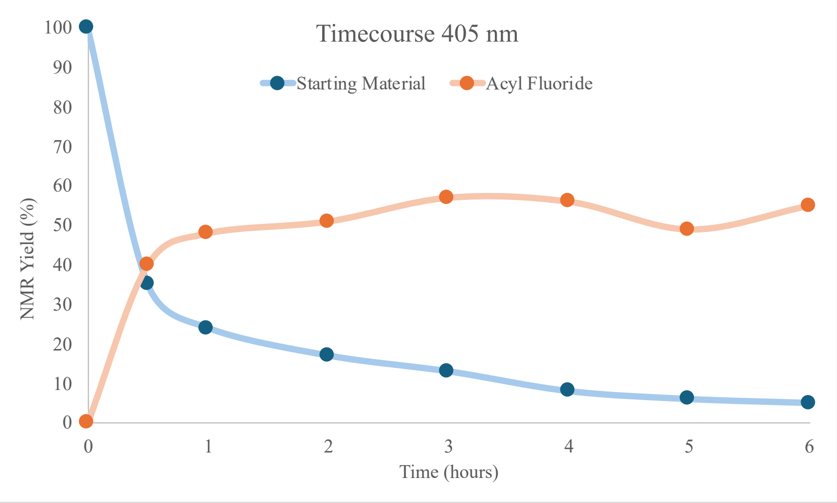

[a]Reactions were conducted on a 0.20 mmol scale.

Table S4: Optimization of Solvent[a]

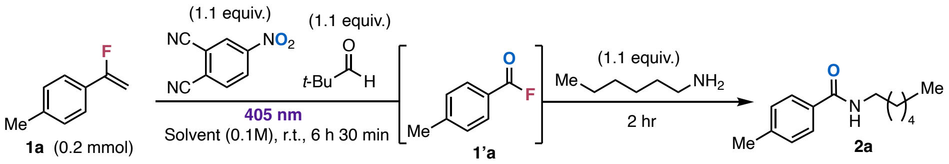

entry	Solvent	1'a yield, %[b]	2a yield, %[c]
1	PhH	19	—
2	DCE	11	—
3	DCM	30	—
4	NO_2CH_3	32	32
5	DMC	42	42
6	EtOAc	59	40
7	$EtOAc_{(anhydrous)}$	50	62
8	MeCN	54	65

[a]Reactions were conducted on a 0.20 mmol scale. [b]Yield determined by ^{19}F NMR using PhF as external standard [c]Yield determined by ^{1}H NMR using CH_2Br_2 as external standard.

Table S5: Acetaldehyde Equivalence Screen[a]

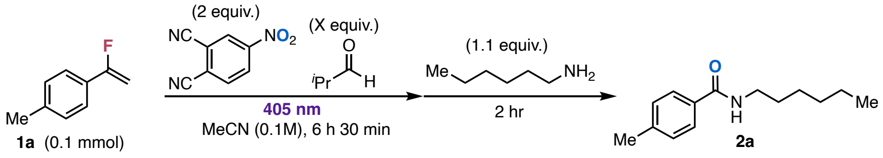

entry	acetaldehyde equivalence	2a yield, %[b]
1	0.50	80
2	1.00	78
3	1.25	76
4	1.50	67
5	2.00	76

[a]Reactions were conducted on a 0.10 mmol scale. [b]Yield determined by ^1H NMR using CH_2Br_2 as external standard

Table S6: Nitroarene Equivalence Screen[a]

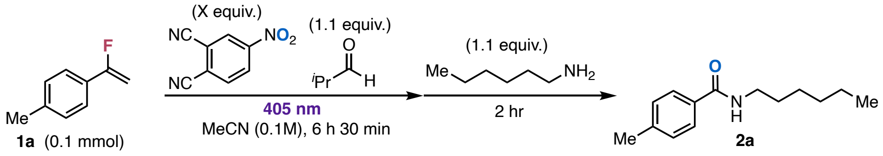

entry	NO_2 equivalence	2a yield, %[b]
1	1.10	87
2	1.50	77
3	2.00	88

[a]Reactions were conducted on a 0.10 mmol scale. [b]Yield determined by ^1H NMR using CH_2Br_2 as external standard

Table S7: Concentration Screen for Aliphatic Substrate

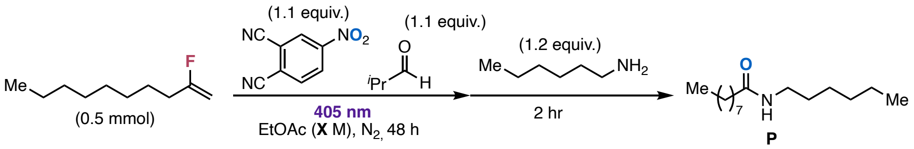

entry	Concentration	P yield, %[b]
1	0.20 M	38
2	0.33 M	26
3	0.40 M	35
4	0.50 M	21

[a]Reactions were conducted on a 0.50 mmol scale. [b]Yield determined by ^1H NMR using CH_2Br_2 as external standard

Table S8: Temperature Optimization for Aliphatic Substrates

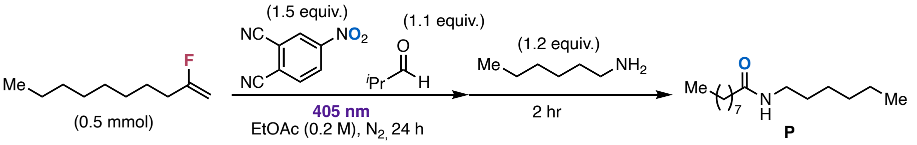

entry	Temperature (step 1)	Temperature (step 2), °C	Deviation from conditions	P yield, %[b]
1	0 °C	60 °C	——	48
2	0 °C	25 °C	——	33
3	30 °C	25 °C	——	30
4	30 °C	25 °C	MeCN	45
5	25 °C	80 °C	MeCN	40
6	30 °C	25 °C	NO_2 (2 equiv.), 390 nm, 48 h	53

[a]Reactions were conducted on a 0.50 mmol scale. [b]Yield determined by ^{1}H NMR using CH_2Br_2 as external standard

Table S9: Dark control Study

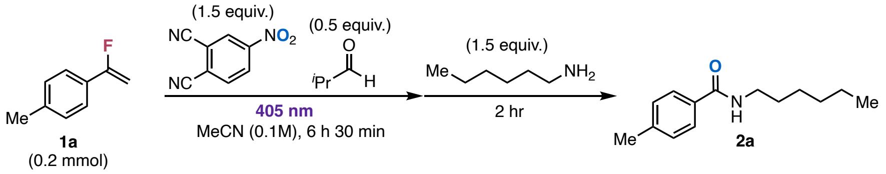

entry	Deviation from conditions	2a yield, %[b]
1	dark, 25 °C	0
2	dark, 40 °C	0

[a]Reactions were conducted on a 0.50 mmol scale. [b]Yield determined by ^{1}H NMR using CH_2Br_2 as external standard

General procedures

General Procedure A: Suzuki-Miyaura Reaction with Iodonium BF_4 Salts

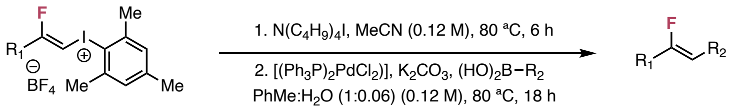

Procedure was adapted from literature report.[1] To a glass vial equipped with a stirrer bar, was added (Z)-iodonium BF_4 (1 equiv.), tetrabutylammonium iodide (1.1 equiv.), and MeCN (0.12 M). The suspension was stirred at 80 ºC using a heating block for 6 hours and the solvent was evaporated under reduced pressure. $[(Ph_3P)_2PdCl_2]$ (0.075 equiv.), boronic acid (2.5 equiv.), and K_2CO_3 (2.5 equiv.) were added. The vial was resealed with a septum and flushed with nitrogen before adding degassed toluene:water (1:0.06) (0.12 M). The mixture was stirred at 80 °C for 18 hours and saturated aqueous solution of ammonium chloride was added. The aqueous and organic layers were separated, and the aqueous phase was extracted with CH_2Cl_2 (3 x). The combined organic layers were evaporated under reduced pressure, and the resulting crude mixture was subjected to flash-column chromatography.

General Procedure B: Oxidative Cleavage Standard Procedure

4-nitrophthalonitrile (1.5 equiv.) & fluoroalkene (1 equiv.) was added to a 7.5 mL borosilicate glass scintillation vial (flame dried or oven dried). The reaction was put under vacuum for 1 min, then purged with N_2 flow (3 x) followed by the addition of anhydrous acetonitrile (0.1 M) or ethyl acetate (0.2 M). The reaction vial was placed 7.62 -10.16 cm in front of a 405 nm lamp or 390 nm lamp with a cooling fan. The reaction was stirred under irradiation until consumption of starting material as determined by TLC or GC-MS analysis. The light is subsequently turned off and an amine nucleophile (1.5 equiv.) was added to the reaction mixture. The reaction is stirred without irradiation until acyl fluoride intermediate is consumed (~2 h). After completion solvent was removed *in vacuo*. The residue was dissolved in deuterated chloroform and NMR standard dibromomethane (CH_2Br_2) (1 equiv.) was added. Crude ^1H NMR yield was taken. After NMR yield was determined, solvent was evaporated, and the crude residue was purified by column chromatography to give desired coupling product.

Note: For styrenyl substrate = MeCN, 405 nm. For aliphatic substrates = EtOAc, 390 nm
Reactions were also set up in duplicate and combined after NMR yields are taken for isolated yield.

Substrate synthesis

Literature Reported

(*Z*)-(2-fluoro-2-phenylvinyl)(mesityl)iodonium tetrafluoroborate[1], methyl 3-(prop-1-yn-1-yl)benzoate[2], 1-(2,2-difluorovinyl)-4-methoxybenzene[3], 4-(2,2-difluorovinyl)benzonitrile[3], 1-(*tert*-butyl)-4-(2,2-difluorovinyl)benzene[4], ethyl 5-cyclopentyl-2-methylpenta-2,3-dienoate[5], 1-(1-fluorovinyl)-4-methylbenzene **(1a)**[6], 1-(1-fluorovinyl)-4-methoxybenzene **(1b)**[7], 1-(*tert*-butyl)-4-(1-fluorovinyl)benzene **(1c)**[6], (1-fluorovinyl)benzene **(1d)**[6], 4-(1-fluorovinyl)benzonitrile **(1e)**[8], 4-(1-fluorovinyl)-1,1'-biphenyl **(1f)**[7], 1-(1-fluorovinyl)-3-methylbenzene **(1g)**[9], 1-bromo-2-(1-fluorovinyl)benzene **(1i)**[9], (*Z*)-(1-fluoroprop-1-en-1-yl)benzene **(1j)**[10], (3-fluorobut-3-en-1-yl)benzene **(1o)**[11], 2-fluorooct-1-ene **(1p)**[12], 6-fluoro-2,3,4,5-tetrahydro-1,1'-biphenyl **(1r)**[13], ethyl (*Z*)-2-(2-(4-cyanophenyl)-1-fluorovinyl)-5-cyclopentyl-2-methylpent-3-ynoate **(1u)**[14], were synthesized according to the literature.

Substrate synthesis

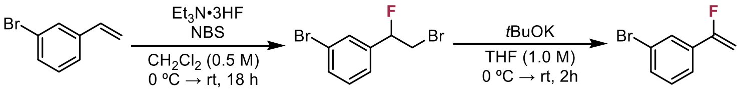

1h

1-bromo-3-(1-fluorovinyl)benzene (1h)

Adapted procedure from literature.[6] To a flame-dry flask, N-bromosuccinimide (4.00 g, 22.5 mmol, 1.5 equiv.) and 1-bromo-3-vinylbenzene (2.0 mL, 15.0 mmol) were dissolved in anhydrous CH_2Cl_2 (30.0 mL, 0.5 M). Triethylamine trihydrofluoride (3.67 mL, 22.5 mmol, 1.5 equiv.) was carefully added at 0 °C and the reaction mixture was stirred 18 h at room temperature. The reaction was quenched with saturated aq. $NaHCO_3$ at 0 °C and extracted with CH_2Cl_2. The combined organic layer was washed with 1N HCl and then concentrated *in vacuo*. The residual crude was filtered through a silica plug with n-pentane to afford 1-bromo-3-(2-bromo-1-fluoroethyl)benzene, which was used for the next step without further purification. In a flame dried flask, 1-bromo-3-(2-bromo-1-fluoroethyl)benzene was dissolved in anhydrous THF (15.0 mL, 1.0 M), and the solution was cooled to 0 °C. Potassium tert-butoxide (1.68 g, 15.0 mmol, 1.0 equiv.) was added, then the reaction mixture was warmed to room temperature and stirred 2 hr. The reaction was quenched with water, extracted with *n*-pentane, dried over Na_2SO_4 and carefully concentrated *in vacuo*. The obtained crude was purified by column chromatography (100% hexanes) to afford **1h** as a colorless oil (1.20 g, 40% yield).

^{1}H NMR (500 MHz, $CDCl_3$) (δ, ppm): 7.72 (d, J = 1.9 Hz, 1H), 7.51 (ddd, J = 7.1, 4.8, 1.7 Hz, 2H), 7.28 (t, J = 4.0 Hz, 1H), 5.08 (dd, J = 49.2, 3.7 Hz, 1H), 4.93 (dd, J = 17.6, 3.7 Hz, 1H).

^{13}C{^{1}H} NMR (126 MHz, $CDCl_3$) (δ, ppm): 161.6 (d, J_{C-F} = 250.8 Hz), 134.1 (d, J_{C-F} = 29.8 Hz), 132.5, 130.2 (d, J_{C-F} = 2.2 Hz), 127.8 (d, J_{C-F} = 7.3 Hz), 123.3 (d, J_{C-F} = 6.8 Hz), 122.8 (d, J_{C-F} = 2.3 Hz), 91.0 (d, J_{C-F} = 22.1 Hz).

^{19}F NMR (471 MHz, $CDCl_3$) (δ, ppm): -108.03 (dd, J = 49.2, 17.6 Hz).

LRMS (ESI): calcd. for C_8H_6BrF $[M]^+$ 199.96, found 199.90

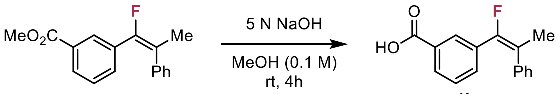

1k

(E)-3-(1-fluoro-2-phenylprop-1-en-1-yl)benzoic acid (1k)

Adapted from literature.[15] A 5 N aqueous NaOH solution (74.0 µL, 370 µmol, 1 equiv.) was added to a solution of methyl (E)-3-(1-fluoro-2-phenylprop-1-en-1-yl)benzoate (100 mg, 370 µmol) in MeOH (3 mL, 0.1 M) at room temperature for 4 h. 1 N HCl (4.5 mL) and H_2O were

then added, followed by extraction with EtOAc (2 x). The organic layers were combined and sequentially washed with H_2O and saturated brine, and then dried over Na_2SO_4. Concentrated under reduced pressure gave to afford **1k** as a white solid (82 mg, 87% yield (E:Z= 1:0.4)).

^{1}H NMR (500 MHz, CD_3OD) (δ, ppm): 7.86 – 7.82 (m, 2H), 7.40 (t, J = 7.7 Hz, 1H), 7.26 (dddd, J = 12.9, 11.2, 6.2, 4.1 Hz, 5H), 7.19 – 7.14 (m, 2H), 2.18 (d, J = 3.9 Hz, 3H), 2.04 (s, 1H).
^{13}C{^{1}H} NMR (126 MHz, CD_3OD) (δ, ppm): 169.1, 153.9 (d, J_{C-F} = 243.2 Hz), 140.9 (d, J_{C-F} = 7.7 Hz), 136.6, 134.6, 134.4, 133.6 (d, J_{C-F} = 5.2 Hz), 133.5, 131.9, 130.4, 130.4, 129.9 (d, J_{C-F} = 3.1 Hz), 129.8, 129.7, 129.6 (d, J_{C-F} = 16.1 Hz), 129.0, 128.5, 126.0, 119.5 (d, J_{C-F} = 21.3 Hz), 87.6, 79.5, 17.8 (d, J_{C-F} = 7.8 Hz).
^{19}F NMR (471 MHz CD_3OD) (δ, ppm): -103.77 (d, J = 4.1 Hz).
HRMS (ESI TOF): calcd. for $C_{16}H_{14}O_2F$ [M+H]$^+$ 257.0978, found 257.0988

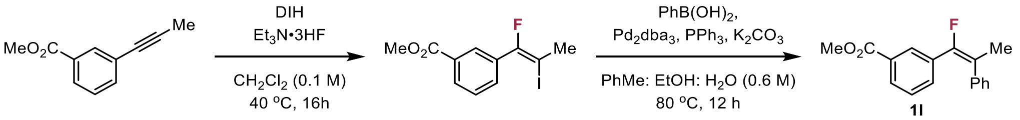

methyl (E)-3-(1-fluoro-2-phenylprop-1-en-1-yl)benzoate (1l)
Adapted from literature.[15] In a Schlenk flask flushed with nitrogen, a solution of methyl 3-(prop-1-yn-1-yl)benzoate (500 mg, 2.87 mmol, 1 equiv.) and 1,3,-diiodo-5,5-dimethylhydantoin (DIH) (1.09 g, 2.87 mmol, 1 equiv.) in CH_2Cl_2 (28 mL, 0.1 M) was prepared. Triethylamine trihydrofluoride (1.40 mL, 8.61 mmol, 3 equiv.) was added before heating to 40 °C in an oil bath for 16 h. The reaction was brought to room temperature and diluted with CH_2Cl_2 before quenched with saturated aq. $NaHCO_3$. The layers were separate, and the aqueous layer was extracted with CH_2Cl_2. The combined organic layer was dried over Na_2SO_4, and volatiles were removed *in vacuo*. Next step proceeded without further purification. Phenylboronic acid (525 mg, 4.31 mmol, 1.5 equiv.), [$Pd_2(dba)_3$] (131 mg, 144 μmol, 5.0 mol%), PPh_3 (151 mg, 574 μmol, 20 mol%), K_2CO_3 (1.19 g, 8.61 mmol, 3 equiv.) as well as degassed (three freeze-pump-thaw cycles) toluene (28 mL), EtOH (8.4 mL), and H_2O (8.4 mL) were added to the filtrate. The mixture was heated to 80 °C in an oil bath for 12 h, cooled to room temperature and diluted with CH_2Cl_2 and H_2O. The layers were separated and the aqueous layer was extracted with CH_2Cl_2. The combined organic layers were dried over Na_2SO_4 and volatiles were removed *in vacuo*. The crude residue was purified via flash column chromatography (0-5% EtOAc/Hexanes) to afford **1l** as a colorless oil (0.59 g, 73% yield (E:Z= 1:0.4)).

^{1}H NMR (500 MHz, $CDCl_3$) (δ, ppm): 7.89 (d, J = 1.9 Hz, 1H), 7.84 (dt, J = 7.5, 1.6 Hz, 1H), 7.35 (t, J = 7.8 Hz, 1H), 7.30 – 7.21 (m, 5H), 7.19 (d, J = 7.6 Hz, 1H), 7.15 (dd, J = 7.7, 1.9 Hz, 2H), 3.91 (s, 1H), 3.85 (s, 3H), 2.20 (d, J = 3.9 Hz, 3H), 2.06 (s, 1H).
^{13}C{^{1}H} NMR (126 MHz, $CDCl_3$) (δ, ppm): 166.7, 166.7, 152.5 (d, J_{C-F} = 243.9 Hz), 139.7 (d, J_{C-F} = 7.8 Hz), 135.8, 133.3 (d, J_{C-F} = 29.9 Hz), 132.8, 132.7, 132.6 (d, J_{C-F} = 5.6 Hz), 130.4, 130.1, 129.3, 129.3, 129.2, 128.9 (d, J_{C-F} = 3.1 Hz), 128.8, 128.7, 128.6, 128.5, 128.4, 128.0, 127.4, 124.6, 118.4 (d, J_{C-F} = 21.9 Hz), 87.1, 79.0, 61.2, 52.3, 52.2, 18.0, 17.9 (d, J_{C-F} = 7.5 Hz), 14.4.
^{19}F NMR (471 MHz, $CDCl_3$) (δ, ppm): -106.20 (d, J = 4.1 Hz).
HRMS (ESI TOF): calcd. for $C_{17}H_{16}O_2F$ [M+H]$^+$ 271.1134, found 271.1137

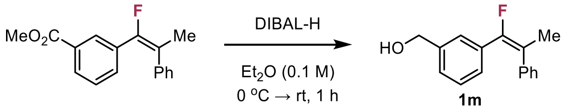

(E)-(3-(1-fluoro-2-phenylprop-1-en-1-yl)phenyl)methanol (1m)

Adapted from literature.[16] The methyl (E)-3-(1-fluoro-2-phenylprop-1-en-1-yl)benzoate (100 mg, 370 µmol) was dissolved in anhydrous Et_2O (3 mL, 0.1 M) and cooled to 0 °C. DIBAL-H (925 µL, 925 µmol, 1.0 M, 2.5 equiv.) was added dropwise, and the reaction mixture was allowed to warm to room temperature and stirred for 1 h. Saturated aq. Na_2SO_4 was carefully added with continued stirring until a solid precipitated, and the supernatant liquid became clear. The solid material was removed by filtration through a sintered funnel and the filtrate was concentrated to afford **1m** as a colorless oil (79 mg, 85% yield (E:Z= 1:0.4)).

^{1}H NMR (500 MHz, $CDCl_3$) (δ, ppm): 7.22 – 7.14 (m, 5H), 7.13 – 7.03 (m, 5H), 6.96 (d, J = 7.7 Hz, 1H), 4.58 (s, 1H), 4.45 (s, 2H), 2.11 (d, J = 3.9 Hz, 3H), 1.98 (s, 1H).
^{13}C{^{1}H} NMR (126 MHz, $CDCl_3$) (δ, ppm): 154.4 (d, J_{C-F} = 244.0 Hz), 141.1, 140.6, 140.2, 140.1, 133.3 (d, J_{C-F} = 29.4 Hz), 130.9, 130.1, 129.0, (d, J_{C-F} = 3.1 Hz), 128.7, 128.6, 128.5, 128.1, 127.7, 127.7, 127.2, 126.8, 126.7 (d, J_{C-F} = 5.6 Hz), 126.6, 124.4, 117.5 (d, J_{C-F} = 22.2 Hz), 86.2, 79.7, 65.2, 65.1, 17.8 (d, J_{C-F} = 7.8 Hz).
^{19}F NMR (471 MHz, $CDCl_3$) (δ, ppm): -105.33 (d, J = 4.0 Hz).
HRMS (ESI TOF): calcd. for $C_{16}H_{15}FO$ $[M]^+$ 242.1107, found, 242.1108

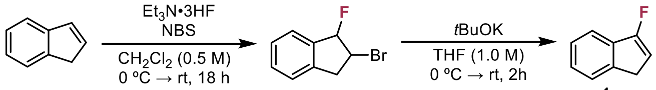

3-fluoro-1H-indene (1n)

Adapted procedure from literature.[6] To a flame-dry flask, N-bromosuccinimide (4.00 g, 22.5 mmol, 1.5 equiv.) and 1H-indene (1.75 mL, 15.0 mmol) were dissolved in anhydrous CH_2Cl_2 (30.0 mL, 0.5 M). Triethylamine trihydrofluoride (3.67 mL, 22.5 mmol, 1.5 equiv.) was carefully added at 0 °C and the reaction mixture was stirred 18 h at room temperature. The reaction was quenched with saturated aq. $NaHCO_3$ at 0 °C and extracted with CH_2Cl_2. The combined organic layers were washed with 1N HCl and then concentrated *in vacuo*. The residual crude was filtered through a silica plug with n-pentane to afford 2-bromo-1-fluoro-2,3-dihydro-1H-indene, which was used for the next step without further purification. In a flame dried flask, 2-bromo-1-fluoro-2,3-dihydro-1H-indene was dissolved in anhydrous THF (15.0 mL, 1.0 M), and the solution was cooled to 0 °C. Potassium tert-butoxide (1.68 g, 15.0 mmol, 1.0 equiv.) was added, the reaction mixture was warmed to room temperature and stirred 2 hr. The reaction was quenched with water, extracted with n-pentane, dried over Na_2SO_4 and carefully concentrated *in vacuo*. The obtained crude was purified by column chromatography (100% hexanes) to afford **1n** as a colorless oil (518 mg, 26% yield).

^{1}H NMR (500 MHz, $CDCl_3$) (δ, ppm): 7.46 – 7.40 (m, 2H), 7.34 (t, J = 7.5 Hz, 1H), 7.31 – 7.26 (m, 1H), 5.78 (t, J = 2.4 Hz, 1H), 3.31 (dd, J = 6.1, 2.4 Hz, 2H).
^{13}C{^{1}H} NMR (151 MHz, $CDCl_3$) (δ, ppm): 160.6 (d, J_{C-F} = 270.4 Hz), 142.1 (d, J_{C-F} = 6.6 Hz), 136.7 (d, J_{C-F} = 26.0 Hz), 126.6, 126.2, 124.4 (d, J_{C-F} = 2.6 Hz), 117.8 (d, J_{C-F} = 2.9 Hz), 105.5 (d, J_{C-F} = 9.5 Hz), 32.9 (d, J_{C-F} = 6.0 Hz).
^{19}F NMR (471 MHz, $CDCl_3$) (δ, ppm): -133.85 – -133.90 (m).
HRMS (ESI TOF): calcd. for C_9H_8F $[M+H]^+$ 135.0610, found 135.0616

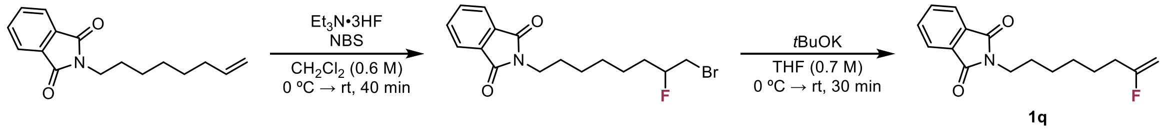

2-(7-fluorooct-7-en-1-yl)isoindoline-1,3-dione (1q)

Adapted procedure from literature.[6] To a flame-dry flask, N-Bromosuccinimide (1.33g, 7.50 mmol, 1.5 equiv.) and 2-(oct-7-en-1-yl)isoindoline-1,3-dione (1.29 g, 5.00 mmol) were dissolved in anhydrous CH_2Cl_2 (8.33 mL, 0.6 M). Triethylamine trihydrofluoride (1.22 mL, 7.50 mmol, 1.5 equiv.) was carefully added at 0 °C and the reaction mixture was stirred 40 min at room temperature. The reaction was quenched with saturated aq. $NaHCO_3$ at 0 °C and extracted with CH_2Cl_2. The combined organic layers were washed with 1N HCl and then concentrated *in vacuo*. The residual crude was filtered through a silica plug with hexane to afford the 2-(8-bromo-7-fluorooctyl)isoindoline-1,3-dione, which was used for the next step without further purification. In a flame dried flask, 2-(8-bromo-7-fluorooctyl)isoindoline-1,3-dione was dissolved in anhydrous THF (7.14 mL, 0.7 M), and the solution was cooled to 0 °C. Potassium tert-butoxide (701 mg, 6.25 mmol, 1.25 equiv.) was added, the reaction mixture was warmed to room temperature and stirred 30 min. The reaction was quenched with water, extracted with hexane, dried over Na_2SO_4 and concentrated *in vacuo*. The obtained crude was purified by column chromatography (100% hexanes) to afford **1q** as a white solid (250 mg, 18% yield).

^{1}H NMR (500 MHz, $CDCl_3$) (δ, ppm): 7.84 (dd, J = 5.4, 3.1 Hz, 2H), 7.71 (dd, J = 5.5, 3.0 Hz, 2H), 4.47 (dd, J = 17.7, 2.7 Hz, 1H), 4.18 (dd, J = 50.5, 2.7 Hz, 1H), 3.68 (t, J = 7.2 Hz, 2H), 2.17 (dt, J = 15.5, 7.5 Hz, 2H), 1.73 – 1.65 (m, 2H), 1.54 – 1.46 (m, 2H), 1.41 – 1.31 (m, 4H).
^{13}C{^{1}H} NMR (126 MHz, $CDCl_3$) (δ, ppm): 168.6 166.9 (d, $J_{C–F}$ = 256.9 Hz), 134.0, 132.3, 123.3, 89.5 (d, $J_{C–F}$ = 20.6 Hz), 38.1, 31.8 (d, $J_{C–F}$ = 27.2 Hz), 28.6, 28.5, 26.7, 25.9 (d, $J_{C–F}$ = 2.2 Hz).
^{19}F{^{1}H} NMR (471 MHz, $CDCl_3$) (δ, ppm): -94.77.
HRMS (ESI TOF): calcd. for $C_{16}H_{19}FNO_2$ $[M+H]^+$ 276.1400, found 276.1402

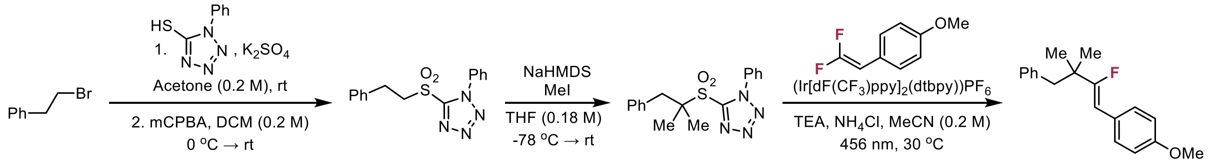

5-(phenethylsulfonyl)-1-phenyl-1*H*-tetrazole

Adapted procedure from literature.[17] In a round-bottomed flask was charged with (2-bromoethyl)benzene (2.50 g, 13.5 mmol), 1-phenyl-1H-tetrazole-5-thiol (2.65 g, 14.9 mmol, 1.1 equiv.), potassium carbonate (3.73g, 27.0 mmol, 2.0 equiv.). Then the reaction was dissolved in acetone (67.5 mL, 0.2 M), and stirring at room temperature overnight. The mixture was filtered through a pad of Celite (eluted with EtOAc) to afford the crude material which was carried forward to the oxidation reaction without further purification. To a solution of 5-(phenethylthio)-1-phenyl-1*H*-tetrazole (3.81 g, 13.5 mmol) in CH_2Cl_2 (67.5 mL, 0.2 M) at 0 °C in an ice/water bath was added 75% mCPBA (6.83 g, 29.7 mmol, 2.2 equiv.) portionwise, and the mixture was allowed to stir to room temperature. After completion the reaction was quenched with saturated aq. $NaHCO_3$ solution and saturated aq. $Na_2S_2O_3$ solution were added slowly, and the mixture was stirred for 10 min. The aqueous layer was extracted with CH_2Cl_2, and the organic layers were dried over Na_2SO_4. The solvent was removed *in vacuo*, and the resulting crude material was purified by recrystallization (CH_2Cl_2/MeOH). Yielding white crystalline solid (2.73 g, 64% yield)

^{1}H NMR (500 MHz, $CDCl_3$) (δ, ppm): 7.71 – 7.67 (m, 2H), 7.66 – 7.58 (m, 3H), 7.37 – 7.30 (m, 2H), 7.30 – 7.22 (m, 3H), 4.00 (td, *J* = 5.5, 4.9, 2.9 Hz, 2H), 3.27 (dt, *J* = 8.0, 5.2, 2.8 Hz, 2H).
^{13}C{^{1}H} NMR (126 MHz, $CDCl_3$) (δ, ppm): 153.5, 136.4, 133.1, 131.67, 129.9, 129.2, 128.7, 127.6, 125.2, 57.4, 28.6.
HRMS (ESI TOF): calcd. for $C_{15}H_{15}N_4O_2S$ $[M+H]^+$ 315.0916, found 315.0915

5-((2-methyl-2-phenylpropyl)sulfonyl)-1-phenyl-1*H*-tetrazole
Adapted procedure from literature.[17] To a flame-dry a two-neck Schlenk flask charged with stir bar followed by the addition of 5-(phenethylsulfonyl)-1-phenyl-1H-tetrazole (2.55 g, 8.11 mmol). The flask was put under vacuum and then back filled with N_2 (3 x). Anhydrous THF (45 mL, 0.18 M) was added to the flask and then subsequently cooled to -78 ºC. Hexamethyldisilazane sodium salt solution (24.3 mL, 1.0 M, 24.3 mmol, 3 equiv.) was added drop wise to the reaction mixture at -78 °C. After stirring for 30 min, MeI (2.04 mL, 32.4 mmol, 4 equiv.) was added, the mixture was stirred at room temperature for 16 h. Reaction was quenched slowly with sat. aq. NH_4Cl. The aqueous layer was extracted with EtOAc (3 x), and the combined organic layer was washed with sat. aq. $NaHCO_3$ and brine. The organic layer was dried over Na_2SO_4, filtered and the solvent was removed *in vacuo*. Residue was purified by column chromatography (0-20% EtOAc:Hexanes). Purification yielded off-white solid (1.88 g, 68% yield)

^{1}H NMR (500 MHz, $CDCl_3$) (δ, ppm): 7.68 – 7.57 (m, 5H), 7.35 - 7.28 (m, 3H), 7.22 – 7.14 (m, 2H), 3.25 (s, 2H), 1.44 (s, 6H).
^{13}C{^{1}H} NMR (126 MHz, $CDCl_3$) (δ, ppm): 152.2, 133.9, 133.6, 131.6, 131.2, 129.5, 128.6, 127.6, 126.3, 68.6, 39.8, 20.0.
HRMS (ESI TOF): calcd. for $C_{17}H_{18}N_4O_2NaS$ $[M+Na]^+$ 365.1048, found 365.1054

(*Z*)-1-(2-fluoro-3,3-dimethyl-4-phenylbut-1-en-1-yl)-4-methoxybenzene (1s)
Adapted procedure from literature.[18] To a 10-mL sealable glass vessel containing a magnetic stirring bar was flame-dried under vacuum and filled with argon after cooling to room temperature. To the glass vessel were added the 5-((2-methyl-2-phenylpropyl)sulfonyl)-1-phenyl-1*H*-tetrazole (1.01 g, 2.92 mmol, 2 equiv.), 1-(2,2-difluorovinyl)-4-methoxybenzene (250 mg, 1.47 mmol), $[Ir\{dF(CF_3)ppy\}_2(dtbpy)]PF_6$ (33.0 mg, 29.4 μmol, 2 mol%), and NH_4Cl (236 mg, 4.41 mmol, 3 equiv.). The mixture was evacuated under vacuum and refilled with N_2 (3 x). Under an N_2 atmosphere, Et_3N (1.23 mL, 8.82 mmol, 6 equiv.) and MeCN (7.35 mL, 0.2 M) were added, and the reaction was sealed and stirred under irradiation for 18 h at 30 °C. Upon completion, the mixture was diluted with EtOAc (~3 mL) and ~2-3 drops of H_2O. The mixture was stirred at room temperature for 5-10 min. The mixture was partitioned between water and EtOAc. The aqueous layer was extracted with EtOAc (2 x). The combined organic layer was washed with brine, dried over Na_2SO_4, filtered, and the organic solvent was evaporated under reduced pressure. The crude residue was then purified via flash column chromatography (50:1 hexanes/EtOAc) to afford **1s** as an orange oil (295.2 mg, 71% yield).

^{1}H NMR (500 MHz, $CDCl_3$) (δ, ppm): 7.39 (d, *J* = 8.8 Hz, 1H), 7.23 (dd, *J* = 8.0, 6.4 Hz, 1H), 7.20 – 7.15 (m, 1H), 7.14 – 7.10 (m, 1H), 6.85 (d, *J* = 8.8 Hz, 1H), 5.25 (d, *J* = 41.4 Hz, 1H), 3.81 (s, 2H), 2.81 (s, 1H), 1.54 (s, 1H), 1.17 (s, 4H).

$^{13}C\{^{1}H\}$ NMR (126 MHz, $CDCl_3$) (δ, ppm): 165.5, 163.4, 158.4 (d, $J_{C–F}$ = 2.8 Hz), 138.3, 130.5, 129.8 (d, $J_{C–F}$ = 7.6 Hz), 127.9, 126.8, 126.3, 113.9, 104.3 (d, $J_{C–F}$ = 10.1 Hz), 55.4, 45.9, 40.0 (d, $J_{C–F}$ = 23.3 Hz), 25.5 (d, $J_{C–F}$ = 3.3 Hz).
^{19}F NMR (471 MHz, $CDCl_3$) (δ, ppm): -111.93 (d, J = 41.3 Hz).
HRMS (ESI TOF): calcd. for $C_{19}H_{22}OF$ $[M+H]^+$ 285.1655, found 285.1648

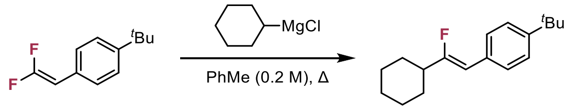

1t

(*Z*)-1-(*tert*-butyl)-4-(2-cyclohexyl-2-fluorovinyl)benzene (1t)
To a solution of 1-(*tert*-butyl)-4-(2,2-difluorovinyl)benzene (392 mg, 2.0 mmol, 1 equiv.) in toluene (10 mL) was added dropwise a solution of cyclohexylmagnesium chloride solution (2 M in THF) (4.00 mL, 8.0 mmol, 4 equiv.) under N_2 atmosphere. The mixture was stirred for 24 h at 110 °C in a heating block. Reaction was monitored by GC-MS, after completion, the reaction mixture was quenched with saturated aq. NH_4Cl (10 mL) and extracted with ethyl acetate (3 × 20 mL). The combined organic layer was washed with water and brine, then dried over anhydrous Na_2SO_4, filtered, and concentrated under vacuum. The crude residue was then purified by column chromatography on silica gel using n-hexane as eluent to afford **1t** as a light yellow oil (367 mg, 70% yield)

^{1}H NMR (500 MHz, $CDCl_3$) (δ, ppm): 7.43 – 7.39 (m, 2H), 7.36 – 7.31 (m, 2H), 5.41 (d, J = 41.0 Hz, 1H), 2.22 (dtt, J = 14.7, 11.0, 3.5 Hz, 1H), 1.98 – 1.91 (m, 2H), 1.81 (dd, J = 8.8, 3.5 Hz, 2H), 1.71 (dtt, J = 12.7, 3.2, 1.7 Hz, 1H), 1.31 (s, 14H).
$^{13}C\{^{1}H\}$ NMR (151 MHz, $CDCl_3$) (δ, ppm): 165.8, 164.0, 149.6, 131.4, 128.2 (d, $J_{C–F}$ = 7.3 Hz), 125.4, 103.4 (d, $J_{C–F}$ = 9.3 Hz), 41.7 (d, $J_{C–F}$ = 24.9 Hz), 34.6, 31.4 (d, $J_{C–F}$ = 6.0 Hz), 30.3 (d, $J_{C–F}$ = 2.1 Hz), 26.1.
^{19}F NMR (471 MHz, $CDCl_3$) (δ, ppm): -106.19 (dd, J = 40.9, 15.7 Hz).
HRMS (ESI TOF): calcd. for $C_{18}H_{25}F$ $[M]^+$ 260.1940, found 260.1946

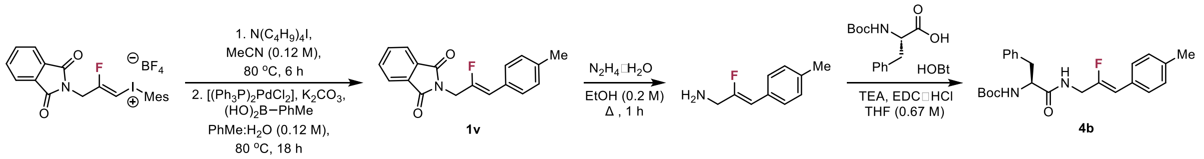

(*Z*)-2-(2-fluoro-3-(*p*-tolyl)allyl)isoindoline-1,3-dione (1v)
Prepared according to general procedure A. (*Z*)-(3-(1,3-dioxoisoindolin-2-yl)-2-fluoroprop-1-en-1-yl)(mesityl)iodonium tetrafluoroborate (426 mg, 0.683 mmol), tetrabutylammonium iodide (277 mg, 0.751 mmol, 1.1 equiv.), and MeCN (6.00 mL, 0.12 M). The suspension was stirred at 80 ºC using a heating block for 6 hours and the solvent was evaporated under reduced pressure. $[(Ph_3P)_2PdCl_2]$ (36 mg, 0.051 mmol, 0.075 equiv.), p-tolylboronic acid (232 mg, 1.71 mmol, 2.5 equiv.), and K_2CO_3 (236 mg, 1.71 mmol, 2.5 equiv.). **1v** was isolated via flash column chromatography (0-20% EtOAc:Hexanes) as an off-white solid (170 mg, 84% yield)

^{1}H NMR (500 MHz, $CDCl_3$) (δ, ppm): 7.90 (dd, J = 5.5, 3.1 Hz, 2H), 7.75 (dd, J = 5.5, 3.1 Hz, 2H), 7.57 (dd, J = 6.7, 3.0 Hz, 1H), 7.18 – 7.12 (m, 3H), 6.01 (d, J = 36.8 Hz, 1H), 4.56 (d, J = 15.5 Hz, 2H), 2.32 (s, 3H).
$^{13}C\{^{1}H\}$ NMR (126 MHz, $CDCl_3$) (δ, ppm): 167.5, 134.2, 132.0, 130.0, 129.2, 127.7, 125.9, 123.6, 107.2, 39.3 (d, $J_{C–F}$ = 32.5 Hz), 20.1.

^{19}**F NMR** (471 MHz, $CDCl_3$) (δ, ppm): -112.31 (dt, J = 36.9, 15.5 Hz).
HRMS (ESI TOF): calcd. for $C_{18}H_{14}FNO_2$ $[M+H]^+$ 296.1087, found 296.1092

(*Z*)-2-fluoro-3-(*p*-tolyl)prop-2-en-1-amine

(*Z*)-2-(2-fluoro-3-(*p*-tolyl)allyl)isoindoline-1,3-dione (93.5 mg, 0.245 mmol) was dissolved in ethanol (1.22 mL, 0.2 M) followed by the addition of hydrazine hydrate (26.4 µL, 0.538 mmol 2.2 equiv). The mixture was heated to reflux using a heating block and allowed to stir for one hour. The reaction was quenched with aq. HCl (0.5 mL, 6.0 M) and stirred for 30 min. The reaction precipitates were filtered off while the filtrate was collected and dried over Na_2SO_4. The filtrate solvent was removed *in vacuo*. The resulting salt was then dissolved in aq. NaOH (2 mL, 6.0 M) and extracted into CH_2Cl_2 (3 x 10 mL). The combined organic layers were washed with brine (5 mL) then dried over Na_2SO_4. The resulting off-white solid (40.5 mg, 99% yield) was used without further purification.

^{1}**H NMR** (500 MHz, $CDCl_3$) (δ, ppm): 7.38 (d, J = 8.0 Hz, 2H), 7.14 (d, J = 7.8 Hz, 2H), 5.62 (d, J = 39.4 Hz, 1H), 3.48 (d, J = 14.5 Hz, 2H), 2.34 (s, 3H).
^{13}**C{^{1}H} NMR** (126 MHz, $CDCl_3$) (δ, ppm): 137.0, 130.5, 129.3, 128.6, 128.5, 105.5, 105.5, 44.1 (d, $J_{C–F}$ = 30.8 Hz), 21.4.
^{19}**F NMR** (471 MHz, $CDCl_3$) (δ, ppm): -111.22 (dt, J = 39.6, 14.5 Hz).
HRMS (ESI TOF): calcd. for $C_{10}H_{13}FN$ $[M+H]^+$ 166.1032, found 166.1036

***tert*-butyl (*S*,*Z*)-(1-((2-fluoro-3-(*p*-tolyl)allyl)amino)-1-oxo-3-phenylpropan-2-yl)carbamate (4b)**

A two dram vial was charged with (*Z*)-2-fluoro-3-(*p*-tolyl)prop-2-en-1-amine (40.5 mg, 0.245 mmol), (Boc)-L-phenylalanine (68.3 mg, 0.257 mmol, 1.05 equiv.), hydroxybenzotriazole (45.0 mg, 0.294 mmol, 1.2 equiv.) and anhydrous THF (366 µL, 0.67 M). Triethylamine (68.3 µL, 0.490 mmol, 2.0 equiv.) was added to the stirring reaction mixture which was subsequently cooled to 0 ºC. 1-ethyl-3-(3-dimethylaminopropyl)carbodiimide•HCl (93.9 mg, 0.490 mmol, 2 equiv.) was added slowly to the mixture. The reaction was allowed to warm to room temperature and left stirring for 16 h. After completion, the reaction was quenched with sat. aq. $NaHCO_3$ (1 mL). The product was extracted from the aqueous solution with CH_2Cl_2 (3 x 3 mL). The combined organic layers were washed with brine (2 mL), dried over Na_2SO_4 and concentrated *in vacuo*. The crude residue was purified via flash column chromatography (0-10% MeOH:CH_2Cl_2) to afford **4b** as a white solid (70.2 mg, 70% yield)

^{1}**H NMR** (500 MHz, $CDCl_3$) (δ, ppm): 7.54 (d, J = 6.8 Hz, 1H), 7.30 – 7.10 (m, 10H), 6.17 (t, J = 6.1 Hz, 1H), 5.77 (d, J = 37.5 Hz, 1H), 4.98 (s, 1H), 4.38 – 4.29 (m, 1H), 4.05 (dt, J = 15.8, 5.4 Hz, 2H), 3.07 (dd, J = 7.1, 3.5 Hz, 2H), 2.26 (s, 3H), 1.38 (s, 10H).
^{13}**C{^{1}H} NMR** (126 MHz, $CDCl_3$) (δ, ppm): 171.3, 135.8, 130.1, 129.3, 129.2 (d, $J_{C–F}$ = 9.6 Hz), 128.8, 127.5, 127.1, 125.9, 41.0 (d, $J_{C–F}$ = 31.2 Hz), 38.4, 28.3, 20.1.
^{19}**F NMR** (471 MHz, $CDCl_3$) (δ, ppm): -111.67 (dt, J = 37.5, 15.6 Hz).
HRMS (ESI TOF): calcd. for $C_{24}H_{29}FN_2O_3Na$ $[M+Na]^+$ 435.2060, found 435.2059

Characterization of oxidative cleavage products

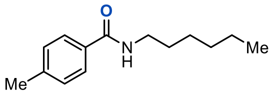

***N*-hexyl-4-methylbenzamide (2a)**

Prepared according to general procedure B (405 nm, 6 hours). 4-nitrophthalonitrile (51.9 mg, 0.300 mmol, 1.5 equiv.), 1-(1-fluorovinyl)-4-methylbenzene (27.3 mg, 0.200 mmol) in acetonitrile (2.0 mL, 0.1 M). Two reactions run simultaneously were combined for isolation. NMR yield = 70.5%. The title compound was isolated via flash column chromatography (gradient 0 – 40% EtOAc/hexanes) as a white solid (44 mg, 50% yield). All analytical data for **2a** was in accordance with literature data.[19]

^{1}H NMR (500 MHz, $CDCl_3$) (δ, ppm): 7.65 (d, *J* = 7.9 Hz, 2H), 7.20 (d, *J* = 7.9 Hz, 2H), 6.21 (t, *J* = 6.0 Hz, 1H), 3.42 (td, *J* = 7.3, 5.7 Hz, 2H), 2.38 (s, 3H), 1.59 (p, *J* = 7.3 Hz, 2H), 1.41 – 1.25 (m, 6H), 0.88 (t, *J* = 6.8 Hz, 3H).
^{13}C{^{1}H} NMR (126 MHz, $CDCl_3$) (δ, ppm): 167.7, 141.8, 132.1, 129.3, 127.0, 40.2, 31.6, 29.8, 26.8, 22.7, 21.5, 14.1.

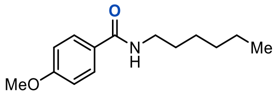

***N*-hexyl-4-methoxybenzamide (2b)**

Prepared according to general procedure B (405 nm, 7 hours). 4-nitrophthalonitrile (51.9 mg, 0.300 mmol, 1.5 equiv.), 1-(1-fluorovinyl)-4-methoxybenzene (30.4 mg, 0.200 mmol) in acetonitrile (2.0 mL, 0.1 M). NMR yield = 46%. The title compound was isolated via flash column chromatography (gradient 5 – 40%EtOAc/hexanes) as a pale yellow solid (18.9 mg, 40% yield). All analytical data for **2b** was in accordance with literature data.[19]

^{1}H NMR (500 MHz, $CDCl_3$) (δ, ppm): (500 MHz, $CDCl_3$) (δ, ppm): 7.72 (d, *J* = 8.8 Hz, 2H), 6.92 (d, *J* = 8.8 Hz, 2H), 5.99 (s, 1H), 3.85 (s, 3H), 3.43 (td, *J* = 7.3, 5.8 Hz, 2H), 1.60 (p, *J* = 7.3 Hz, 2H), 1.46 – 1.22 (m, 8H), 0.97 – 0.85 (m, 3H).
^{13}C{^{1}H} NMR (126 MHz, $CDCl_3$) (δ, ppm): 167.1, 162.2, 128.7, 127.3, 113.9, 55.6, 40.2, 31.7, 29.9, 26.8, 22.7, 14.2.

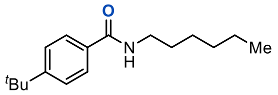

4-(*tert*-butyl)-N-hexylbenzamide (2c)

Prepared according to general procedure B (405 nm, 6 hours). 4-nitrophthalonitrile (51.9 mg, 0.300 mmol, 1.5 equiv.), 1-(*tert*-butyl)-4-(1-fluorovinyl)benzene (35.7 mg, 0.200 mmol) in acetonitrile (2.0 mL, 0.1 M). Two reactions run simultaneously were combined for isolation. NMR yield = 69%. The title compound was isolated via flash column chromatography (gradient 0 – 4% EtOAc/hexanes) as a pale yellow solid (38 mg, 53% yield).

^{1}H NMR (500 MHz, $CDCl_3$) (δ, ppm): 7.69 (d, *J* = 8.3 Hz, 2H), 7.43 (d, *J* = 8.2 Hz, 2H), 6.12 (t, *J* = 5.9 Hz, 1H), 3.44 (td, *J* = 7.1, 5.7 Hz, 2H), 1.60 (p, *J* = 7.2 Hz, 2H), 1.37 (dt, *J* = 8.6, 3.7 Hz, 2H), 1.33 (s, 13H), 0.93 – 0.85 (m, 3H).
^{13}C{^{1}H} NMR (126 MHz, $CDCl_3$) (δ, ppm): 167.6, 154.9, 132.1, 126.8, 125.6, 40.2, 35.0, 31.7, 31.3, 29.8, 26.8, 22.7, 14.2.
HRMS (ESI TOF): calcd. for $C_{17}H_{27}NONa$ $[M+Na]^+$ 284.1990, found 284.2000

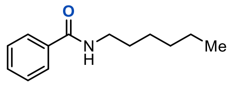

***N*-hexylbenzamide (2d)**
Prepared according to general procedure B (405 nm, 8 hours). 4-nitrophthalonitrile (85.4 mg, 0.494 mmol, 1.5 equiv.), (1-fluorovinyl)benzene (40.2 mg, 0.329 mmol) in acetonitrile (3.3 mL, 0.1 M). Two reactions run simultaneously were combined for isolation. NMR yield = 70%. The title compound was isolated via flash column chromatography (gradient 0 – 40% EtOAc/hexanes) as a clear oil (70.8 mg, 52% yield). All analytical data for **2d** was in accordance with literature data.[19]

^{1}H NMR (500 MHz, $CDCl_3$) (δ, ppm): 7.76 (d, *J* = 7.3 Hz, 2H), 7.44 (t, *J* = 7.4 Hz, 1H), 7.36 (t, *J* = 7.6 Hz, 2H), 6.58 (t, *J* = 5.9 Hz, 1H), 3.39 (td, *J* = 7.4, 5.7 Hz, 2H), 1.56 (p, *J* = 6.8 Hz, 2H), 1.37 – 1.22 (m, 6H), 0.86 (t, *J* = 6.8 Hz, 3H).
^{13}C{^{1}H} NMR (126 MHz, $CDCl_3$) (δ, ppm): 167.7, 135.0, 131.5, 128.7, 126.9, 40.3, 31.7, 29.8, 26.8, 22.7, 14.2.

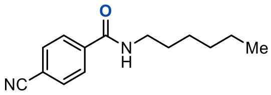

4-cyano-*N*-hexylbenzamide (2e)
Prepared according to general procedure B (405 nm, 8 hours). 4-nitrophthalonitrile (51.9 mg, 0.300 mmol, 1.5 equiv.), 4-(1-fluorovinyl)benzonitrile (29.4 mg, 0.200 mmol) in acetonitrile (2.0 mL, 0.1 M). Two reactions run simultaneously were combined for isolation. The title compound was isolated via flash column chromatography (gradient 10-15% EtOAc/hexanes) as a light orange solid (47.7 mg, 51% yield). All analytical data for **2e** was in accordance with literature data.[19]

^{1}H NMR (500 MHz, $CDCl_3$) (δ, ppm): 7.85 (d, *J* = 7.9 Hz, 2H), 7.74 (d, *J* = 8.0 Hz, 2H), 6.10 (s, 1H), 3.47 (q, *J* = 6.8 Hz, 2H), 1.62 (p, *J* = 7.3 Hz, 2H), 1.43 – 1.29 (m, 6H), 0.90 (d, *J* = 6.5 Hz, 3H).
^{13}C{^{1}H} NMR (126 MHz, $CDCl_3$) (δ, ppm): 165.8, 138.9, 132.6, 127.7, 118.2, 115.2, 40.5, 31.6, 29.7, 26.8, 22.7, 14.2.

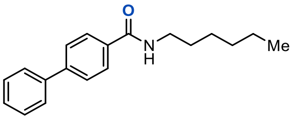

N-hexyl-[1,1'-biphenyl]-4-carboxamide (2f)
Prepared according to general procedure B (405 nm, 8 hours). 4-nitrophthalonitrile (51.9 mg, 0.300 mmol, 1.5 equiv.), 4-(1-fluorovinyl)-1,1'-biphenyl (39.6 mg, 0.200 mmol) in acetonitrile (2.0 mL, 0.1 M). Two reactions run simultaneously were combined for isolation. NMR yield = 54%. The title compound was isolated via flash column chromatography (gradient 0-3% MeOH/DCM) as a white solid (53.2 mg, 47% yield).

^{1}H NMR (500 MHz, $CDCl_3$) (δ, ppm): 7.83 (d, *J* = 8.0 Hz, 2H), 7.65 (d, *J* = 8.0 Hz, 2H), 7.61 (d, *J* = 7.7 Hz, 2H), 7.46 (t, *J* = 7.5 Hz, 2H), 7.39 (t, *J* = 7.4 Hz, 1H), 6.14 (t, *J* = 5.8 Hz, 1H), 3.48 (q, *J* = 6.8 Hz, 2H), 1.64 (p, *J* = 7.3 Hz, 2H), 1.46 – 1.30 (m, 6H), 0.95 – 0.86 (m, 3H).
^{13}C{^{1}H} NMR (101 MHz, $CDCl_3$) (δ, ppm): 167.3, 144.3, 140.2, 133.7, 129.1, 128.1, 127.5, 127.4, 127.5, 40.3, 31.7, 29.8, 26.8, 22.7, 14.2.
HRMS (ESI TOF): calcd. for $C_{19}H_{24}NO$ $[M+H]^+$ 282.1858, found 282.1862

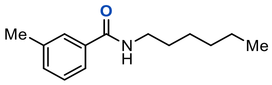

N-hexyl-3-methylbenzamide (2g)

Prepared according to general procedure B (405 nm, 6 hours). 4-nitrophthalonitrile (51.9 mg, 0.300 mmol, 1.5 equiv.), 1-(1-fluorovinyl)-3-methylbenzene (27.3 mg, 0.200 mmol) in acetonitrile (2.0 mL, 0.1 M). NMR yield = 75%. The title compound was isolated via flash column chromatography (gradient 0 – 40% EtOAc/hexanes) as a white solid (31 mg, 71% yield). All analytical data for **2g** was in accordance with literature data.[20]

^{1}H NMR (500 MHz, $CDCl_3$) (δ, ppm): 7.58 (d, J = 2.0 Hz, 1H), 7.52 (dt, J = 5.9, 2.6 Hz, 1H), 7.29 (dd, J = 4.7, 1.9 Hz, 2H), 6.16 (s, 1H), 3.43 (td, J = 7.3, 5.7 Hz, 2H), 1.60 (p, J = 7.2 Hz, 2H), 1.42 – 1.28 (m, 7H), 0.94 – 0.84 (m, 3H).
^{13}C{^{1}H} NMR (126 MHz, $CDCl_3$) (δ, ppm): 167.9, 138.5, 135.0, 132.2, 128.5, 127.7, 123.9, 40.2, 31.6, 29.8, 26.8, 22.7, 21.5, 14.2.

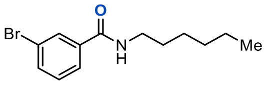

3-bromo-N-hexylbenzamide (2h)

Prepared according to general procedure B (405 nm, 12 hours). 4-nitrophthalonitrile (51.9 mg, 0.300 mmol, 1.5 equiv.), 1-bromo-3-(1-fluorovinyl)benzene (40.2 mg, 0.200 mmol) in acetonitrile (2.0 mL, 0.1 M). Two reactions run simultaneously were combined for isolation. NMR yield = 71.5%. The title compound was isolated via flash column chromatography (gradient 0 – 35% EtOAc/hexanes) as a clear oil (63.5 mg, 56% yield). All analytical data for **2h** was in accordance with literature data.[21]

^{1}H NMR (500 MHz, $CDCl_3$) (δ, ppm): 7.91 (d, J = 2.0 Hz, 1H), 7.69 (d, J = 7.8 Hz, 1H), 7.59 (dd, J = 8.0, 2.0 Hz, 1H), 7.27 (t, J = 7.8 Hz, 1H), 6.57 (s, 1H), 3.46 – 3.36 (m, 2H), 1.60 (p, J = 7.3 Hz, 2H), 1.40 – 1.23 (m, 6H), 0.89 (t, J = 6.7 Hz, 3H).
^{13}C{^{1}H} NMR (126 MHz, $CDCl_3$) (δ, ppm): 166.3, 137.0, 134.3, 130.3, 130.1, 125.6, 122.8, 40.4, 31.6, 29.6, 26.8, 22.6, 14.1.

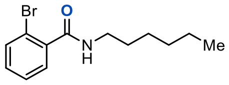

2-bromo-N-hexylbenzamide (2i)

Prepared according to general procedure B (405 nm, 24 hours). 4-nitrophthalonitrile (51.9 mg, 0.300 mmol, 1.5 equiv.), 1-bromo-2-(1-fluorovinyl)benzene (40.2 mg, 0.200 mmol) in acetonitrile (2.0 mL, 0.1 M). Two reactions run simultaneously were combined for isolation. NMR yield = 57%. The title compound was isolated via flash column chromatography (gradient 0 – 4% EtOAc/hexanes) as a pale yellow solid (55.0 mg, 48% yield). All analytical data for **2i** was in accordance with literature data.[22]

^{1}H NMR (500 MHz, $CDCl_3$) (δ, ppm): 7.57 (d, J = 8.0 Hz, 1H), 7.52 (dd, J = 7.6, 1.8 Hz, 1H), 7.34 (t, J = 7.6 Hz, 1H), 7.25 (td, J = 7.7, 1.7 Hz, 1H), 5.97 (s, 1H), 3.45 (q, J = 6.8 Hz, 2H), 1.62 (p, J = 7.3 Hz, 2H), 1.44 – 1.37 (m, 2H), 1.34 – 1.30 (m, 4H), 0.92 – 0.88 (m, 3H).
^{13}C{^{1}H} NMR (126 MHz, $CDCl_3$) (δ, ppm): 167.4, 138.0, 133.2, 131.0, 129.5, 127.4, 119.1, 40.0, 31.4, 29.3, 26.6, 22.5, 13.9.

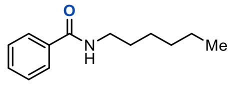

***N*-hexylbenzamide (2j)**
Prepared according to general procedure B (405 nm, 6 hours). 4-nitrophthalonitrile (51.9 mg, 0.300 mmol, 1.5 equiv.), (*Z*)-(1-fluoroprop-1-en-1-yl)benzene (27.2 mg, 0.200 mmol) in acetonitrile (2.0 mL, 0.1 M). Two reactions run simultaneously were combined for isolation. NMR yield = 69%. The title compound was isolated via flash column chromatography (gradient 10 – 15% EtOAc/hexanes) as a white solid (46.9 mg, 57% yield). All analytical data for **2j** was in accordance with literature data.[19]

^{1}H NMR (500 MHz, $CDCl_3$) (δ, ppm): 7.79 – 7.73 (m, 2H), 7.53 – 7.46 (m, 1H), 7.43 (dd, *J* = 8.2, 6.7 Hz, 2H), 6.07 (s, 1H), 3.46 (td, *J* = 7.2, 5.7 Hz, 2H), 1.62 (p, *J* = 7.3 Hz, 2H), 1.42 – 1.30 (m, 6H), 0.94 – 0.87 (m, 3H).
^{13}C{^{1}H} NMR (126 MHz, $CDCl_3$) (δ, ppm): 167.6, 135.1, 131.5, 128.7, 127.0, 40.3, 31.7, 29.8, 26.8, 22.7, 14.2.

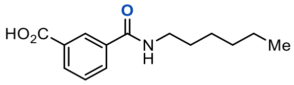

3-(hexylcarbamoyl)benzoic acid (2k)
Prepared according to general procedure B (405 nm, 18 hours). 4-nitrophthalonitrile (63.8 mg, 0.368 mmol, 1.5 equiv.), (Z)-3-(1-fluoro-2-phenylvinyl)benzoic acid (59.5 mg, 0.246 mmol) in acetonitrile (2.46 mL, 0.1 M). Two reactions run simultaneously were combined for isolation. The title compound was isolated via flash column chromatography (gradient 0 – 10% EtOAc/hexanes) as a white solid (36.0 mg, 58% yield). All analytical data for **2k** was in accordance with literature data.[23]

^{1}H NMR (500 MHz, CD_3OD) (δ, ppm): 8.64 (d, *J* = 16.5 Hz, 1H), 8.47 (s, 1H), 8.17 (d, *J* = 7.8 Hz, 1H), 8.02 (d, *J* = 7.8 Hz, 1H), 7.57 (t, *J* = 7.8 Hz, 1H), 3.39 (q, *J* = 6.8 Hz, 2H), 1.63 (p, *J* = 7.2 Hz, 2H), 1.44 – 1.33 (m, 6H), 0.96 – 0.88 (m, 3H).
^{13}C{^{1}H} NMR (126 MHz, CD_3OD) (δ, ppm): 169.4, 169.0, 136.4, 134.8, 133.4, 132.5, 129.8, 129.5, 41.3, 32.7, 30.4, 27.8, 23.7, 14.4.

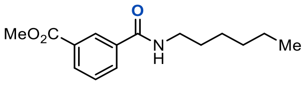

methyl 3-(hexylcarbamoyl)benzoate (2l)
Prepared according to general procedure B (405 nm, 18 hours). 4-nitrophthalonitrile (51.9 mg, 0.300 mmol, 1.5 equiv.), methyl (E)-3-(1-fluoro-2-phenylprop-1-en-1-yl)benzoate (54.1 mg, 0.200 mmol) in acetonitrile (2.0 mL, 0.1 M). Two reactions run simultaneously were combined for isolation. The title compound was isolated via flash column chromatography (gradient 0 – 5% EtOAc/hexanes) as a white solid (24 mg, 45% yield). All analytical data for **2l** was in accordance with literature data.[23]

^{1}H NMR (500 MHz, $CDCl_3$) (δ, ppm): 8.35 (t, *J* = 1.8 Hz, 1H), 8.15 (dd, *J* = 7.7, 1.6 Hz, 1H), 8.04 (dt, *J* = 7.9, 1.6 Hz, 1H), 7.53 (t, *J* = 7.7 Hz, 1H), 6.21 (s, 1H), 3.94 (s, 3H), 3.51 – 3.41 (m, 2H), 1.62 (d, *J* = 8.7 Hz, 2H), 1.40 (q, *J* = 7.5 Hz, 2H), 1.32 (h, *J* = 3.8 Hz, 4H), 0.95 – 0.85 (m, 3H).
^{13}C{^{1}H} NMR (126 MHz, $CDCl_3$) (δ, ppm): 166.6, 135.3, 132.4, 132.0, 130.6, 129.1, 127.5, 52.5, 40.4, 31.7, 29.8, 26.8, 22.7, 14.2.

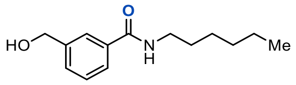

N-hexyl-3-(hydroxymethyl)benzamide (2m)
Prepared according to general procedure B (405 nm, 18 hours). 4-nitrophthalonitrile (51.9 mg, 0.300 mmol, 1.5 equiv.), methyl (E)-3-(1-fluoro-2-phenylprop-1-en-1-yl)benzoate (54.1 mg, 0.200 mmol) in acetonitrile (2.0 mL, 0.1 M). ^{1}H NMR yield determined using CH_2Br_2 as an external standard NMR yield = 43%. Two reactions run simultaneously were combined for isolation. The title compound was isolated via flash column chromatography (gradient 0 – 5% EtOAc/hexanes) as a colorless oil (19 mg, 40% yield).

^{1}H NMR (600 MHz, $CDCl_3$) (δ, ppm): 7.74 (td, *J* = 1.7, 0.8 Hz, 1H), 7.66 (dt, *J* = 7.7, 1.5 Hz, 1H), 7.48 (ddd, *J* = 7.6, 1.8, 1.1 Hz, 1H), 7.41 (t, *J* = 7.6 Hz, 1H), 6.15 (s, 1H), 4.74 (s, 2H), 3.44 (td, *J* = 7.3, 5.7 Hz, 2H), 1.65 – 1.57 (m, 2H), 1.42 – 1.30 (m, 6H), 0.92 – 0.87 (m, 3H).
^{13}C{^{1}H} NMR (151 MHz, $CDCl_3$) (δ, ppm): 167.6, 141.6, 135.3, 129.9, 128.9, 126.2, 125.3, 65.0, 40.3, 31.6, 29.8, 26.8, 22.7, 14.2.
HRMS (ESI TOF): calcd. for $C_{14}H_{22}NO_2$ $[M+H]^+$ 236.1651, found 236.1663

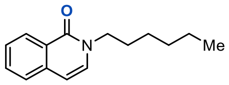

2-hexylisoquinolin-1(2H)-one (2n)
Prepared according to general procedure B (405 nm, 18 hours). 4-nitrophthalonitrile (51.9 mg, 0.300 mmol, 1.5 equiv.), 3-fluoro-1H-indene (26.8 mg, 0.200 mmol) in acetonitrile (2.0 mL, 0.1 M). Upon complete consumption of the alkenyl fluoride as determined by GCMS analysis, the reaction was concentrated under reduced pressure. The mixture was then diluted with 2 mL of diethyl ether and stirred for 5 minutes. The mixture was filtered through celite to remove solids and the mixture was concentrated under reduced pressure. The mixture was then diluted with 2 mL of acetonitrile. Hexylamine (39.6 µL, 0.300 mmol, 1.5 equiv.) was added to the mixture and stirred at 60 °C in a heating block for two hours. ^{1}H NMR yield determined using CH_2Br_2 as an external standard NMR yield = 34%. Two reactions run simultaneously were combined for isolation. The title compound was isolated via flash column chromatography (gradient 10 – 20% EtOAc/hexanes) as a white solid (29.0 mg, 32% yield). Analytical data for **2n** was in accordance to literature data.[24]

^{1}H NMR (500 MHz, $CDCl_3$) (δ, ppm): 8.44 (d, *J* = 8.1 Hz, 1H), 7.65 – 7.59 (m, 1H), 7.53 – 7.44 (m, 2H), 7.06 (d, *J* = 7.3 Hz, 1H), 6.48 (d, *J* = 7.3 Hz, 1H), 3.99 (t, *J* = 7.5 Hz, 2H), 1.78 (p, *J* = 7.4 Hz, 2H), 1.43 – 1.25 (m, 6H), 0.91 – 0.85 (m, 3H).
^{13}C{^{1}H} NMR (126 MHz, $CDCl_3$) (δ, ppm): 162.2, 137.2, 132.1, 131.9, 128.0, 126.9, 125.9, 106.0, 49.6, 31.6, 29.4, 26.6, 22.7, 14.2.
HRMS (ESI TOF): calcd. for $C_{15}H_{20}NO$ $[M+H]^+$ 230.1545, found 230.1547

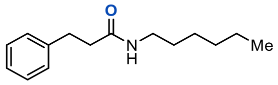

N-hexyl-3-phenylpropanamide (2o)
Prepared according to general procedure B (390 nm, 72 hours). 4-nitrophthalonitrile (69.3 mg, 0.400 mmol, 2.0 equiv.), (3-fluorobut-3-en-1-yl)benzene (30.0 mg, 0.200 mmol) in ethyl acetate (1.0 mL, 0.2 M). Two reactions run simultaneously were combined for isolation. NMR yield = 47%. The title compound was isolated via flash column chromatography (gradient 0 – 40% EtOAc/hexanes) as an yellow oil (31.9 mg, 34% yield). All analytical data for **2o** was in accordance with literature data.[25]

^{1}H NMR (500 MHz, $CDCl_3$) (δ, ppm): 7.28 (t, J = 7.7 Hz, 2H), 7.22 – 7.16 (m, 3H), 5.28 (s, 1H), 3.19 (q, J = 6.7 Hz, 2H), 2.96 (t, J = 7.7 Hz, 2H), 2.46 (t, J = 7.7 Hz, 2H), 1.40 (p, J = 7.1 Hz, 2H), 1.31 – 1.21 (m, 6H), 0.88 (t, J = 6.8 Hz, 3H).
^{13}C{^{1}H} NMR (126 MHz, $CDCl_3$) (δ, ppm): 172.1, 141.1, 128.7, 128.5, 126.4, 39.7, 38.9, 32.0, 31.6, 29.7, 26.7, 22.7, 14.2.

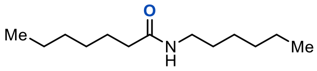

N-hexylheptanamide (2p)
Prepared according to general procedure B (390 nm, 48 hours). 4-nitrophthalonitrile (69.3 mg, 0.400 mmol, 2.0 equiv.), 2-fluorooct-1-ene (26.0 mg, 0.200 mmol) in ethyl acetate (1.0 mL, 0.2 M). Two reactions run simultaneously were combined for isolation. NMR yield = 70.0%. The title compound was isolated via flash column chromatography (gradient 0 – 25% EtOAc/hexanes) as a pale yellow solid (57.8 mg, 67% yield). All analytical data for **2p** was in accordance with literature data.[26]

^{1}H NMR (500 MHz, $CDCl_3$) (δ, ppm): 5.38 (s, 1H), 3.24 (q, J = 6.8 Hz, 2H), 2.15 (t, J = 7.7 Hz, 2H), 1.61 (h, J = 8.2, 7.8 Hz, 3H), 1.48 (q, J = 7.2 Hz, 2H), 1.38 – 1.22 (m, 11H), 0.88 (dt, J = 7.0, 3.2 Hz, 6H).
^{13}C{^{1}H} NMR (126 MHz, $CDCl_3$) (δ, ppm): 173.2, 39.6, 37.1, 31.7, 31.6, 29.8, 29.1, 26.7, 26.0, 22.7, 22.7, 14.2, 14.2.

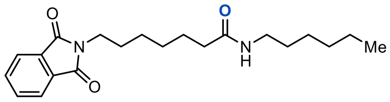

7-(1,3-dioxoisoindolin-2-yl)-N-hexylheptanamide (2q)
Prepared according to general procedure B (390, 48 hours). 4-nitrophthalonitrile (69.3 mg, 0.400 mmol, 2.0 equiv.), 2-(7-fluorooct-7-en-1-yl)isoindoline-1,3-dione (55.1 mg, 0.200 mmol) in ethyl acetate (1.0 mL, 0.2 M). Two reactions run simultaneously were combined for isolation. NMR yield = 67%. The title compound was isolated via flash column chromatography (gradient 20 - 100% EtOAc/hexanes) as a pale yellow solid (76.8 mg, 54% yield).

^{1}H NMR (500 MHz, $CDCl_3$) (δ, ppm): 7.87 – 7.80 (m, 2H), 7.74 – 7.67 (m, 2H), 5.43 (s, 1H), 3.67 (t, J = 7.2 Hz, 2H), 3.27 – 3.19 (m, 2H), 2.14 (t, J = 7.6 Hz, 2H), 1.69-1.59 (m, 4H), 1.49-1.46 (m, 2H),1.37 – 1.22 (m, 9H), 0.92 – 0.84 (m, 3H).
^{13}C{^{1}H} NMR (126 MHz, $CDCl_3$) (δ, ppm): 172.9, 168.6, 134.0, 132.3, 123.3, 39.7, 38.0, 36.9, 31.6, 29.8, 28.9, 28.6, 26.7, 26.6, 25.8, 22.7, 14.2.
HRMS (ESI TOF): calcd. for $C_{21}H_{31}N_2O_3$ $[M+H]^+$ 359.2335, found 359.2343

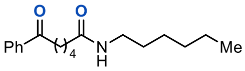

2-benzoyl-*N*-hexylpentanamide (2r)
Prepared according to general procedure B (405 nm, 6 hours). 4-nitrophthalonitrile (52 mg, 0.300 mmol, 1.5 equiv.), 6-fluoro-2,3,4,5-tetrahydro-1,1'-biphenyl (32.3 mg, 0.200 mmol) in acetonitrile (2.0 mL, 0.1 M). Two reactions run simultaneously were combined for isolation. NMR yield = 52%. The title compound was isolated via flash column chromatography (gradient 0 – 40% EtOAc/hexanes) as an off-white solid (47 mg, 41% yield).

^{1}**H NMR** (500 MHz, $CDCl_3$) (δ, ppm): 7.95 – 7.90 (m, 2H), 7.54 (t, *J* = 7.4 Hz, 1H), 7.44 (t, *J* = 7.6 Hz, 2H), 5.79 (t, *J* = 5.8 Hz, 1H), 3.22 (q, *J* = 6.9 Hz, 2H), 2.99 (t, *J* = 6.8 Hz, 2H), 2.22 (t, *J* = 7.0 Hz, 2H), 1.85 – 1.63 (m, 4H), 1.48 (p, *J* = 7.2 Hz, 2H), 1.35 – 1.21 (m, 6H), 0.86 (t, *J* = 6.7 Hz, 3H).
^{13}**C{^{1}H} NMR** (126 MHz, $CDCl_3$) (δ, ppm): 200.3, 172.7, 137.0, 133.2, 128.7, 128.1, 39.7, 38.3, 36.7, 31.6, 29.7, 26.7, 25.4, 23.8, 22.6, 14.1.
HRMS (ESI TOF): calcd. for $C_{18}H_{28}NO_2$ $[M+H]^+$ 290.2120, found 290.2120

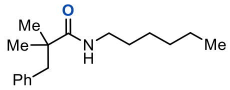

***N*-hexyl-2,2-dimethyl-3-phenylpropanamide (2s)**
Prepared according to general procedure B (405 nm, 6 hours). 4-nitrophthalonitrile (33 mg, 0.193 mmol, 1.5 equiv.), (E/*Z*)-1-(2-fluoro-3,3-dimethyl-4-phenylbut-1-en-1-yl)-4-methoxybenzene (36.6 mg, 0.129 mmol) in acetonitrile (1.29 mL, 0.1 M). NMR yield = 74%. The title compound was isolated via flash column chromatography (gradient 0 – 1% acetone:CH_2Cl_2) as a colorless oil (21.8 mg, 65% yield).

^{1}**H NMR** (500 MHz, $CDCl_3$) (δ, ppm): 7.24 (t, *J* = 7.3 Hz, 2H), 7.20 (d, *J* = 7.0 Hz, 1H), 7.14 – 7.09 (m, 2H), 5.46 – 5.27 (m, 1H), 3.20 – 3.12 (m, 2H), 2.83 (s, 2H), 1.38 (p, *J* = 7.2 Hz, 2H), 1.32 – 1.17 (m, 7H), 1.17 (s, 6H), 0.87 (t, *J* = 6.9 Hz, 3H).
^{13}**C{^{1}H} NMR** (126 MHz, $CDCl_3$) (δ, ppm): 176.7, 138.1, 130.1, 127.8, 126.2, 46.8, 43.2, 39.5, 31.3, 29.2, 26.4, 25.1, 22.4, 13.9.
HRMS (ESI TOF): calcd. for $C_{17}H_{28}NO$ $[M+H]^+$ 262.2171, found 262.2168

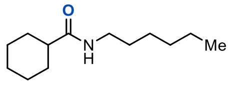

***N*-hexylcyclohexanecarboxamide (2t)**
Prepared according to general procedure B (405 nm, 6 hours). 4-nitrophthalonitrile (52 mg, 0.300 mmol, 1.5 equiv.), (E/*Z*)-1-(*tert*-butyl)-4-(2-cyclohexyl-2-fluorovinyl)benzene (52.1 mg, 0.200 mmol) in acetonitrile (2.0 mL, 0.1 M). Two reactions run simultaneously were combined for isolation. NMR yield = 84%. The title compound was isolated via flash column chromatography (gradient 10 – 15% EtOAc/hexanes) as a white solid (43.3 mg, 51% yield). All analytical data for **2t** was in accordance with literature data.[27]

^{1}**H NMR** (500 MHz, $CDCl_3$) (δ, ppm): 5.38 (s, 1H), 3.23 (q, *J* = 6.7 Hz, 2H), 2.04 (td, *J* = 11.9, 3.6 Hz, 1H), 1.90 – 1.55 (m, 5H), 1.51 – 1.15 (m, 13H), 0.87 (d, *J* = 7.0 Hz, 3H).
^{13}**C{^{1}H} NMR** (126 MHz, $CDCl_3$) (δ, ppm): 176.1, 45.8, 39.5, 31.6, 29.9, 29.89, 29.8, 26.7, 25.9, 22.7, 14.2.

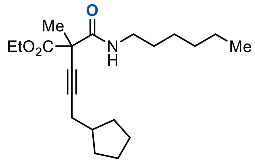

5-cyclopentyl-2-((ethylperoxy)-λ2-methyl)-*N*-hexyl-2-methylpent-3-ynamide (2u)
Prepared according to general procedure B (405 nm, 10 hours). 4-nitrophthalonitrile (52 mg, 0.300 mmol, 1.5 equiv.), (*Z*)-4-(6-cyclopentyl-3-((ethylperoxy)-λ2-methyl)-2-fluoro-3-methylhex-1-en-4-yn-1-yl)benzonitrile (70.7 mg, 0.200 mmol) in acetonitrile (2.0 mL, 0.1 M). Two reactions run simultaneously were combined for isolation. NMR yield = 62%. The title

compound was isolated via flash column chromatography (0 – 7% EtOAc/hexanes gradient then 1:1 DCM/hexanes) as a yellow oil (56.8 mg, 45% yield).

^{1}H NMR (500 MHz, $CDCl_3$) (δ, ppm): 6.80 (t, J = 6.1 Hz, 1H), 4.20 (qd, J = 7.1, 5.5 Hz, 2H), 3.35 – 3.18 (m, 2H), 2.26 (d, J = 6.7 Hz, 2H), 2.06 (p, J = 7.4 Hz, 1H), 1.88 – 1.72 (m, 2H), 1.70 – 1.46 (m, 9H), 1.40 – 1.20 (m, 12H), 0.98 – 0.78 (m, 3H).
^{13}C{^{1}H} NMR (126 MHz, $CDCl_3$) (δ, ppm): 169.2, 168.5, 87.1, 78.2, 62.3, 50.4, 40.2, 39.0, 32.1, 32.0, 31.6, 29.4, 26.6, 25.4, 24.7, 23.3, 22.7, 14.1.
HRMS (ESI TOF): calcd. for $C_{20}H_{34}NO_3$ $[M+H]^+$ 336.2539, found 336.2539

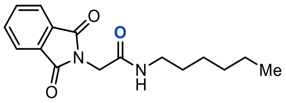

2-(1,3-dioxoisoindolin-2-yl)-*N*-hexylacetamide (2v)
Prepared according to general procedure B (2 405 nm lamps, 36 hours). 4-nitrophthalonitrile (260 mg, 1.50 mmol, 1.5 equiv.), (*Z*)-2-(2-fluoro-3-(*p*-tolyl)allyl)isoindoline-1,3-dione (295 mg, 1.00 mmol) in acetonitrile (10.0 mL, 0.1 M). Two reactions run simultaneously were combined for isolation. The title compound was isolated via flash column chromatography (gradient 0 – 7% EtOAc/hexanes) as a light pink solid (150 mg, 63% yield).

^{1}H NMR (500 MHz, $CDCl_3$) (δ, ppm): 7.92 – 7.85 (m, 2H), 7.78 – 7.71 (m, 2H), 5.69 (s, 1H), 4.32 (s, 2H), 3.27 (td, J = 7.3, 5.8 Hz, 2H), 1.53 – 1.43 (m, 2H), 1.36 – 1.24 (m, 6H), 0.91 – 0.84 (m, 3H).
^{13}C{^{1}H} NMR (126 MHz, $CDCl_3$) (δ, ppm): 167.9, 166.0, 134.4, 132.2, 123.8, 41.1, 40.1, 31.5, 29.6, 26.6, 22.7, 14.1.
HRMS (ESI TOF): calcd. for $C_{16}H_{21}N_2O_3$ $[M+H]^+$ = 289.1552, found 289.1550

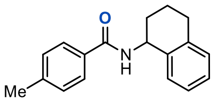

4-methyl-N-(1,2,3,4-tetrahydronaphthalen-1-yl)benzamide (3a)
Prepared according to general procedure B (405 nm, 6 hours). 4-nitrophthalonitrile (51.9 mg, 0.300 mmol, 1.5 equiv.), 1-(1-fluorovinyl)-4-methylbenzene (27.2 mg, 0.200 mmol), in acetonitrile (2.0 mL, 0.1 M). 1,2,3,4-tetrahydronaphthalen-1-amine (43.0 µL, 0.300 mmol, 1.5 equiv.) was added after starting material consumption. Two reactions run simultaneously were combined for isolation. NMR yield = 56%. The title compound was isolated via flash column chromatography (gradient 10 - 15 % EtOAc/hexanes) as a pale orange solid (42.4 mg, 40% yield).

^{1}H NMR (500 MHz, $CDCl_3$) (δ, ppm): 7.68 (d, J = 7.9 Hz, 2H), 7.37 – 7.31 (m, 1H), 7.24 – 7.11 (m, 5H), 6.29 (d, J = 8.4 Hz, 1H), 5.43 – 5.36 (m, 1H), 2.83 (qt, J = 16.7, 6.2 Hz, 2H), 2.39 (s, 3H), 2.15 (tt, J = 11.2, 5.3 Hz, 1H), 1.97 – 1.86 (m, 3H).
^{13}C{^{1}H} NMR (126 MHz, $CDCl_3$) (δ, ppm): 166.8, 142.0, 137.9, 136.9, 132.0, 129.4, 129.3, 129.0, 127.5, 127.1, 126.5, 48.0, 30.4, 29.5, 21.6, 20.2.
HRMS (ESI TOF): calcd. for $C_{18}H_{19}NONa$ $[M+Na]^+$ 288.1364, found 288.1378

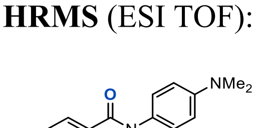

N-(4-(dimethylamino)phenyl)-4-methylbenzamide (3b)

Prepared according to general procedure B (405 nm, 6 hours). 4-nitrophthalonitrile (51.9 mg, 0.300 mmol, 1.5 equiv.), 1-(1-fluorovinyl)-4-methylbenzene (27.3 mg, 0.200 mmol), in acetonitrile (2.0 mL, 0.1 M). N,N-dimethylbenzene-1,4-diamine (40.9 mg, 0.300 mmol, 1.5 equiv.) was added after starting material consumption. NMR yield = 81%. The title compound was isolated via flash column chromatography (gradient 10-20% EtOAc/hexanes) as a light orange solid (35.6 mg, 70% yield).

^{1}H NMR (500 MHz, $CDCl_3$) (δ, ppm): δ 7.76 (d, *J* = 7.7 Hz, 2H), 7.62 (s, 1H), 7.48 (d, *J* = 8.5 Hz, 2H), 7.33 – 7.26 (m, 2H), 6.77 – 6.71 (m, 2H), 2.94 (s, 6H), 2.42 (s, 3H).
^{13}C{^{1}H} NMR (126 MHz, $CDCl_3$) (δ, ppm): 165.5, 148.3, 142.8, 132.6, 129.5, 128.0, 127.1, 122.1, 113.2, 41.1, 21.6.
HRMS (ESI TOF): calcd. for $C_{16}H_{18}N_2ONa$ $[M+Na]^+$ 277.1317, found 277.1318

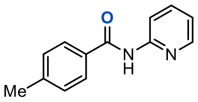

4-methyl-N-(pyridin-2-yl)benzamide (3c)
Prepared according to general procedure B (405 nm, 6 hours). 4-nitrophthalonitrile (51.9 mg, 0.300 mmol, 1.5 equiv.), 1-(1-fluorovinyl)-4-methylbenzene (27.3 mg, 0.200 mmol), in acetonitrile (2.0 mL, 0.1 M). Pyridin-2-amine (28.2 mg, 0.300 mmol, 1.5 equiv.) was added after starting material consumption at 100 °C using a heating block. Two reactions run simultaneously were combined for isolation. NMR yield = 22%. The title compound was isolated via flash column chromatography (gradient 10-20% EtOAc/hexanes) as an off-white solid (15.4 mg, 18% yield).

^{1}H NMR (500 MHz, $CDCl_3$) (δ, ppm): 8.55 (s, 1H), 8.39 (d, *J* = 8.4 Hz, 1H), 8.30 (dd, *J* = 5.1, 1.9 Hz, 1H), 7.83 (d, *J* = 8.3 Hz, 2H), 7.75 (ddd, *J* = 8.7, 7.4, 2.0 Hz, 1H), 7.30 (d, *J* = 7.9 Hz, 2H), 7.07 (ddd, *J* = 7.4, 4.9, 1.0 Hz, 1H), 2.43 (s, 3H).
^{13}C{^{1}H} NMR (126 MHz, $CDCl_3$) (δ, ppm): 165.7, 151.8, 148.1, 143.1, 138.6, 134.1, 131.5, 129.7, 127.4, 120.9, 120.0, 114.2, 21.7.
HRMS (ESI TOF): calcd. for $C_{13}H_{13}N_2O$ $[M+H]^+$ 213.1028, found 213.1031

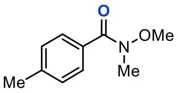

N-methoxy-N,4-dimethylbenzamide (3d)
Prepared according to general procedure B (405 nm, 6 hours). 4-nitrophthalonitrile (51.9 mg, 0.300 mmol, 1.5 equiv), 1-(1-fluorovinyl)-4-methylbenzene (27.2 mg, 0.200 mmol), in acetonitrile (2.0 mL, 0.1 M). N,O-dimethylhydroxylamine (18.3 mg, 0.300 mmol, 1.5 equiv) was added after starting material consumption. Two reactions run simultaneously were combined for isolation. NMR yield = 33%. The title compound was isolated via flash column chromatography (gradient 20-40 % EtOAc/hexanes) as a colorless oil (54.2 mg, 28% yield). All analytical data for **3d** was in accordance with literature data.[28]

^{1}H NMR (500 MHz, $CDCl_3$) (δ, ppm): 7.62 – 7.57 (m, 2H), 7.20 (d, *J* = 7.9 Hz, 2H), 3.56 (s, 3H), 3.35 (s, 3H), 2.39 (s, 3H).
^{13}C{^{1}H} NMR (126 MHz, $CDCl_3$) (δ, ppm): 170.1, 141.0, 131.3, 128.8, 128.5, 61.1, 34.0, 21.6.

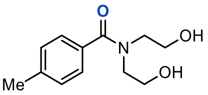

N,N-bis(2-hydroxyethyl)-4-methylbenzamide (3e)
Prepared according to general procedure B (405 nm, 6 hours). 4-nitrophthalonitrile (51.9 mg, 0.300 mmol, 1.5 equiv.), 1-(1-fluorovinyl)-4-methylbenzene (27.2 mg, 0.200 mmol), in acetonitrile (2.0 mL, 0.1 M). 2,2'-azanediylbis(ethan-1-ol) (31.5 mg, 0.300 mmol, 1.5 equiv.) was added after starting material consumption. Two reactions run simultaneously were combined for isolation. NMR yield = 61%. The title compound was isolated via flash column chromatography (gradient 0-5 % MeOH/CH_2Cl_2) as a pale yellow oil (47.0 mg, 53% yield).

^{1}H NMR (500 MHz, $CDCl_3$) (δ, ppm): 7.39 (d, *J* = 7.8 Hz, 2H), 7.20 (d, *J* = 7.8 Hz, 2H), 3.99 (s, 2H), 3.83 – 3.67 (m, 4H), 3.56 – 3.15 (m, 4H), 2.37 (s, 3H).
^{13}C{^{1}H} NMR (101 MHz, $CDCl_3$) (δ, ppm): 207.2, 174.3, 139.9, 133.4, 129.2, 127.5, 31.1, 21.5.
HRMS (ESI TOF): calcd. for $C_{12}H_{18}NO_3$ $[M+H]^+$ 224.1287, found 224.1303

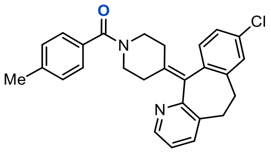

(4-(8-chloro-5,6-dihydro-11H-benzo[5,6]cyclohepta[1,2-b]pyridin-11-ylidene)piperidin-1-yl)(p-tolyl)methanone (3f)

Prepared according to general procedure B (405 nm, 6 hours). 4-nitrophthalonitrile (51.9 mg, 0.300 mmol, 1.5 equiv), 1-(1-fluorovinyl)-4-methylbenzene (27.2 mg, 0.200 mmol) in acetonitrile (2.0 mL, 0.1 M). Desloratadine (93.2 mg, 0.300 mmol, 1.5 equiv) was added after starting material consumption. Two reactions run simultaneously were combined for isolation. NMR yield = 46%. The title compound was isolated via flash column chromatography (2% MeOH/CH_2Cl_2) as an off-white solid (54.5 mg, 32% yield).

^{1}H NMR (500 MHz, $CDCl_3$) (δ, ppm): 8.38 (d, *J* = 11.2 Hz, 1H), 7.42 (d, *J* = 7.7 Hz, 1H), 7.29 (d, *J* = 7.9 Hz, 2H), 7.17 – 7.06 (m, 6H), 4.17 (s, 1H), 3.67 (d, *J* = 20.7 Hz, 1H), 3.43 – 3.11 (m, 4H), 2.90 – 2.74 (m, 2H), 2.47-2.30 (m, 7H).
^{13}C{^{1}H} NMR (126 MHz, $CDCl_3$) (δ, ppm): 170.7, 156.9, 146.8, 139.8, 139.7, 137.7, 137.1, 134.7, 133.5, 133.2, 130.6, 129.1, 127.1, 126.3, 122.4, 48.7, 43.4, 31.8, 31.7, 30.6, 21.4.
HRMS (ESI TOF): calcd. for $C_{27}H_{26}ClN_2O$ $[M+H]^+$ 429.1734, found 429.1737

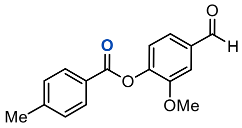

4-formyl-2-methoxyphenyl 4-methylbenzoate (3g)
Prepared according to general procedure B (405 nm, 6 hours). 4-nitrophthalonitrile (51.9 mg, 0.300 mmol mmol, 1.5 equiv.), 1-(1-fluorovinyl)-4-methylbenzene (27.2 mg, 0.200 mmol) in acetonitrile (2.0 mL, 0.1 M). Vanillin (36.5 mg, 0.240 mmol, 1.2 equiv.), TEA (30.7 µL, 0.220 mmol, 1.1 equiv.), and DMAP (7.33 mg, 0.200 mmol, 0.4 equiv.) was added after starting material consumption. Two reactions run simultaneously were combined for isolation. NMR yield = 42%. The title compound was isolated via flash column chromatography (gradient 0 – 10% EtOAc/hexanes) as a white solid (43.3 mg, 40% yield).

^{1}H NMR (500 MHz, $CDCl_3$) (δ, ppm): 9.98 (s, 1H), 8.13 – 8.07 (m, 2H), 7.56 – 7.50 (m, 2H), 7.38 – 7.30 (m, 3H), 3.89 (s, 3H), 2.46 (s, 3H).

$^{13}C\{^1H\}$ NMR (126 MHz, $CDCl_3$) (δ, ppm): 191.3, 164.4, 152.4, 145.5, 144.9, 135.4, 130.6, 129.5, 126.2, 125.0, 123.8, 111.0, 56.3, 21.9.
HRMS (ESI TOF): calcd. for $C_{16}H_{15}O_4$ $[M+H]^+$ 271.0970, found 271.0971

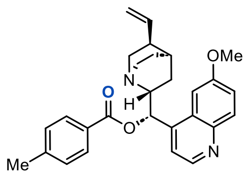

(R)-(6-methoxyquinolin-4-yl)((1S,2S,4S,5R)-5-vinylquinuclidin-2-yl)methyl 4-methylbenzoate (3h)

Prepared according to general procedure B (405 nm, 6 hours). 4-nitrophthalonitrile (51.9 mg, 0.300 mmol mmol, 1.5 equiv.), 1-(1-fluorovinyl)-4-methylbenzene (27.2 mg, 0.200 mmol) in acetonitrile (2.0 mL, 0.1 M). Upon complete consumption of the alkenyl fluoride as determined by GCMS analysis, the reaction was concentrated under reduced pressure. The mixture was then diluted with 2 mL of diethyl ether and stirred for 5 minutes. The mixture was filtered through celite to remove solids, and the mixture was concentrated under reduced pressure. The mixture was then diluted with 2 mL of acetonitrile. Quinine (77.9 mg, 0.240 mmol, 1.2 equiv.), DMAP (7.33 mg, 0.060 mmol, 0.3 equiv.), and TEA (30.7 µL, 0.220 mmol, 1.1 equiv.) were added and the mixture was stirred for 2 hours. The solvent was removed and the crude product was purified by column chromatography. Two reactions run simultaneously were combined for isolation.NMR yield = 32%. The title compound was isolated via flash column chromatography (gradient 10 – 40% acetone/hexanes) as a white solid (49.8 mg, 28% yield).Analytical data for **3h** was in accordance to literature data.[29]

^1H NMR (600 MHz, $CDCl_3$) (δ, ppm): 8.71 (d, J = 4.5 Hz, 1H), 8.00 (dd, J = 16.0, 8.7 Hz, 3H), 7.54 – 7.49 (m, 1H), 7.47 – 7.34 (m, 2H), 7.27 (d, J = 8.0 Hz, 2H), 6.75 (d, J = 6.1 Hz, 1H), 5.84 (ddd, J = 17.4, 10.4, 7.4 Hz, 1H), 5.06 – 4.98 (m, 2H), 3.98 (s, 3H), 3.50 (td, J = 8.7, 3.9 Hz, 1H), 3.23 (s, 1H), 3.15 – 3.08 (m, 1H), 2.78 – 2.62 (m, 2H), 2.43 (s, 3H), 2.32 (m, 1H), 1.95 – 1.87 (m, 2H), 1.81 – 1.73 (m, 2H), 1.59 (m, 1H).
$^{13}C\{^1H\}$ NMR (151 MHz, $CDCl_3$) (δ, ppm): 165.7, 158.2, 147.6, 145.0, 144.5, 143.8, 141.7, 132.0, 129.9, 129.5, 127.2, 127.0, 122.1, 118.8, 114.8, 101.6, 74.3, 59.5, 56.8, 55.8, 42.7, 39.8, 27.8, 27.2, 24.1, 21.9.

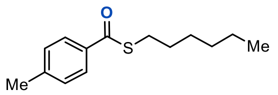

S-hexyl 4-methylbenzothioate (3i)
Prepared according to general procedure B (405 nm, 6 hours). 4-nitrophthalonitrile (51.9 mg, 0.300 mmol, 1.5 equiv.), 1-(1-fluorovinyl)-4-methylbenzene (27.2 mg, 0.200 mmol) in acetonitrile (2.0 mL, 0.1 M). Hexanethiol (42.4 µL, 0.300 mmol, 1.5 equiv.), TEA (39.9 µL, 1.5 equiv.) and DMAP (7.33 mg, 0.060 mmol, 0.3 equiv.) was added after starting material consumption. Two reactions run simultaneously were combined for isolation. NMR yield = 42%. The title compound was isolated via flash column chromatography (gradient 0 – 10% EtOAc/hexanes) as a pale-yellow oil (36.8 mg, 40% yield). All analytical data for **3i** was in accordance with literature data.[30]

^1H NMR (500 MHz, $CDCl_3$) (δ, ppm): 7.87 (d, J = 7.9 Hz, 2H), 7.24 (d, J = 7.9 Hz, 2H), 3.05 (t, J = 7.4 Hz, 2H), 2.40 (s, 3H), 1.66 (p, J = 7.5 Hz, 2H), 1.43 (p, J = 7.2 Hz, 2H), 1.37 – 1.27 (m, 4H), 0.93 – 0.86 (m, 3H).

$^{13}C\{^1H\}$ **NMR** (126 MHz, $CDCl_3$) (δ, ppm): 191.9, 144.1, 134.9, 129.4, 127.4, 31.5, 29.7, 29.1, 28.8, 22.7, 21.8, 14.2.
HRMS (ESI TOF): calcd. for $C_{14}H_{21}OS$ $[M+H]^+$ 237.1313, found 237.1313

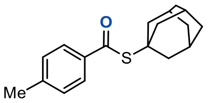

S-((3R,5S)-adamantan-1-yl) 4-methylbenzothioate (3j)
Prepared according to general procedure B (405 nm, 6 hours). 4-nitrophthalonitrile (51.9 mg, 0.300 mmol, 1.5 equiv.), 1-(1-fluorovinyl)-4-methylbenzene (27.2 mg, 0.200 mmol) in acetonitrile (2.0 mL, 0.1 M). Adamantane-1-thiol (50.5 mg, 0.300 mmol, 1.5 equiv.), TEA (39.9 µL, 1.5 equiv.) and DMAP (7.33 mg, 0.060 mmol, 0.3 equiv.) was added after starting material consumption. Two reactions run simultaneously were combined for isolation. NMR yield = 42%. The title compound was isolated via flash column chromatography (gradient 0 – 10% EtOAc/hexanes) as a white solid (47.0 mg, 41% yield).

^{1}**H NMR** (400 MHz, $CDCl_3$) (δ, ppm): 7.80 (d, J = 8.1 Hz, 2H), 7.20 (d, J = 8.0 Hz, 2H), 2.39 (s, 3H), 2.26 (d, J = 2.9 Hz, 6H), 2.09 (s, 3H), 1.85 – 1.70 (m, 6H).
$^{13}C\{^1H\}$ **NMR** (101 MHz, $CDCl_3$) (δ, ppm): 192.4, 143.7, 136.1, 129.2, 127.2, 51.1, 42.2, 36.6, 30.1, 21.8.
HRMS (ESI TOF): calcd. for $C_{18}H_{23}OS$ $[M+H]^+$ 287.1470, found 287.1473

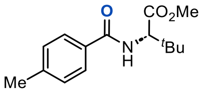

methyl (S)-3,3-dimethyl-2-(4-methylbenzamido)butanoate (3k)
Prepared according to general procedure B (405 nm, 6 hours). 4-nitrophthalonitrile (51.9 mg, 0.300 mmol, 1.5 equiv.), 1-(1-fluorovinyl)-4-methylbenzene (27.2 mg, 0.200 mmol), methyl (S)-2-amino-3,3-dimethylbutanoate (43.6 mg, 0.300 mmol, 1.5 equiv.) in acetonitrile (2.0 mL, 0.1 M) Two reactions run simultaneously were combined for isolation. NMR yield = 62%. The title compound was isolated via flash column chromatography (gradient 0-15% EtOAc/hexanes) as a yellow oil (54.2 mg, 52% yield).

^{1}**H NMR** (500 MHz, $CDCl_3$) (δ, ppm): 7.72 – 7.67 (m, 2H), 7.25 (d, J = 8.2 Hz, 2H), 6.60 (d, J = 9.4 Hz, 1H), 4.70 (d, J = 9.4 Hz, 1H), 3.75 (s, 3H), 2.40 (s, 3H), 1.05 (s, 9H)
$^{13}C\{^1H\}$ **NMR** (126 MHz, $CDCl_3$) (δ, ppm): 172.5, 167.2, 142.3, 131.6, 129.4, 127.2, 60.3, 52.0, 35.3, 26.8, 21.6
HRMS (ESI TOF): calcd. for $C_{15}H_{21}NO_3Na$ $[M+Na]^+$ 286.1419, found 286.1432

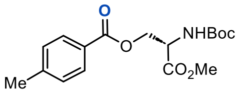

(S)-2-((tert-butoxycarbonyl)amino)-3-methoxy-3-oxopropyl 4-methylbenzoate (3l)
Prepared according to general procedure B (405 nm, 6 hours). 4-nitrophthalonitrile (51.9 mg, 0.300 mmol, 1.5 equiv.), 1-(1-fluorovinyl)-4-methylbenzene (27.2 mg, 0.200 mmol) in acetonitrile (2.0 mL, 0.1 M). Upon complete consumption of the alkenyl fluoride as determined by GCMS analysis, the reaction was concentrated under reduced pressure. The mixture was then diluted with 2 mL of diethyl ether and stirred for 5 minutes. The mixture was filtered through celite to remove solids and the mixture was concentrated under reduced pressure. The mixture was then diluted with 2 mL of acetonitrile. N-[(1,1-Dimethylethoxy)carbonyl]-D-serine methyl ester (48.7 µL, 0.240 mmol, 1.2 equiv.), DMAP (7.33 mg, 0.060 mmol, 0.3

equiv.), and TEA (30.7 μL, 0.220 mmol, 1.1 equiv.) were added and the mixture was stirred for 2 hours. The solvent was removed and the crude product was purified by column chromatography. Two reactions run simultaneously were combined for isolation. NMR yield = 42%. The title compound was isolated via flash column chromatography (gradient 0-20 % EtOAc/hexanes) as a white solid (55.1 mg, 40% yield).

^{1}H NMR (600 MHz, $CDCl_3$) (δ, ppm): 7.90 – 7.86 (m, 2H), 7.29 – 7.20 (m, 2H), 5.37 (d, J = 8.6 Hz, 1H), 4.71 (dt, J = 8.5, 4.0 Hz, 1H), 4.66 – 4.54 (m, 2H), 3.78 (s, 3H), 2.41 (s, 3H), 1.45 (s, 9H).
$^{13}C\{^1H\}$ NMR (151 MHz, $CDCl_3$) (δ, ppm): 170.6, 166.2, 155.3, 144.7, 129.9, 129.3, 126.8, 80.5, 65.0, 52.9, 28.4, 27.2, 21.8.
HRMS (ESI TOF): calcd. for $C_{17}H_{23}NO_6Na$ $[M+Na]^+$ = 360.1423, found 360.1425

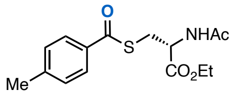

ethyl N-acetyl-S-(4-methylbenzoyl)-L-cysteinate (3m)
Prepared according to general procedure B (405 nm, 6 hours). 4-nitrophthalonitrile (130 mg, 0.750 mmol, 1.5 equiv.), 1-(1-fluorovinyl)-4-methylbenzene (68.1 mg, 0.500 mmol) in acetonitrile (5.0 mL, 0.1 M). Ethyl acetyl-L-cysteinate (143 mg, 0.750 mmol, 1.5 equiv.) and DMAP (24.4 mg, 0.200 mmol, 0.4 equiv.) was added after starting material consumption. NMR yield = 72%. The title compound was isolated via flash column chromatography (gradient 0 – 60% EtOAc/hexanes) as a pink/beige solid (87 mg, 56% yield).

^{1}H NMR (500 MHz, $CDCl_3$) (δ, ppm): 7.85 (d, J = 8.0 Hz, 2H), 7.25 (s, 2H), 6.32 (d, J = 7.5 Hz, 1H), 4.87 (dt, J = 7.5, 5.4 Hz, 1H), 4.22 (q, J = 7.2 Hz, 2H), 3.57 (dd, J = 5.4, 1.1 Hz, 2H), 2.42 (s, 3H), 2.00 (s, 3H), 1.29 (t, J = 7.1 Hz, 3H).
$^{13}C\{^1H\}$ NMR (126 MHz, $CDCl_3$) (δ, ppm): 190.8, 170.3, 170.0, 144.9, 133.9, 129.4, 127.5, 62.1, 52.5, 23.1, 21.7, 14.1.
HRMS (ESI TOF): calcd. for $C_{15}H_{20}NO_4S$ $[M+H]^+$ 310.1113, found 310.1116

Mechanistic studies
Proposed mechanism

Based on our prior[31] and additional mechanistic studies (Table S3), we proposed the following mechanism (Scheme S1). Photoexcitation of the nitroarene in the presence of the alkenyl fluoride leads to the 4-fluoro-1,3,2-dioxazolidine (I) intermediate, which undergoes spontaneous fragmentation to yield the acyl fluoride and carbonyl amine IV (pathway a). NAS of the in-situ generated acyl fluoride furnishes the acyl coupling product, while the carbonyl imine IV then reacts with the sacrificial aldehyde to form a second dioxazolidine (V) intermediate to avoid undesired cyclization with the key acyl fluoride intermediate, ultimately leading to the carbonyl cleavage byproduct. Fragmentation of I can also lead to the carbonyl cleavage product and the α-fluorinated carbonyl imine (II, pathway b). The latter undergoes cyclization with the carbonyl product, potentially leading to dioxazolidine III, which, upon fragmentation, rescues the acyl fluoride. Once irradiation is completed, the acyl fluoride is now primed to undergo NAS with the amine nucleophile. Confirmation by crude ^{19}F NMR followed by isolation proved that acyl fluorides are produced from the cleavage reaction. As for the fate of the fluoride ion, we hypothesize that any HF produced would get quenched with the slight excess of amine nucleophile. To our knowledge, we see no evidence that much HF is produced, as the reaction vessels do not become etched.

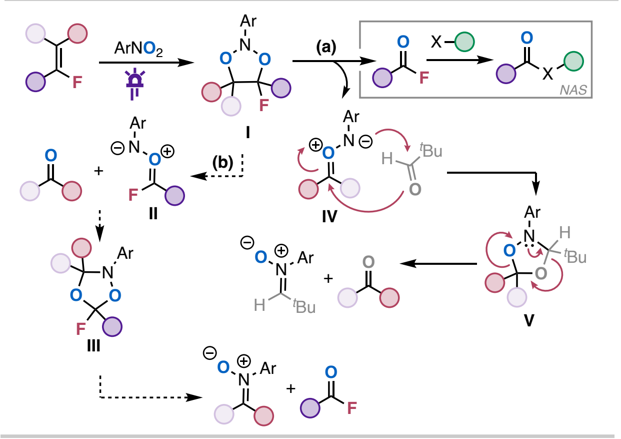

Scheme S1: Proposed mechanism

Isolation of acyl fluoride

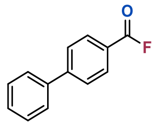

[1,1'-biphenyl]-4-carbonyl fluoride (1'f)

Prepared according to general procedure B (405 nm, 8 hours). 4-nitrophthalonitrile (51.9 mg, 0.300 mmol, 1.5 equiv.), 4-(1-fluorovinyl)-1,1'-biphenyl (39.6 mg, 0.200 mmol) in acetonitrile (2.0 mL, 0.1 M). Two reactions run simultaneously were combined for isolation. The title compound was isolated via flash column chromatography (1-2% EtOAc (0.1% AcOH)/hexanes) as a white solid (36.5 mg, 46% yield). All analytical data for **1f'** was in accordance with literature data.[32]

^{1}H NMR (500 MHz, $CDCl_3$) (δ, ppm): 8.15 – 8.09 (m, 2H), 7.75 (d, J = 8.4, 1.2 Hz, 2H), 7.68 – 7.61 (m, 2H), 7.57 – 7.47 (m, 2H), 7.47 – 7.41 (m, 1H).
^{13}C{^{1}H} NMR (101 MHz, $CDCl_3$) (δ, ppm): 157.5 (d, J_{C-F} = 343.3 Hz), 148.3, 139.4, 132.1 (d, J_{C-F} = 3.9 Hz), 129.3, 128.9, 127.8 (d, J_{C-F} = 1.3 Hz), 127.5, 123.6 (d, J_{C-F} = 61.3 Hz).
^{19}F{^{1}H} NMR (337 MHz, $CDCl_3$) (δ, ppm): 18.14.

^{1}H NMR (500 MHz, $CDCl_3$) of [1,1'-biphenyl]-4-carbonyl fluoride (1'f)

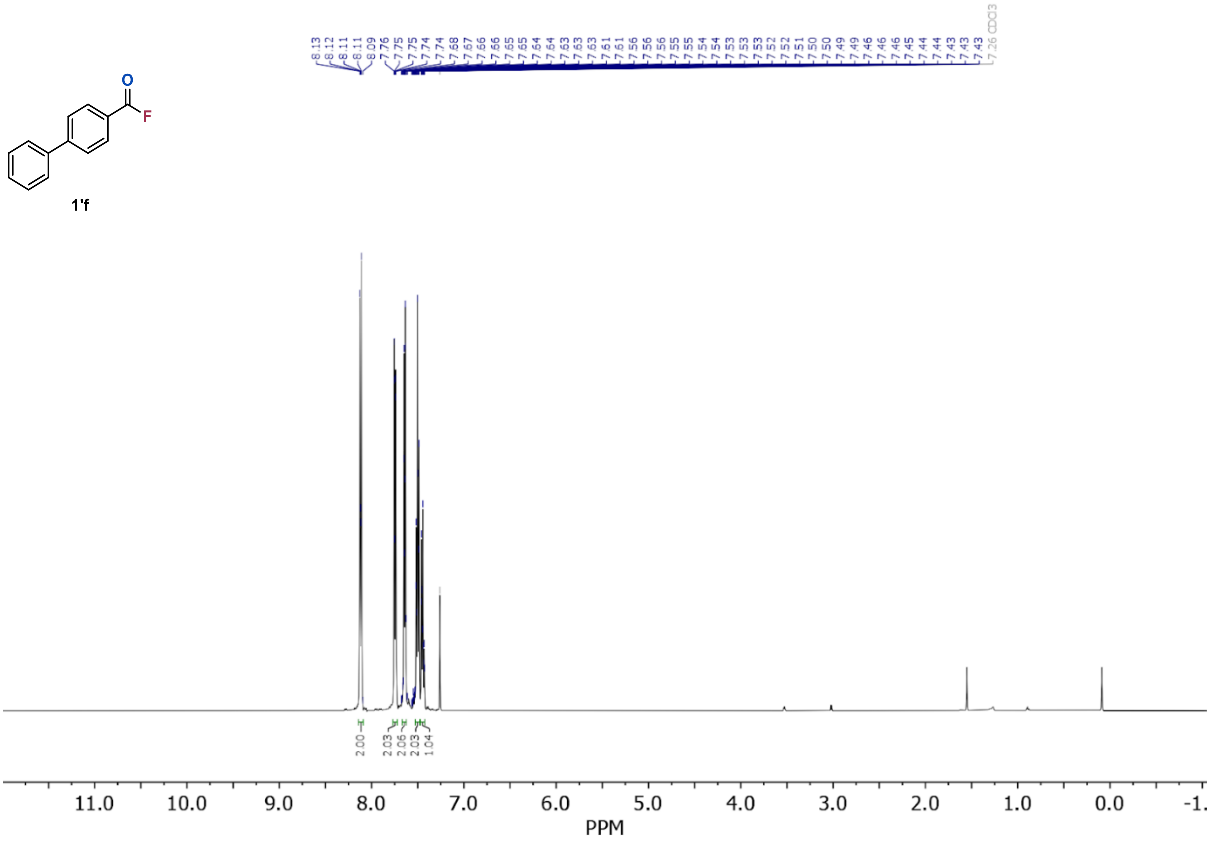

$^{13}C\{^1H\}$ NMR (101 MHz, $CDCl_3$) of [1,1'-biphenyl]-4-carbonyl fluoride (1’f)

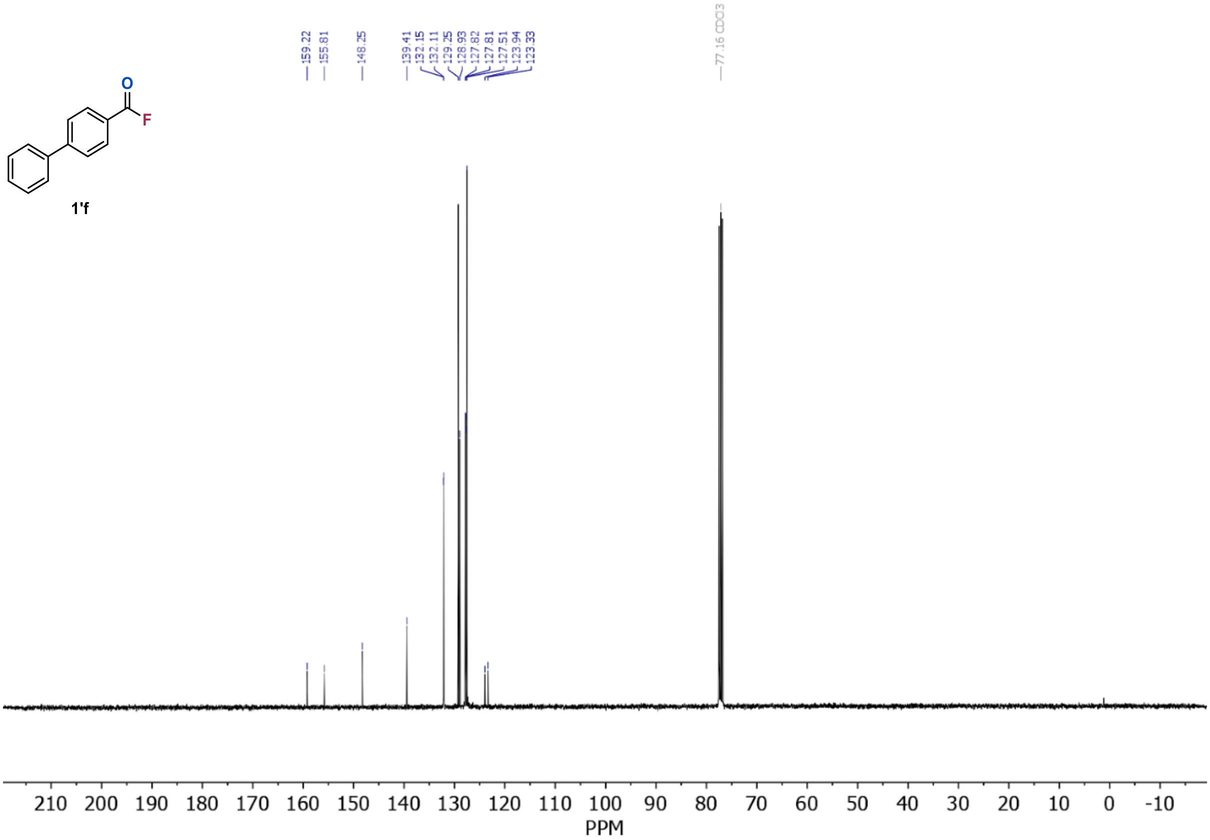

$^{19}F\{^1H\}$ NMR (337 MHz, $CDCl_3$) of [1,1'-biphenyl]-4-carbonyl fluoride (1’f)

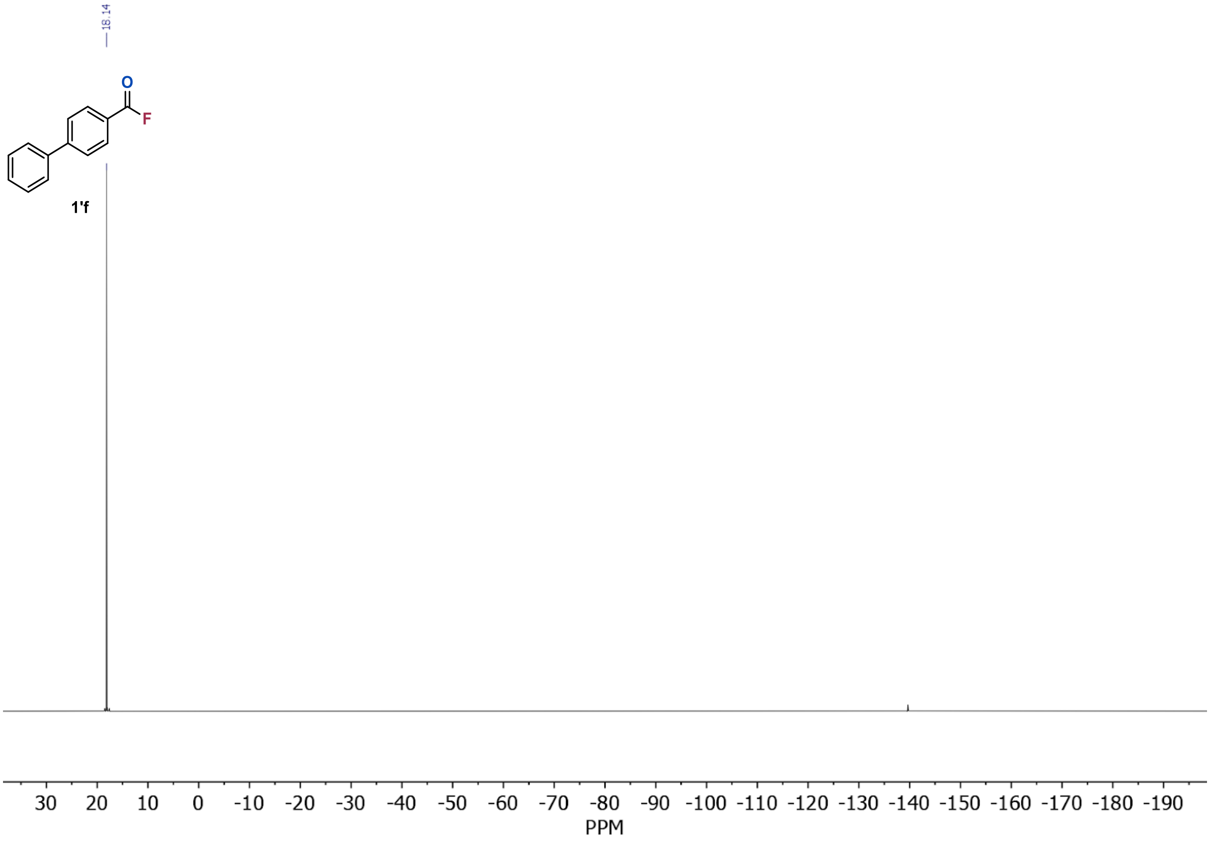

Selectivity of Alkene vs Fluoroalkenes under reaction conditions

1-methyl-4-vinylbenzene vs 1-(1-fluorovinyl)-4-methylbenzene

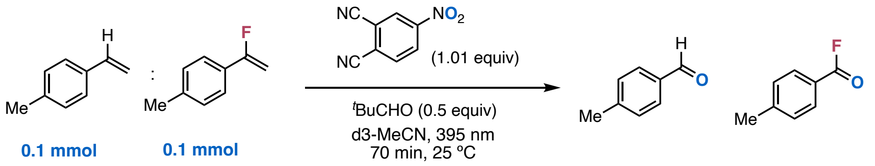

Reaction Setup according to general procedure B in a 1-dram vial. 4-nitrophtalonitrile (17.5 mg, 1.01 equiv, 0.101 mmol), 1-methyl-4-vinylbenzene (11.7 µL, 1 equiv, 0.100 mmol), 1-(1-fluorovinyl)-4-methylbenzene (13.6 mg, 1 equiv, 0.100 mmol), pivaldehyde (5.5 µL, 0.5 equiv, 50.0 µmol) in CD_3CN (1.0 mL, 0.1M). The mixture was then transferred to a N_2 purged PhotoNMR tube equipped with an innder cell for an optical insert (part number: NE:379-5-A). A coaxial cable, connected to an ultra-high power 395 nm LED was inserted and and sample was manually inserted to a Bruker NMR at 25 ºC. The reaction was monitored by 1H NMR spectroscopy over the course of 70 minutes. For each 1H NMR spectrum 16 scans were acquired for a total data collection time of 105 seconds. The first scan was run before the light was turned on as time = 0.

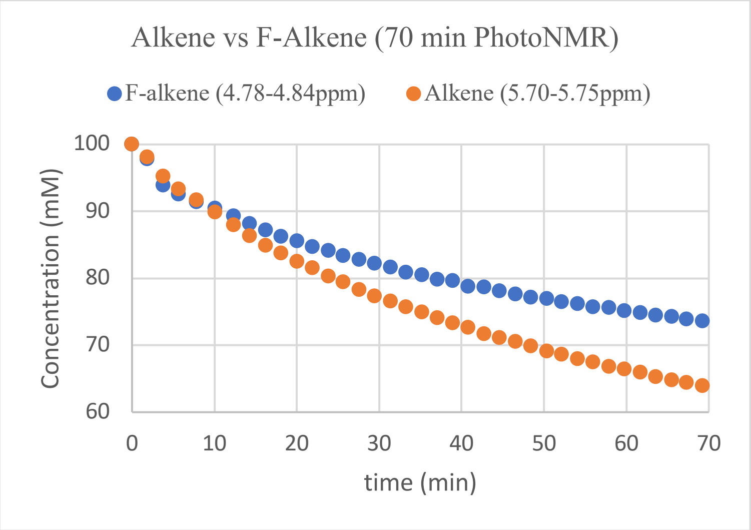

Figure S1:Concentration versus time graph of consumption of fluoroalkene and methyl-styrene over the course of 70 minutes. Monitored from ^{1}H NMR.

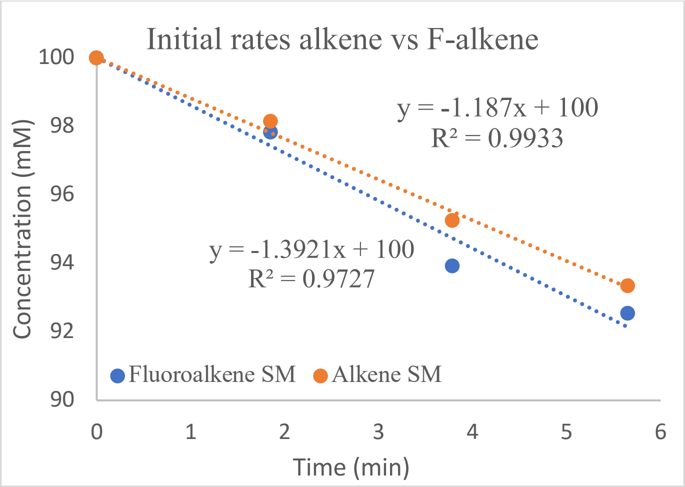

Figure S2: Initial rates from concentration versus time graph of consumption of fluoroalkene and methyl-styrene over the course of 5 min. Monitored by ^{1}H NMR

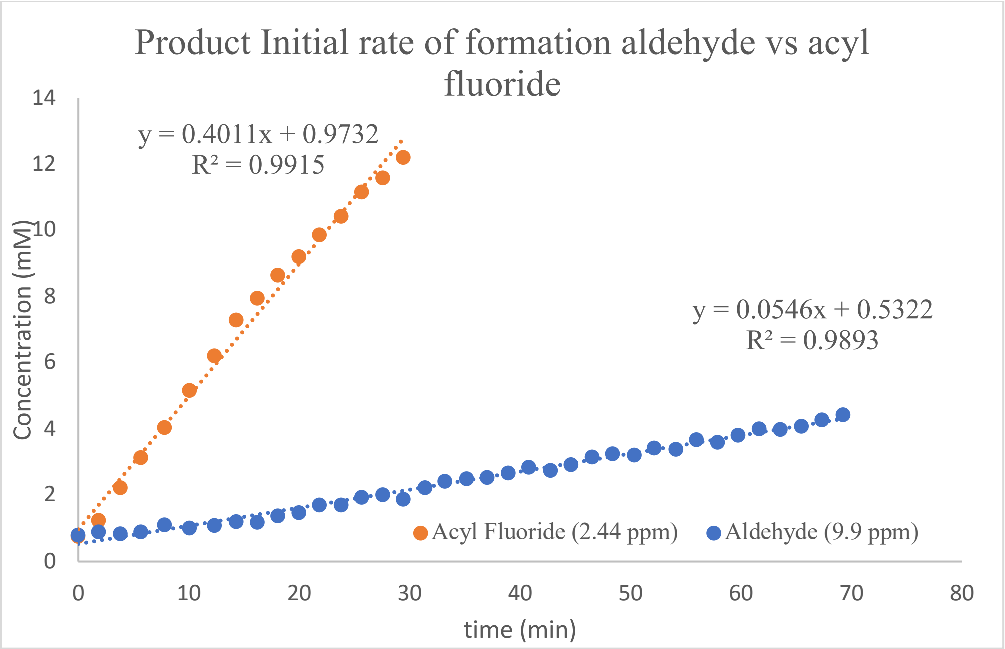

Figure S3: Initial rates of product formation from concentration versus time graph of acyl fluoride and benzaldehyde over the course of 1H NMR monitoring.

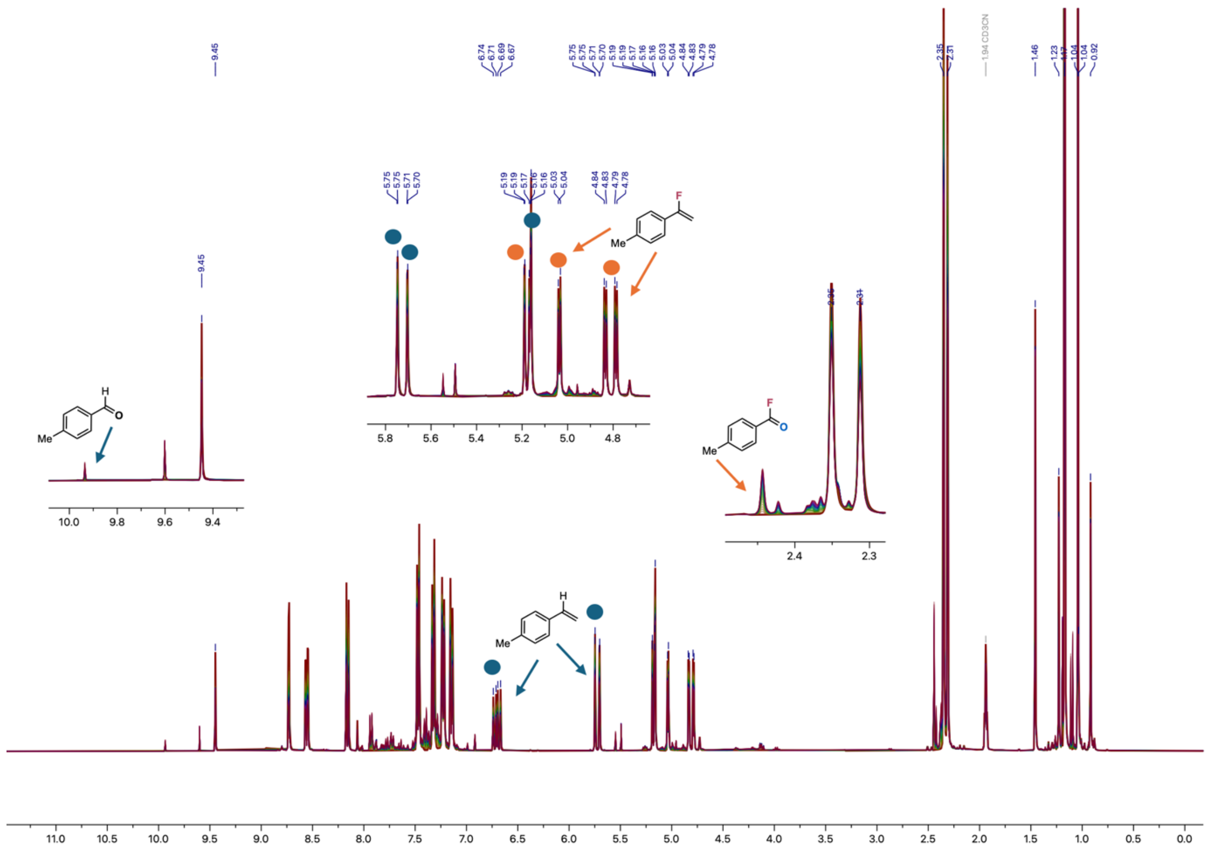

Figure S4: superimposed ^{1}H NMR Spectra over the course of 70 minutes with key peaks denoted that were used to monitor reaction progress.

Analysis: Based off the timecourse experiment from the continuous ^{1}H photoNMR there is no distinct selectivity between the two types of alkenes. Their initial rates extrapolated from the first five minutes of reaction time (**Figure S2**) are comparable. However, of note the growth of product formation (**Figure S3**) indicates that fragmentation of the dioxazolidine **I** is more efficient at producing desired acyl fluoride product over the dioxazolidine formed with the methyl styrene substrate as growth of benzaldehyde is significantly slower.

but-3-en-1-ylbenzene vs 1-(1-fluorovinyl)-4-methylbenzene

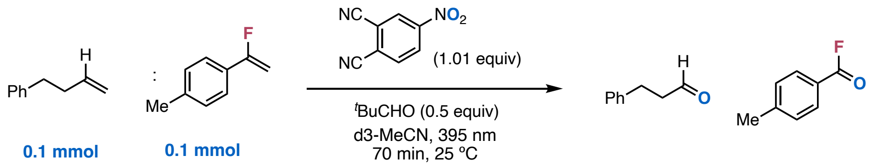

Reaction Setup according to general procedure B in a 1-dram vial. 4-nitrophtalonitrile (17.5 mg, 1.01 equiv, 0.101 mmol), but-3-en-1-ylbenzene (15 µL, 1 equiv, 0.100 mmol), 1-(1-fluorovinyl)-4-methylbenzene (13.6 mg, 1 equiv, 0.100 mmol), pivaldehyde (5.5 µL, 0.5 equiv, 50.0 µmol) in CD_3CN (1.0 mL, 0.1M). The mixture was then transferred to a N_2 purged PhotoNMR tube equipped with an inner cell for an optical insert (part number: NE:379-5-A). A coaxial cable, connected to an ultra-high power 395 nm LED was inserted and and sample was manually inserted to a Bruker NMR at 25 ºC. The reaction was monitored by 1H NMR

spectroscopy over the course of 70 minutes. For each 1H NMR spectrum 16 scans were acquired for a total data collection time of 105 seconds. The first scan was run before the light was turned on as time = 0.

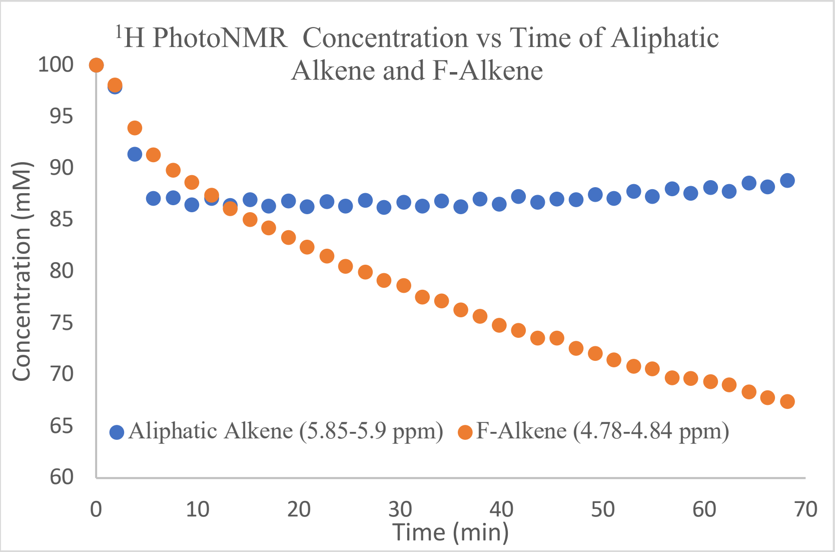

Figure S5: Concentration versus time graph of consumption of fluoroalkene and aliphatic alkene over the course of 70 minutes. Monitored from ^{1}H NMR

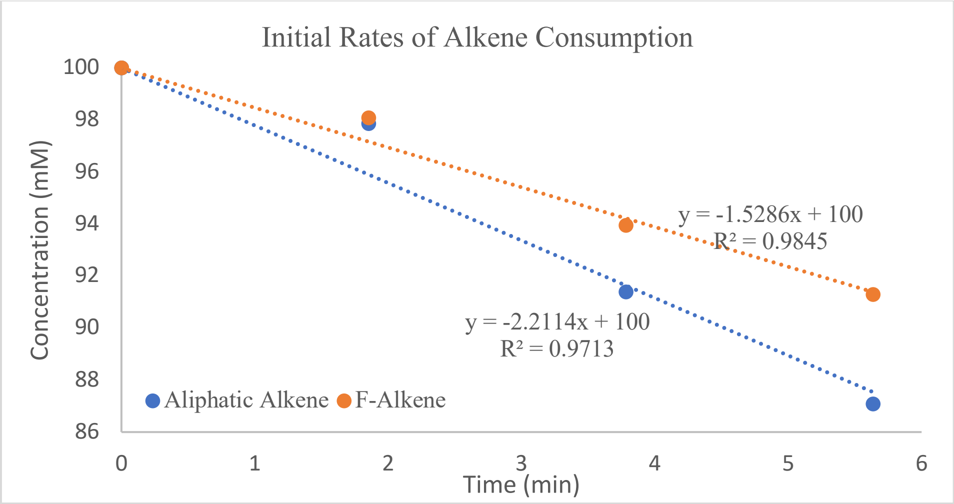

Figure S6: Initial rates from concentration versus time graph of the consumption of fluoroalkene and aliphatic alkene over the course of 5 min. Monitored by ^{1}H NMR

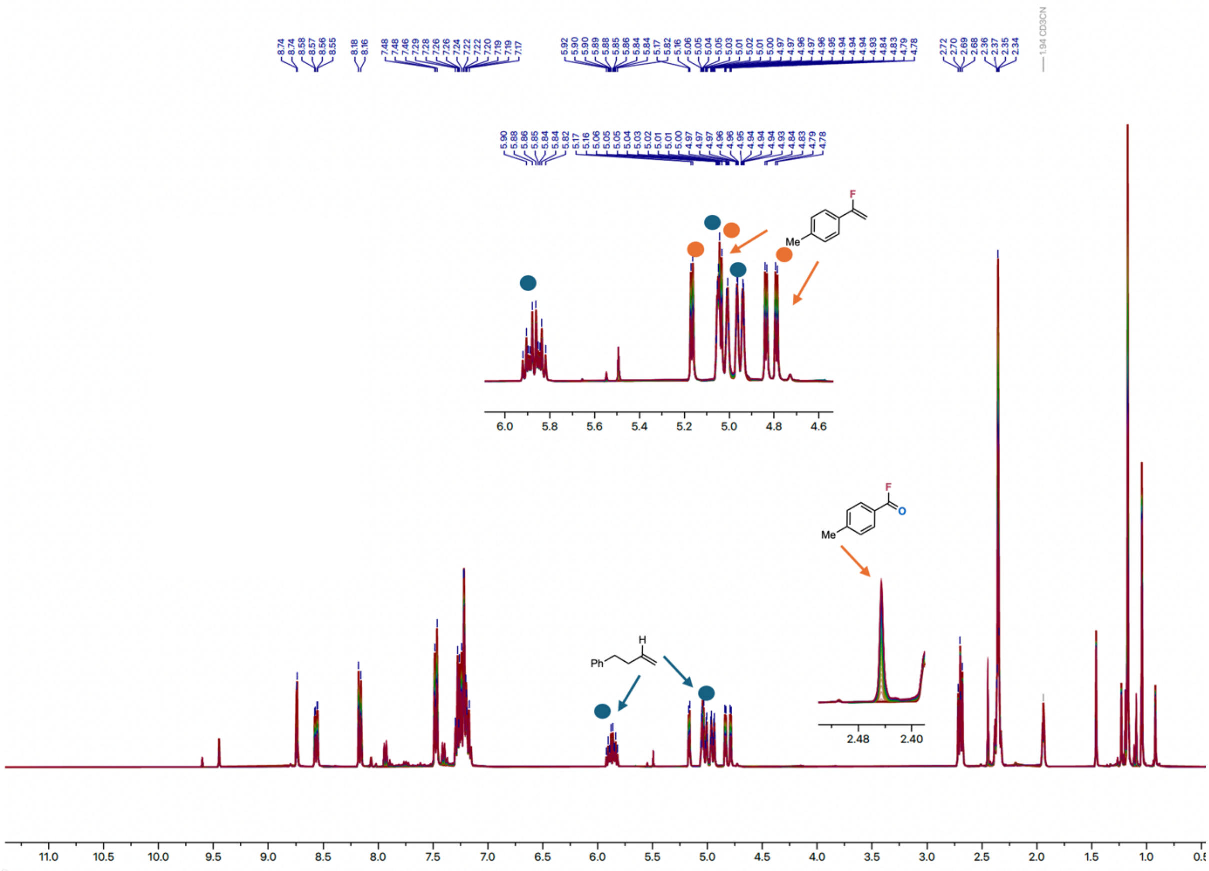

Figure S7: superimposed ^{1}H NMR Spectra over the course of 70 minutes with key peaks denoted that were used to monitor reaction progress.

Analysis: Based off the timecourse experiment from the continuous ^{1}H photoNMR there is preferential selectivity towards the styrenyl alkenyl fluoride over that of the aliphatic alkene (**Figure S5**). Their initial rates extrapolated from the first five minutes of reaction time (**Figure S6**) are comparable. However, after 5 minutes of irradiation the nitroarene no longer reacts with the aliphatic alkene.

Quantifying Oxidative Cleavage Products

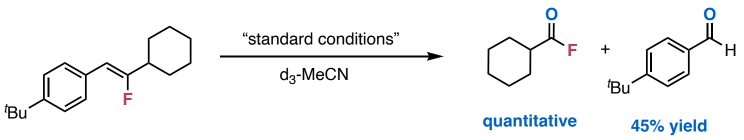

Prepared according to general procedure B (405 nm, 6 hours). 4-nitrophthalonitrile (52 mg, 0.300 mmol, 1.5 equiv.), (E/*Z*)-1-(*tert*-butyl)-4-(2-cyclohexyl-2-fluorovinyl)benzene (52.1 mg, 0.200 mmol) in CD_3CN (2.0 mL, 0.1 M). NMR standard dibromomethane (CH_2Br_2) (1 equiv.) was added and crude ^{1}H NMR yield was taken. Characterization of 4-(*tert*-butyl)benzaldehyde and cyclohexanecarbonyl fluoride matches literature reports.[31,33]

This study is in accordance with our proposed mechanism since close to 50% benzaldehyde was detected. We hypothesize this is the result of 0.5 equivalence of pivaldehyde reacting with carbonyl imine (IV) ultimately leading to the benzaldehyde product.

^{1}H NMR (500 MHz, $CDCl_3$) Yield of 4-(*tert*-butyl)benzaldehyde and cyclohexanecarbonyl fluoride

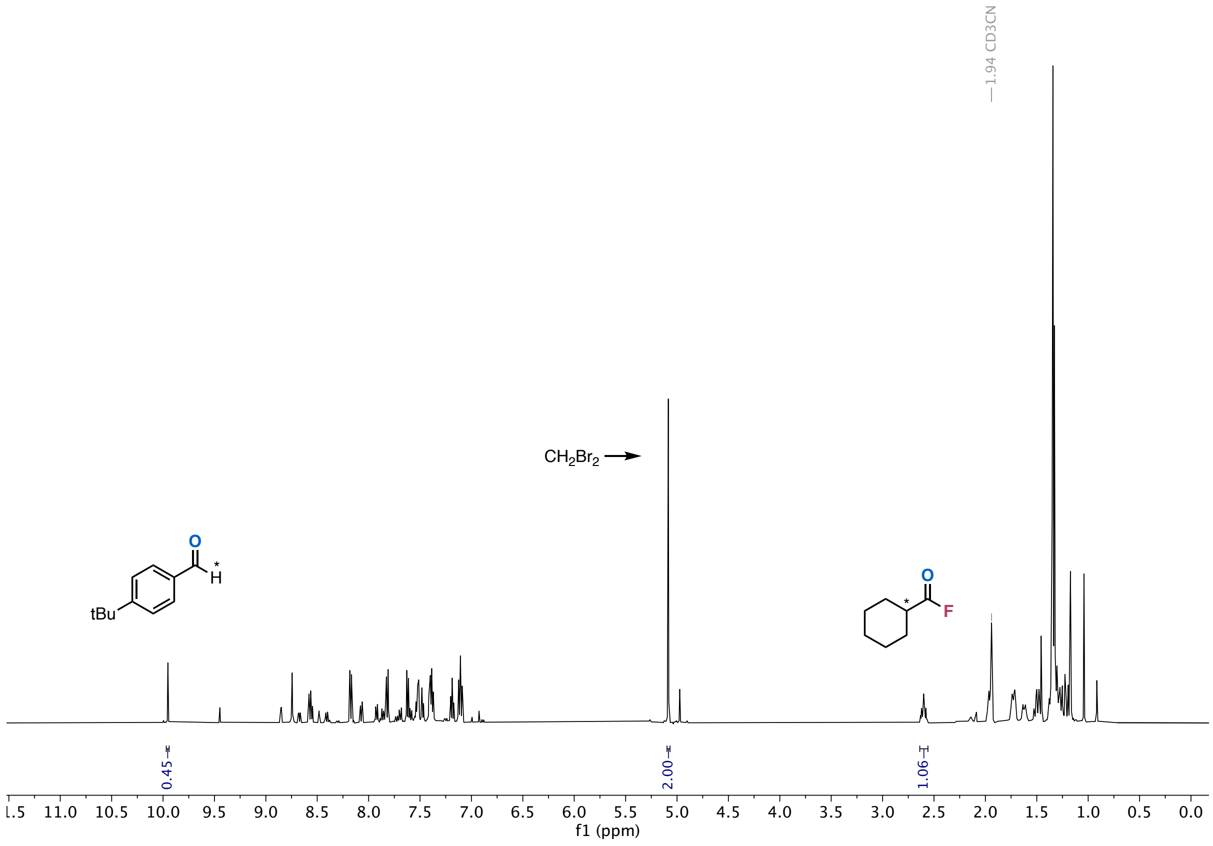

Peptide substrate synthesis and cleavage products

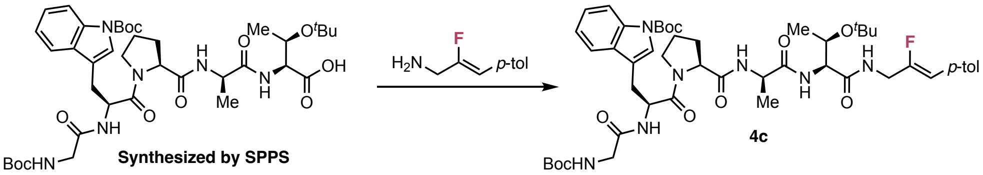

BocHN-Gly-Trp(Boc)-Pro-(*D*)-Ala-Thr(*t*Bu)-OH

Procedure was adapted from literature report.[34] *Adding 1st amino acid*: To a 12 mL filtration tube was added 2-chlorotrityl chloride resin (1.05 mmol, 953 mg, 1.05 equiv). A solution of Fmoc-(*t*Bu)-Thr-OH (1.0 equiv) in 6 mL DCM was added, followed by DIPEA (1.0 equiv) and shaken for 5 minutes. More DIPEA (1.5 equiv) was added, and the vessel was shaken for 1 h. Methanol (0.5 mL) was added, then shaken for 15 minutes. The suspension was filtered and washed with 6 mL DCM (3x). The remaining resin was washed with 6 mL DMF (2x), DCM (2x), then MeOH (3x). Resin was dried under vacuum.

Elongation: Then, 6 mL of 20% piperidine in DMF was added to the vessel and shaken for 10 minutes. The suspension was filtered and washed with 6 mL DMF (6x). Then, Fmoc-*D*-Ala (3.0 equiv) in DMF, HCTU (3.0 equiv) in DMF, and DIPEA (6.0 equiv) were added to the vessel and shaken for 2 h. The suspension was filtered and then washed with 6 mL DMF (6x). Three more iterations of deprotection/coupling were conducted, sequentially adding Fmoc-Pro-OH, Fmoc-(Boc)-Trp-OH, and (Boc)Gly-OH. Then the suspension was washed with DMF (6x) and DCM (6x).

Resin cleavage: To the resin with attached pentapeptide was added 6 mL DCM:HFIP (4:1) and shaken for 1 h, then filtered (2x). The remaining resin was rinsed with 6 mL DCM (2x), and the combined filtrates were concentrated and subjected to reverse-phase column chromatography with the following conditions: (20%-100% B in A), where B = 95:5 MeCN:H_2O with 0.1% TFA and A = H_2O with 0.1% TFA. The product was obtained as an off-white solid (655 mg, 83% yield).

^{1}H NMR (500 MHz, DMSO) (δ, ppm): 12.57 (s, 1H), 8.24 (d, *J* = 8.5 Hz, 1H), 8.02 (d, *J* = 7.9 Hz, 1H), 7.84 (d, *J* = 7.7 Hz, 1H), 7.69 (d, *J* = 7.7 Hz, 1H), 7.55 (s, 1H), 7.36 (d, *J* = 9.1 Hz, 1H), 7.34 – 7.28 (m, 1H), 7.26 (td, *J* = 7.5, 1.1 Hz, 1H), 6.84 (t, *J* = 6.1 Hz, 1H),4.86 (td, *J* = 8.6, 5.1 Hz, 1H), 4.36 (p, *J* = 7.2 Hz, 1H), 4.28 (dd, *J* = 8.7, 3.8 Hz, 1H), 4.23 (dd, *J* = 9.1, 2.5 Hz, 1H), 4.12 (qd, *J* = 6.2, 2.4 Hz, 1H), 3.72 – 3.65 (m, 1H), 3.64 – 3.36 (m, 3H) 3.19 (dd, *J* = 14.9, 5.1 Hz, 1H), 2.99 (ddd, *J* = 41.5, 14.2, 7.0 Hz, 1H), 2.83 (dd, *J* = 14.9, 8.7 Hz, 1H), 2.08 (s, 3H), 2.00 – 1.93 (m, 1H), 1.91 – 1.79 (m, 2H), 1.62 (s, 9H), 1.34 (s, 9H), 1.16 (d, *J* = 7.2 Hz, 3H), 1.09 (s, 9H), 1.02 (d, *J* = 6.2 Hz, 3H).

^{13}C{^{1}H} NMR (206 MHz, DMSO) (δ, ppm): 207.1, 172.7, 172.1, 171.6, 169.8, 169.4, 158.8, 158.6, 158.4, 158.2, 155.9, 149.2, 134.8, 130.5, 124.5, 124.2, 119.3, 116.7, 116.6, 115.1, 114.9, 83.7, 78.2, 73.4, 67.1, 60.6, 57.4, 55.1, 50.4, 48.4, 47.1, 43.1, 30.9, 29.4, 28.3, 28.3, 27.8, 26.7, 24.6, 20.5, 17.8.

HRMS (ESI TOF): calcd. For $C_{39}H_{59}N_6O_{11}$ $[M+H]^+$ = 787.4254, found 787.4242

HPLC:

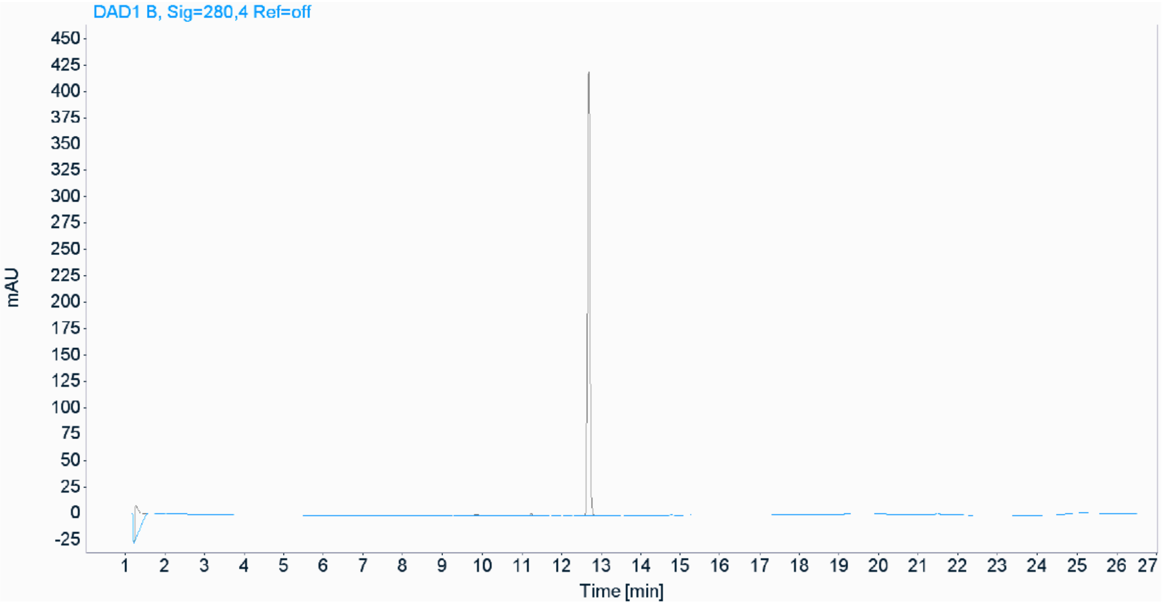

BocHN-Gly-Trp(Boc)-Pro-(*D*)-Ala-Thr(*t*Bu)-(Z)-3F-3(*p*-toly)prop-2-ene) (4c)

To a flame-dried 6-dram vial equipped with a stir bar was added peptide (1.0 equiv, 0.25 mmol, 197 mg) and amine (1.05 equiv, 263 mmol, 43.4 mg), followed by DMF/MeCN (1:4, 2.5 mL). The solution was cooled to 0 °C under nitrogen. HBTU (1.5 equiv, 0.375 mmol, 142 mg) and HOBt (1.5 equiv, 0.375 mmol, 50.7 mg) were added sequentially, followed by triethylamine (2.0 equiv, 0.5 mmol, 70 μL). The reaction mixture was allowed to warm to room temperature and stirred for 24 h. Upon completion, the reaction was partitioned between chloroform and 1 M $NaHSO_4$. The organic phase was washed sequentially with 1 M $NaHSO_4$, 0.5 M Na_2CO_3, and brine. The combined organic layers were dried over anhydrous Na_2SO_4, filtered, and concentrated under reduced pressure. The crude product was purified by normal-phase column chromatography (0–5% B in A), where B = MeOH with 0.1% formic acid and A = CH_2Cl_2 with 0.1% formic acid afforded the desired product as a white solid (113.7 mg, 49% yield). Note: ^{13}C data was obtained from Z:E mixture of **4c**.

^{1}H NMR (500 MHz, CD_3OD) (δ, ppm): 8.12 (d, *J* = 8.2 Hz, 1H), 7.65 (d, *J* = 8.2 Hz, 1H), 7.58 (s, 1H), 7.32 (t, *J* = 7.6 Hz, 1H), 7.29 – 7.24 (m, 3H), 7.03 (d, *J* = 8.0 Hz, 2H), 5.70 (d, *J* = 39.6 Hz, 1H), 5.04 – 4.98 (m, 1H), 4.55 (d, *J* = 6.7 Hz, 1H), 4.45 (q, *J* = 7.2 Hz, 1H), 4.38 (t, *J* = 6.6 Hz, 1H), 4.31 – 4.25 (m, 1H), 4.05 (dd, *J* = 13.6, 8.4 Hz, 2H), 3.77– 3.73 (m, 1H), 3.63 (q, *J* = 17.4, 16.5 Hz, 2H), 3.50 – 3.41 (m, 1H), 3.22 (dd, *J* = 14.9, 5.4 Hz, 1H), 3.03 (dd, *J* = 15.0, 8.7 Hz, 1H), 2.36 – 2.26 (m, 3H), 2.23 – 2.13 (m, 1H), 2.09 – 2.01 (m, 1H), 2.00 – 1.87 (m, 2H), 1.71 – 1.66 (m, 10H), 1.48 –1.41 (m, 12H), 1.31 – 1.28 (m, 4H), 1.22 (s, 9H), 1.18 (s, 2H).

^{13}C{^{1}H} NMR (201 MHz, CD_3OD)(δ, ppm): 175.0, 174.9, 174.2, 172.3, 172.2, 172.0, 158.3, 157.4, 156.0, 151.0, 138.2, 131.9, 131.4, 130.0, 129.6 (dd, $J_{C\text{–}F}$ = 9.9, 6.9 Hz), 125.8, 125.5 (d, $J_{C\text{–}F}$ = 29.8 Hz), 123.9, 123.7, 120.2, 119.9, 117.0, 116.2 (d, $J_{C\text{–}F}$ = 7.5 Hz), 108.3, 85.1, 84.9, 80.7, 75.7, 75.4, 68.1, 68.0, 61.9 (d, $J_{C\text{–}F}$ = 23.7 Hz), 60.5 (dd, $J_{C\text{–}F}$ = 16.4, 5.6 Hz), 53.5, 52.4, 51.4, 50.6, 48.0, 44.5, 41.5 (dd, $J_{C\text{–}F}$ = 33.5, 23.2 Hz), 32.1, 30.2, 29.2, 28.8, 28.7, 28.66, 28.5, 28.4, 28.0, 26.0, 23.0, 21.2, 20.8, 20.3, 17.9, 17.7.

^{19}F{^{1}H} NMR (784 MHz, CD_3OD)(δ, ppm): -111.66 (ddt, *J* = 52.5, 39.7, 13.2 Hz).

HRMS (ESI TOF): calcd for $C_{49}H_{69}N_7O_{10}F$ $[M+H]^+$ = 934.5080, found 934.5090

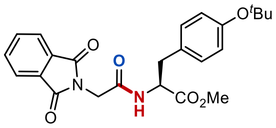

methyl (S)-3-(4-(tert-butoxy)phenyl)-2-(2-(1,3-dioxoisoindolin-2-yl)acetamido)propanoate (5a)

Prepared according to general procedure B (405 nm, 6 hours). 4-nitrophthalonitrile (52.3 mg, 0.302 mmol, 1.5 equiv.), (*Z*)-2-(2-fluoro-3-(*p*-tolyl)allyl)isoindoline-1,3-dione (59.5 mg, 0.201 mmol), methyl (*S*)-2-amino-3-(4-(tert-butoxy)phenyl)propanoate (76.0 mg, 0.302 mmol, 1.5 equiv.) in acetonitrile (2.0 mL, 0.1 M). Two reactions were run simultaneously were combined for isolation. The title compound was isolated via flash column chromatography (gradient 0 – 7% EtOAc/hexanes) as an orange solid (48 mg, 55% yield).

^{1}H NMR (600 MHz, $CDCl_3$) (δ, ppm): 7.88 (dd, *J* = 5.4, 3.1 Hz, 2H), 7.77 (dd, *J* = 5.4, 3.0 Hz, 2H), 7.00 – 6.97 (m, 2H), 6.87 – 6.84 (m, 2H), 6.23 (d, *J* = 7.7 Hz, 1H), 4.79 (dt, *J* = 7.7, 5.7 Hz, 1H), 4.35 – 4.26 (m, 2H), 3.70 (s, 3H), 3.07 (qd, *J* = 13.9, 5.7 Hz, 2H), 1.30 (s, 9H).
^{13}C{^{1}H} NMR (151 MHz, $CDCl_3$) (δ, ppm): 171.9, 168.0, 165.9, 155.1, 134.7, 132.4, 130.8, 130.2, 124.5, 123.9, 78.6, 53.9, 52.7, 41.1, 37.4, 29.0.
HRMS (ESI TOF): calcd. for $C_{24}H_{26}N_2O_6$ $[M+H]^+$ 439.1869, found 439.1870

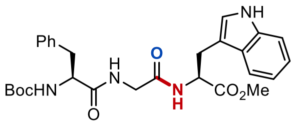

methyl (*tert*-butoxycarbonyl)-*L*-phenylalanylglycyl-*L*-tryptophanate (5b)

Prepared according to general procedure B (405 nm, 6 hours). 4-nitrophthalonitrile (27.9 mg, 0.161 mmol, 1.5 equiv.), tert-butyl (S,Z)-(1-((2-fluoro-3-(p-tolyl)allyl)amino)-1-oxo-3-phenylpropan-2-yl)carbamate (44.3 mg, 0.107 mmol) in acetonitrile (1.1 mL, 0.1 M). methyl tryptophanate (35.2 mg, 0.161 mmol, 1.5 equiv.) was added after starting material consumption. NMR yield = 60%. The title compound was isolated via flash column chromatography (gradient 0 – 50% EtOAc/hexanes) as a yellow solid (30.1 mg, 54% yield).

^{1}H NMR (500 MHz, $CDCl_3$) (δ, ppm): 8.74 (s, 1H), 7.49 (d, *J* = 7.9 Hz, 1H), 7.28 (ddd, *J* = 25.3, 14.9, 7.5 Hz, 4H), 7.13 (tt, *J* = 14.9, 7.4 Hz, 4H), 6.99 (s, 1H), 6.95 (d, *J* = 7.7 Hz, 1H), 6.81 (d, *J* = 6.6 Hz, 1H), 5.16 (d, *J* = 8.0 Hz, 1H), 4.85 (q, *J* = 6.4 Hz, 1H), 4.37 (q, *J* = 7.6 Hz, 1H), 3.84 (dd, *J* = 16.9, 5.8 Hz, 1H), 3.67 (s, 3H), 3.64 (s, 1H), 3.37 – 3.26 (m, 2H), 3.07 (dd, *J* = 14.0, 5.9 Hz, 1H), 2.89 (dd, *J* = 14.2, 8.1 Hz, 1H), 1.40 (s, 9H).
^{13}C{^{1}H} NMR (126 MHz, $CDCl_3$) (δ, ppm): 172.3, 172.1, 168.6, 155.8, 136.6, 136.3, 129.3, 128.8, 127.5, 127.1, 123.6, 122.2, 119.6, 118.5, 111.6, 109.5, 80.6, 55.9, 52.7, 52.6, 43.1, 38.5, 28.4, 27.4.
HRMS (ESI TOF): calcd. for $C_{28}H_{35}N_4O_6$ $[M+H]^+$ 523.2557, found 523.2555

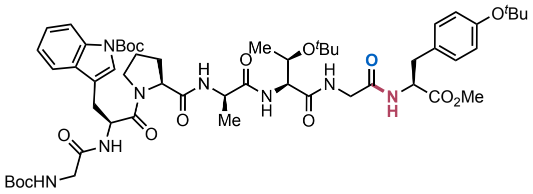

BocHN-Gly-Trp(Boc)-Pro-*D*-Ala-Thr(*t*Bu)-Gly-Tyr(*t*Bu)-OMe (5c)

Prepared according to general procedure B (405 nm, 4 hours). 4-nitrophthalonitrile (22.9 mg, 0.132 mmol, 1.5 equiv.), pivaldehyde (4.78 µL, 0.044 mmol, 0.5 equiv.), tert-butyl (S,Z)-(1-((2-fluoro-3-(p-tolyl)allyl)amino)-1-oxo-3-phenylpropan-2-yl)carbamate (93.4 mg, 0.88 mmol (88%wt), 1.0 equiv.) in acetonitrile (1.0 mL, 0.1 M). *O-tert*-butyl-L-tyrosine methyl ester (33.2 mg, 0.132 mmol, 1.5 equiv.) was added after starting material consumption. The crude reaction mixture solvent was removed in vacuo then resuspended in CH_2Cl_2. The crude solution was loaded to a silica plug and flushed with 100% ether. The silica plug was then flushed with 10% MeOH/CH_2Cl_2 which was then collected, and the solvent was removed in vacuo. Followed by reverse-phase column chromatography with the following conditions: (10%-100% B in A), where B = 95:5 MeCN:H_2O with 0.1% TFA and A = H_2O with 0.1% TFA. Fractions were extracted in CH_2Cl_2, dried over Na_2SO_4. The title compound was isolated via pTLC (5:94:1 MeOH/CH_2Cl_2/formic acid) as a yellow solid (46.3 mg, 38% yield based on purity of 78% determined by quantitative ^{1}H NMR assay with 1.14 equiv. pivaldehyde as external standard). A portion of the mixture was further purified on pHPLC to obtain cleaner spectra.

^{1}H NMR (800 MHz, CD_3OD) (δ, ppm): 8.07 (d, *J* = 8.1 Hz, 2H), 7.82 (dd, *J* = 15.3, 7.5 Hz, 2H), 7.63 (d, *J* = 7.7 Hz, 1H), 7.59 – 7.53 (m, 2H), 7.32 – 7.26 (m, 2H), 7.25 – 7.21 (m, 2H), 7.06 (dd, *J* = 10.8, 8.3 Hz, 2H), 6.87 (dd, *J* = 8.2, 5.9 Hz, 2H), 4.99 (dd, *J* = 8.2, 5.7 Hz, 2H), 4.71 (t, *J* = 7.7 Hz, 1H), 4.60 (ddd, *J* = 8.9, 6.4, 2.3 Hz, 1H), 4.52 – 4.46 (m, 2H), 4.35 (dp, *J* = 5.9, 2.0 Hz, 2H), 4.26 (q, *J* = 2.6 Hz, 1H), 4.18 (dd, *J* = 6.4, 2.7 Hz, 1H), 4.14 – 4.08 (m, 2H), 3.94 – 3.85 (m, 4H), 3.82 (d, *J* = 5.9 Hz, 1H), 3.78 – 3.72 (m, 2H), 3.60 (d, *J* = 11.0 Hz, 3H), 3.54 – 3.49 (m, 2H), 3.21 (dd, *J* = 15.0, 5.7 Hz, 2H), 3.11 (dd, *J* = 7.7, 3.8 Hz, 1H), 3.03 (ddd, *J* = 24.8, 14.4, 7.4 Hz, 3H), 2.96 (ddd, *J* = 13.9, 11.8, 7.9 Hz, 2H), 2.19 – 2.14 (m, 1H), 2.01 (dt, *J* = 12.5, 6.5 Hz, 2H), 1.95 (dq, *J* = 12.4, 6.1 Hz, 2H), 1.90 (dq, *J* = 12.4, 6.5 Hz, 2H), 1.66 (d, *J* = 1.4 Hz, 4H), 1.43 (s, 1H), 1.39 (s, 2H), 1.36 (t, *J* = 7.5 Hz, 3H), 1.31 (d, *J* = 7.1 Hz, 2H), 1.29 (s, 1H), 1.28 (s, 6H), 1.18 (s, 5H), 1.12 (s, 1H), 1.10 – 1.07 (m, 2H).
^{13}C{^{1}H} NMR (201 MHz, CD_3OD) δ 173.5, 172.9, 171.8, 171.0, 169.7, 156.9, 154.1, 149.7, 131.6, 130.5, 129.4, 124.4, 124.1, 123.9, 122.4, 118.8, 118.5, 115.5, 114.8, 83.5, 79.3, 78.1, 74.6, 66.7, 60.7, 58.9, 54.1, 54.1, 51.3, 51.1, 48.8, 43.1, 42.1, 36.5, 29.0, 27.8, 27.3, 27.1, 27.0, 26.7, 24.7, 21.7, 18.3, 16.3
HRMS (ESI TOF): calcd. for $C_{55}H_{81}N_8O_{14}$ $[M+H]^+$ 1077.5872, found 1077.5875
LCMS of purified peptide

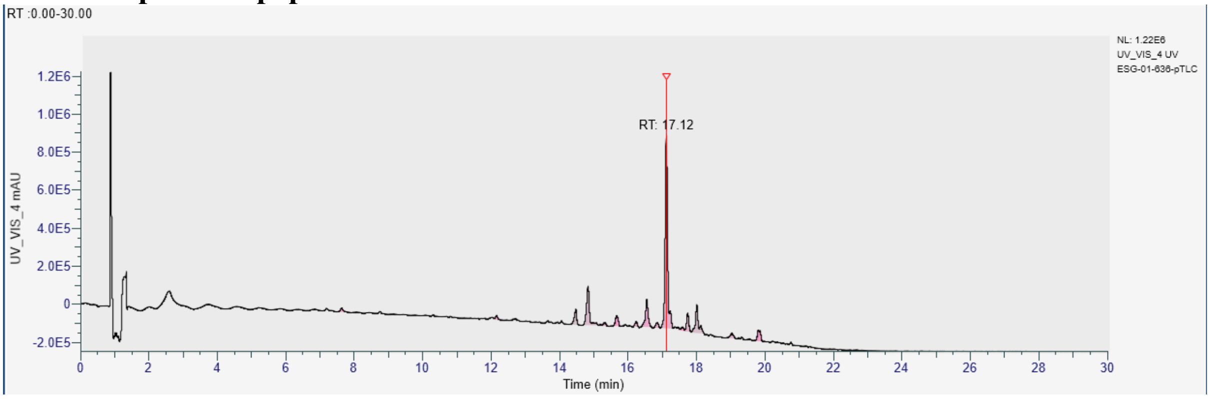

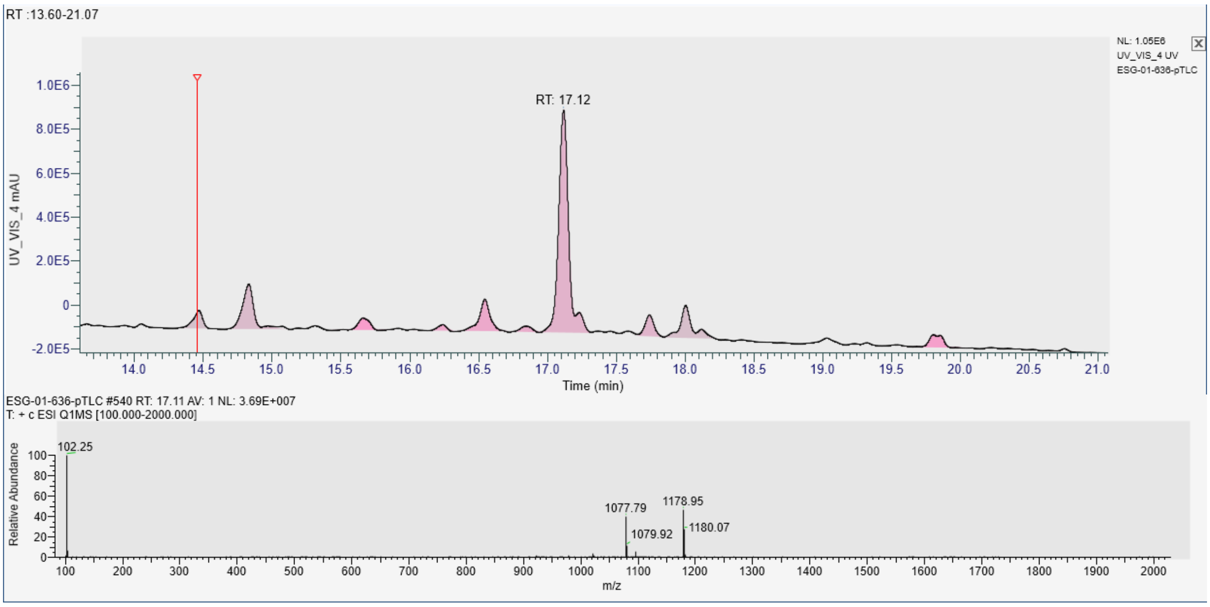

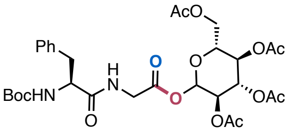

(2*R*,3*R*,4*S*,5*R*)-2-(acetoxymethyl)-6-(((*tert*-butoxycarbonyl)-*L*-phenylalanylglycyl)oxy)tetrahydro-2*H*-pyran-3,4,5-triyl triacetate (5d)

Prepared according to general procedure B (405 nm, 6 hours). 4-nitrophthalonitrile (26.0 mg, 0.150 mmol, 1.5 equiv.), tert-butyl (S,Z)-(1-((2-fluoro-3-(p-tolyl)allyl)amino)-1-oxo-3-phenylpropan-2-yl)carbamate (41.3 mg, 0.100 mmol) in acetonitrile (1.0 mL, 0.1 M). 2,3,4,6-tetra-O-acetyl-D-glucopyranose (34..8 mg, 0.100 mmol, 1.0 equiv.), TEA (27.9 µL, 0.200 mmol, 2.0 equiv.) and DMAP (6.11 mg, 0.050 mmol, 0.5 equiv.) was added after starting material consumption. The title compound is reported as 51% ^{1}H NMR yield (1.0 equiv. of CH_2Br_2 was used as an external standard).

Selected signals from crude NMR.

^{1}H NMR (500 MHz, $CDCl_3$) (δ, ppm): 6.64 (d, J = 6.3 Hz, 1H), 5.54 (t, J = 9.8 Hz, 1H), 5.43 (d, J = 3.5 Hz, 1H), 5.07 (t, J = 9.8 Hz, 1H), 4.89 (dd, J = 10.2, 3.7 Hz, 1H), 4.27 (ddd, J = 10.1, 4.3, 2.3 Hz, 1H), 4.22 (dd, J = 12.2, 4.3 Hz, 1H), 4.09 (dd, J = 12.2, 2.2 Hz, 1H).
HRMS (ESI TOF): calcd. for $C_{30}H_{41}N_2O_{14}$ $[M+H]^+$ = 653.2553, found 653.2546

Synthesis of substrate 5d standard

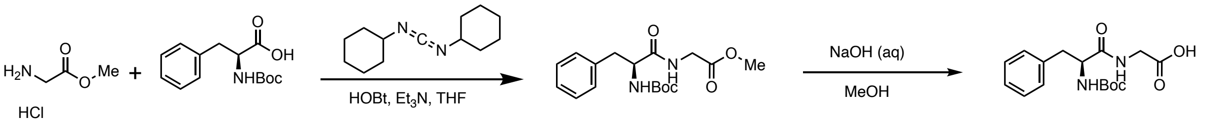

BocNH-Phe-Gly-O*t*Bu

Dipeptide was made through the following scheme using literature reported methods, all analytical data matched previously reported data.[35-36]

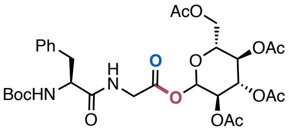

(2*R*,3*R*,4*S*,5*R*)-2-(acetoxymethyl)-6-(((*tert*-butoxycarbonyl)-*L* phenylalanylglycyl)oxy)tetrahydro-2*H*-pyran-3,4,5-triyl triacetate

To a flame dried 100 mL round bottom flask equipped with stir bar was added (tert-butoxycarbonyl)-L-phenylalanylglycine (150 mg, 0.46 mmol, 1 equiv.), triethylamine (130 µL, 0.93 mmol, 2 equiv.), and 30 mL of dry THF. The mixture was stirred for 5 minutes, and then 1-Hydroxy-1H-benzotriazole (126 mg, 0.93 mmol, 2 equiv.) and 2,3,4,6-Tetra-O-acetyl-a-D-glucopyranose (324 mg, 0.93 mmol, 2 equiv.) were added. The mixture was cooled to 0 °C in an ice bath, and then Dicyclohexylcarbodiimide (96.0 mg, 0.46 mmol, 1 equiv.) dissolved in 5 mL of dry THF was added in one portion. The mixture was stirred for 15 minutes at 0 °C and then was allowed to warm to room temperature and allowed to stir overnight. The solids were filtered off and the solvent was removed under reduced pressure. The crude oil was dissolved in 15 mL of EtOAc and placed in the freezer for 30 minutes. The solids were filtered off and then mixture was transferred to a separatory funnel and washed 2x with saturated sodium bicarbonate solution. The combined aqueous layers were extracted 2x with DCM (10 mL) and the combined organic layer was dried over sodium sulfate. The solvent was removed and the product was isolated by preparative TLC (98:2 $CHCl_3$/MeOH) to afford the product was a white solid (22 mg, 7% yield)

^{1}H NMR (500 MHz, $CDCl_3$) (δ, ppm): 7.30 (t, *J* = 7.3 Hz, 2H), 7.24 – 7.11 (m, 3H), 6.53 (s, 1H), 5.74 (d, *J* = 8.3 Hz, 1H), 5.26 (t, *J* = 9.4 Hz, 1H), 5.18 – 5.07 (m, 2H), 4.39 (d, *J* = 9.8 Hz, 1H), 4.29 (dd, *J* = 12.5, 4.5 Hz, 1H), 4.16 – 4.04 (m, 3H), 3.98 – 3.89 (m, 1H), 3.85 (ddd, *J* = 10.1, 4.6, 2.2 Hz, 1H), 3.15 (dd, *J* = 14.0, 6.6 Hz, 1H), 2.98 (dd, *J* = 14.0, 7.5 Hz, 1H), 2.13 – 1.97 (m, 12H), 1.41 (s, 9H).
^{13}C{^{1}H} NMR (126 MHz, $CDCl_3$) 171.9, 170.7, 170.2, 169.8, 169.5, 167.7, 136.9, 129.5, 128.8, 127.0, 92.4, 73.0, 72.7, 70.2, 67.8, 61.5, 60.5, 41.4, 28.4, 21.2, 20.8, 20.8, 20.7, 14.3.
HRMS (ESI TOF): calcd. for $C_{30}H_{41}N_2O_{14}$ $[M+H]^+$ = 653.2558, found 653.2560

NMR spectra of substrates

^{1}H NMR (500 MHz, CDCl$_3$) of 1-bromo-3-(1-fluorovinyl)benzene (1h)

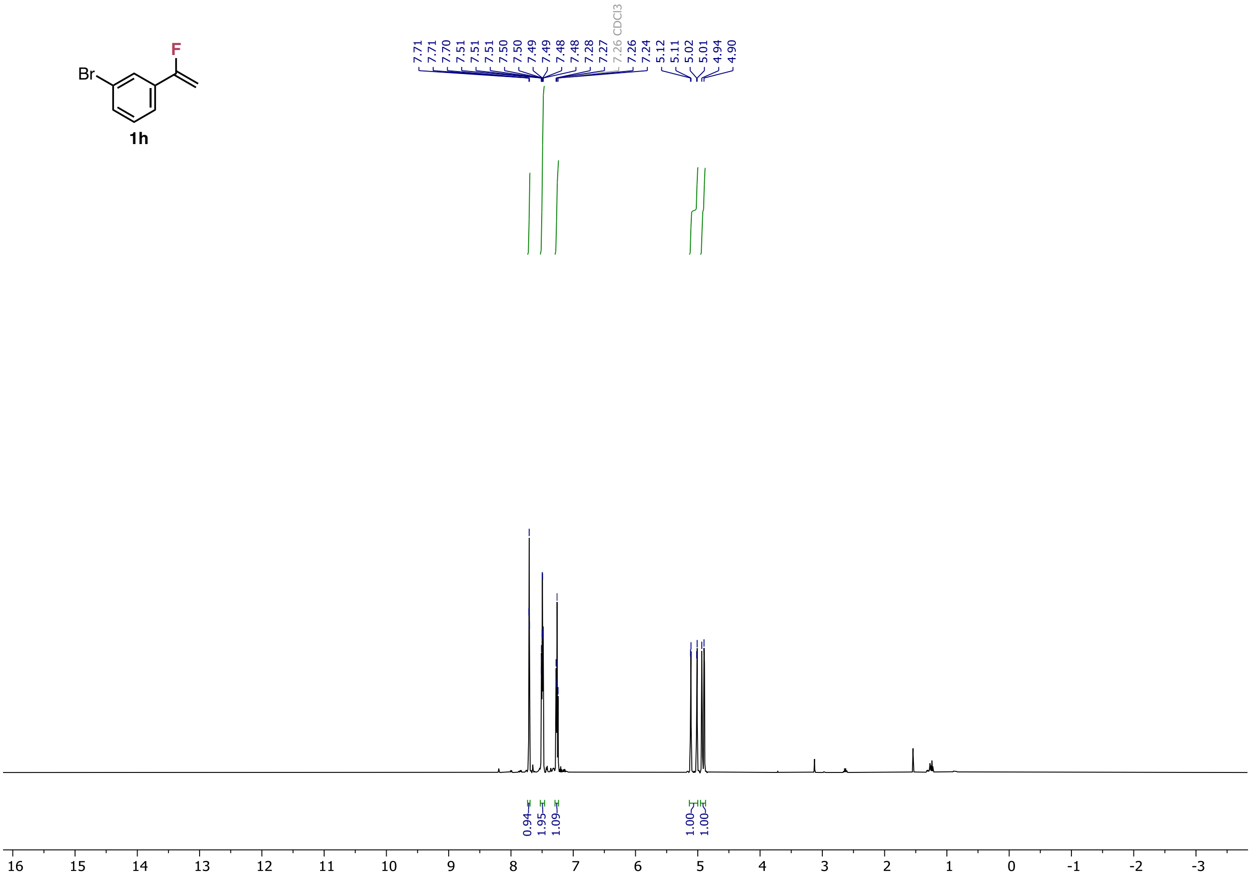

^{13}C{^{19}F} NMR (126 MHz, CDCl$_3$) of 1-bromo-3-(1-fluorovinyl)benzene (1h)

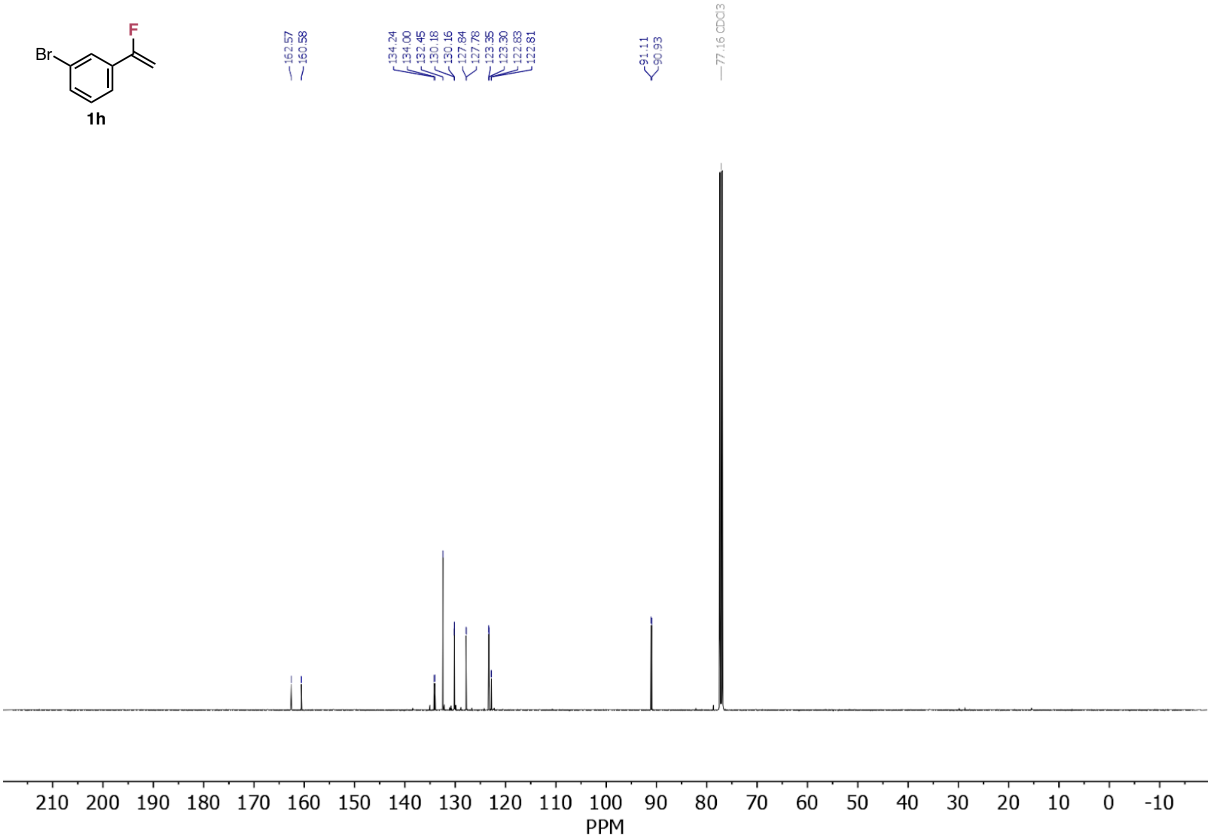

$^{19}F\{^{1}H\}$ NMR (471 MHz, $CDCl_3$) of 1-bromo-3-(1-fluorovinyl)benzene (1h)

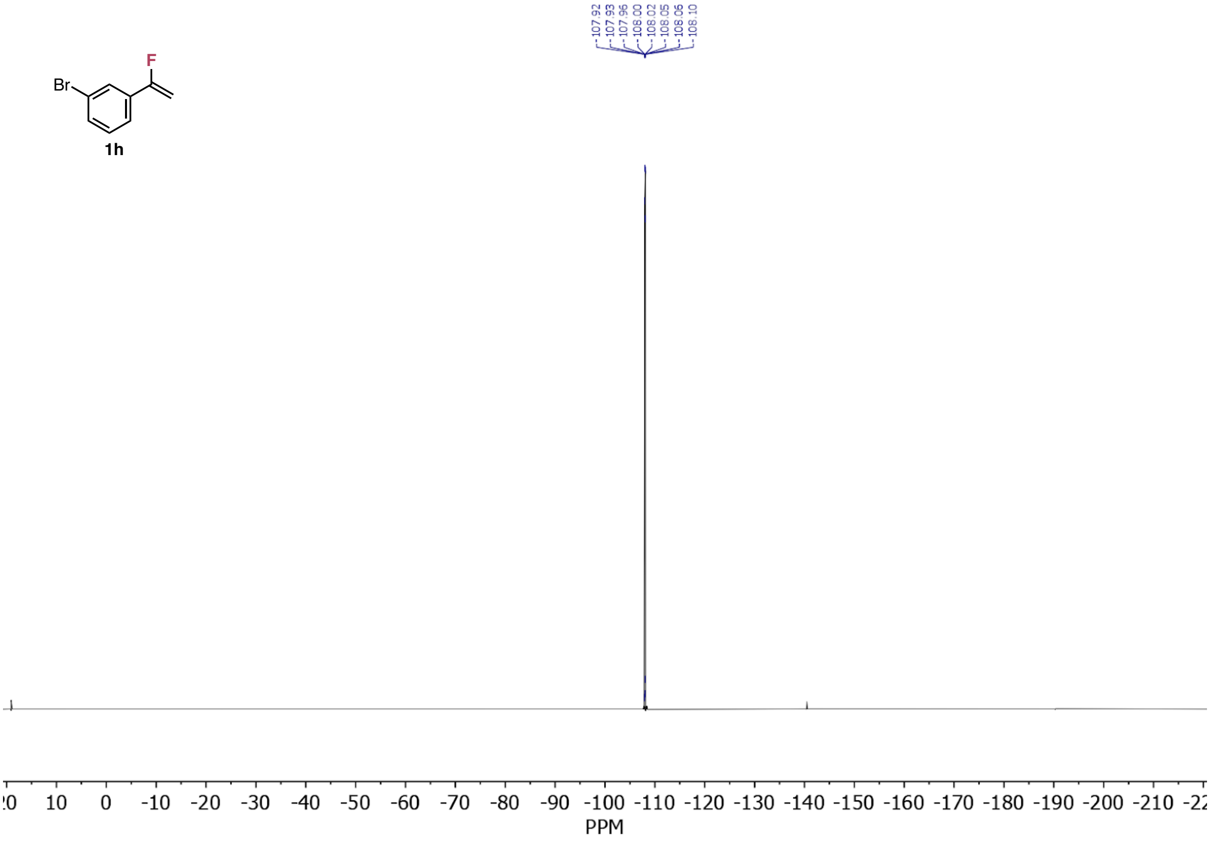

^{1}H NMR (500 MHz, $CDCl_3$) of methyl (E)-3-(1-fluoro-2-phenylprop-1-en-1-yl)benzoate (1l)

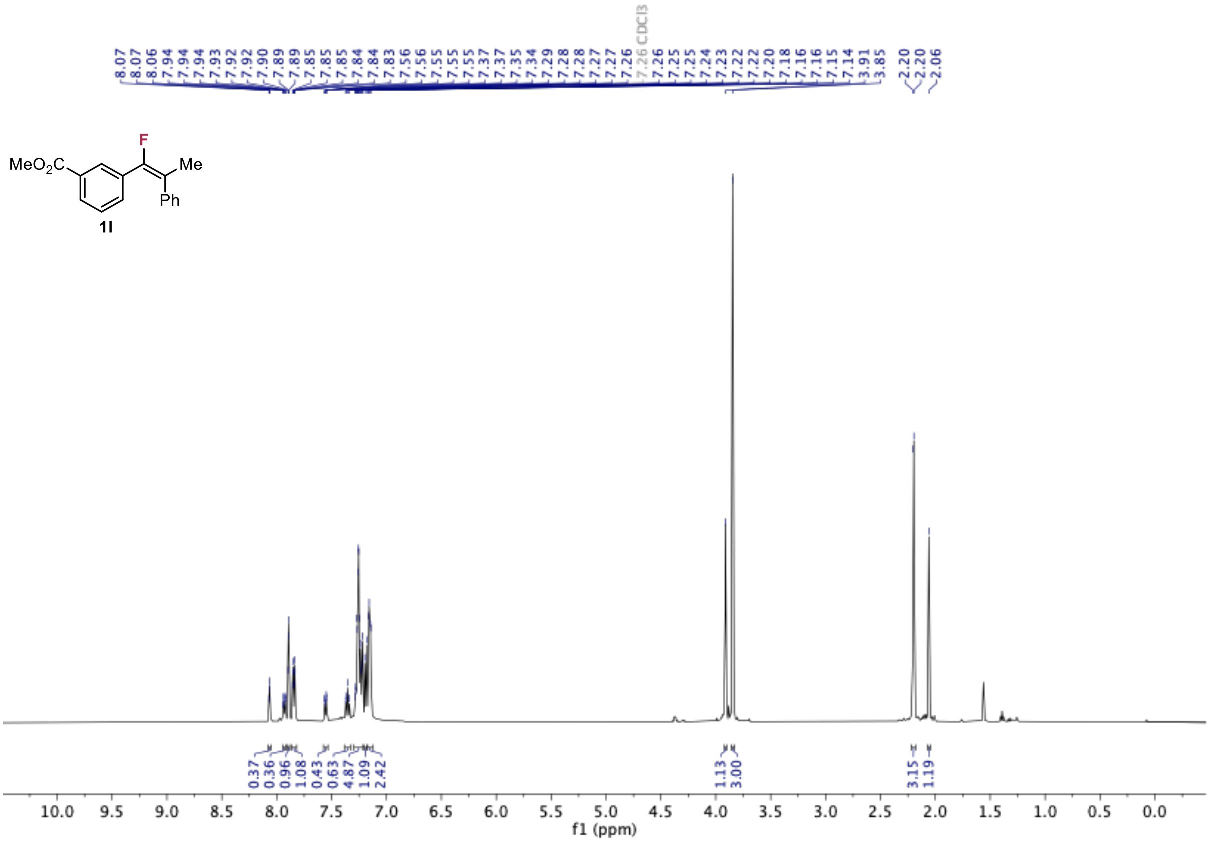

$^{13}C\{^{1}H\}$ NMR (126 MHz, $CDCl_3$) of methyl (E)-3-(1-fluoro-2-phenylprop-1-en-1-yl)benzoate (1l)

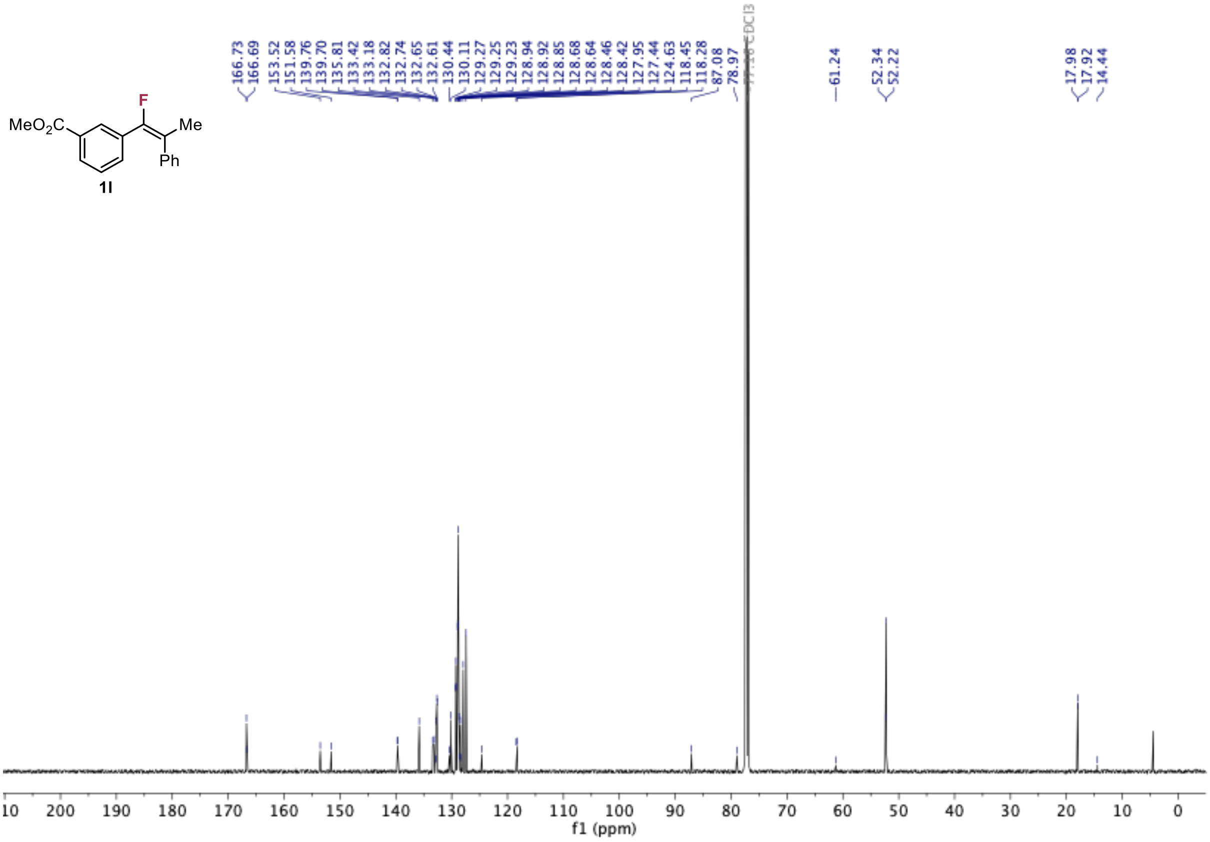

^{19}F NMR (471 MHz, $CDCl_3$) of methyl (E)-3-(1-fluoro-2-phenylprop-1-en-1-yl)benzoate (1l)

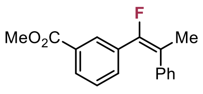

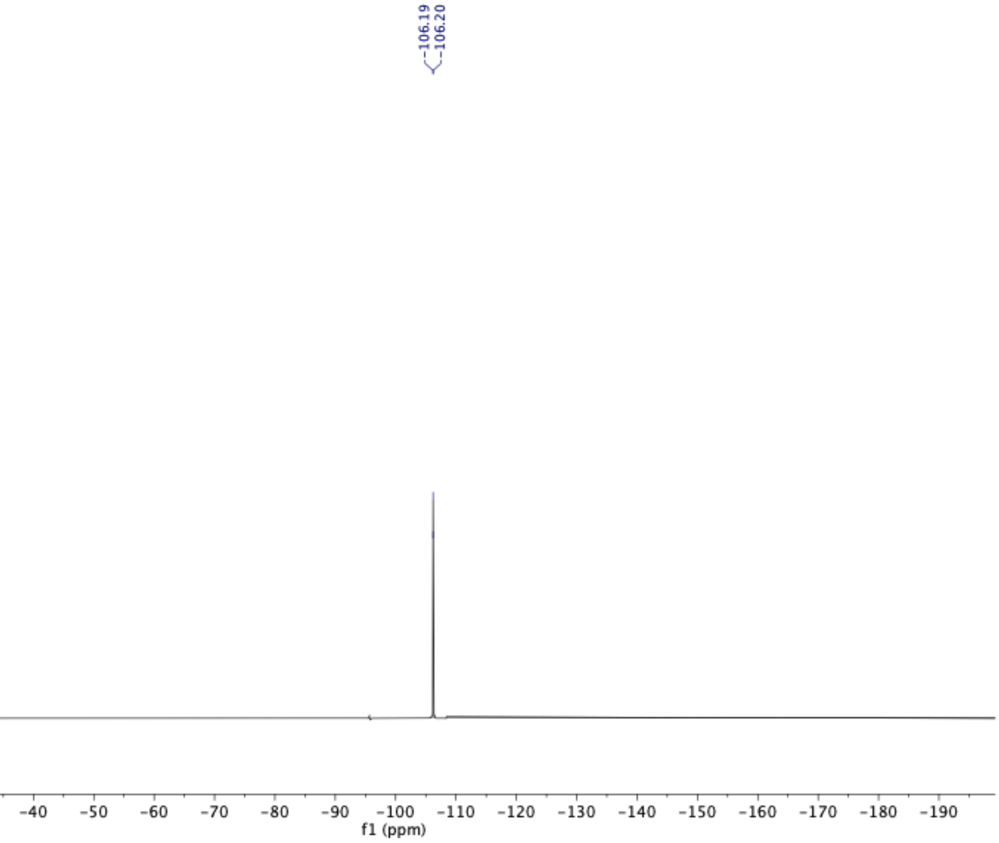

^{1}H NMR (500 MHz, CD$_3$OD) of (E)-3-(1-fluoro-2-phenylprop-1-en-1-yl)benzoic acid (1k)

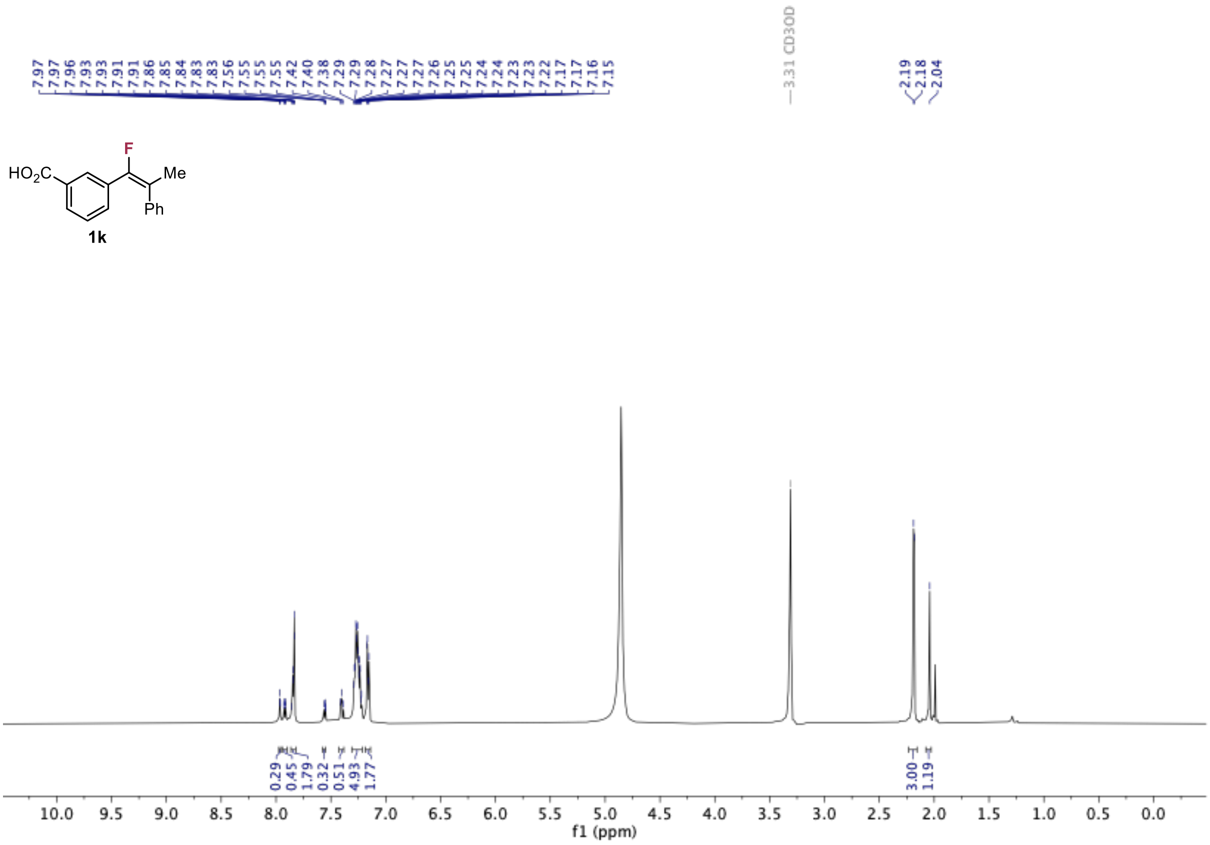

^{13}C{^{1}H} NMR (126 MHz CD$_3$OD) of (E)-3-(1-fluoro-2-phenylprop-1-en-1-yl)benzoic acid (1k)

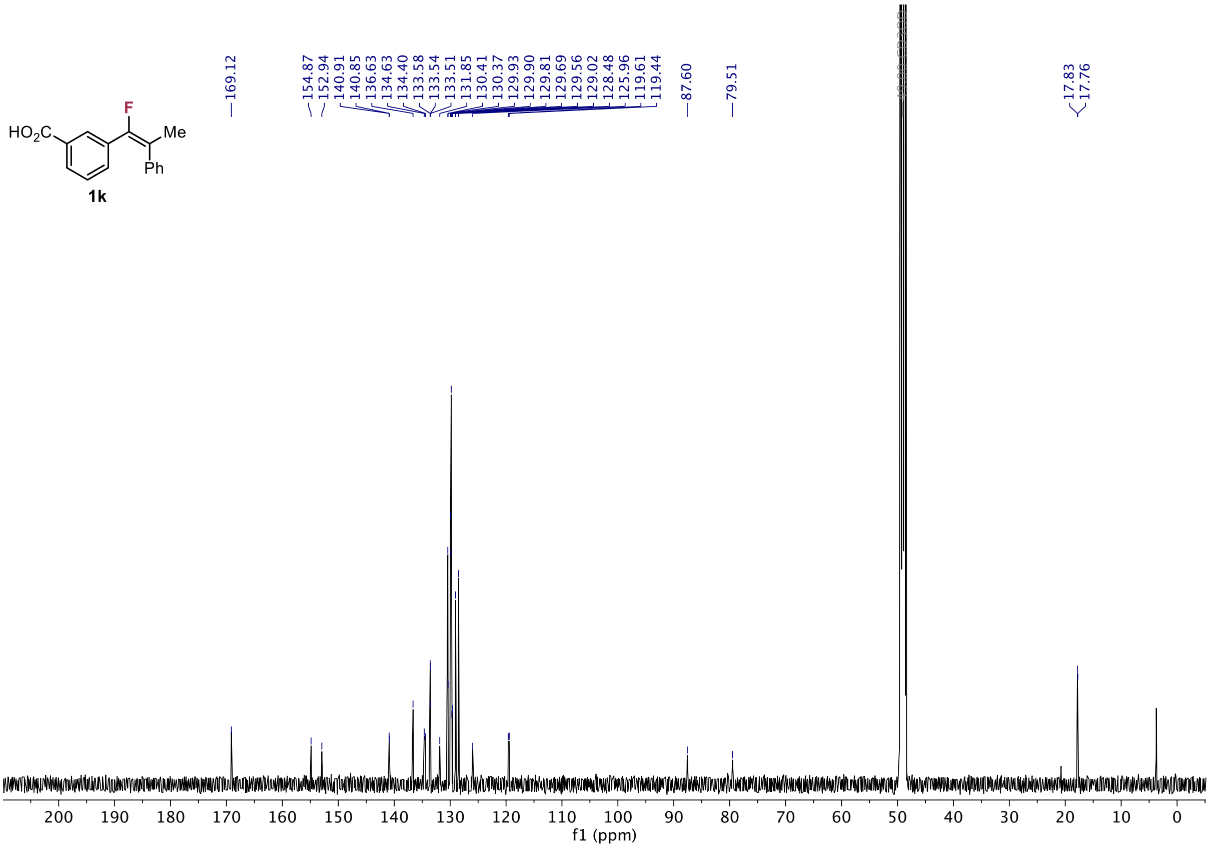

^{19}F NMR (471 MHz CD3OD) of (E)-3-(1-fluoro-2-phenylprop-1-en-1-yl)benzoic acid (1k)

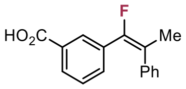

1k

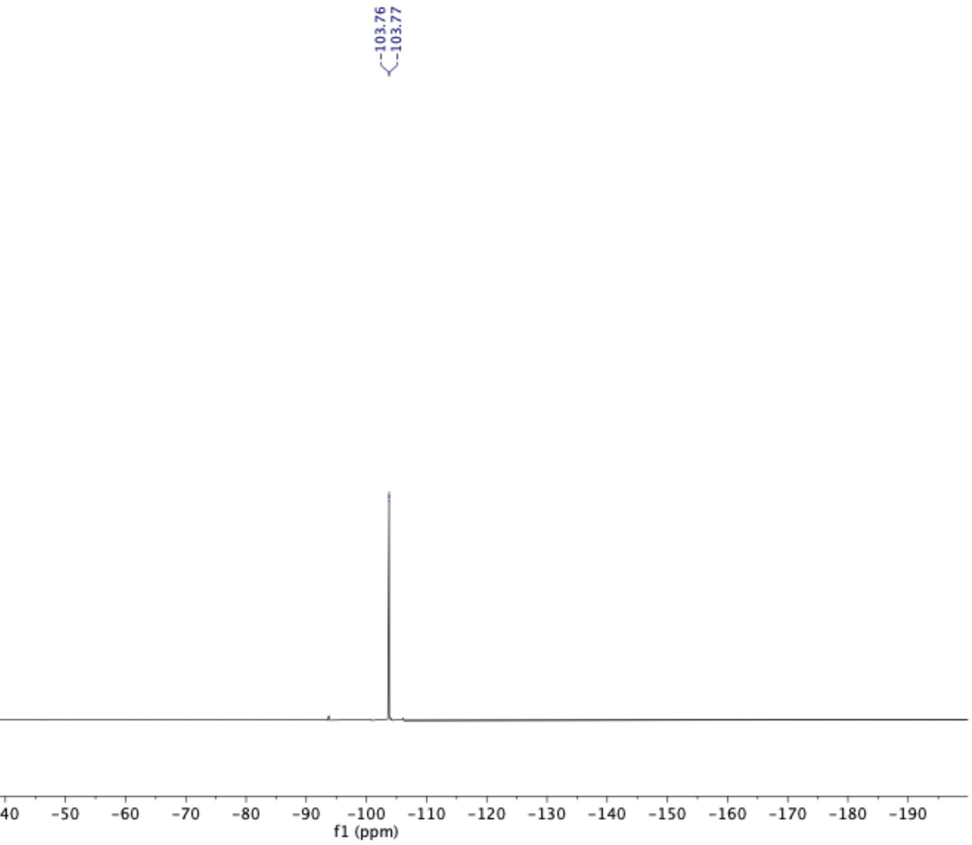

^{1}H NMR (500 MHz, $CDCl_3$) of (E)-(3-(1-fluoro-2-phenylprop-1-en-1-yl)phenyl)methanol (1m)

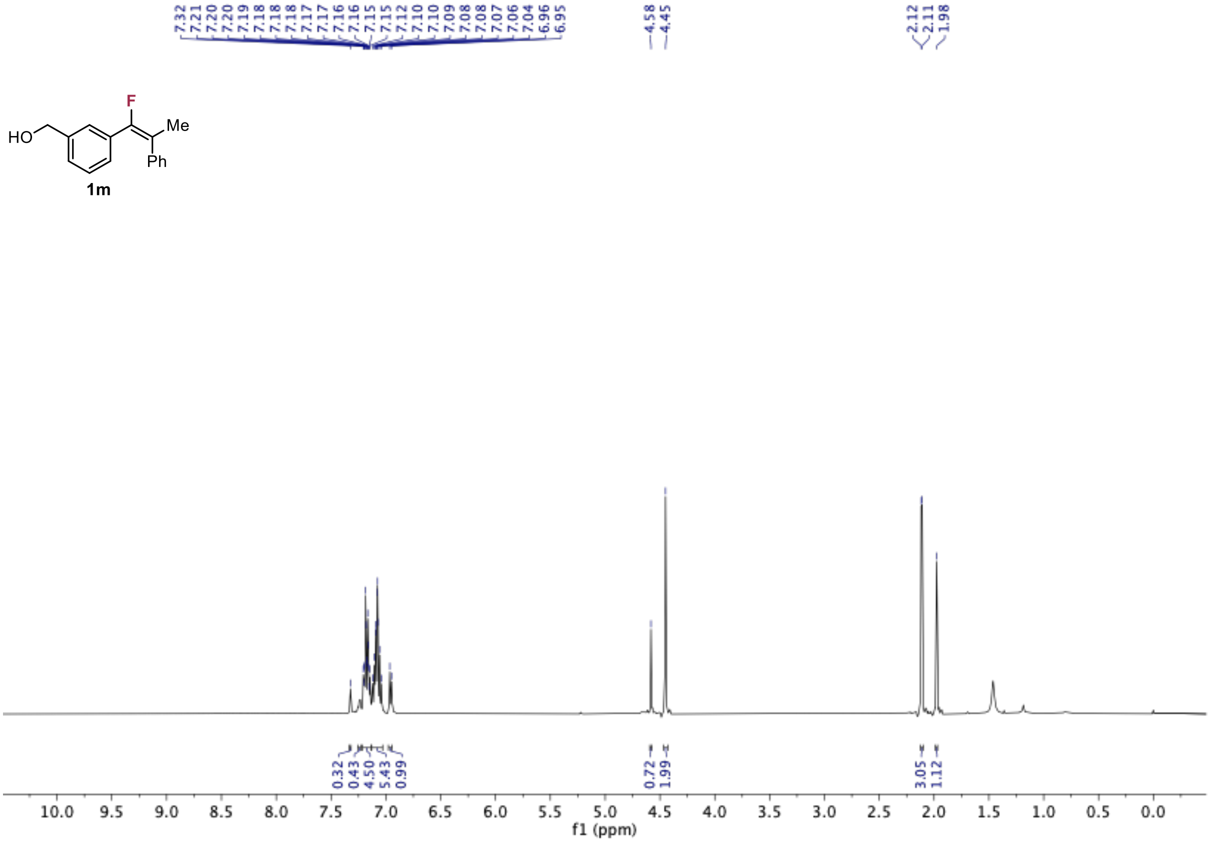

^{13}C{^{1}H} NMR (126 MHz, $CDCl_3$) of (E)-(3-(1-fluoro-2-phenylprop-1-en-1-yl)phenyl)methanol (1m)

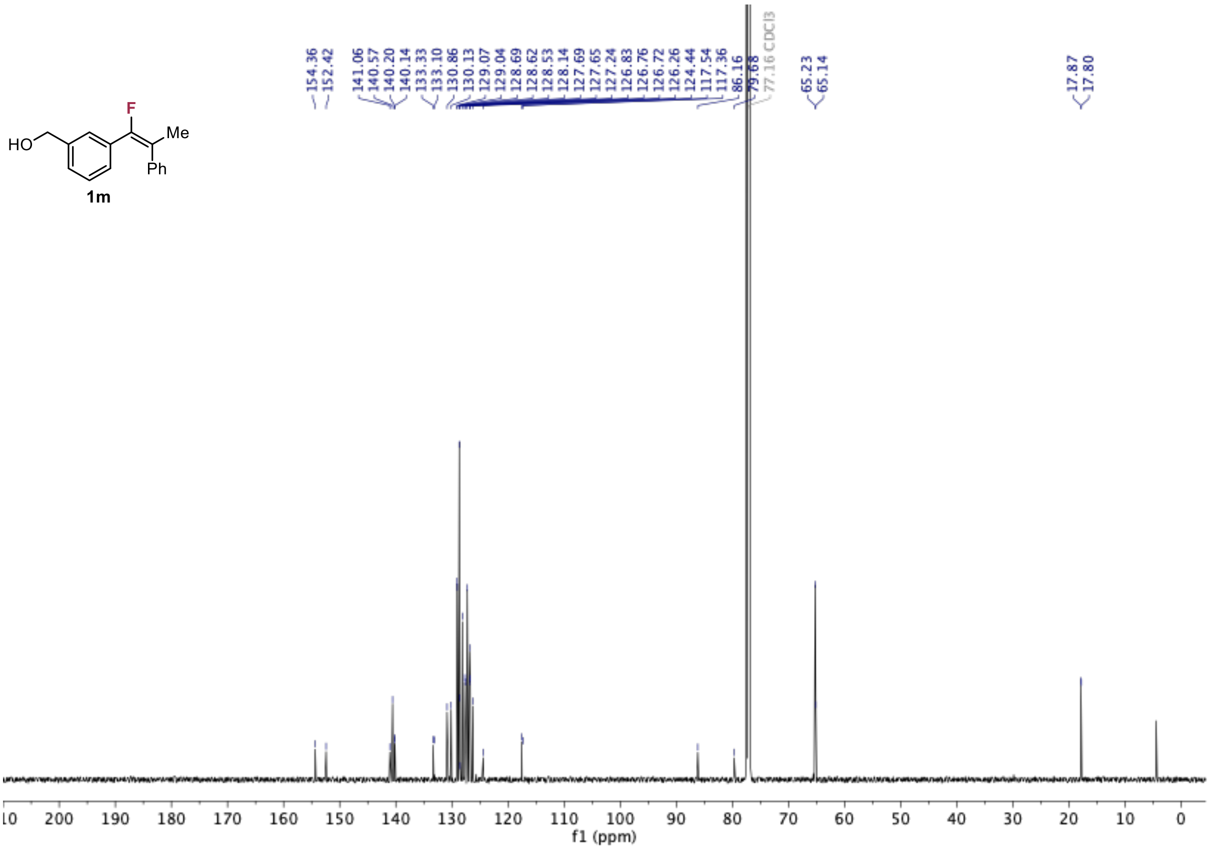

^{19}F NMR (471 MHz, CDCl$_3$) of (E)-(3-(1-fluoro-2-phenylprop-1-en-1-yl)phenyl)methanol (1m)

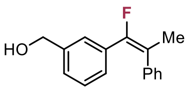

1m

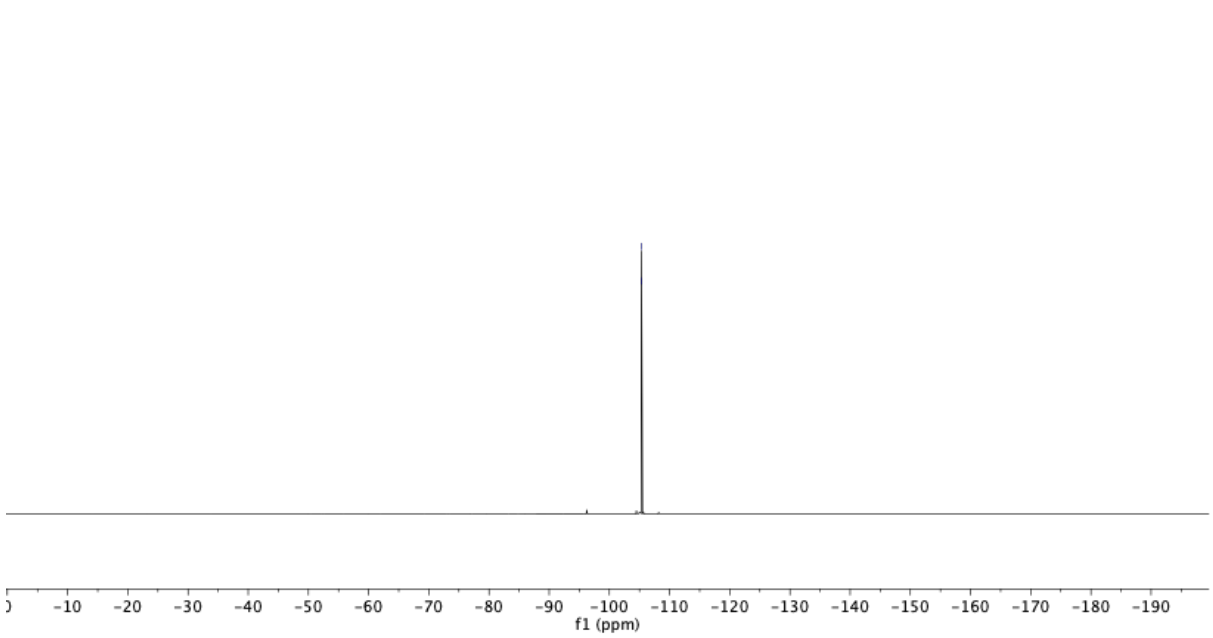

^{1}H NMR (500 MHz, $CDCl_3$) of 3-fluoro-1H-indene (1n)

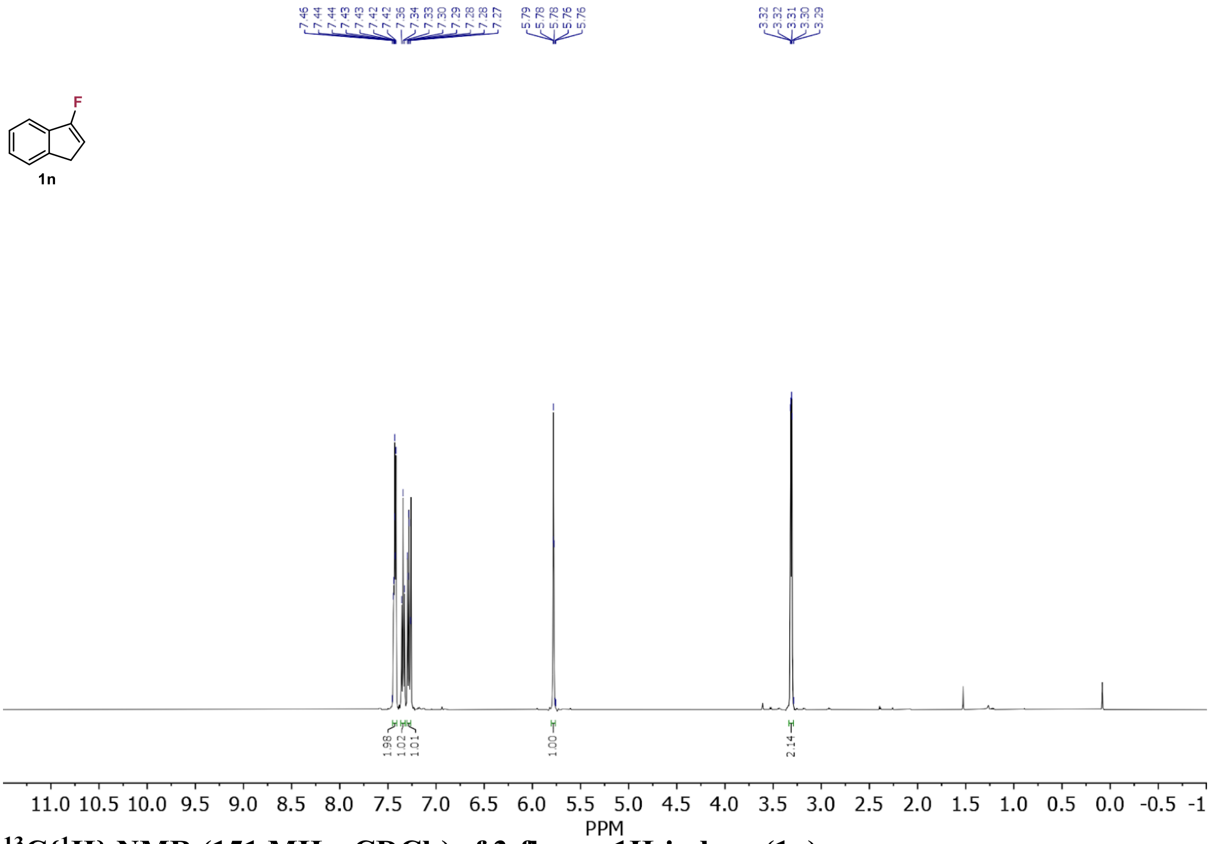

^{13}C{^{1}H} NMR (151 MHz, $CDCl_3$) of 3-fluoro-1H-indene (1n)

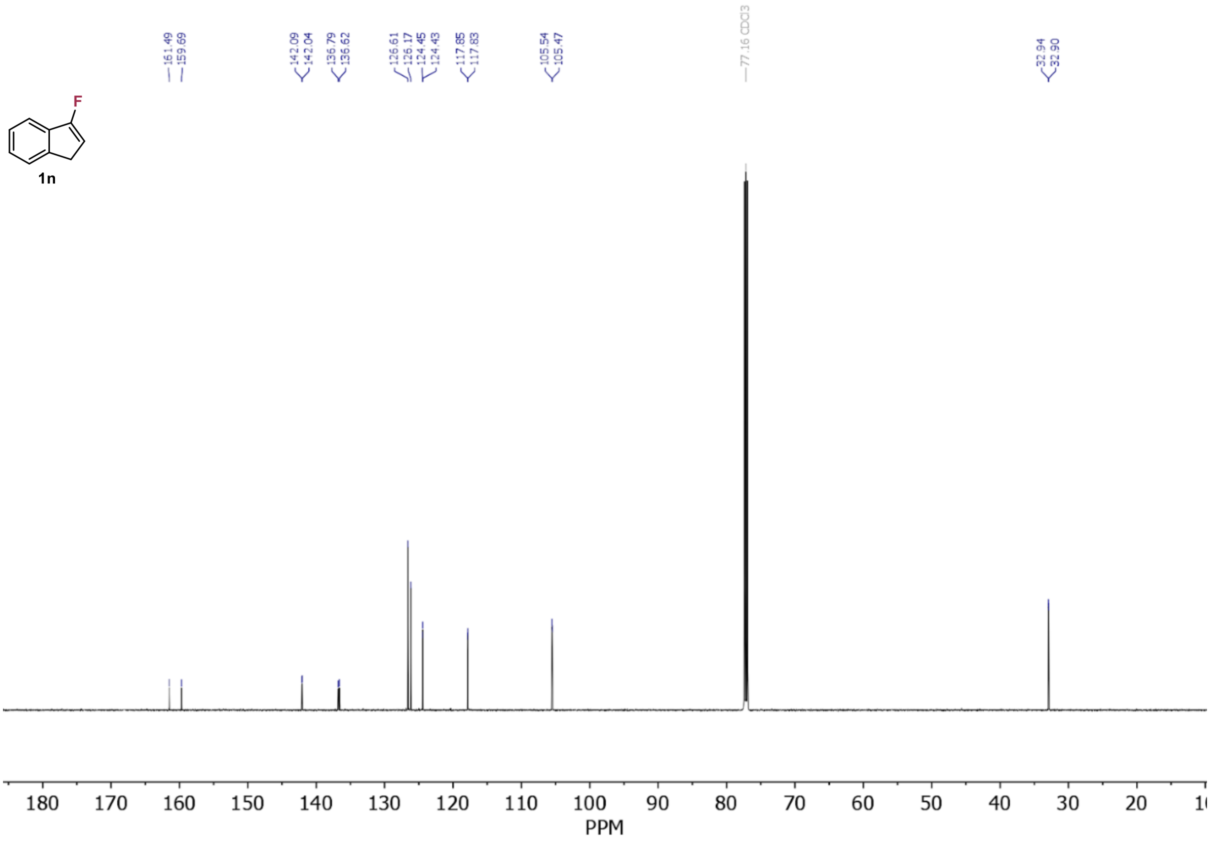

^{19}F {^{1}H} NMR (471 MHz, $CDCl_3$) of 3-fluoro-1H-indene (1n)

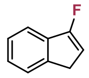

1n

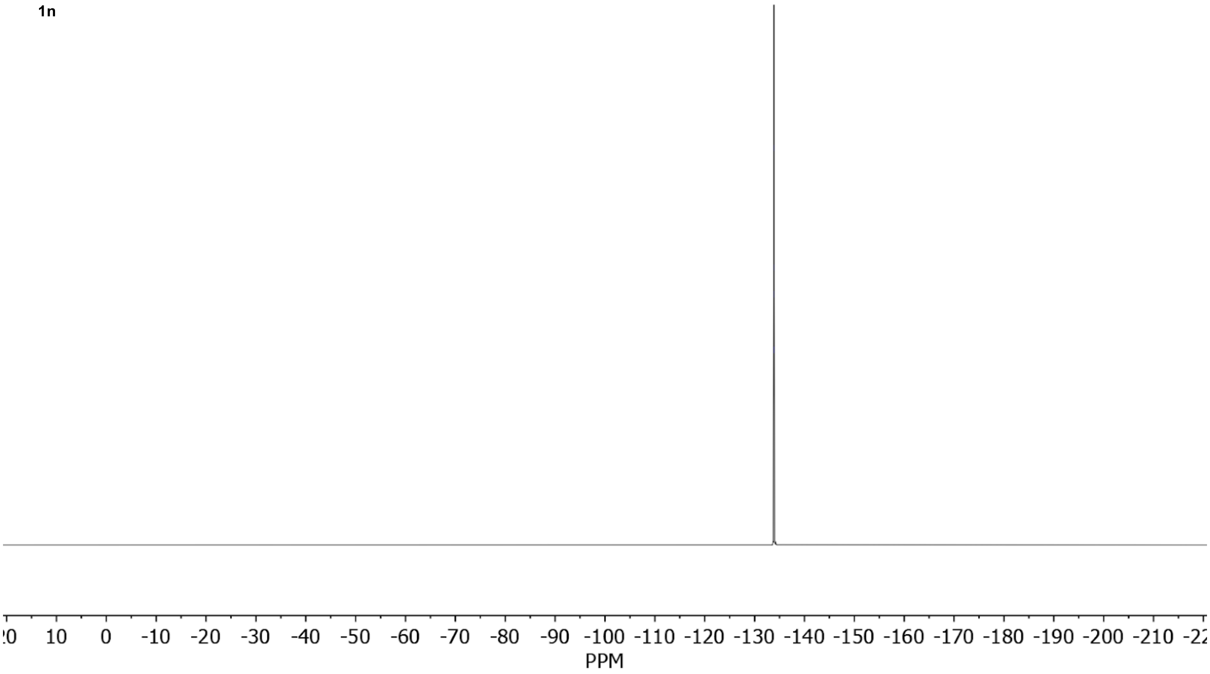

^{1}H NMR (500 MHz, CDCl$_3$) of 2-(7-fluorooct-7-en-1-yl)isoindoline-1,3-dione (1q)

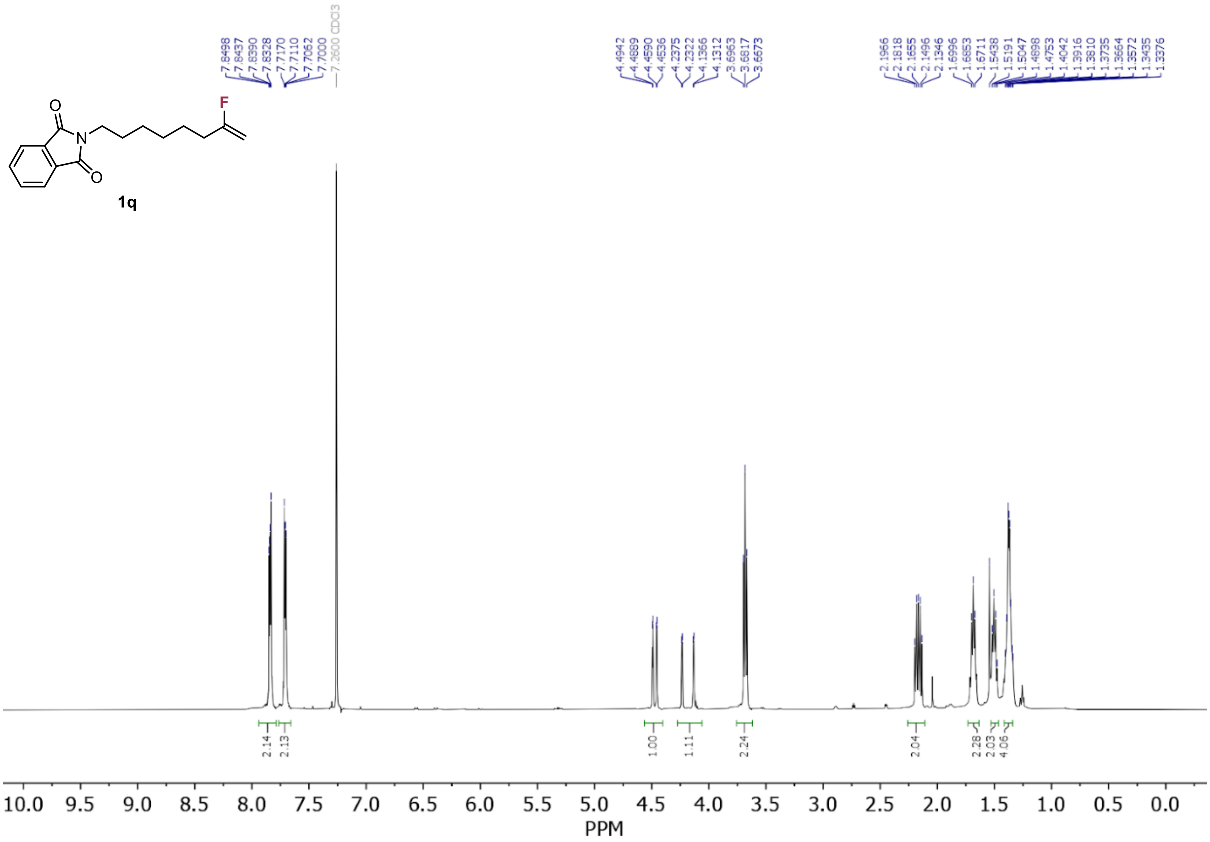

^{13}C{^{1}H} NMR (126 MHz, CDCl$_3$) of 2-(7-fluorooct-7-en-1-yl)isoindoline-1,3-dione (1q)

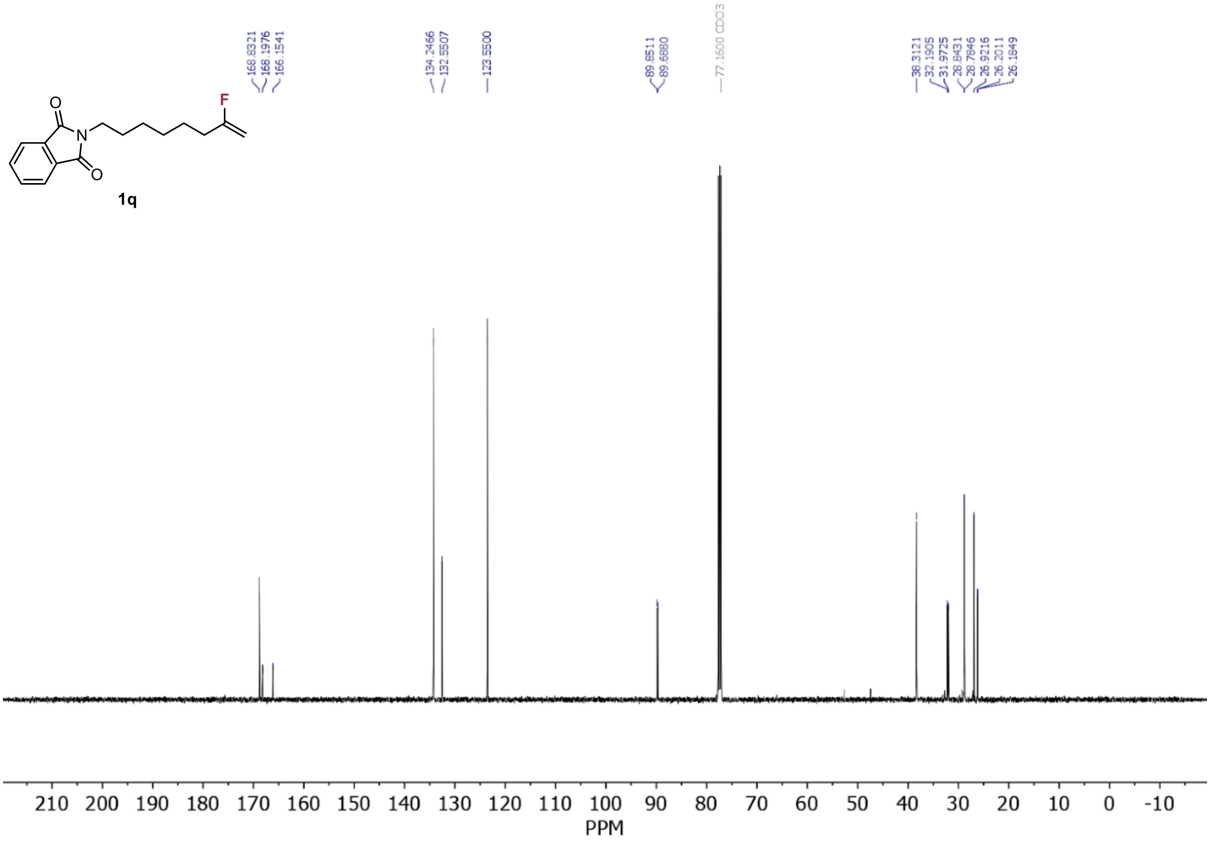

$^{19}F\{^{1}H\}$ NMR (471 MHz, $CDCl_3$) of 2-(7-fluorooct-7-en-1-yl)isoindoline-1,3-dione (1q)

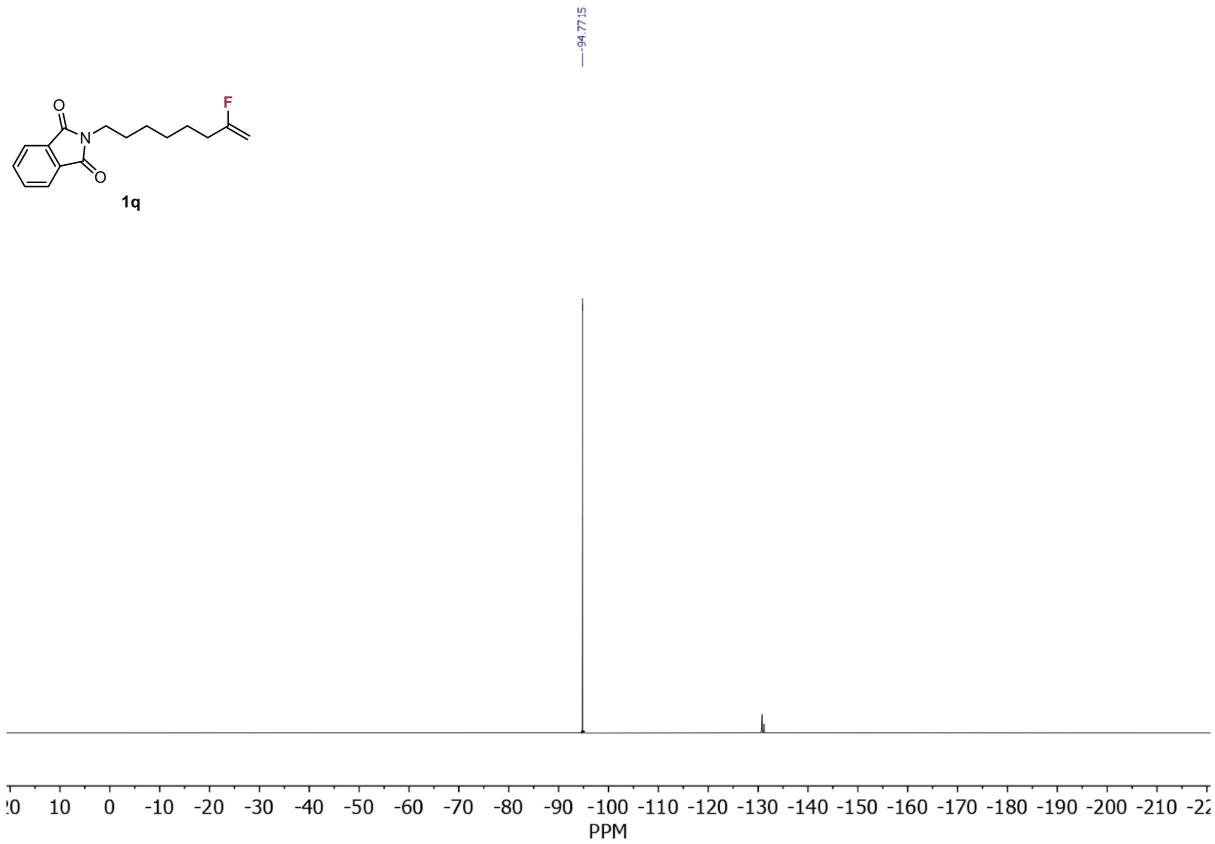

^{1}H NMR (500 MHz, $CDCl_3$) of 5-(phenethylsulfonyl)-1-phenyl-1*H*-tetrazole

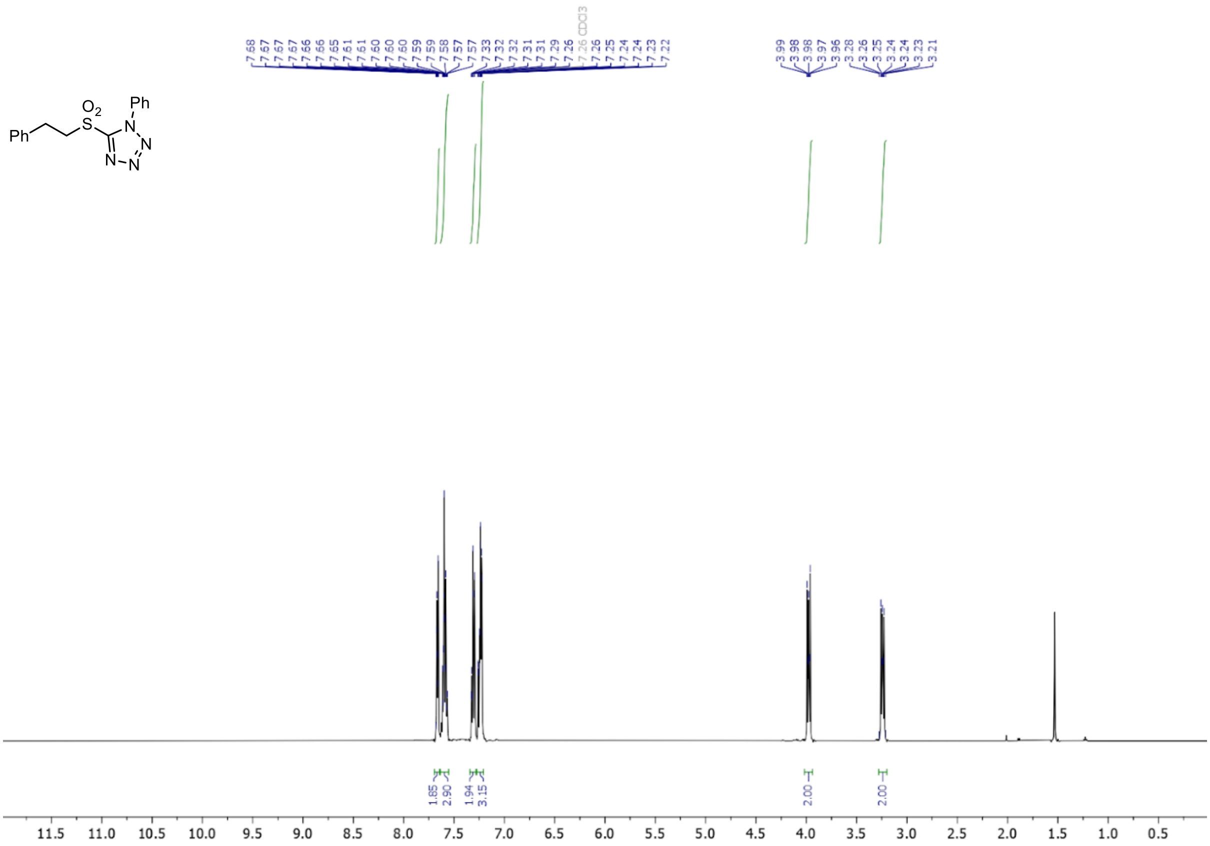

$^{13}C\{^{1}H\}$ NMR (126 MHz, $CDCl_3$) of 5-(phenethylsulfonyl)-1-phenyl-1*H*-tetrazole

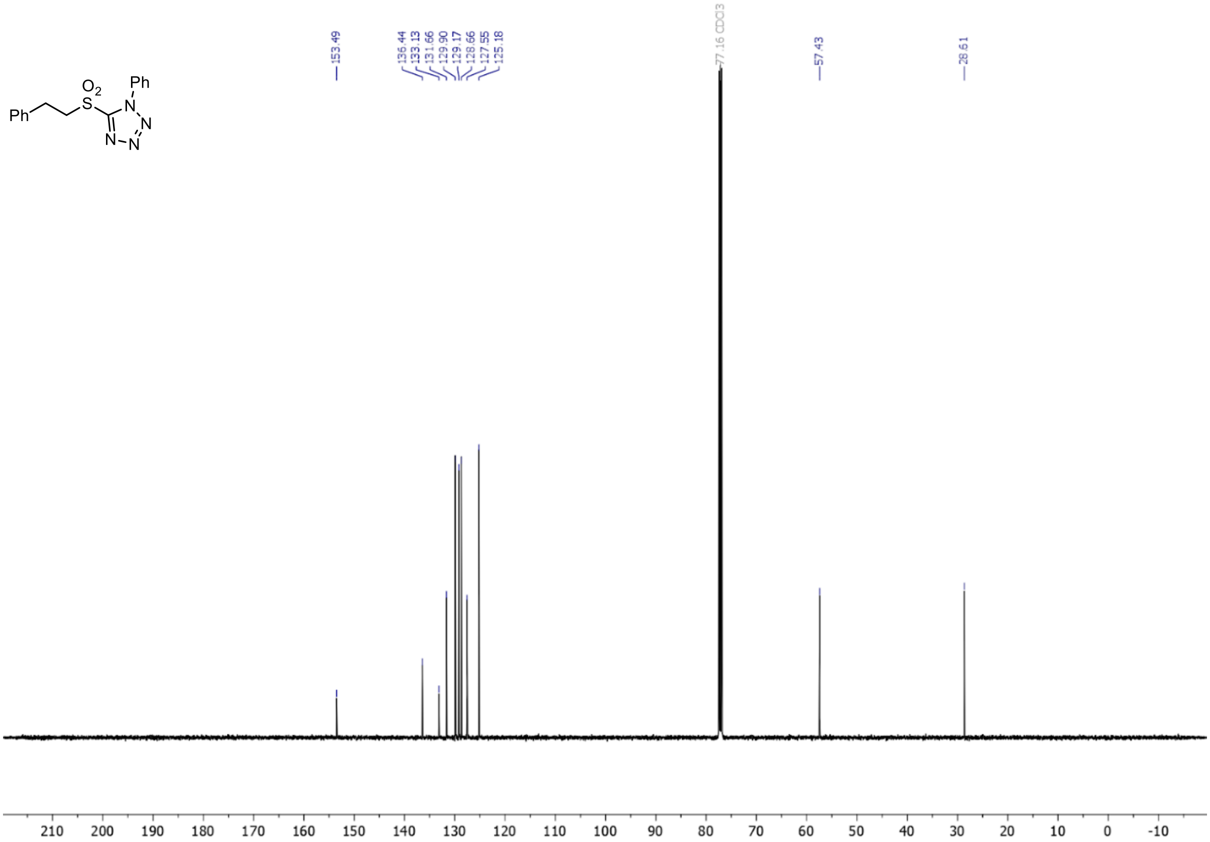

^{1}H NMR (500 MHz, CDCl$_3$) of 5-((2-methyl-2-phenylpropyl)sulfonyl)-1-phenyl-1*H*-tetrazole

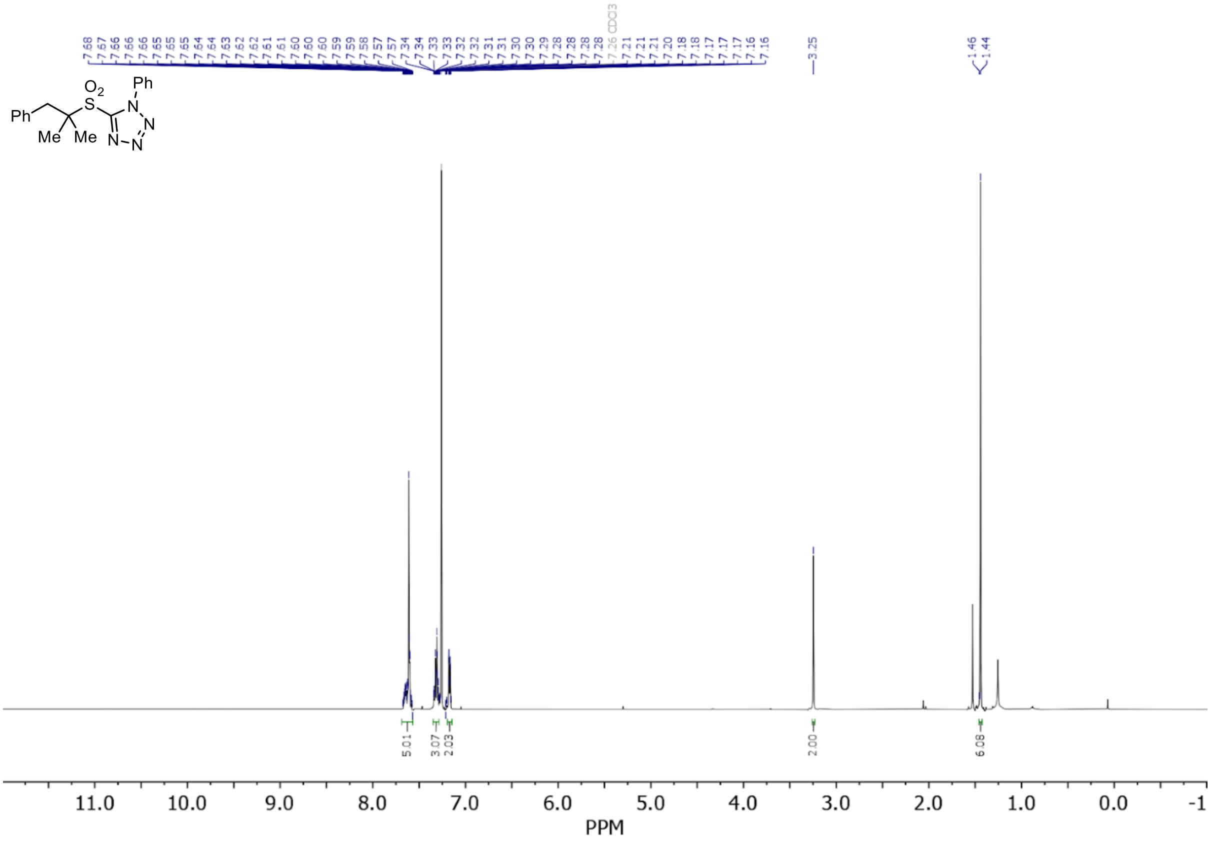

^{13}C{^{1}H} NMR (126 MHz, CDCl$_3$) of 5-((2-methyl-2-phenylpropyl)sulfonyl)-1-phenyl-1*H*-tetrazole

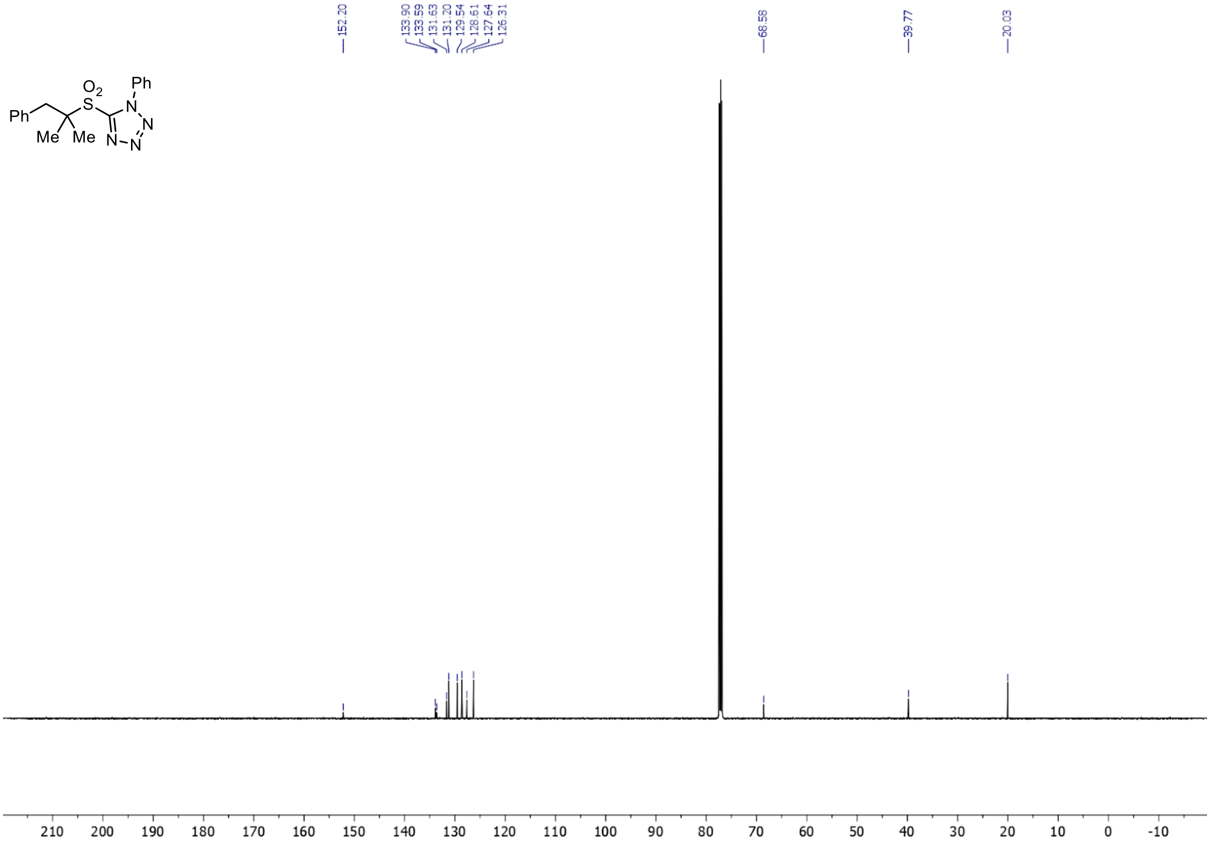

¹H NMR (500 MHz, CDCl₃) of (*Z*)-1-(2-fluoro-3,3-dimethyl-4-phenylbut-1-en-1-yl)-4-methoxybenzene (1s)

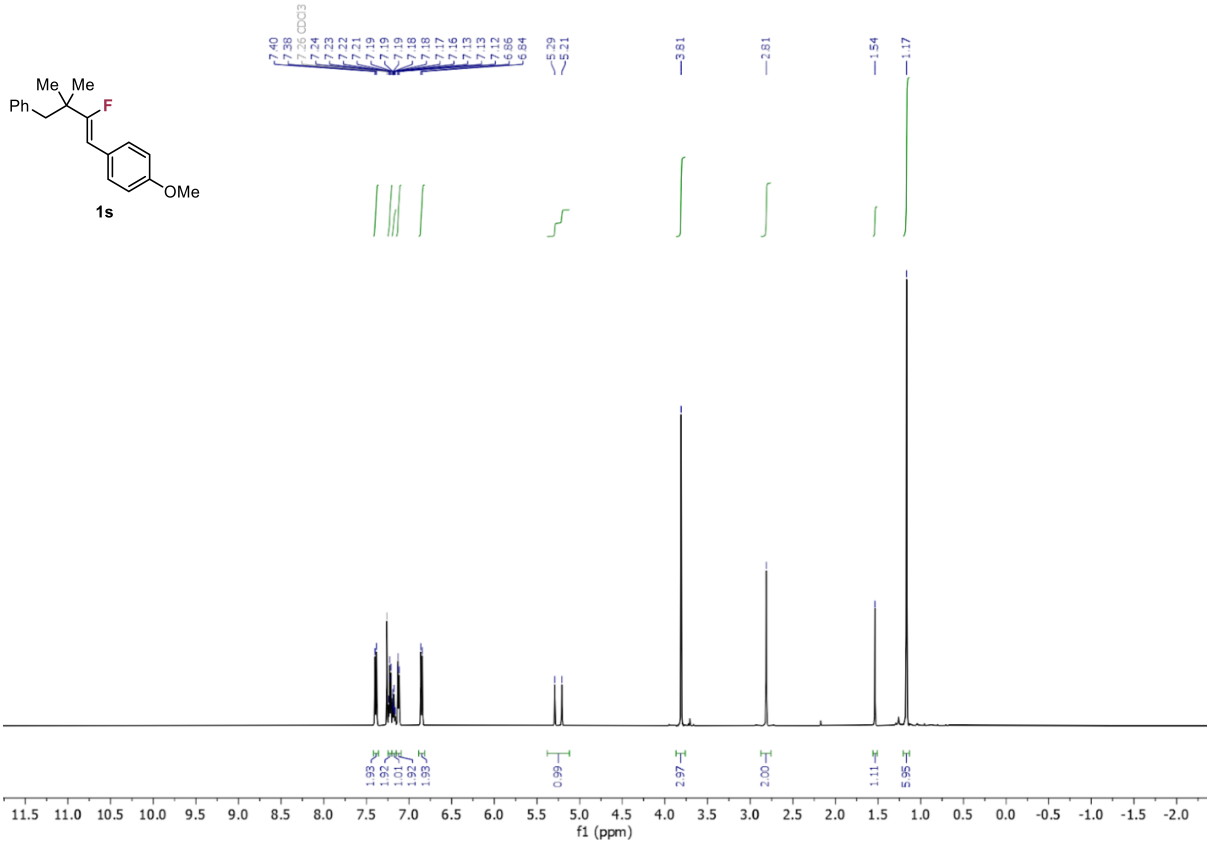

$^{13}C\{^{1}H\}$ NMR (126 MHz, CDCl₃) of (*Z*)-1-(2-fluoro-3,3-dimethyl-4-phenylbut-1-en-1-yl)-4-methoxybenzene (1s)

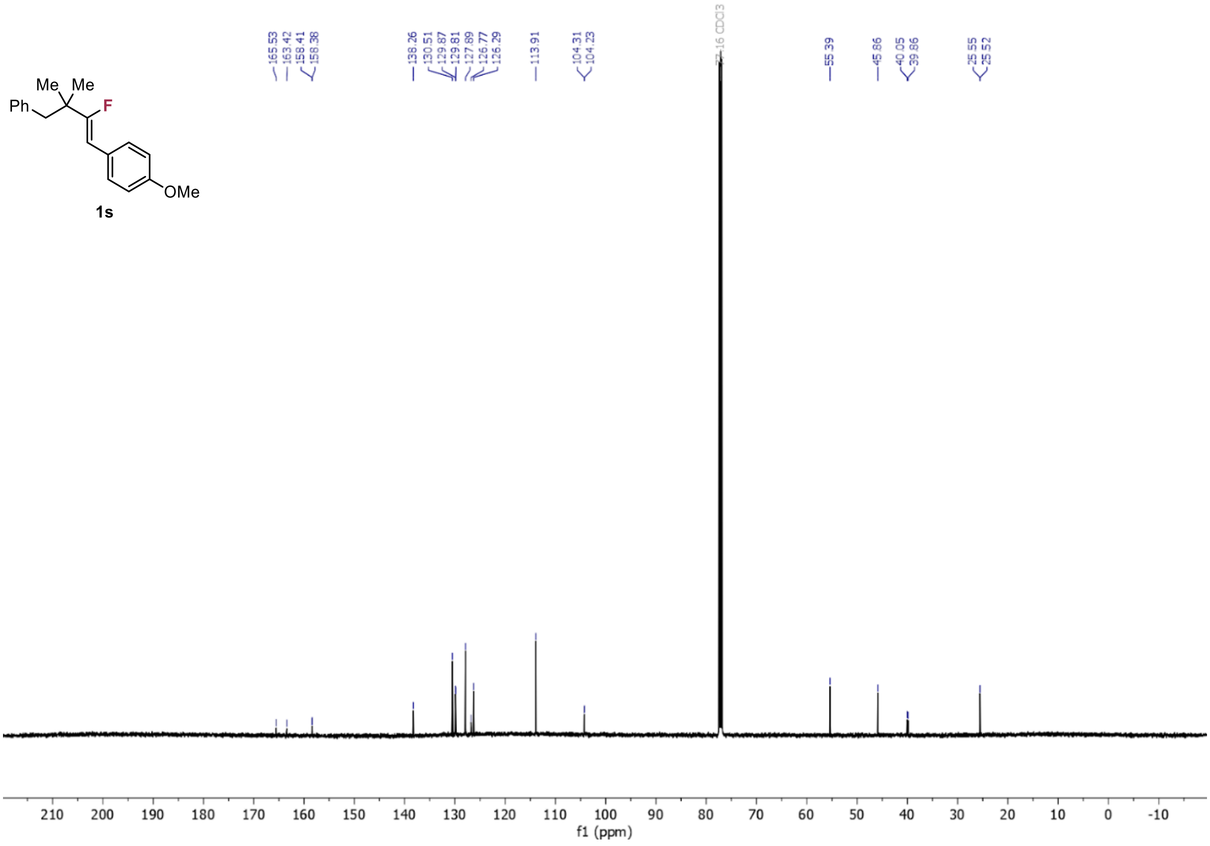

$^{19}F\{^1H\}$ NMR (471 MHz, CDCl$_3$) of **(Z)-1-(2-fluoro-3,3-dimethyl-4-phenylbut-1-en-1-yl)-4-methoxybenzene (1s)**

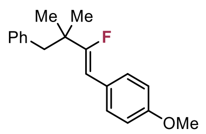

1s

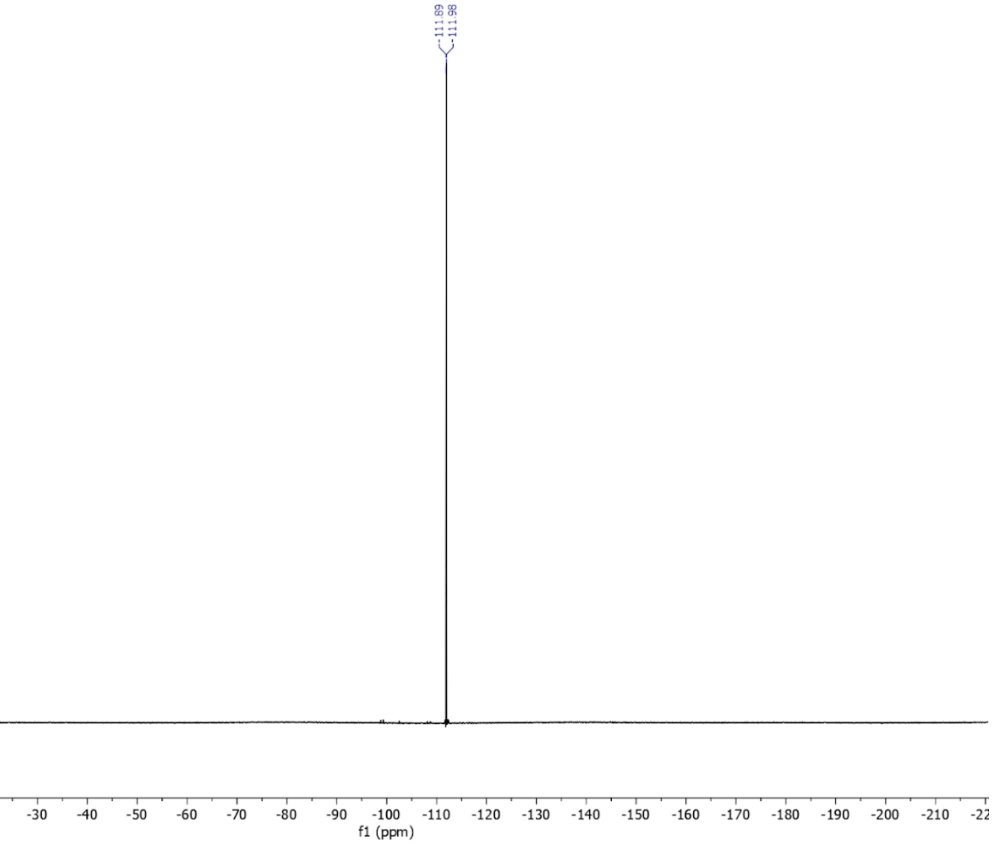

^{1}H NMR (500 MHz, $CDCl_3$) of (*Z*)-1-(*tert*-butyl)-4-(2-cyclohexyl-2-fluorovinyl)benzene (1t)

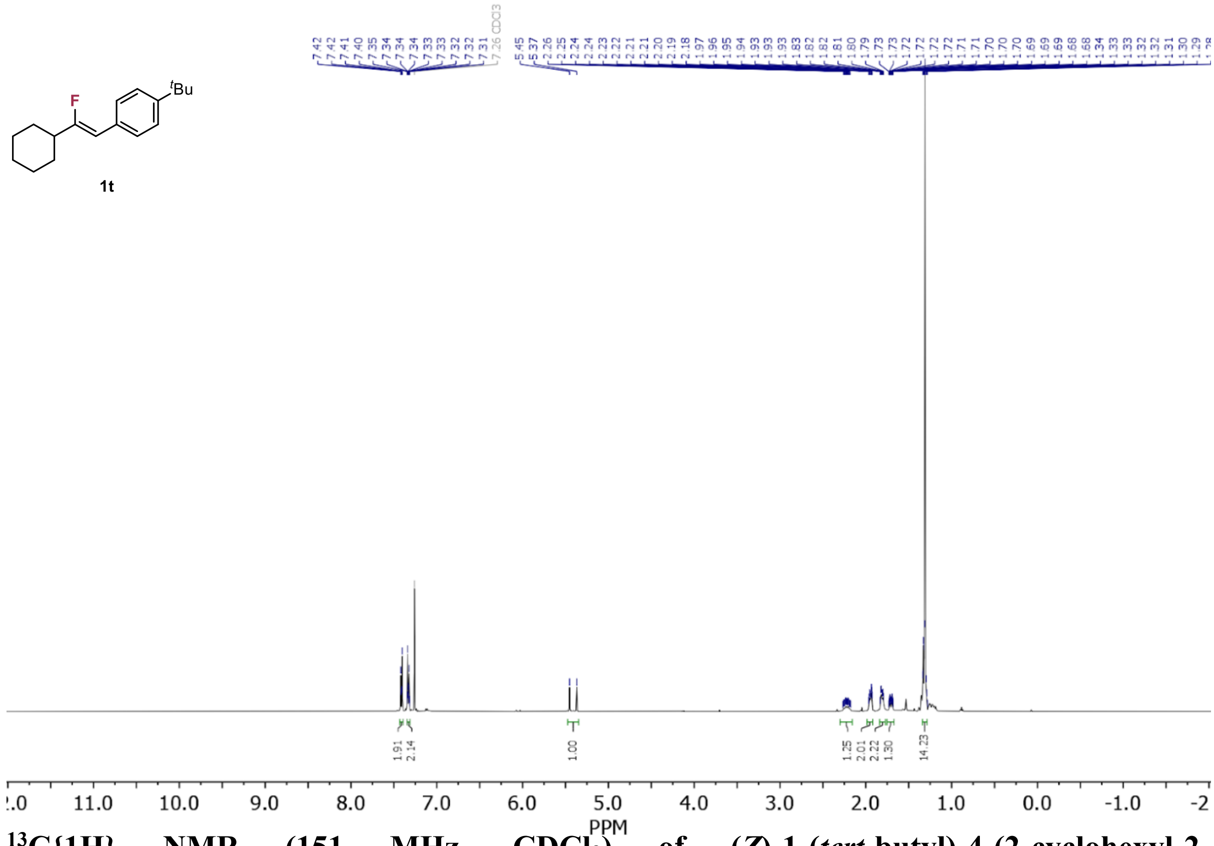

^{13}C{1H} NMR (151 MHz, $CDCl_3$) of (*Z*)-1-(*tert*-butyl)-4-(2-cyclohexyl-2-fluorovinyl)benzene (1t)

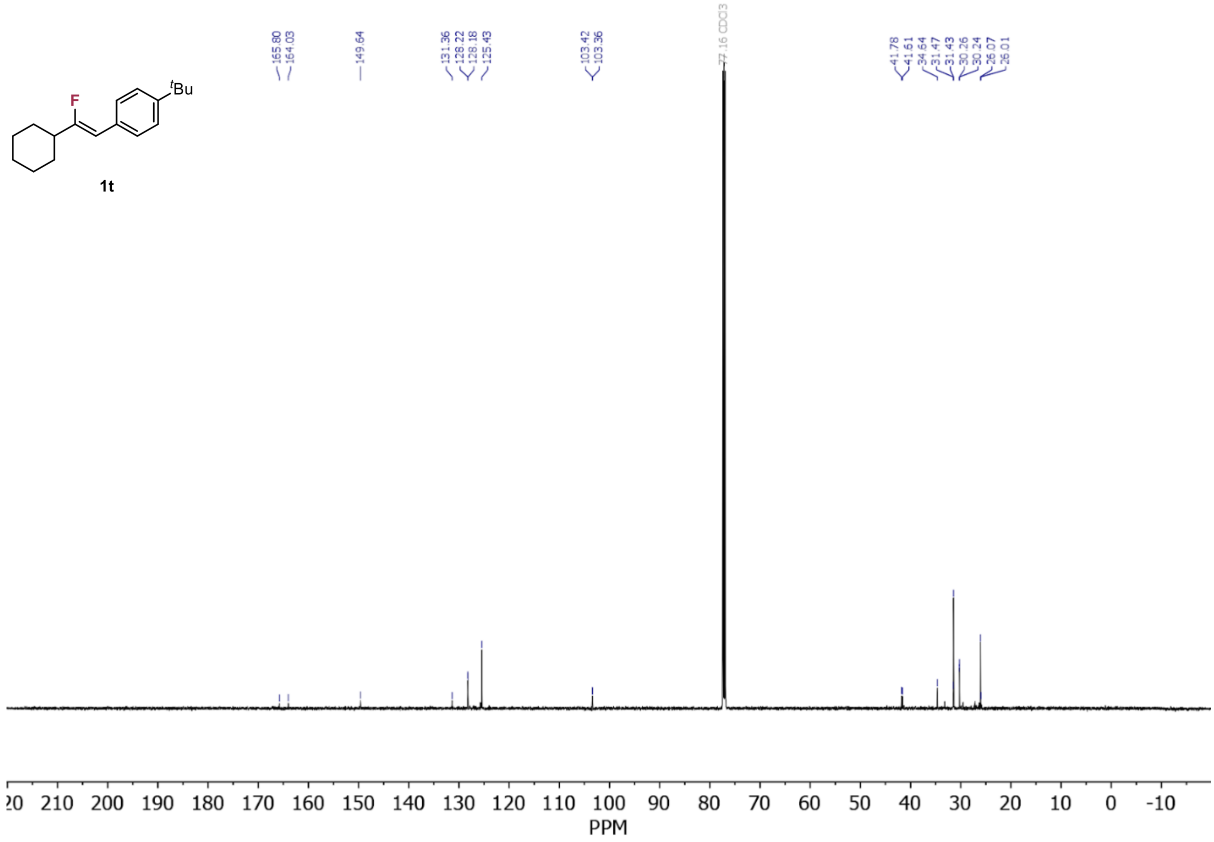

$^{19}F\{^{1}H\}$ NMR (471 MHz, $CDCl_3$) of (*Z*)-1-(*tert*-butyl)-4-(2-cyclohexyl-2-fluorovinyl)benzene (1t)

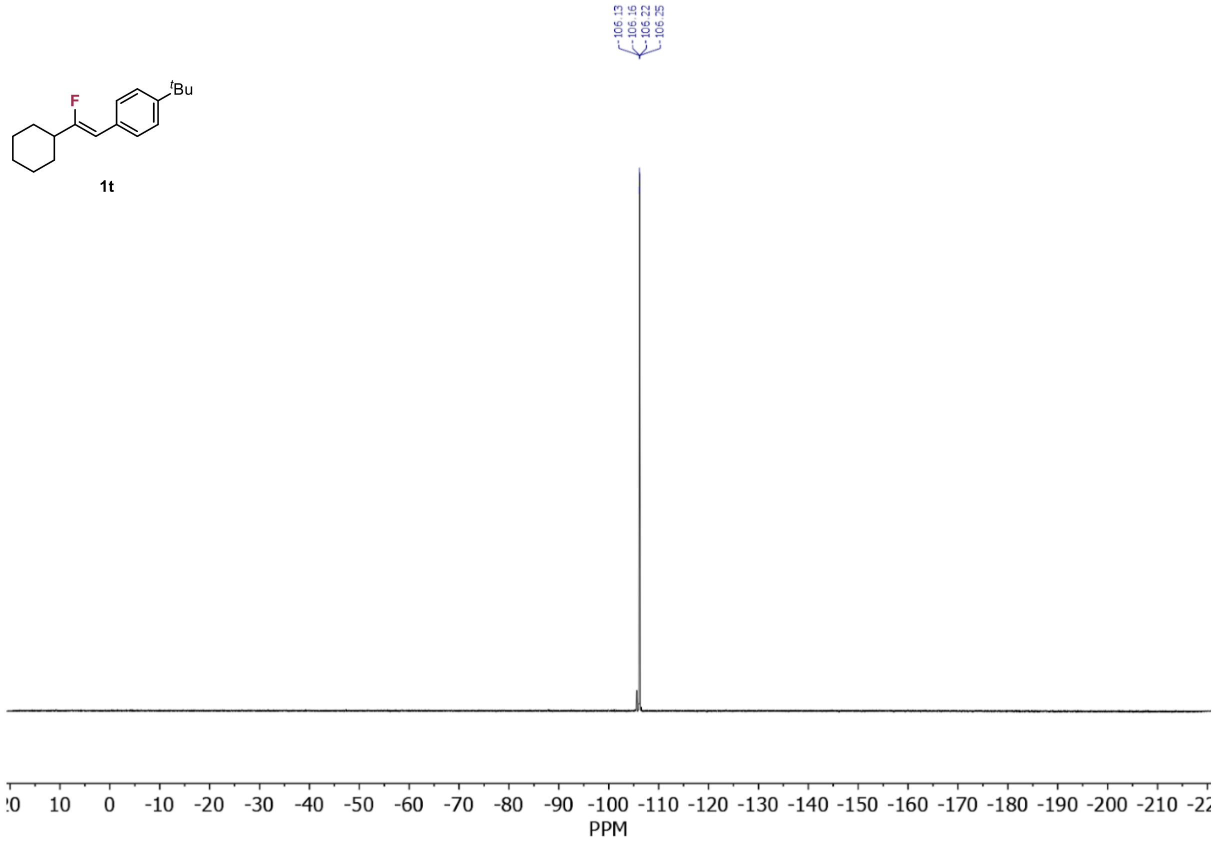

^{1}H NMR (500 MHz, CDCl$_3$) of (*Z*)-2-(2-fluoro-3-(*p*-tolyl)allyl)isoindoline-1,3-dione (1v)

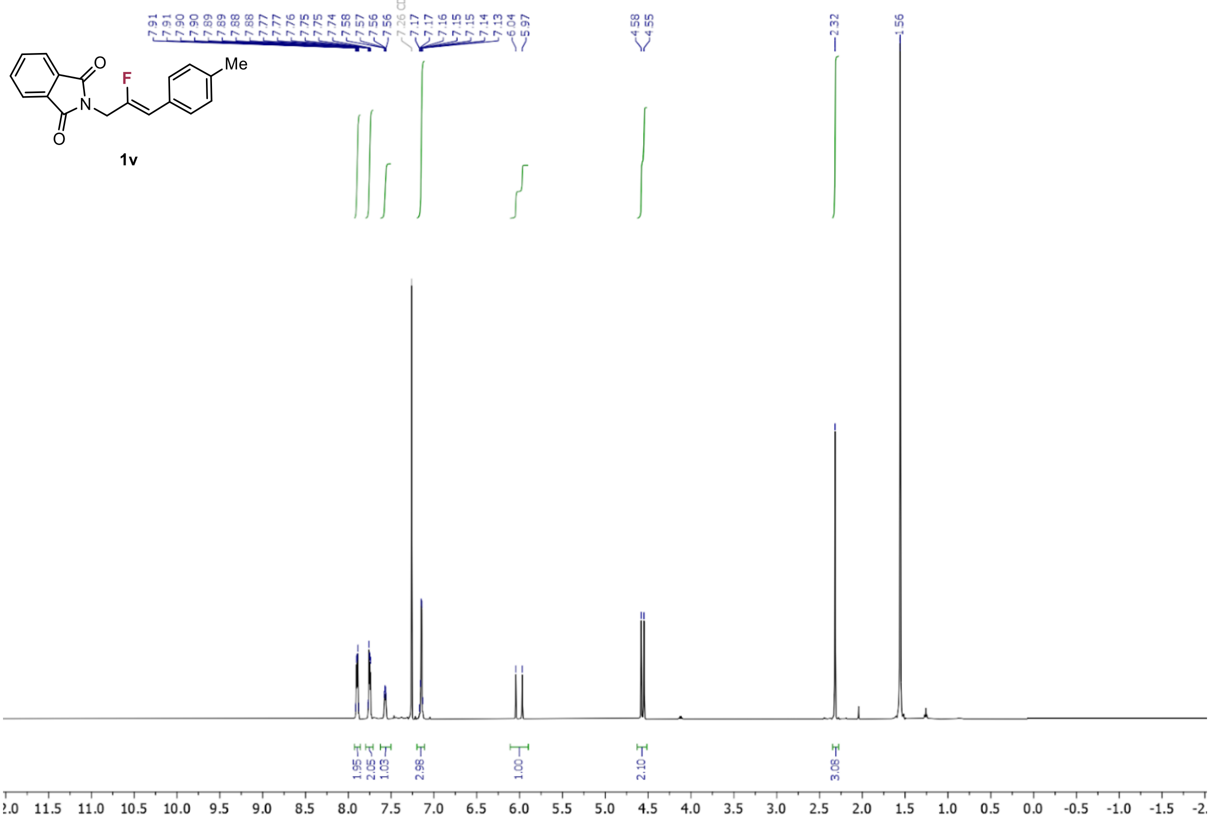

^{13}C{^{1}H} NMR (126 MHz, CDCl$_3$) of (*Z*)-2-(2-fluoro-3-(*p*-tolyl)allyl)isoindoline-1,3-dione (1v)

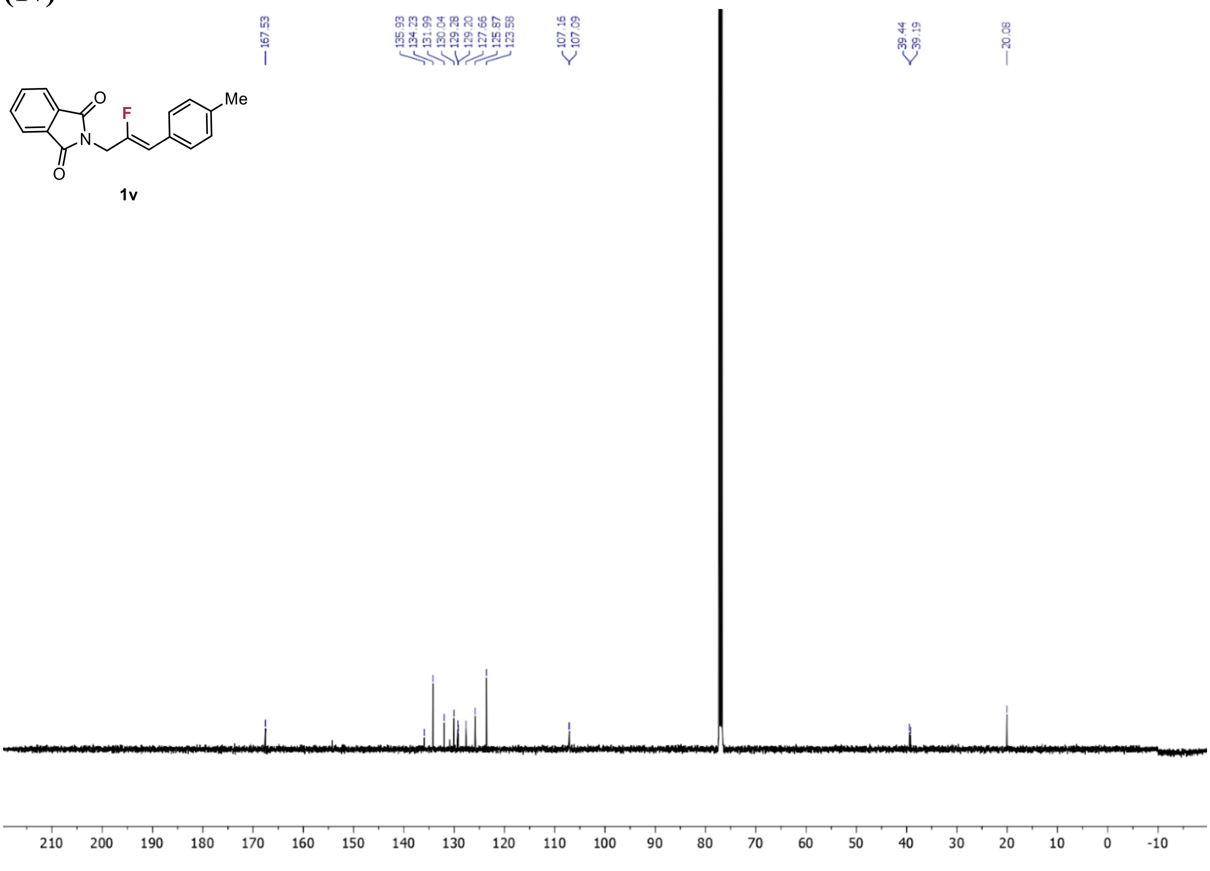

$^{19}F\{^1H\}$ NMR (471 MHz, $CDCl_3$) of (*Z*)-2-(2-fluoro-3-(*p*-tolyl)allyl)isoindoline-1,3-dione (1v)

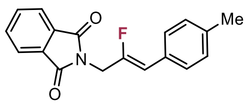

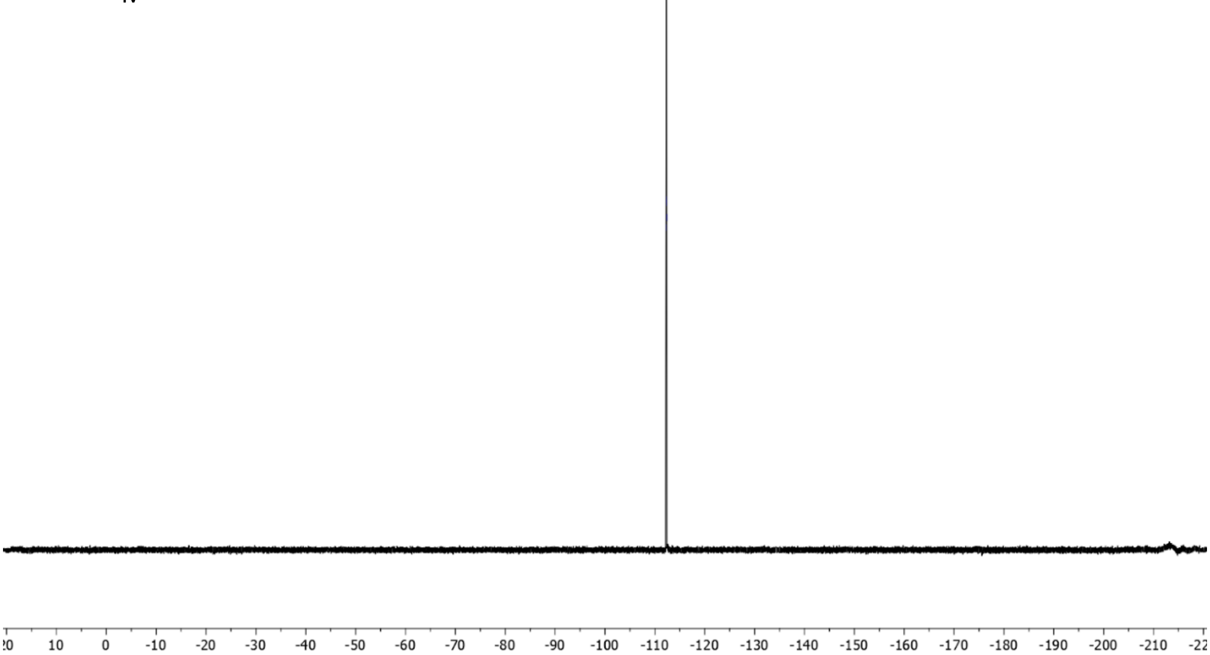

^{1}H NMR (500 MHz, $CDCl_3$) of (*Z*)-2-fluoro-3-(*p*-tolyl)prop-2-en-1-amine

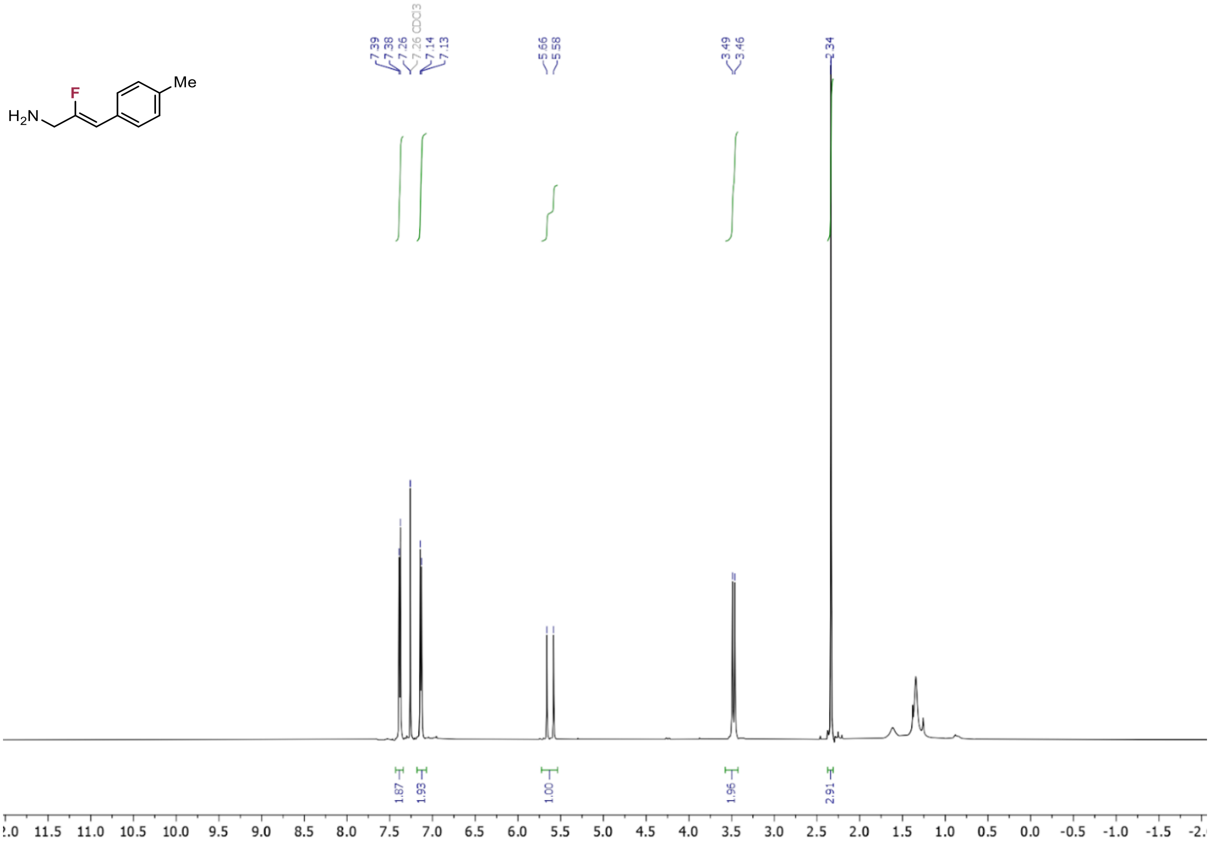

^{13}C{^{1}H} NMR (126 MHz, $CDCl_3$) of (*Z*)-2-fluoro-3-(*p*-tolyl)prop-2-en-1-amine

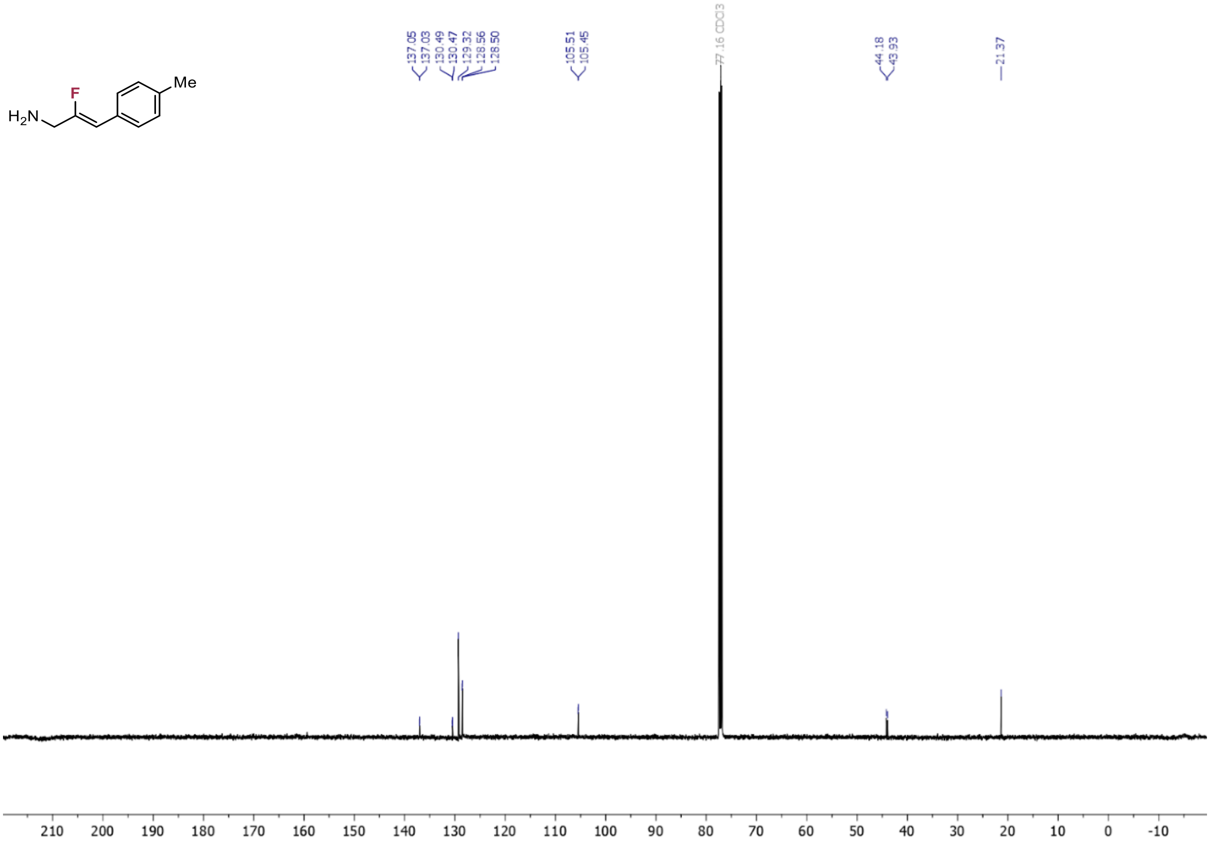

$^{19}F\{^1H\}$ NMR (471 MHz, $CDCl_3$) of (*Z*)-2-fluoro-3-(*p*-tolyl)prop-2-en-1-amine

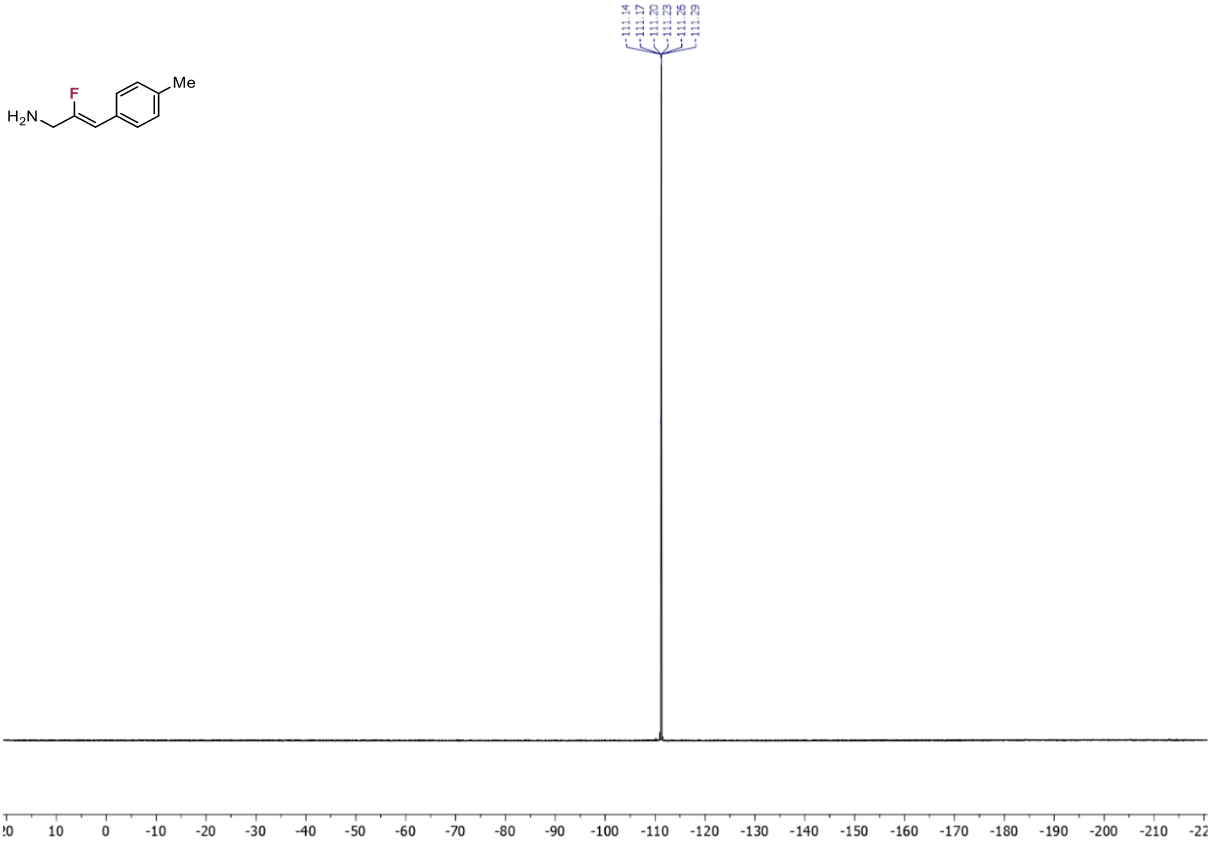

^{1}H NMR (500 MHz, CDCl$_3$) of *tert*-butyl (*S*,*Z*)-(1-((2-fluoro-3-(*p*-tolyl)allyl)amino)-1-oxo-3-phenylpropan-2-yl)carbamate (4b)

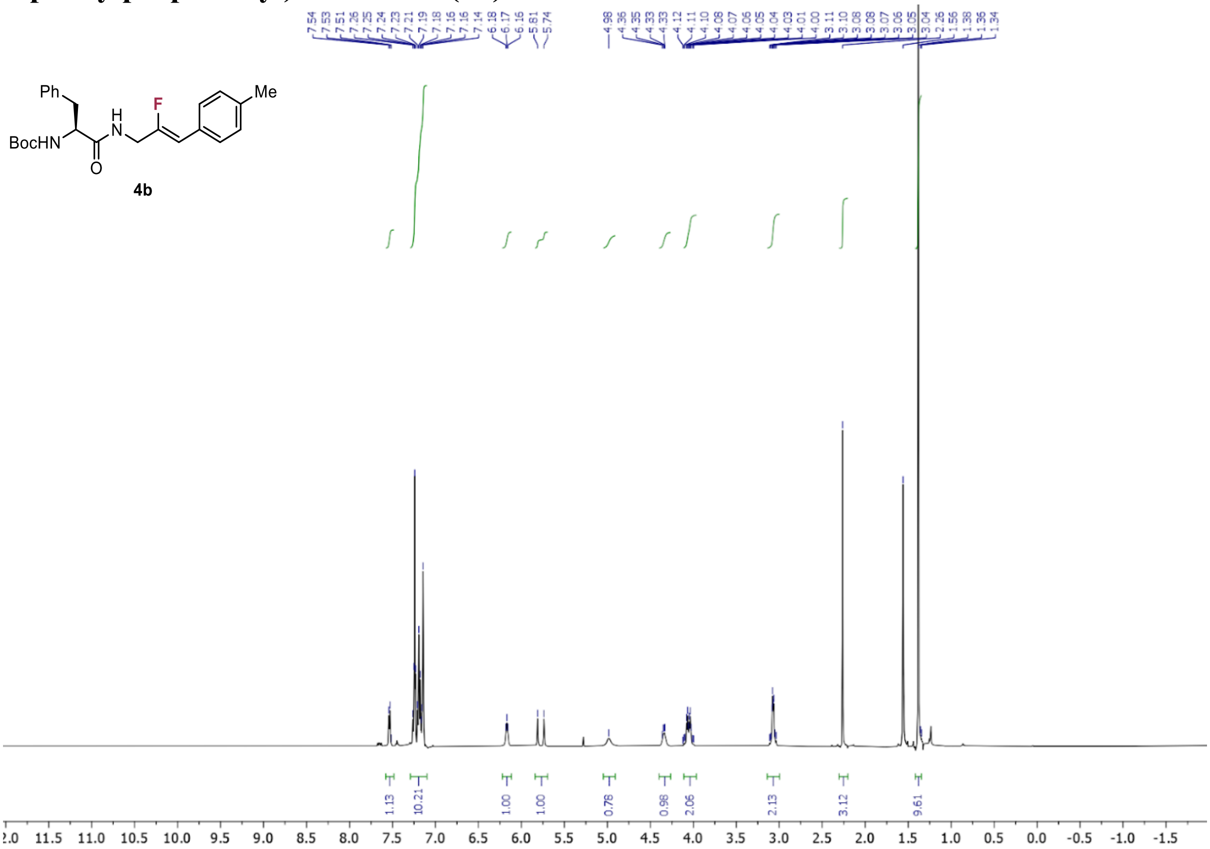

^{13}C{^{1}H} NMR (126 MHz, CDCl$_3$) of *tert*-butyl (*S*,*Z*)-(1-((2-fluoro-3-(*p*-tolyl)allyl)amino)-1-oxo-3-phenylpropan-2-yl)carbamate (4b)

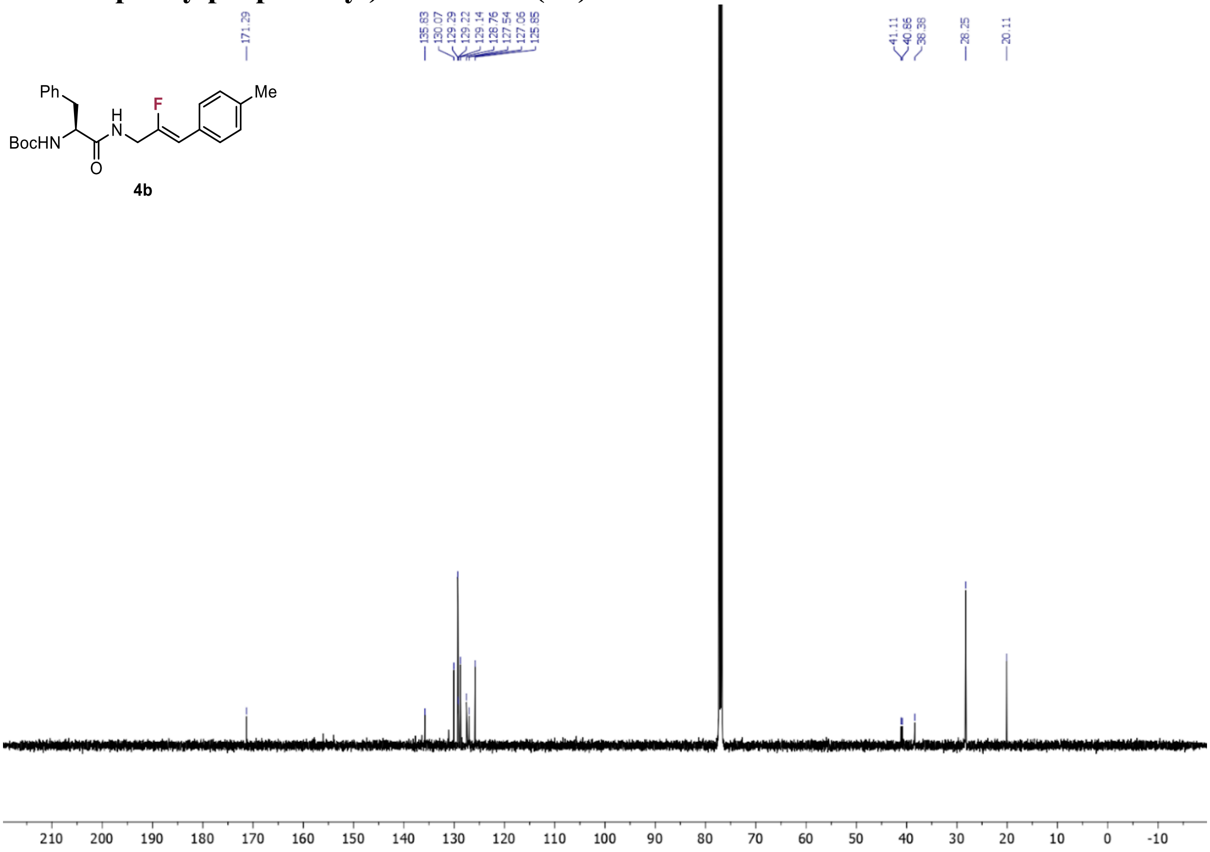

$^{19}F\{^{1}H\}$ NMR (471 MHz, $CDCl_3$) of *tert*-butyl (*S*,*Z*)-(1-((2-fluoro-3-(*p*-tolyl)allyl)amino)-1-oxo-3-phenylpropan-2-yl)carbamate (4b)

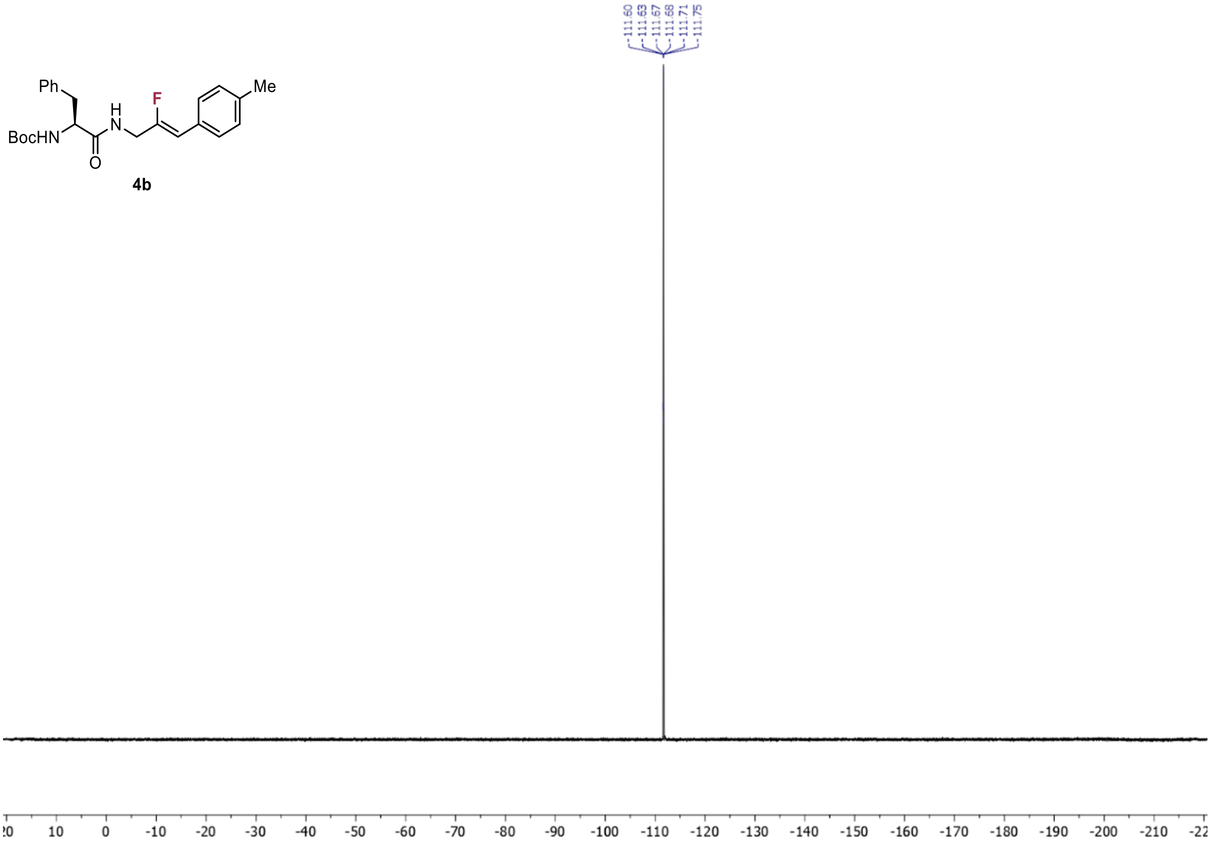

^{1}H NMR (500 MHz, DMSO) of BocHN-Gly-Trp(Boc)-Pro-(*D*)-Ala-Thr(*t*Bu)-OH

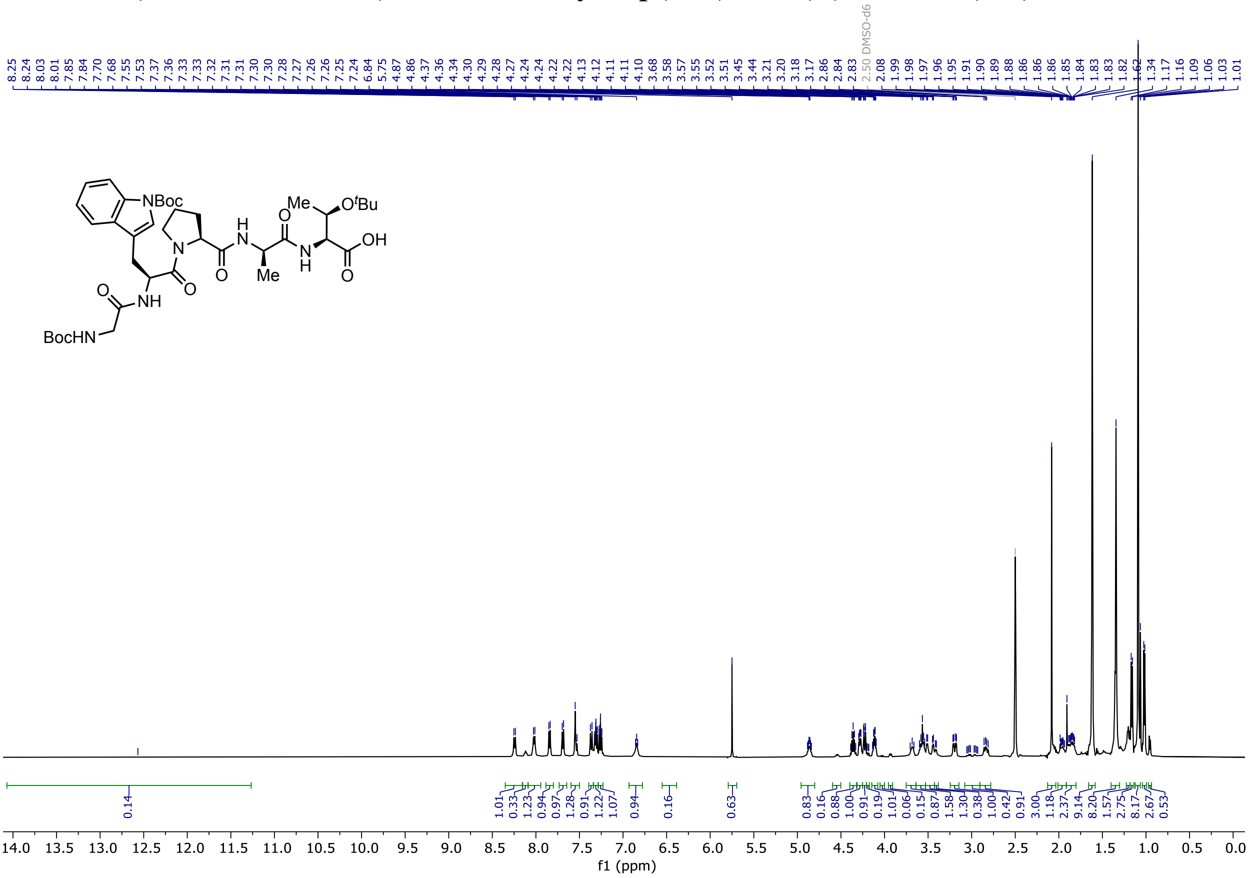

^{13}C{^{1}H} NMR (206 MHz, DMSO) of BocHN-Gly-Trp(Boc)-Pro-(*D*)-Ala-Thr(*t*Bu)-OH

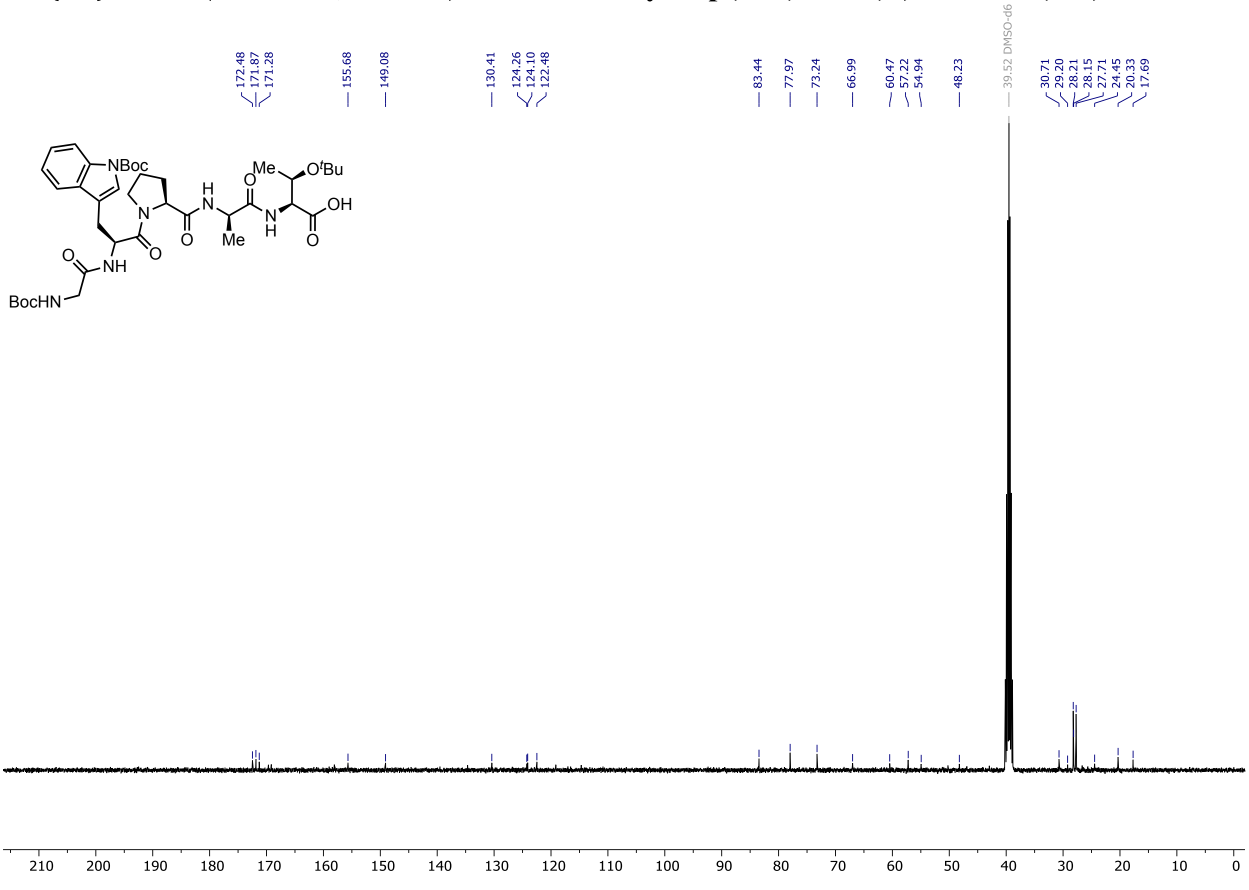

^{1}H NMR (500 MHz, CD$_3$OD) of BocHN-Gly-Trp(Boc)-Pro-(*D*)-Ala-Thr(^{t}Bu)-(Z)-3F-3(*p*-toly)prop-2-ene) (4c)

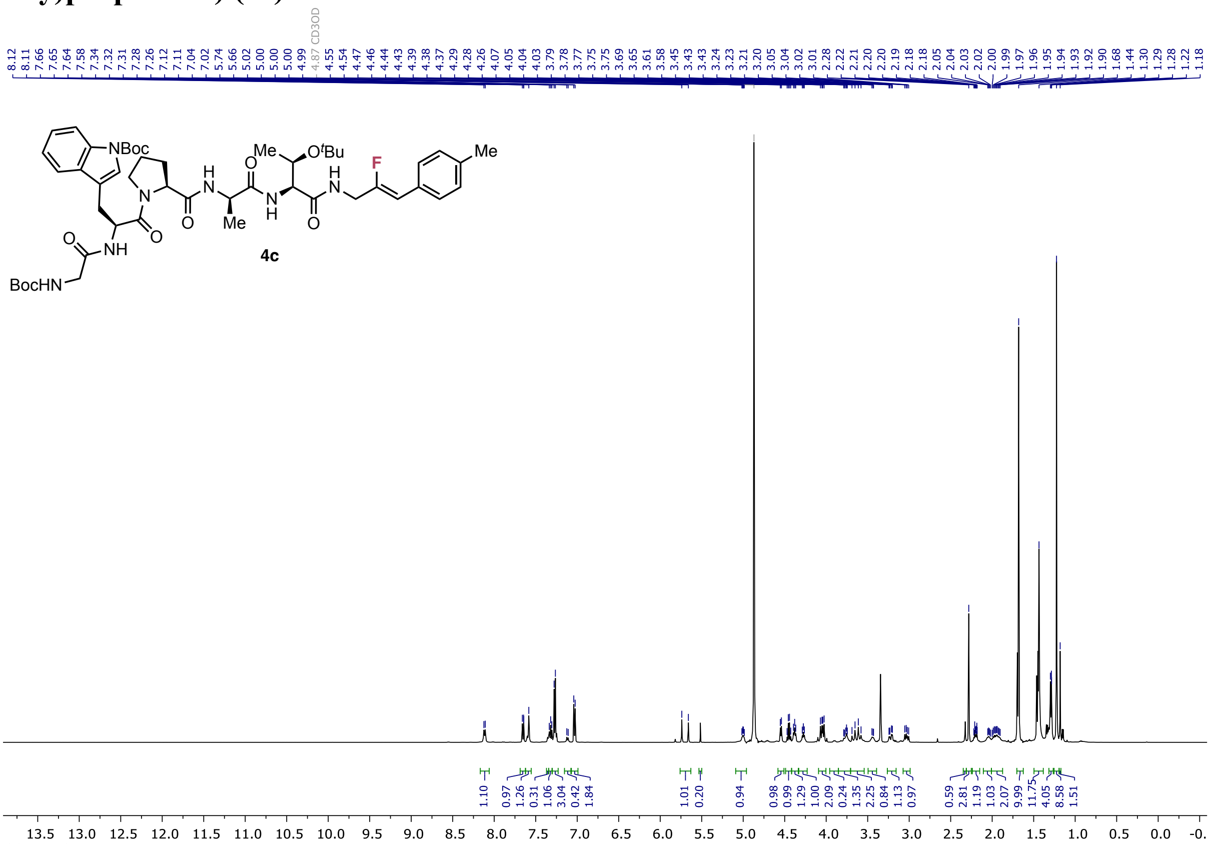

^{13}C{^{1}H} NMR (201 MHz, CD$_3$OD) of BocHN-Gly-Trp(Boc)-Pro-(*D*)-Ala-Thr(^{t}Bu)-(Z/E)-3F-3(*p*-toly)prop-2-ene) (4c)

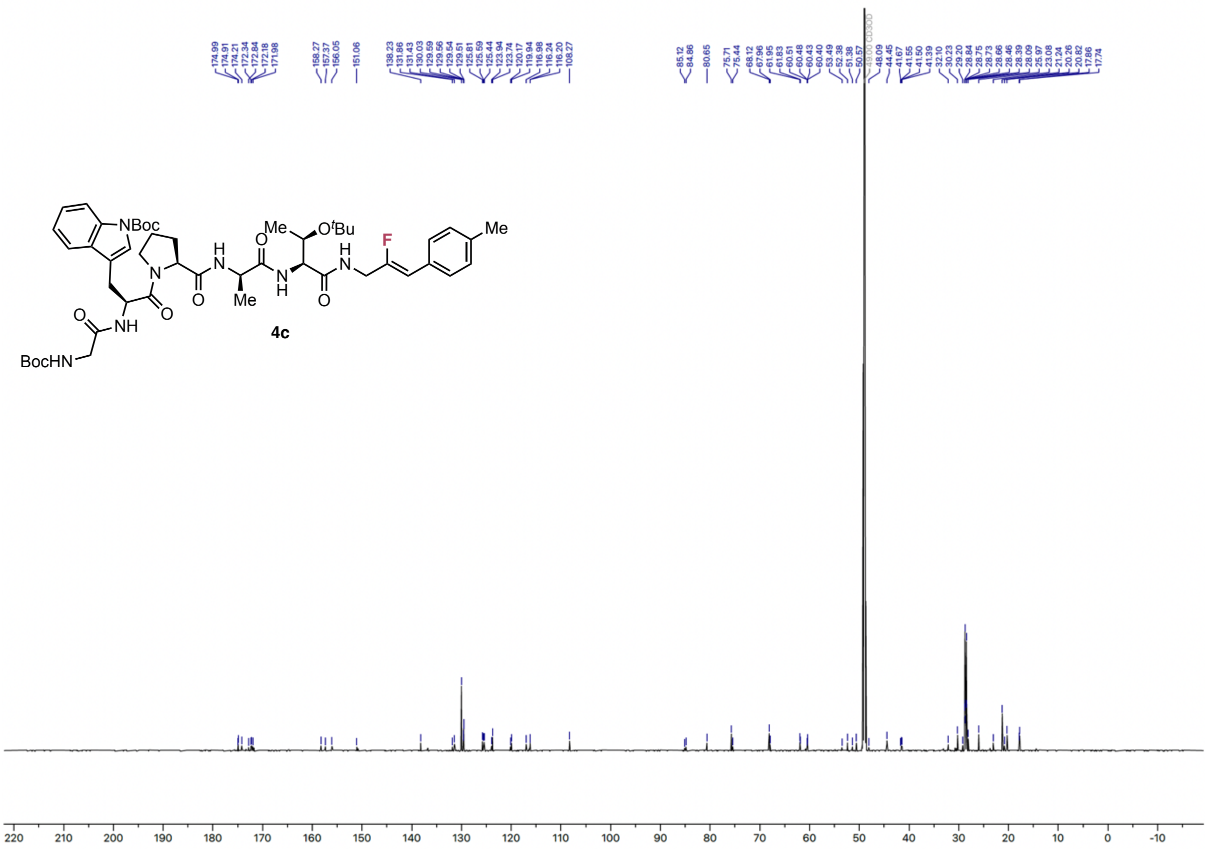

$^{19}F\{^{1}H\}$ NMR (758 MHz, CD_3OD) of BocHN-Gly-Trp(Boc)-Pro-(*D*)-Ala-Thr(^{t}Bu)-(Z)-3F-3(*p*-toly)prop-2-ene) (4c)

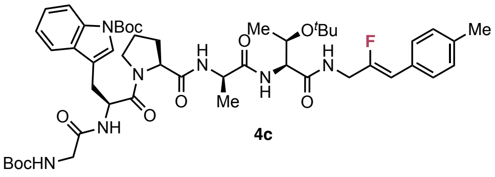

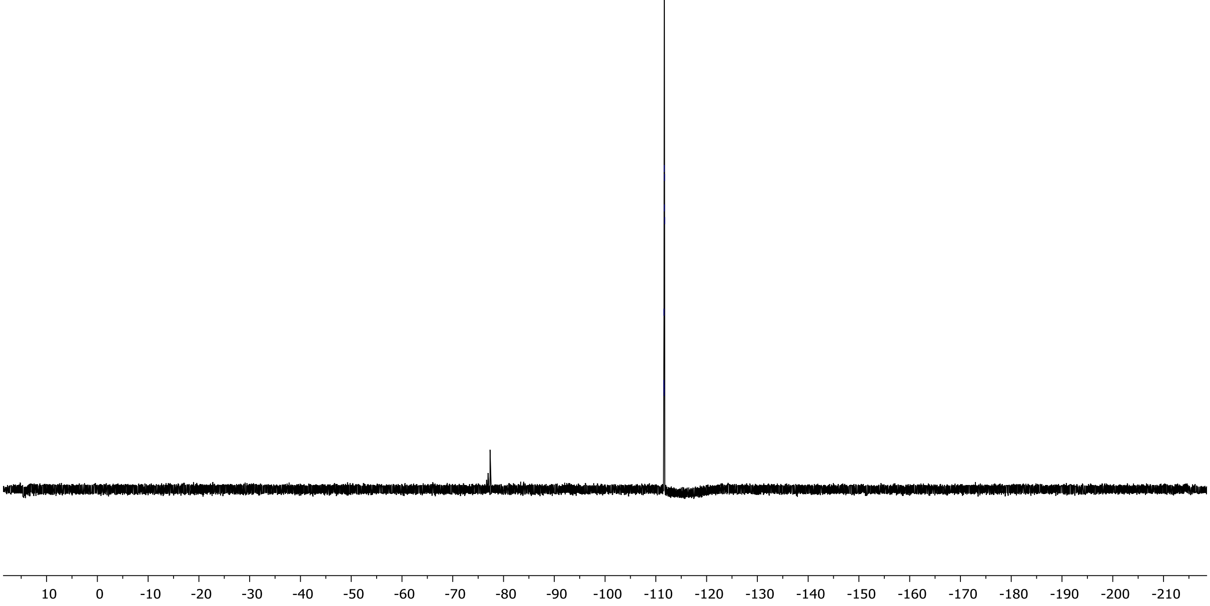

NMR spectra of products

^{1}H NMR (500 MHz, $CDCl_3$) of *N*-hexyl-4-methylbenzamide (2a)

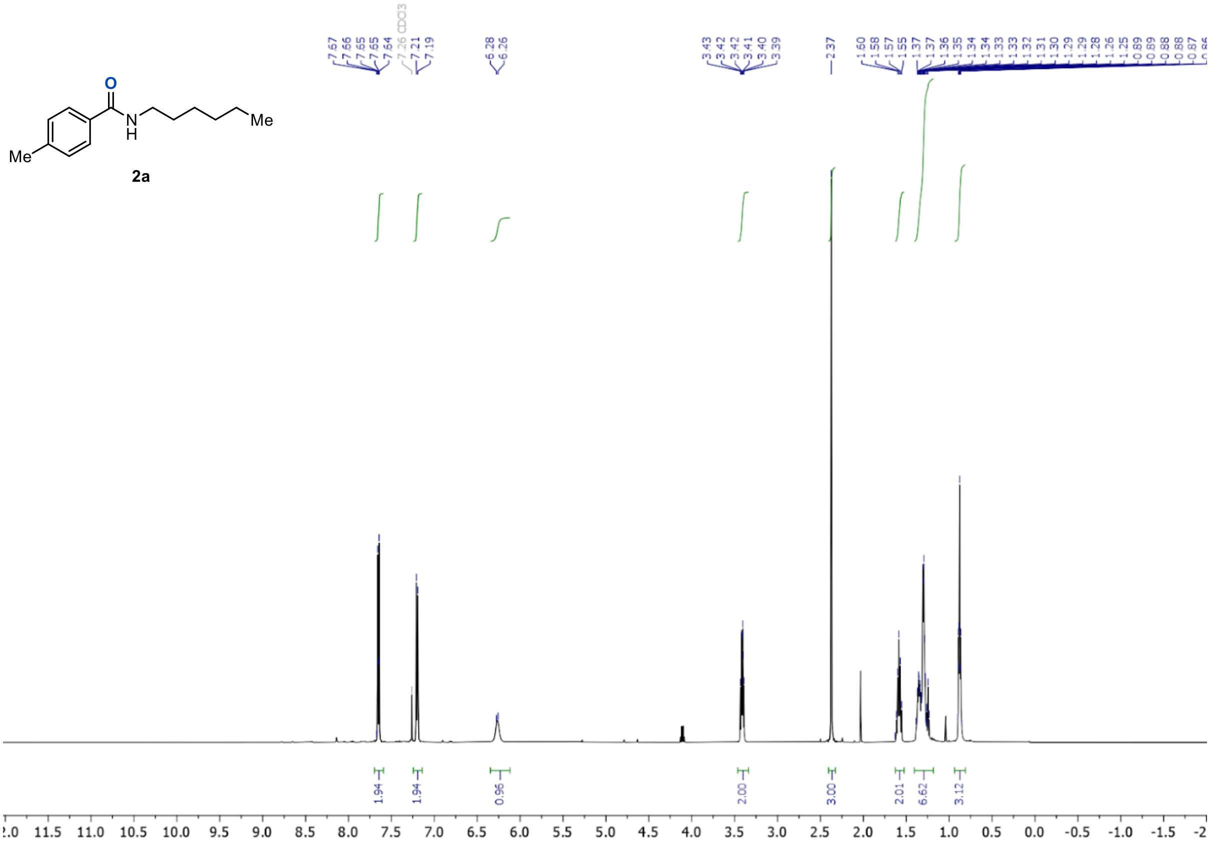

^{13}C{^{1}H} NMR (126 MHz, $CDCl_3$) of *N*-hexyl-4-methylbenzamide (2a)

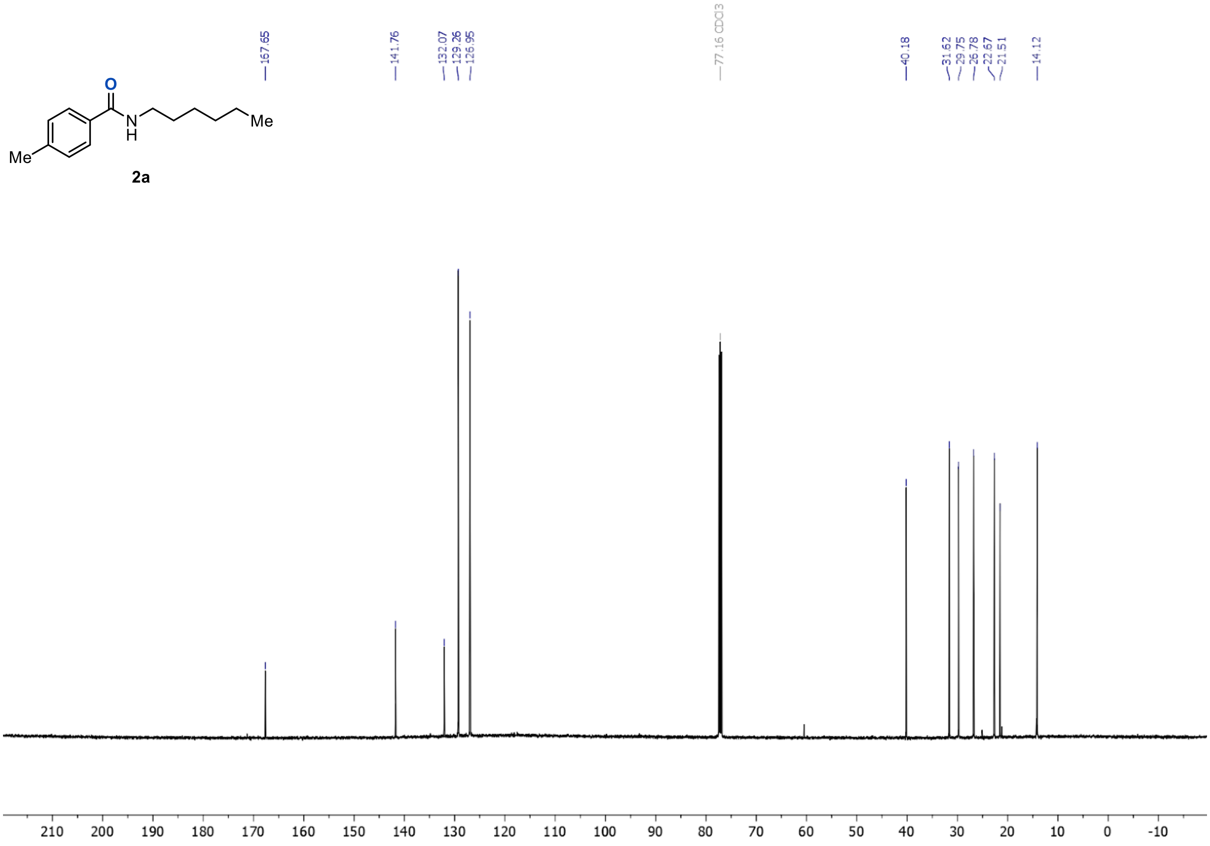

^{1}H NMR (500 MHz, CDCl$_3$) of *N*-hexyl-4-methoxybenzamide (2b)

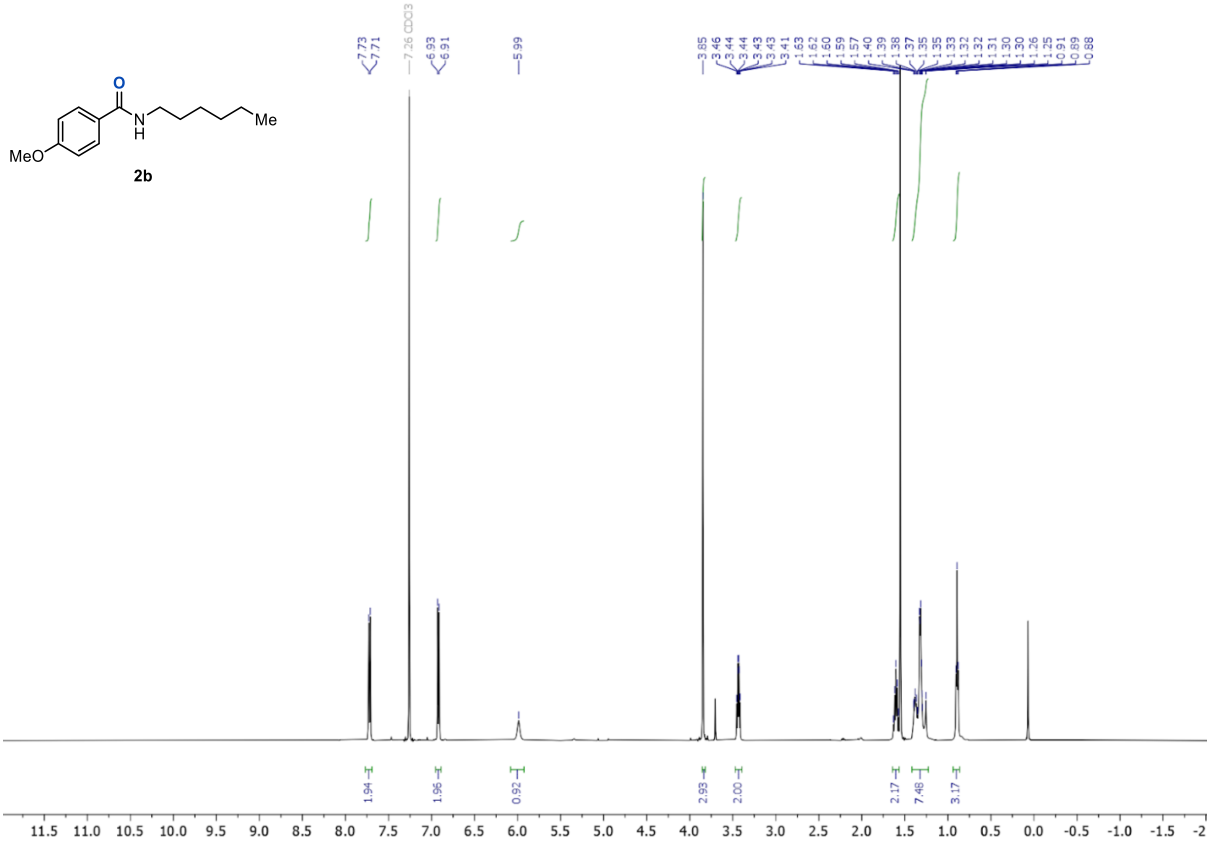

^{13}C{^{1}H} NMR (126 MHz, CDCl$_3$) of *N*-hexyl-4-methoxybenzamide (2b)

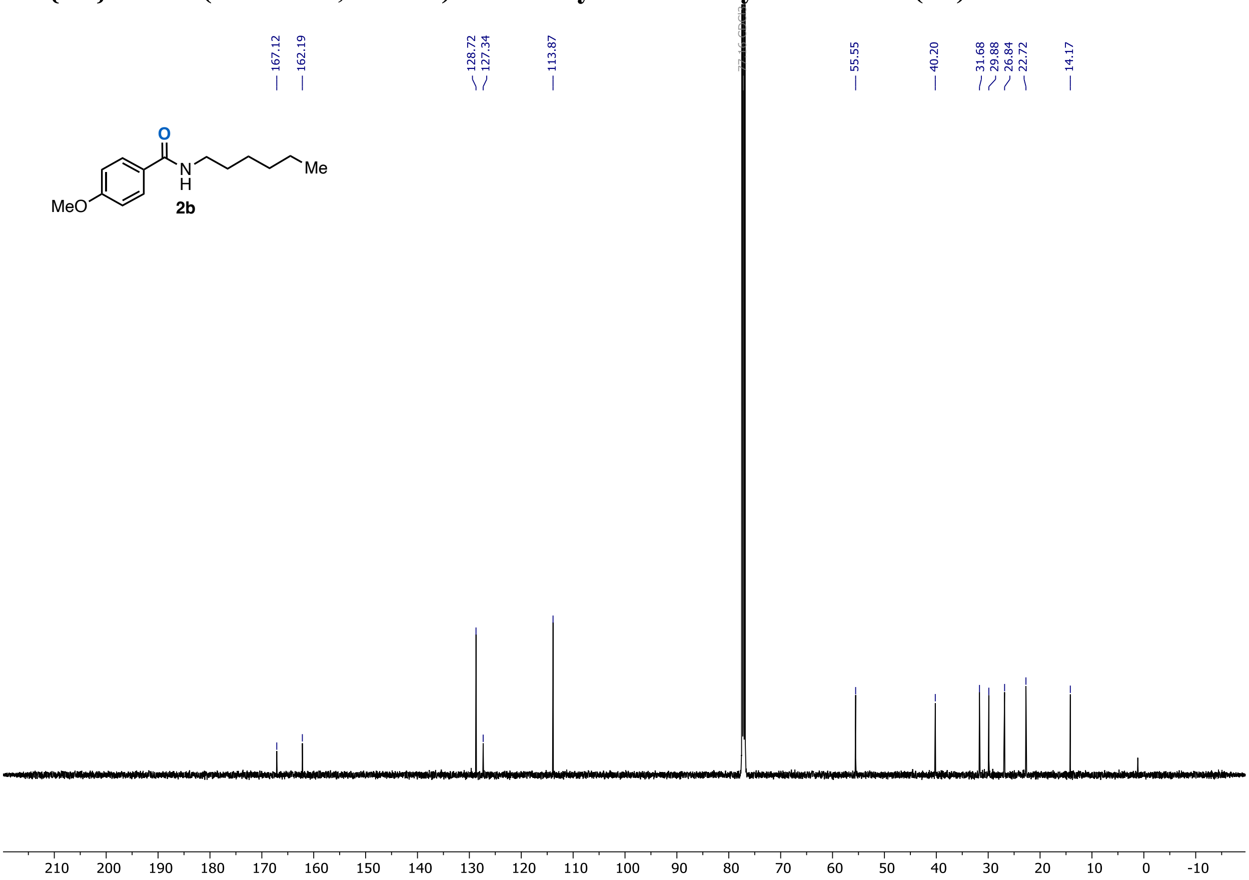

^{1}H NMR (500 MHz, $CDCl_3$) of 4-(tert-butyl)-*N*-hexylbenzamide (2c)

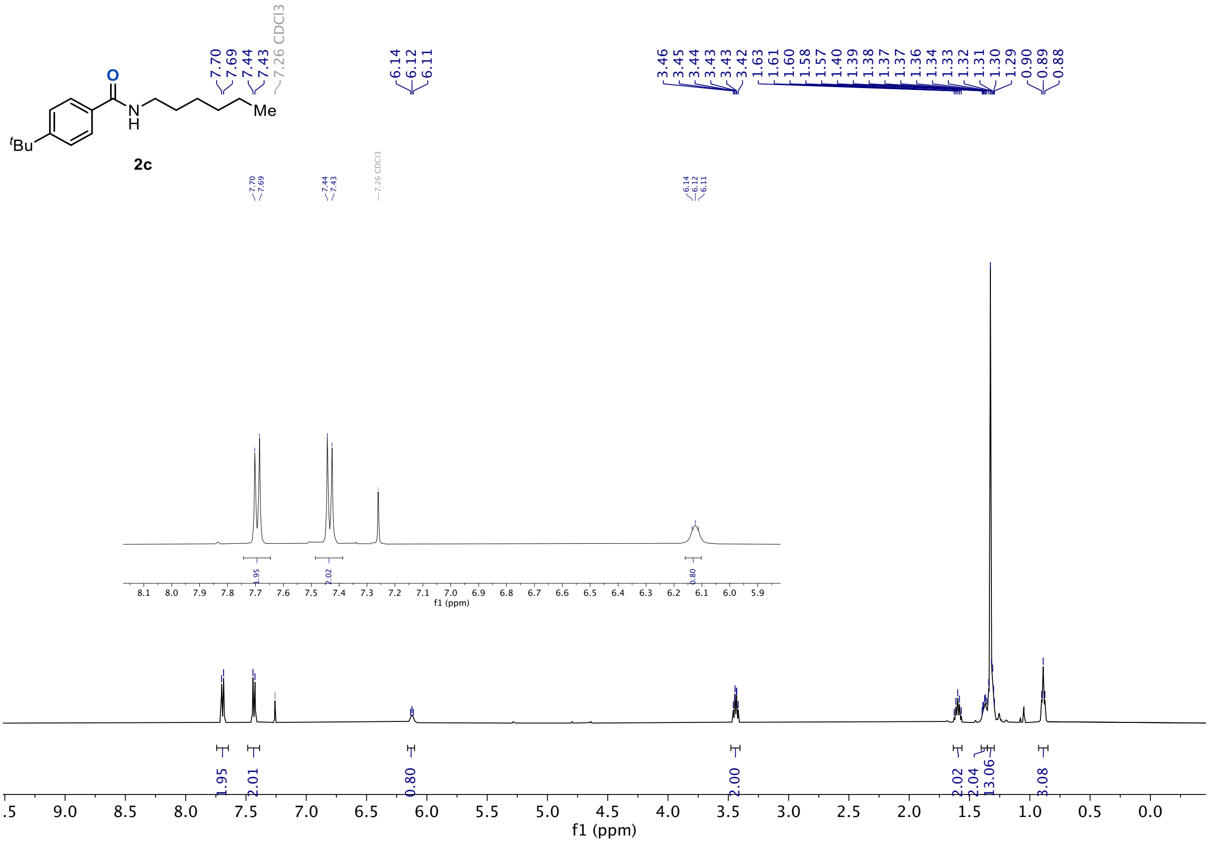

^{13}C{^{1}H} NMR (126 MHz, $CDCl_3$) of 4-(tert-butyl)-*N*-hexylbenzamide (2c)

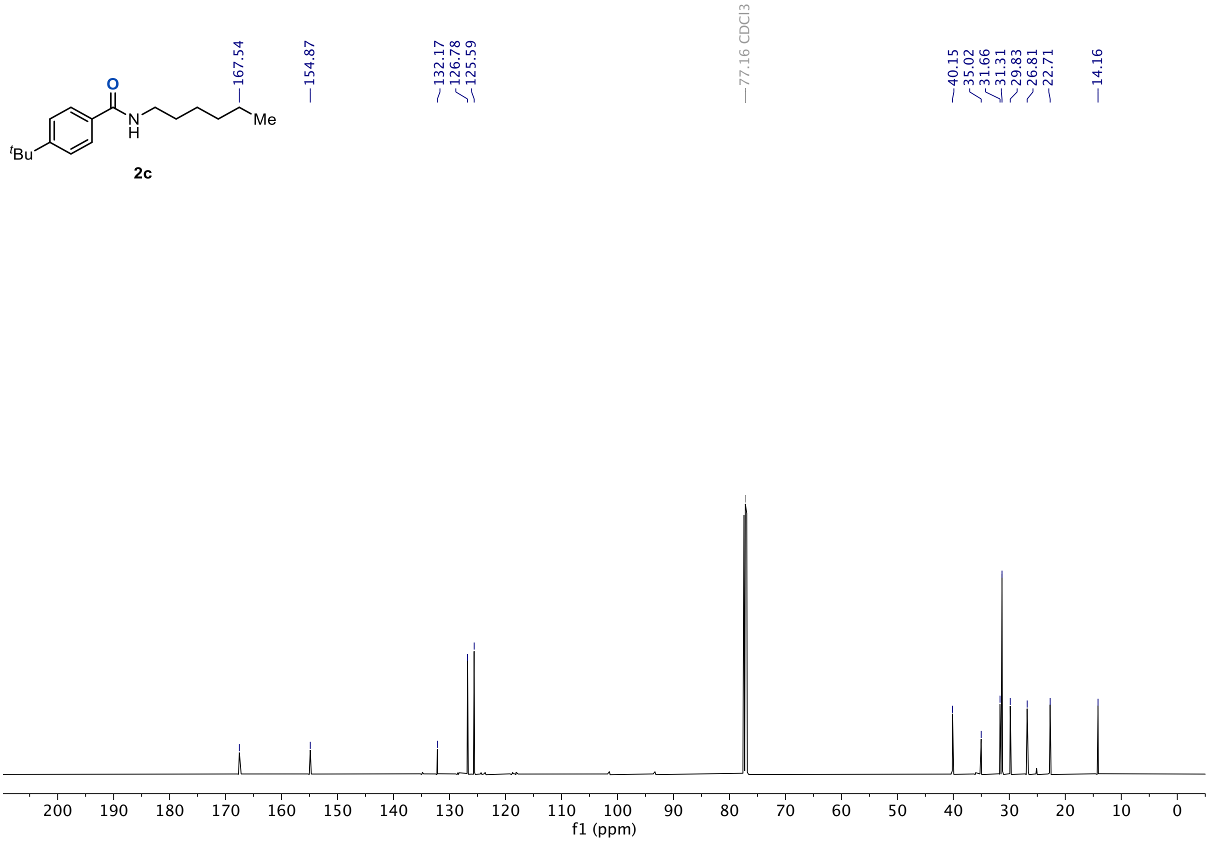

^{1}H NMR (500 MHz, CDCl$_3$) of *N*-hexylbenzamide (2d)

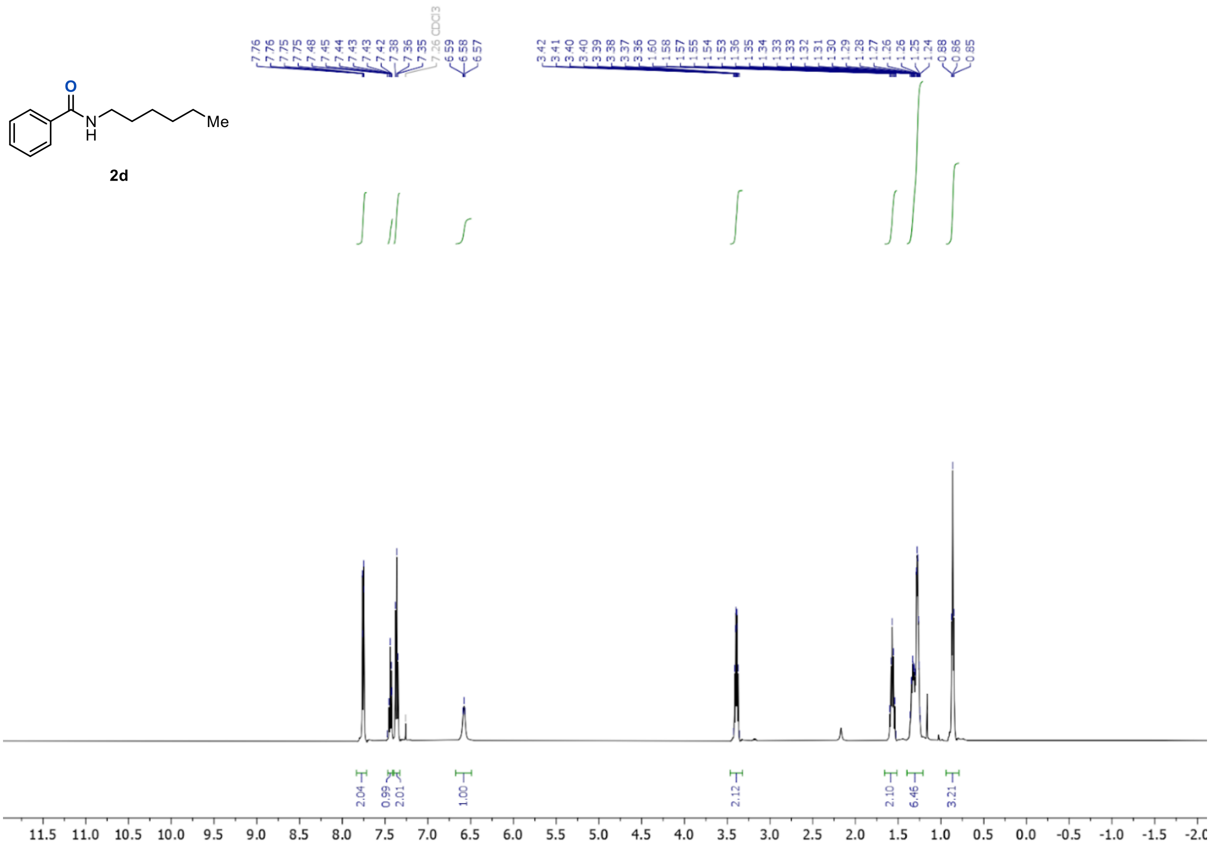

^{13}C{^{1}H} NMR (126 MHz, CDCl$_3$) of N-hexylbenzamide (2d)

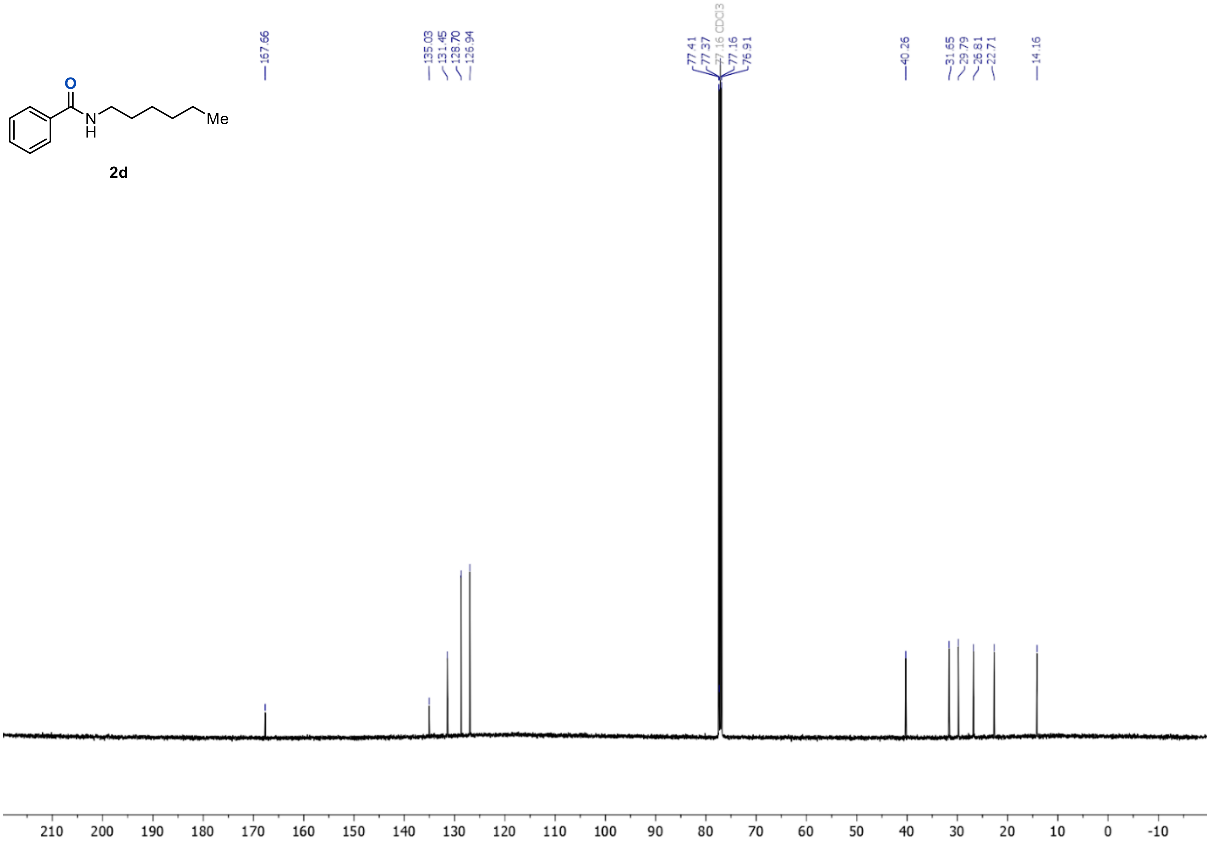

^{1}H NMR (500 MHz, $CDCl_3$) of 4-cyano-N-hexylbenzamide (2e)

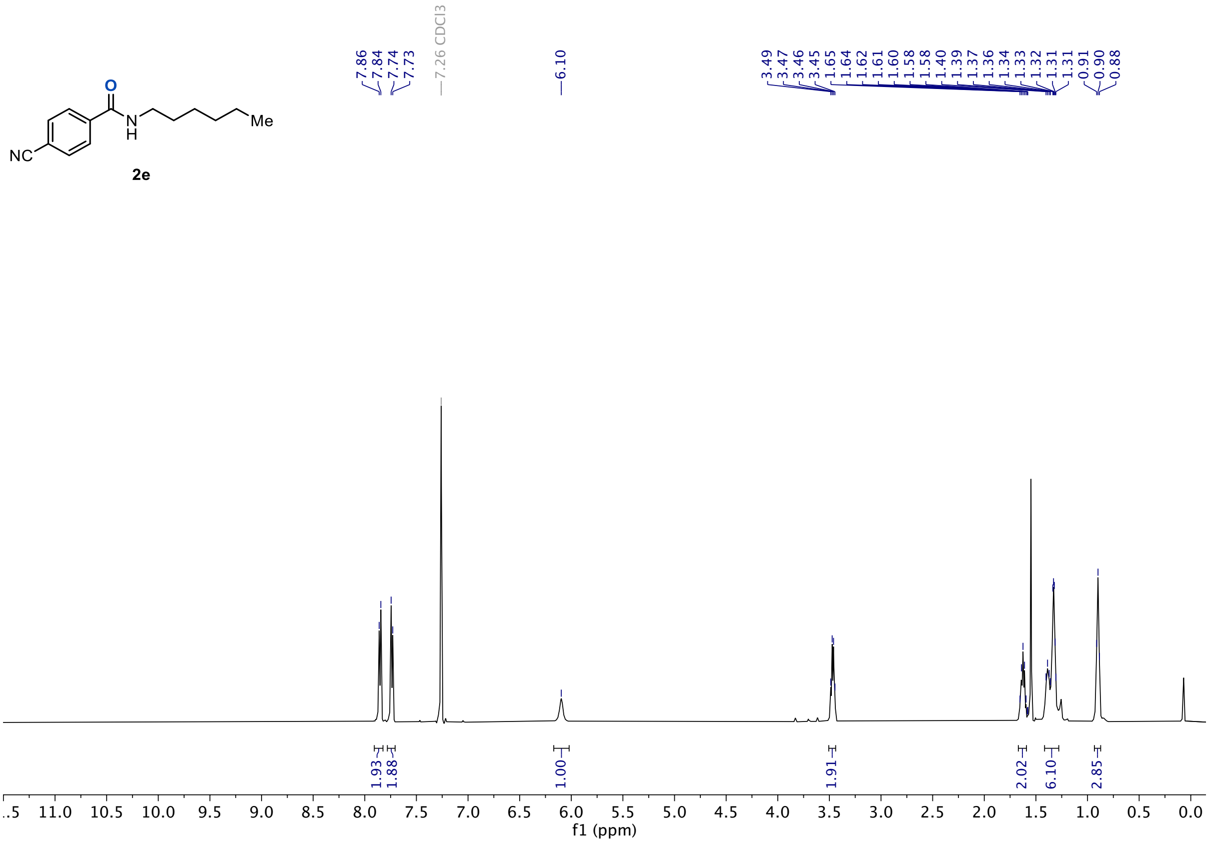

^{13}C{H} NMR (126 MHz, $CDCl_3$) of 4-cyano-N-hexylbenzamide (2e)

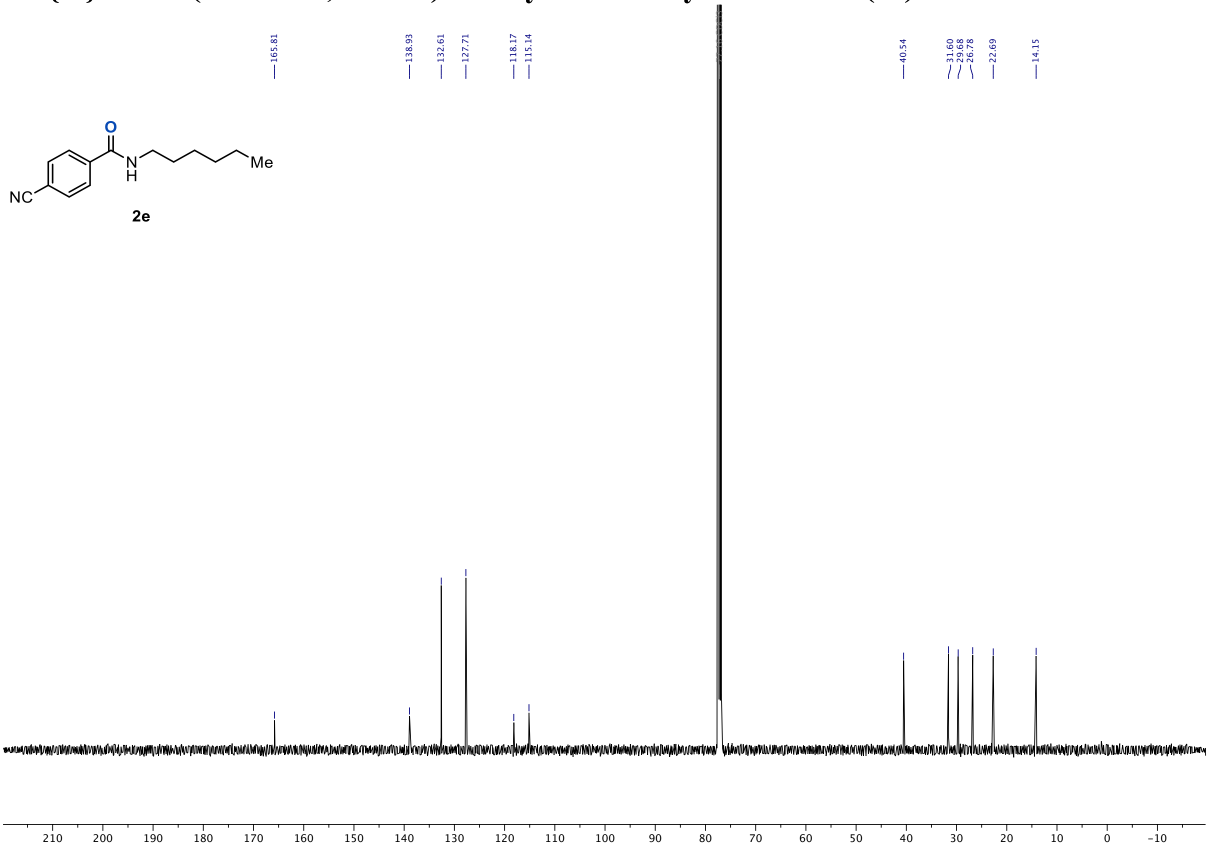

¹H NMR (500 MHz, CDCl₃) of N-hexyl-[1,1'-biphenyl]-4-carboxamide (2f)

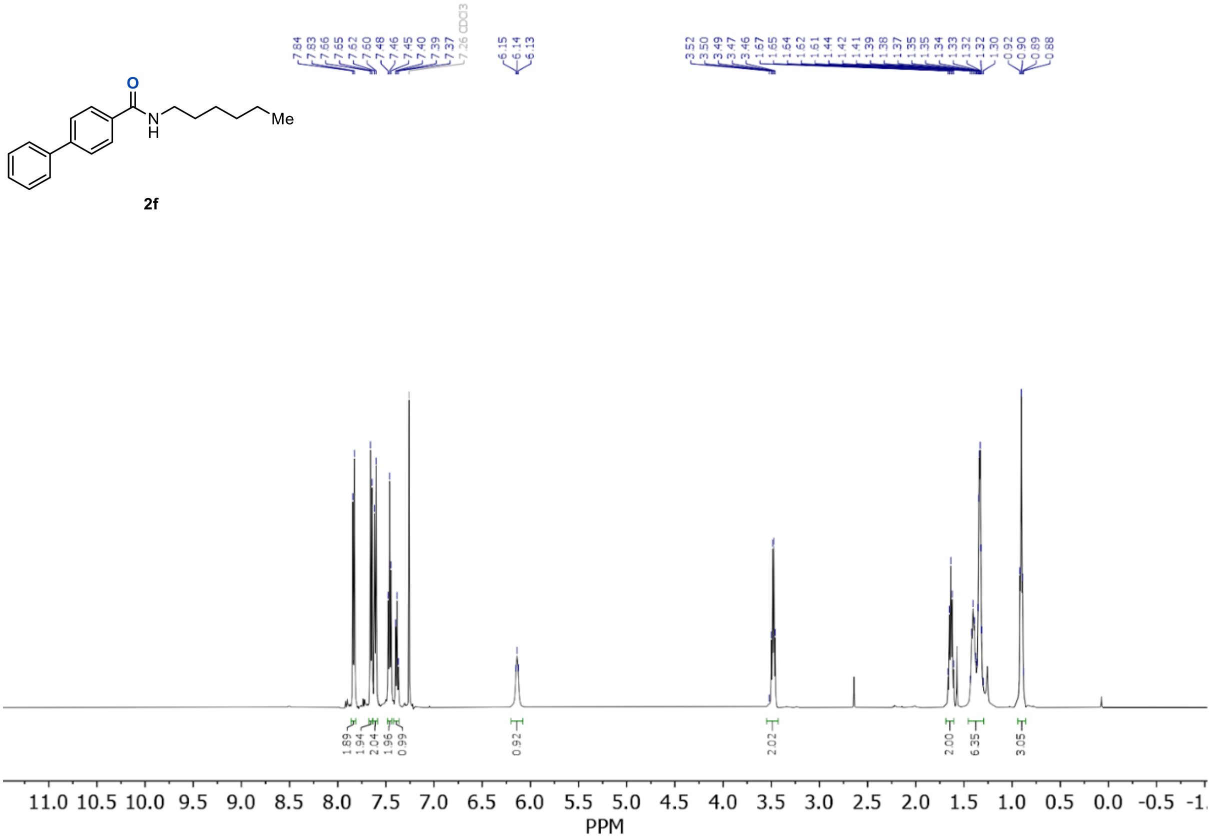

¹³C{¹H} NMR (101 MHz, CDCl₃) of N-hexyl-[1,1'-biphenyl]-4-carboxamide (2f)

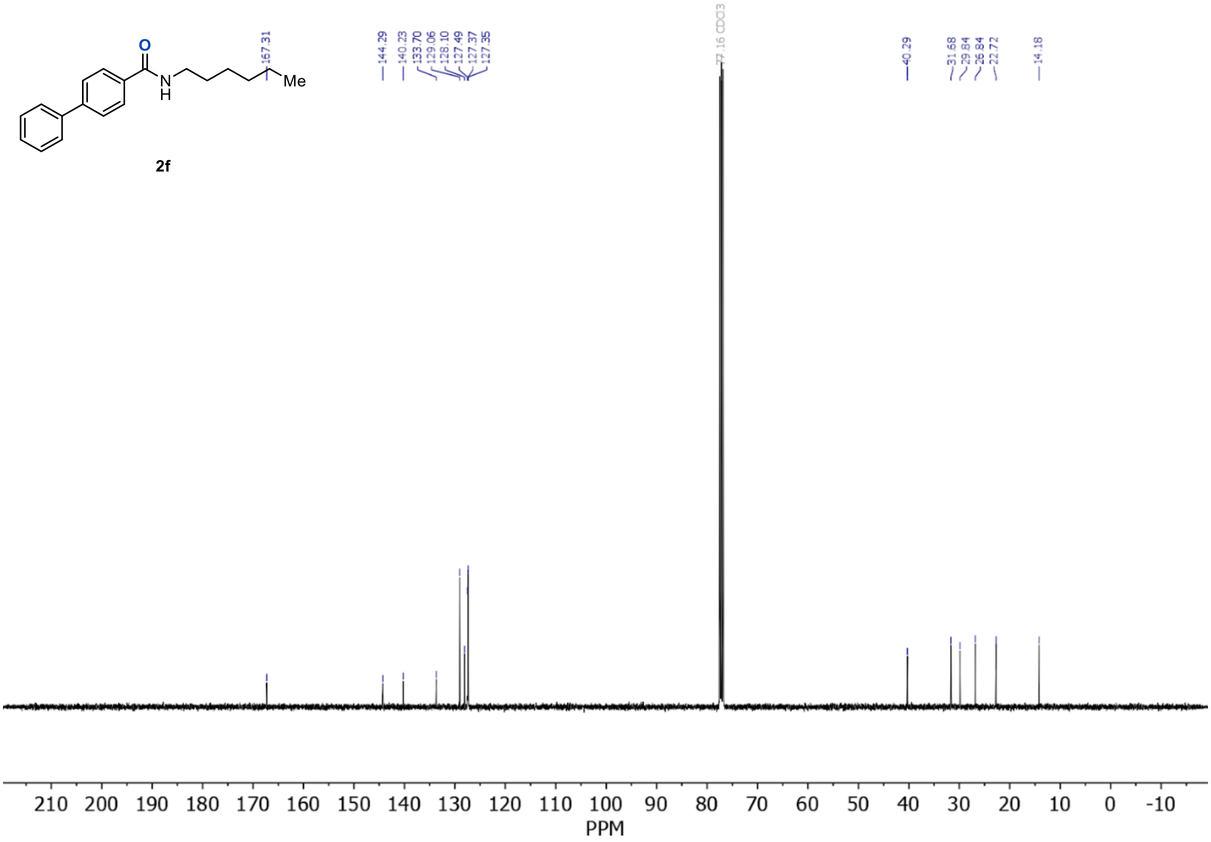

^{1}H NMR (500 MHz, $CDCl_3$) of N-hexyl-3-methylbenzamide (2g)

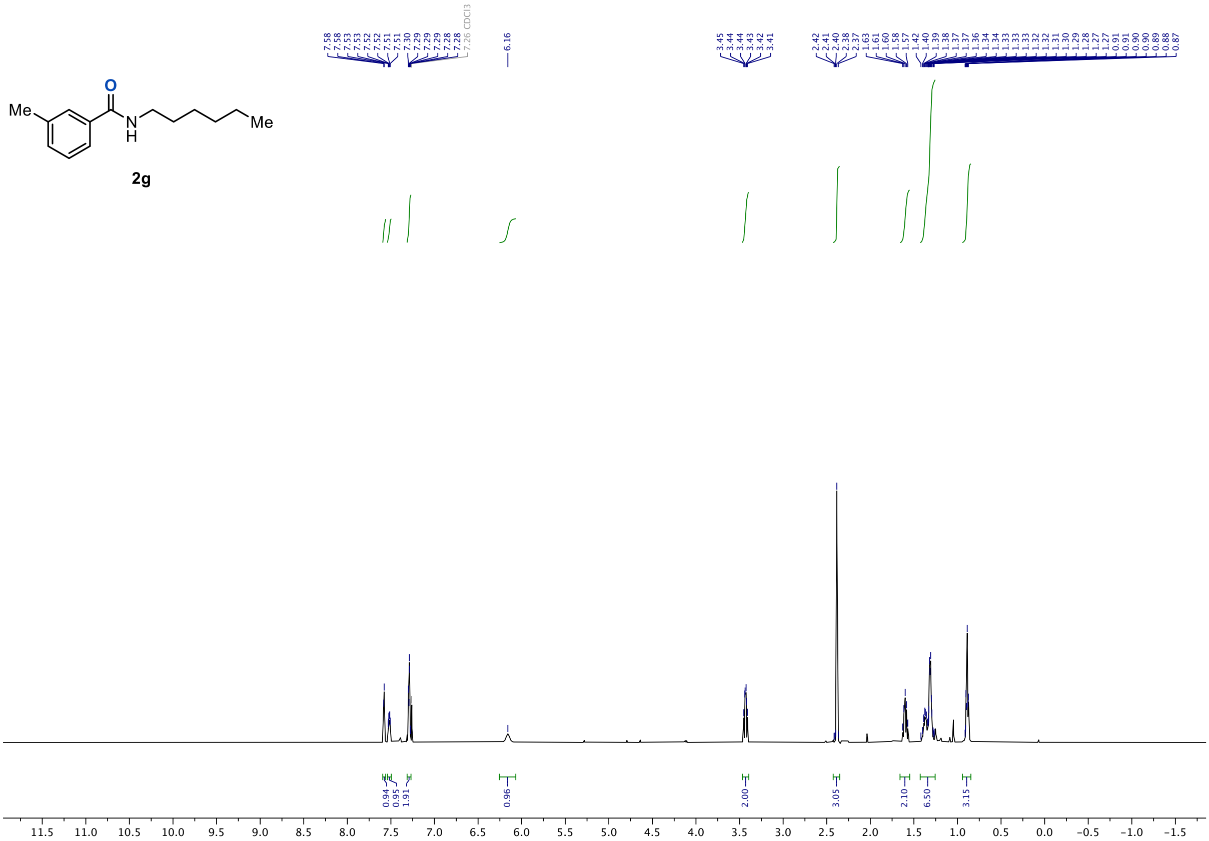

^{13}C{^{1}H} NMR (126 MHz, $CDCl_3$) of N-hexyl-3-methylbenzamide (2g)

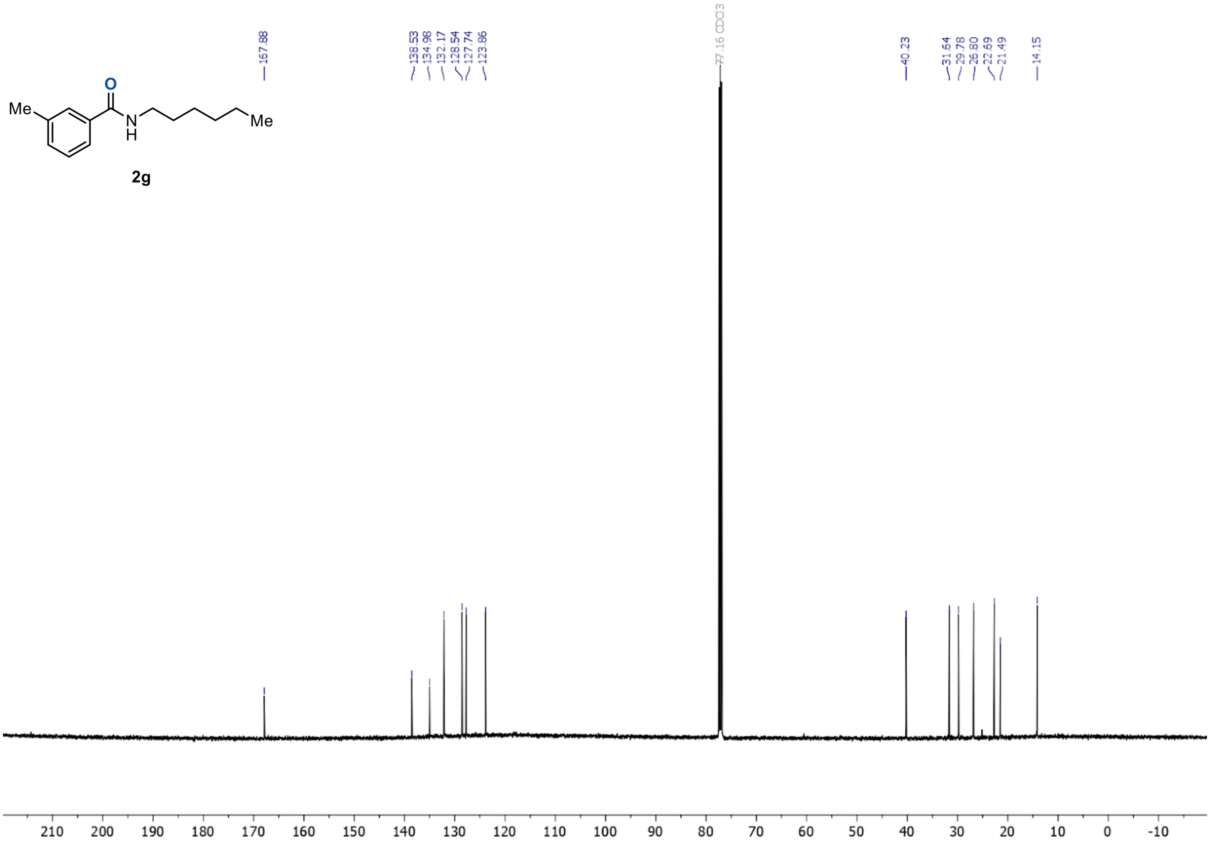

^{1}H NMR (500 MHz, $CDCl_3$) of 3-bromo-N-hexylbenzamide (2h)

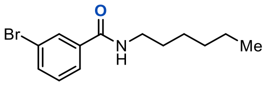

2h

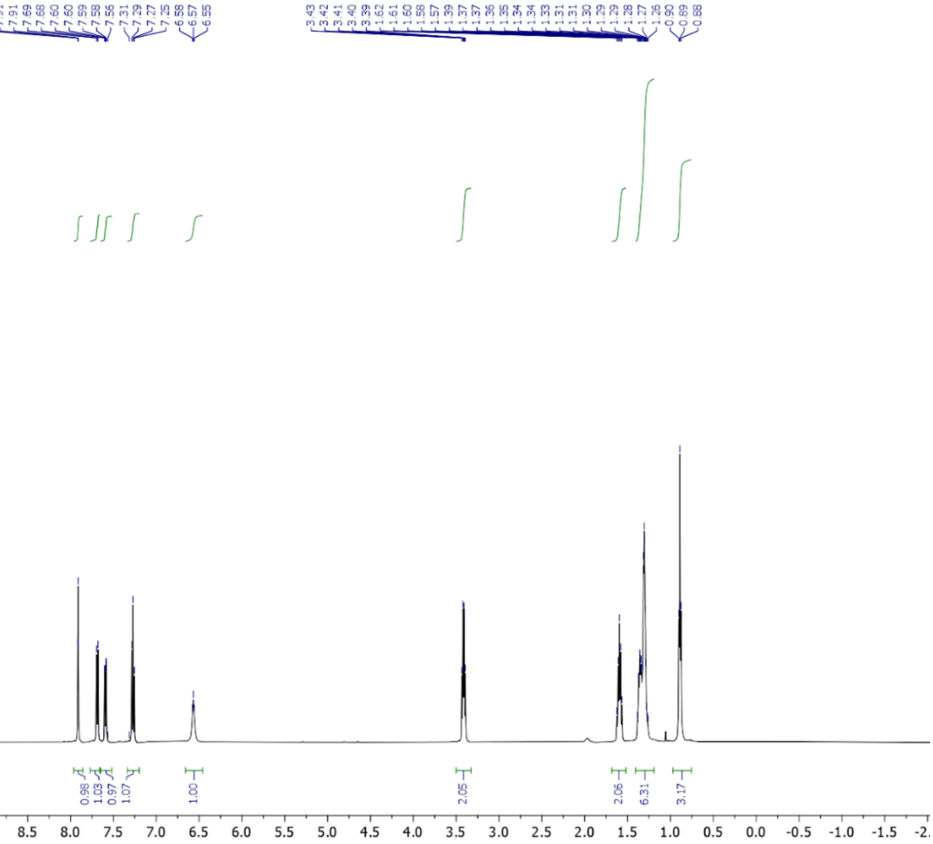

^{13}C{^{1}H} NMR (126 MHz, $CDCl_3$) of 3-bromo-N-hexylbenzamide (2h)

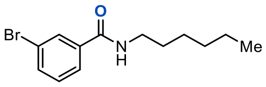

2h

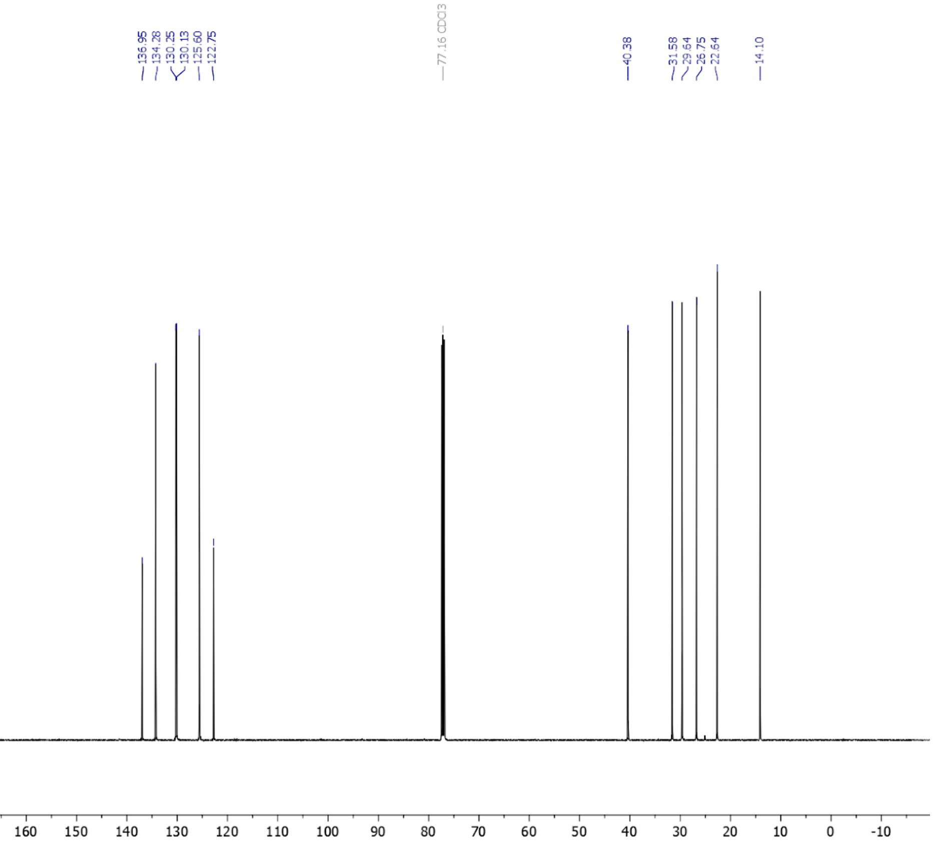

^{1}H NMR (500 MHz, $CDCl_3$) of 2-bromo-N-hexylbenzamide (2i)

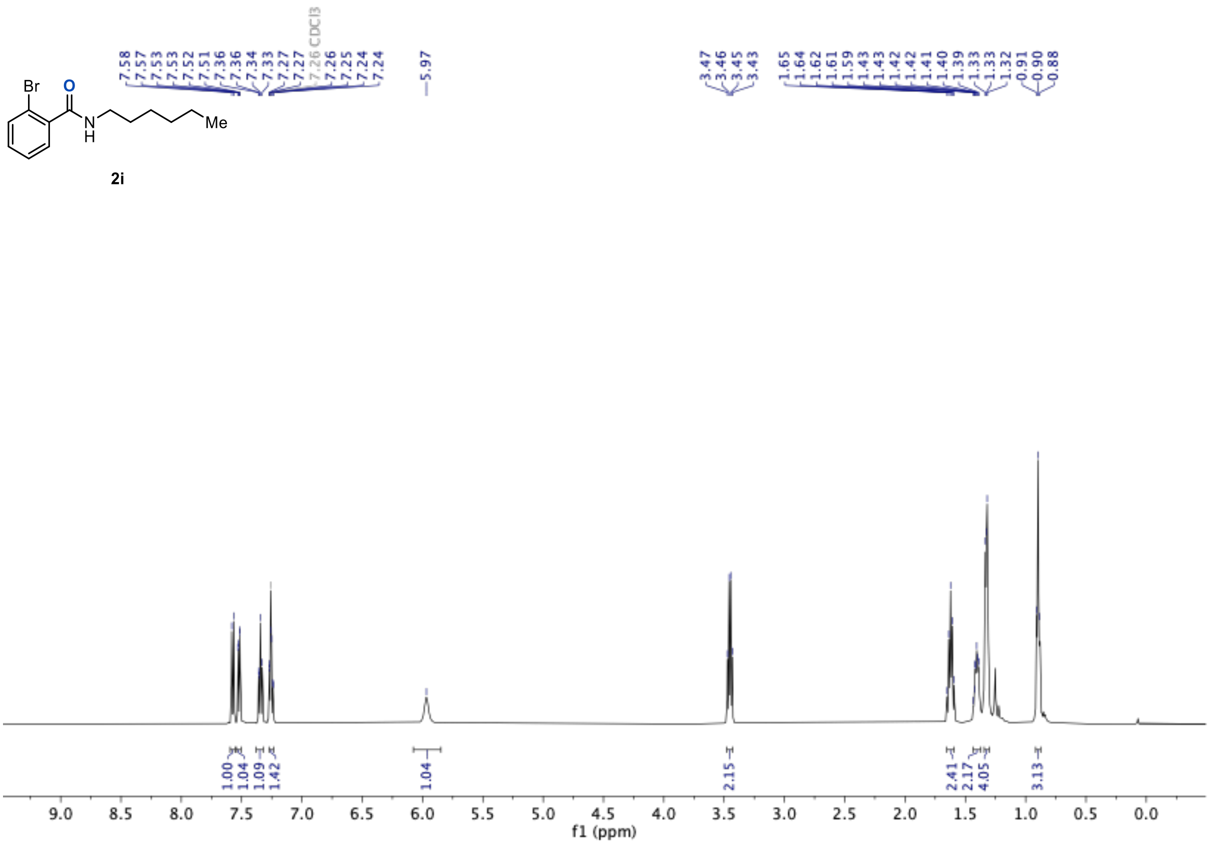

$^{13}C\{^{1}H\}$ NMR (126 MHz, $CDCl_3$) of 2-bromo-N-hexylbenzamide (2i)

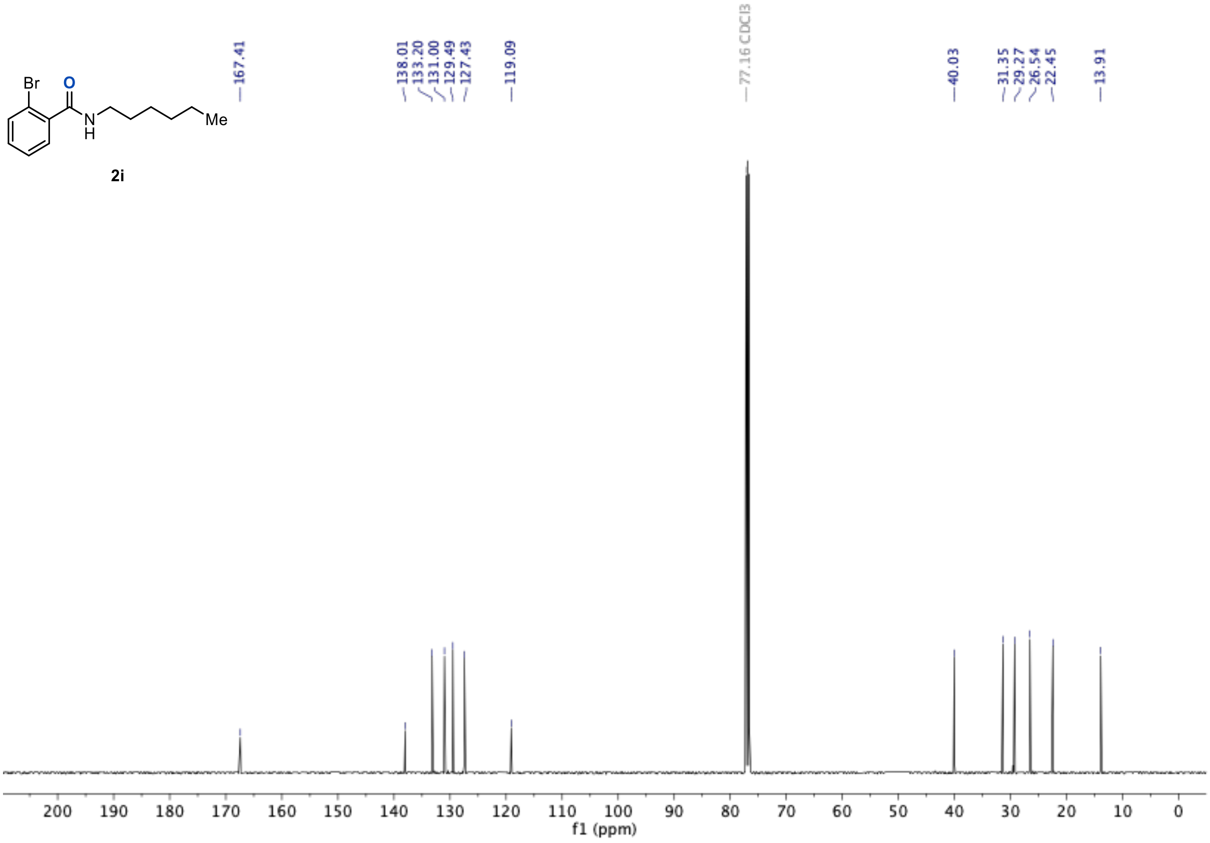

^{1}H NMR (500 MHz, CDCl$_3$) of N-hexylbenzamide (2j)

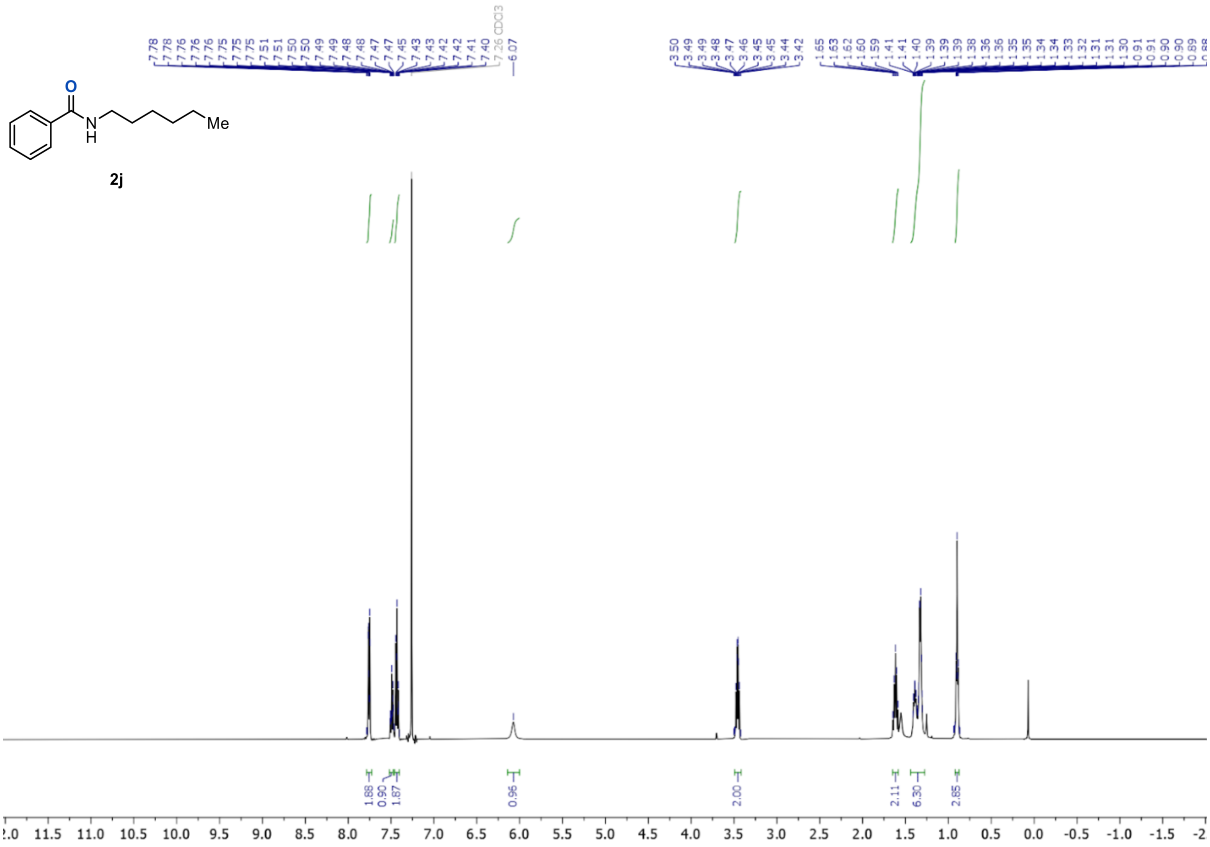

^{13}C{^{1}H} NMR (126 MHz, CDCl$_3$) of N-hexylbenzamide (2j)

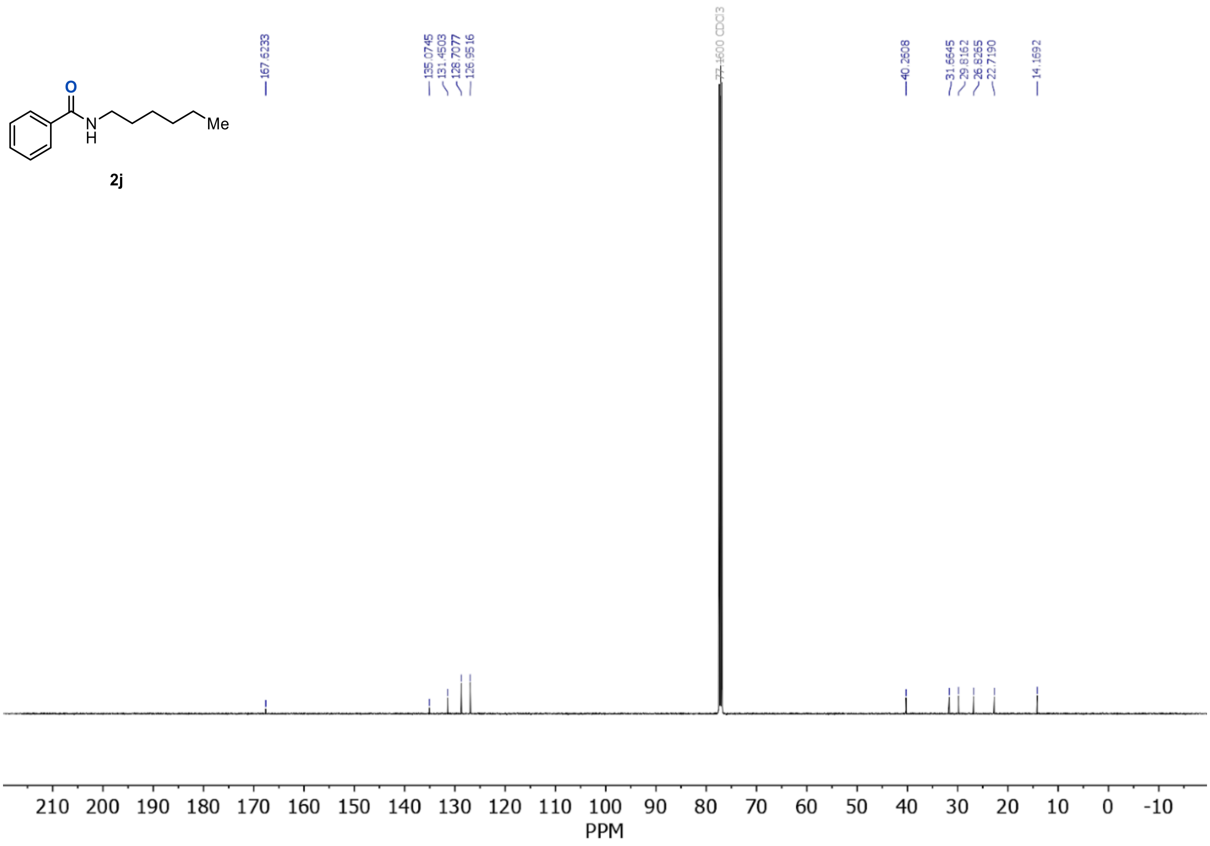

^{1}H NMR (500 MHz, CD_3OD) of 3-(hexylcarbamoyl)benzoic acid (2k)

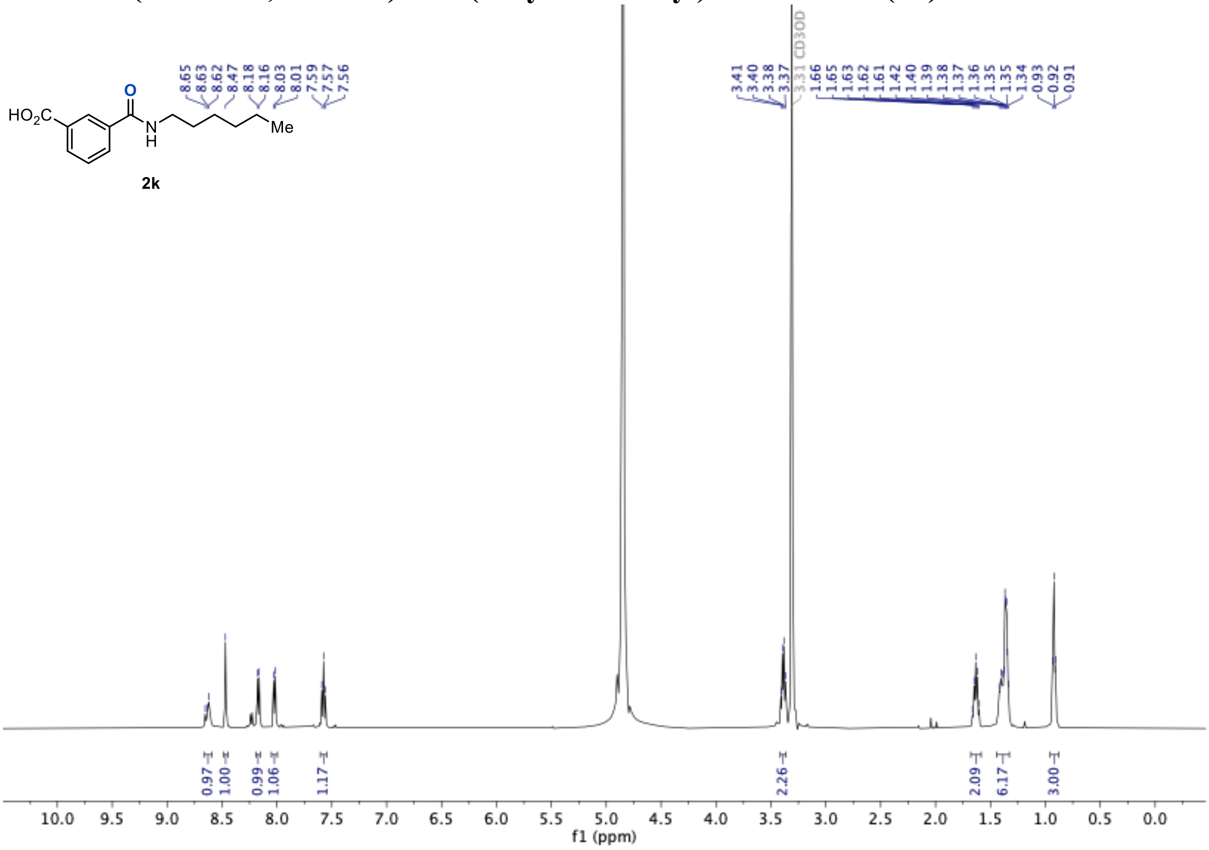

^{13}C{^{1}H} NMR (126 MHz, CD_3OD) of 3-(hexylcarbamoyl)benzoic acid (2k)

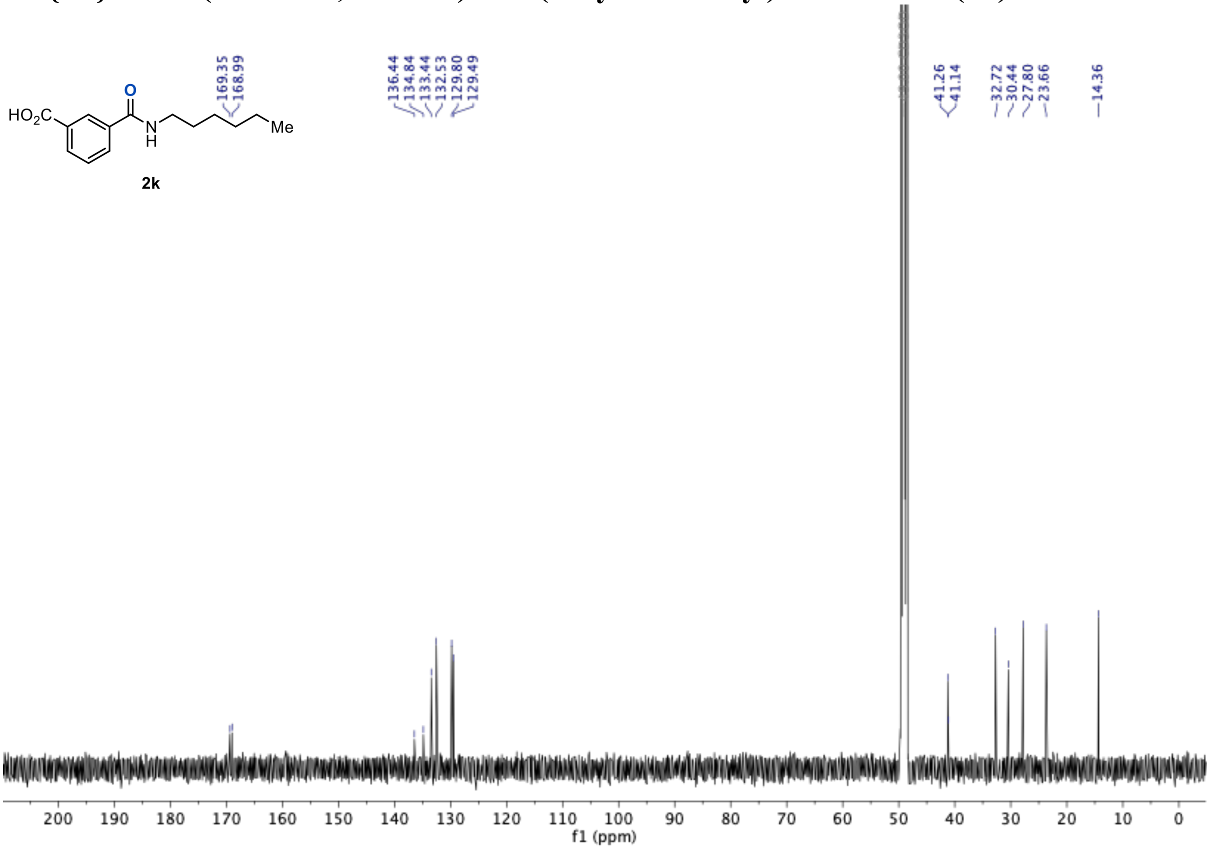

^{1}H NMR (500 MHz, $CDCl_3$) of methyl 3-(hexylcarbamoyl)benzoate (2l)

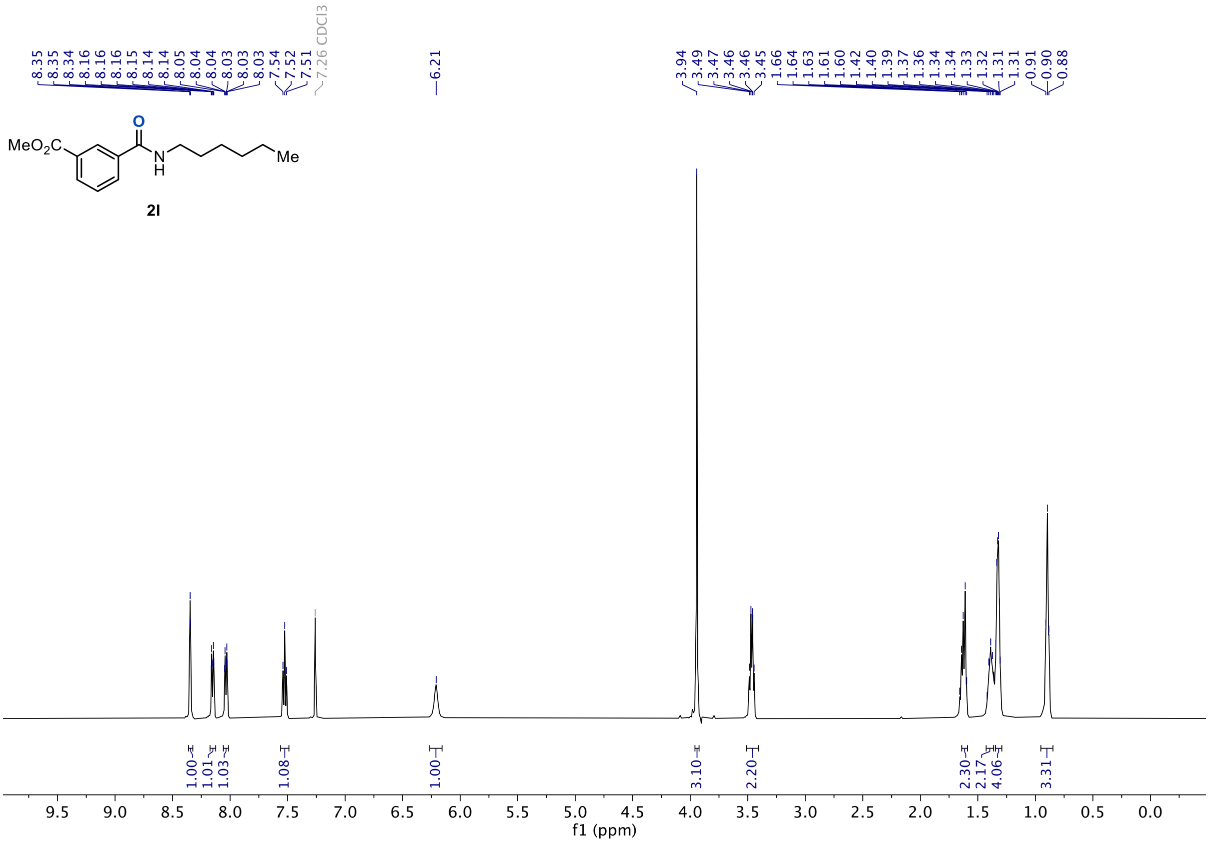

$^{13}C\{^{1}H\}$ NMR (126 MHz, $CDCl_3$) of methyl 3-(hexylcarbamoyl)benzoate (2l)

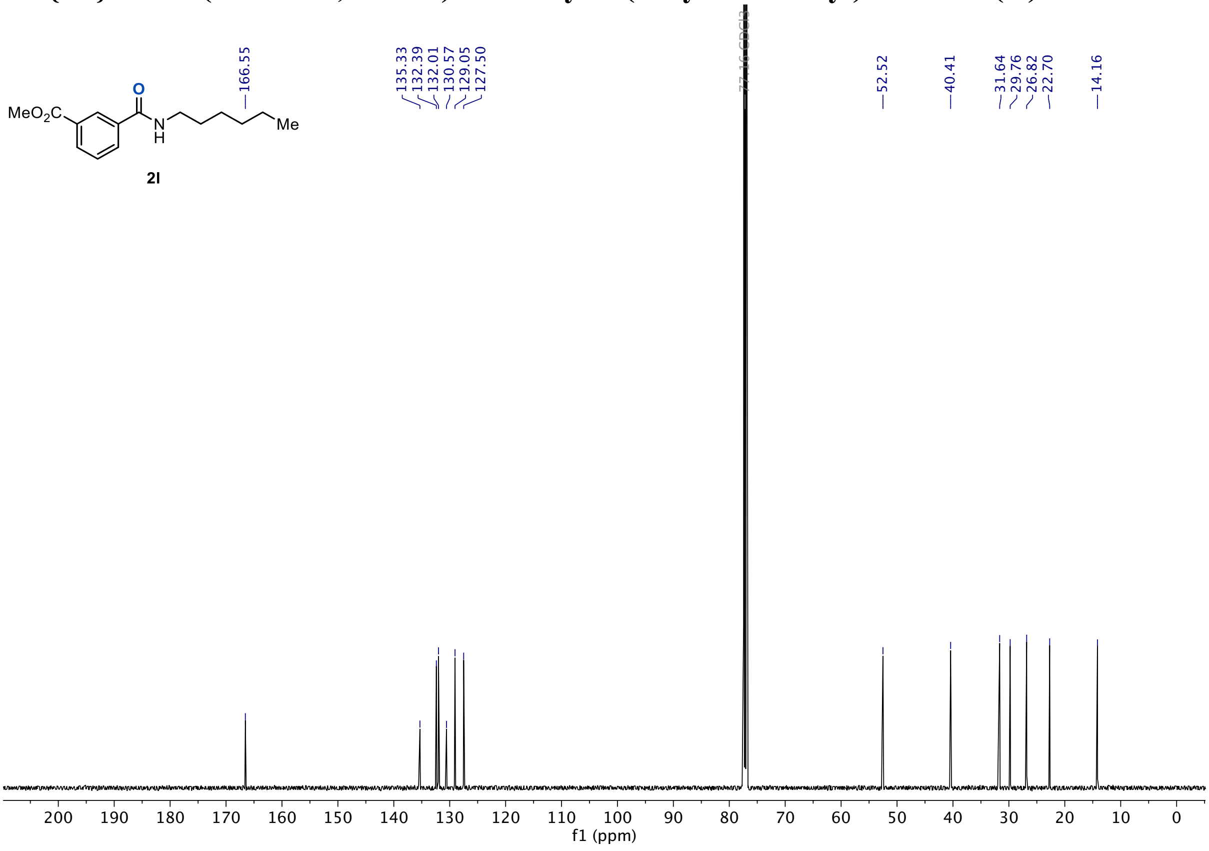

^{1}H NMR (600 MHz, $CDCl_3$) of *N*-hexyl-3-(hydroxymethyl)benzamide (2m)

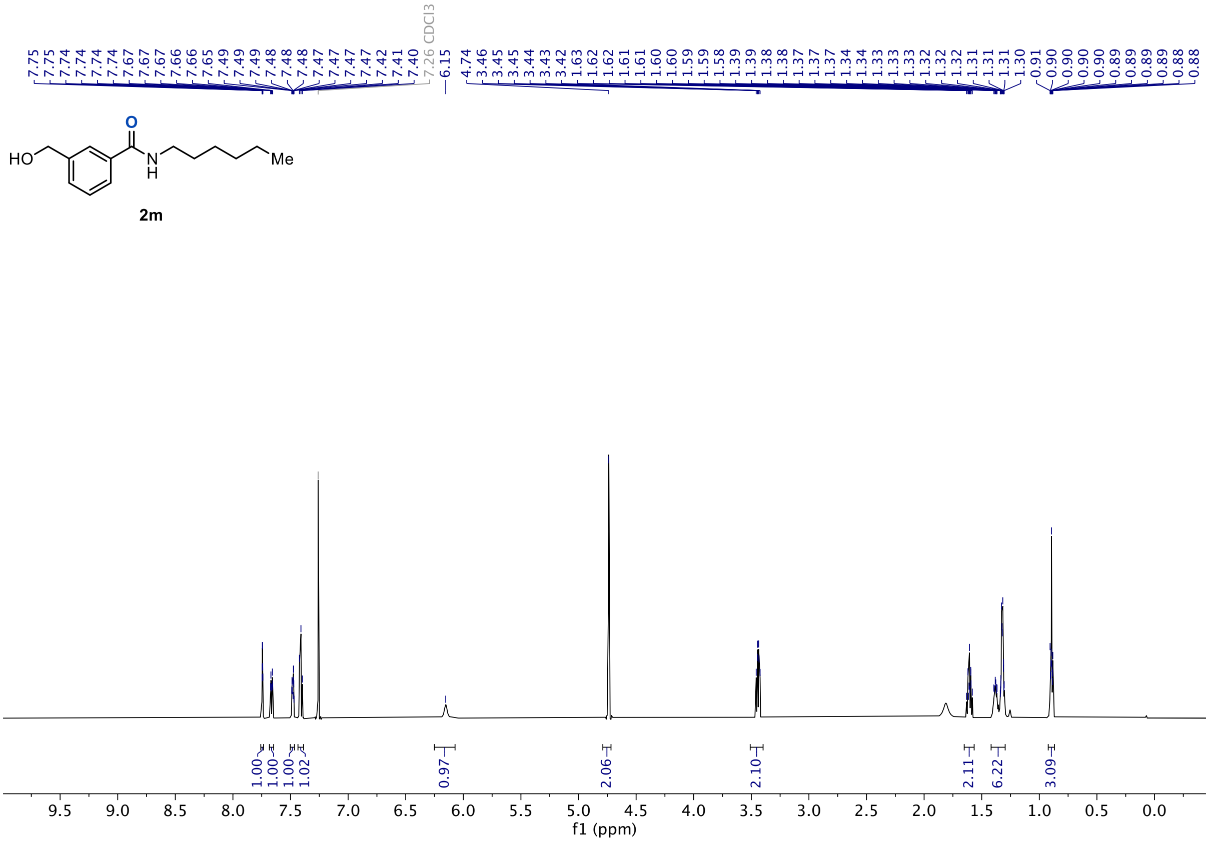

^{13}C{^{1}H} NMR (151 MHz, $CDCl_3$) of *N*-hexyl-3-(hydroxymethyl)benzamide (2m)

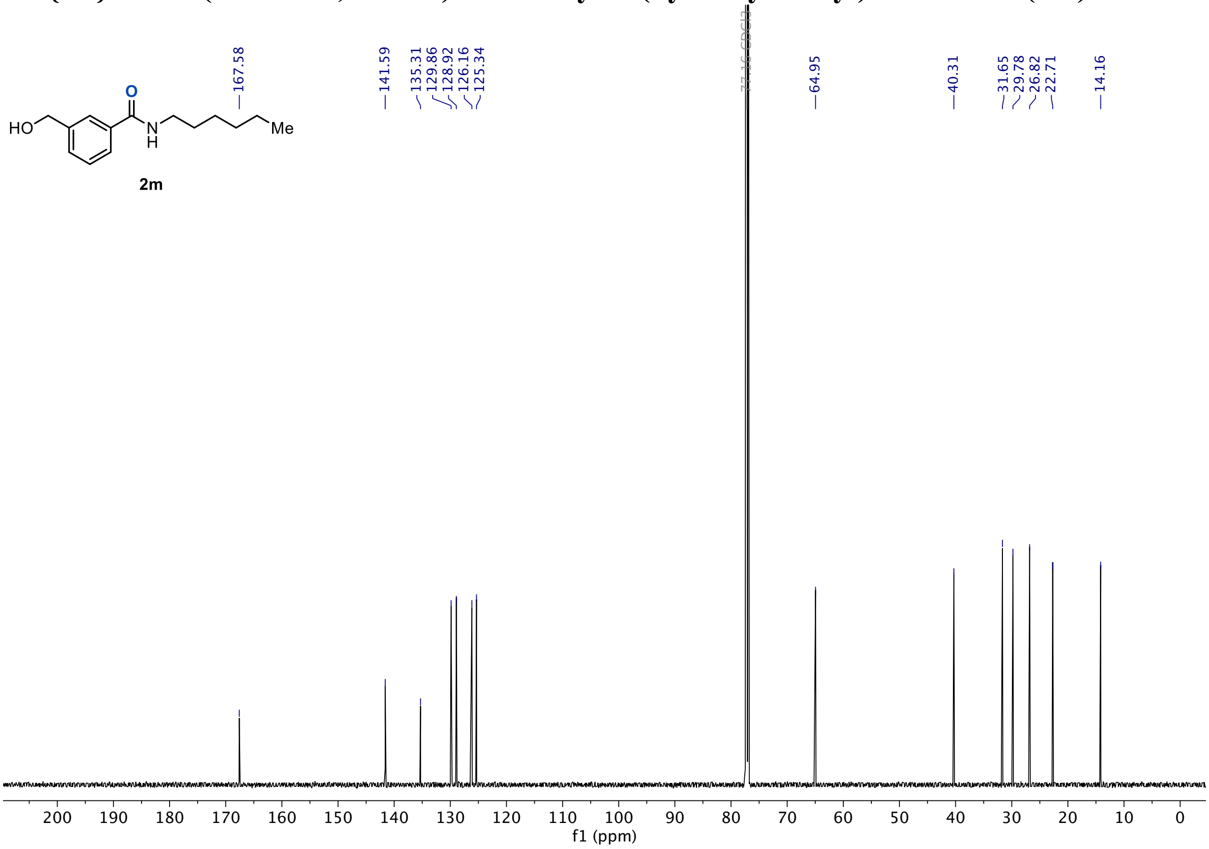

^{1}H NMR (500 MHz, $CDCl_3$) of 2-hexylisoquinolin-1(2H)-one (2n)

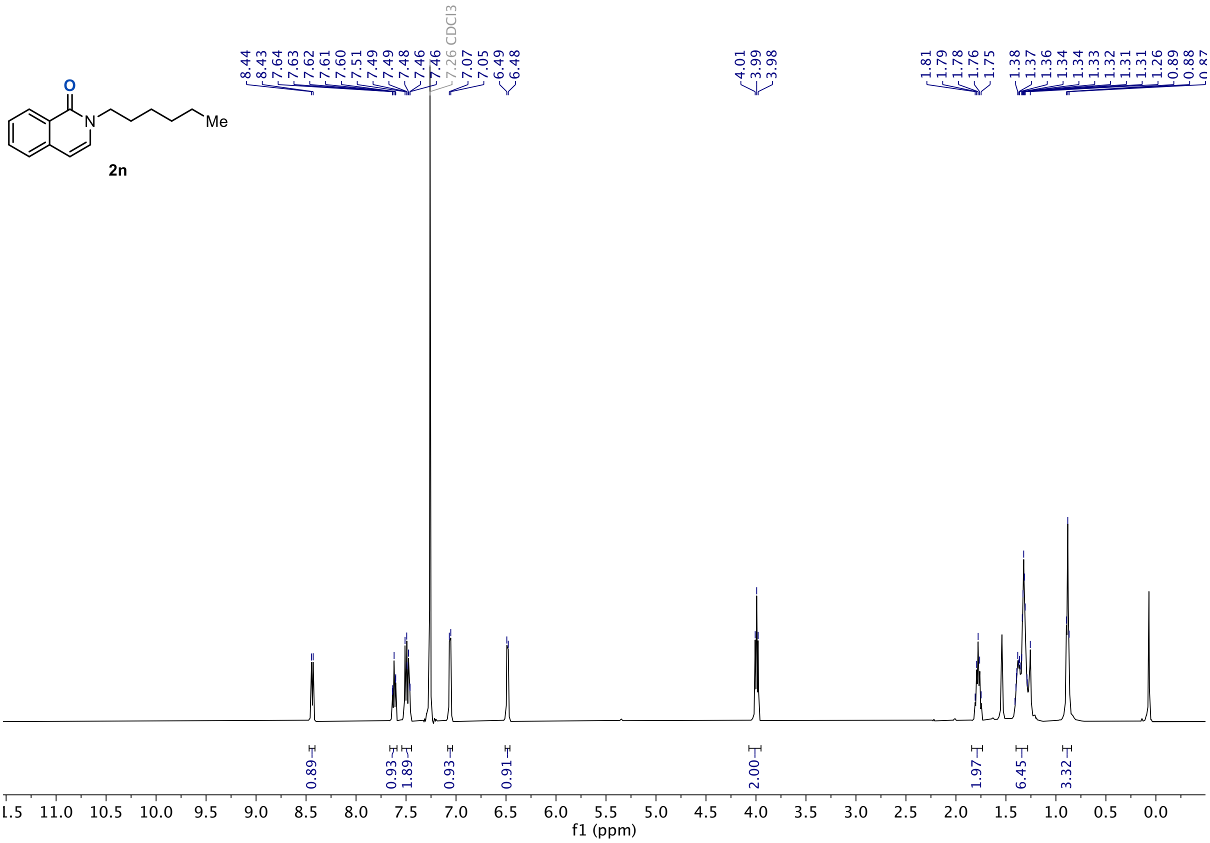

^{13}C{^{1}H} NMR (126 MHz, $CDCl_3$) of 2-hexylisoquinolin-1(2H)-one (2n)

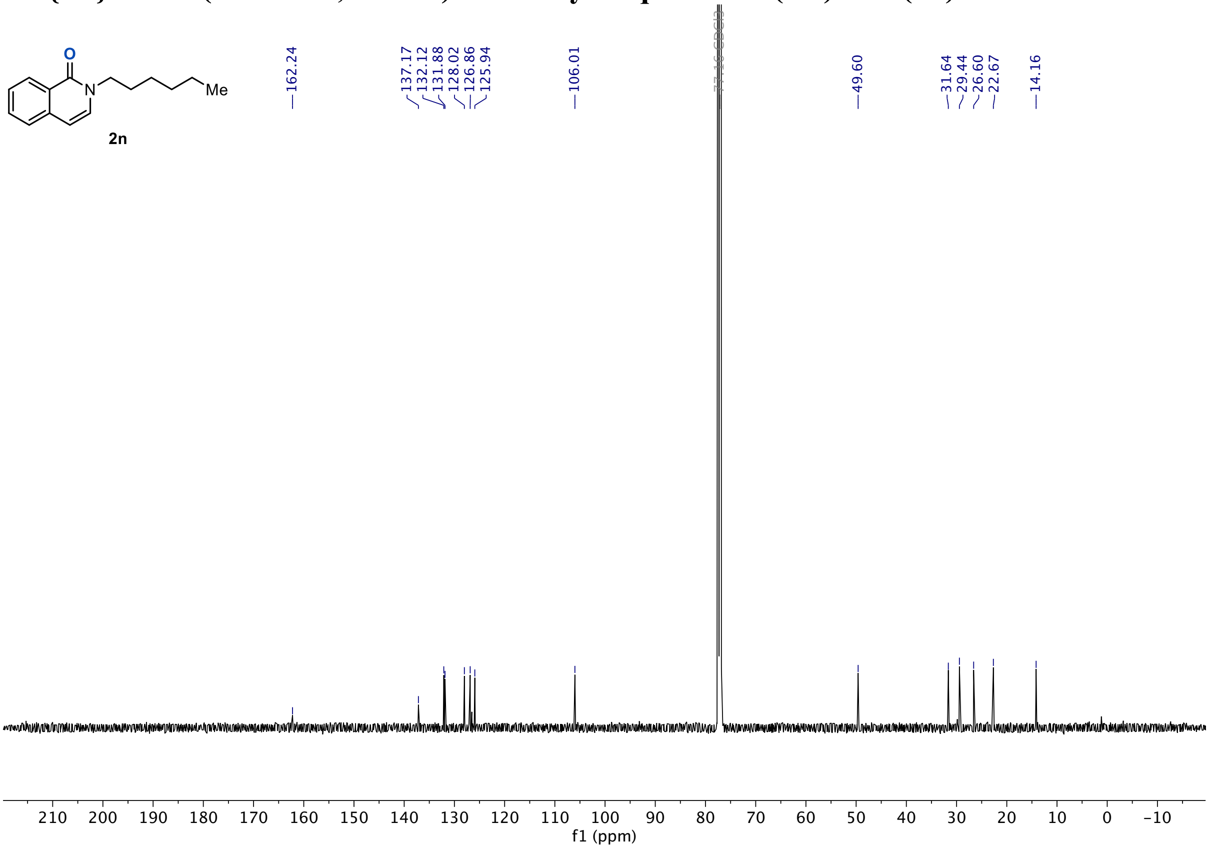

^{1}H NMR (500 MHz, CDCl$_3$) of *N*-hexyl-3-phenylpropanamide (2o)

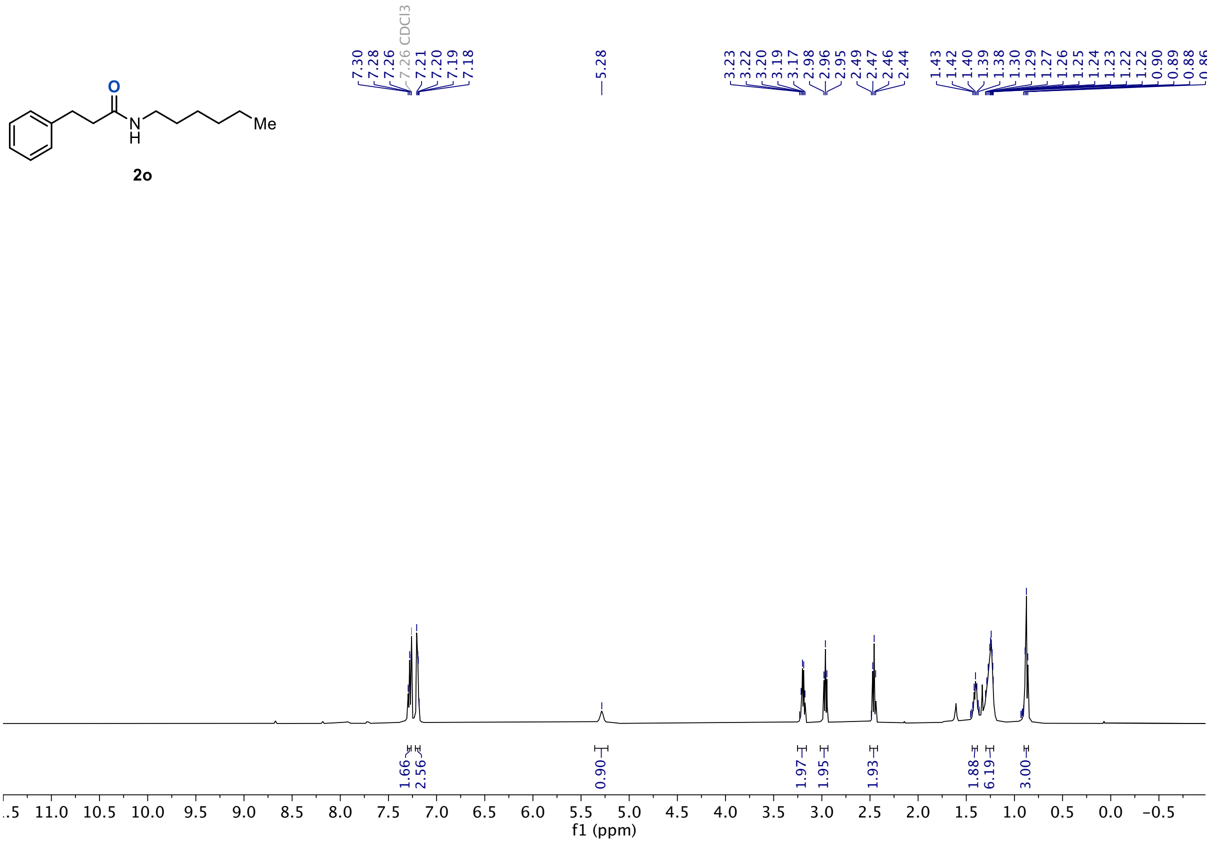

^{13}C{^{1}H} NMR (126 MHz, CDCl$_3$) of *N*-hexyl-3-phenylpropanamide (2o)

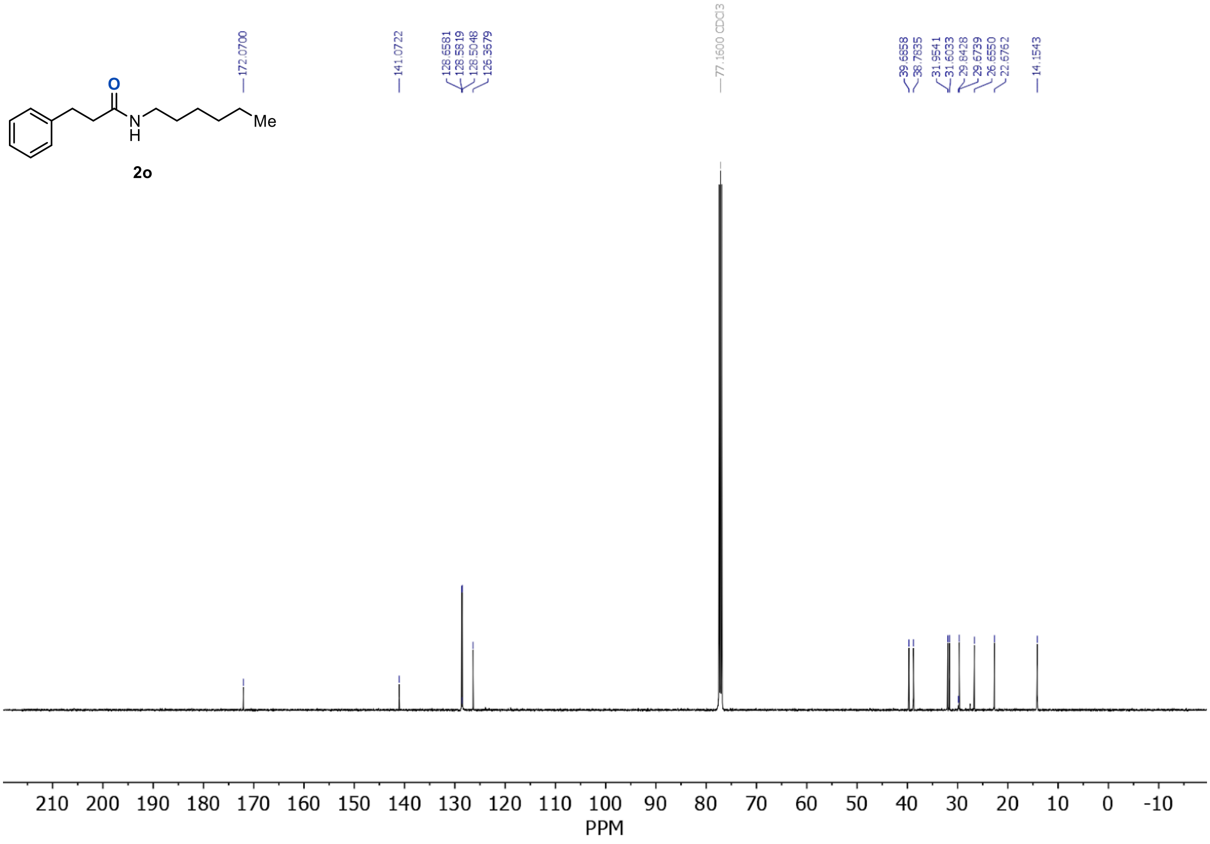

^{1}H NMR (500 MHz, $CDCl_3$) of *N*-hexylheptanamide (2p)

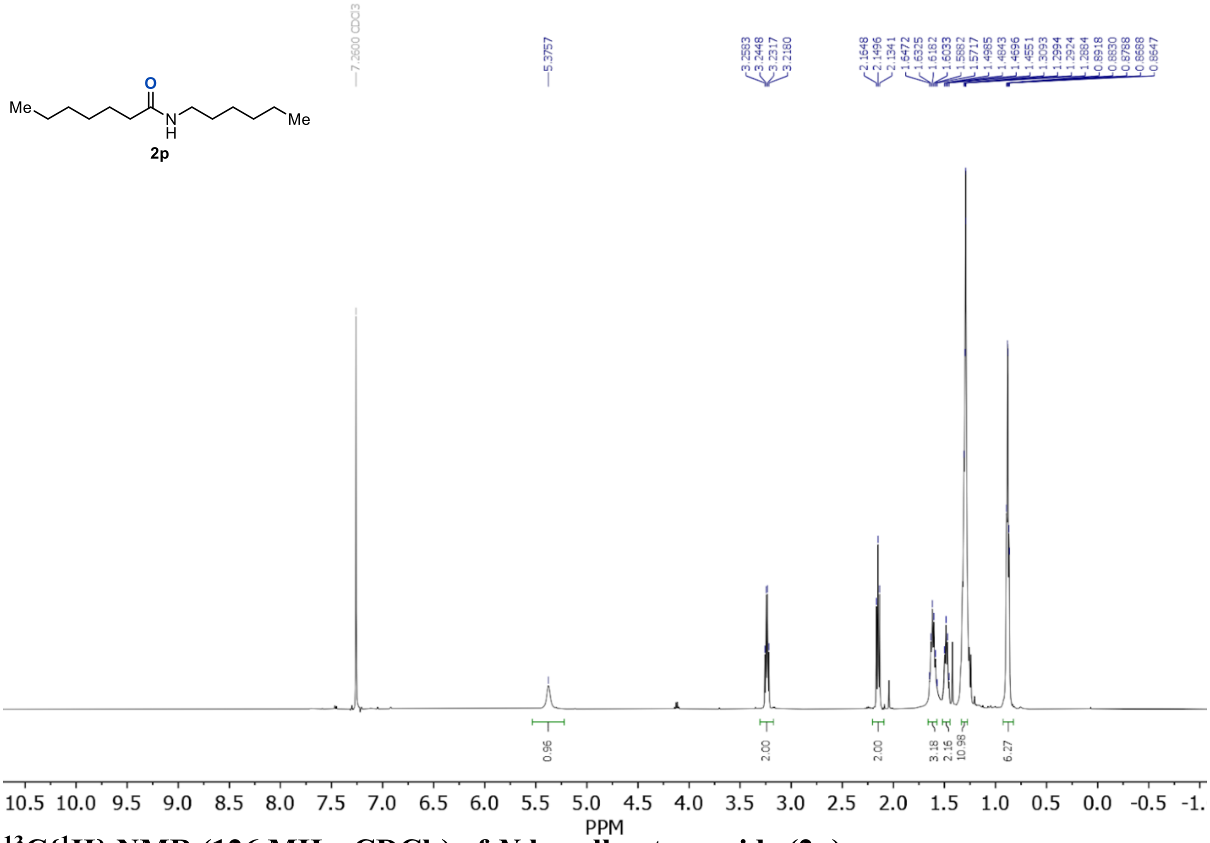

^{13}C{^{1}H} NMR (126 MHz, $CDCl_3$) of *N*-hexylheptanamide (2p)

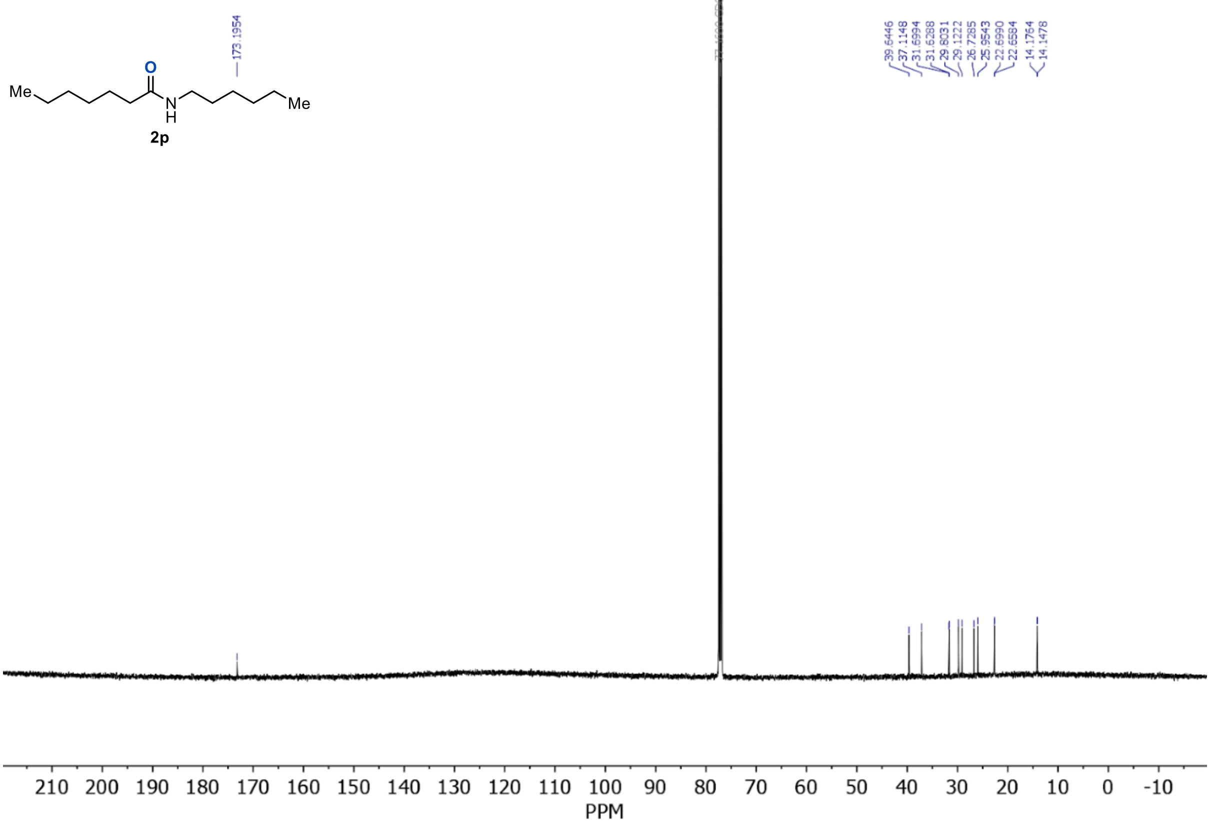

^{1}H NMR (500 MHz, CDCl$_3$) of 7-(1,3-dioxoisoindolin-2-yl)-*N*-hexylheptanamide (2q)

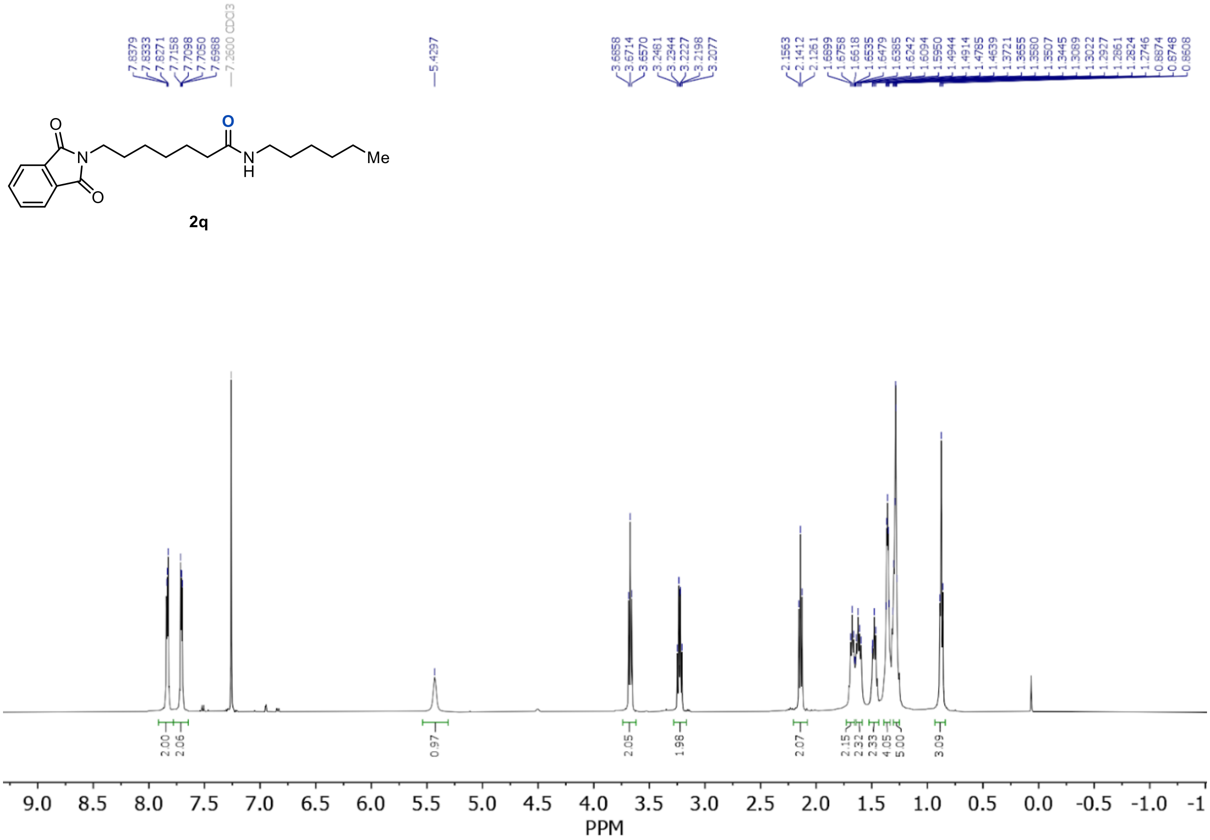

^{13}C{^{1}H} NMR (126 MHz, CDCl$_3$) of 7-(1,3-dioxoisoindolin-2-yl)-*N*-hexylheptanamide (2q)

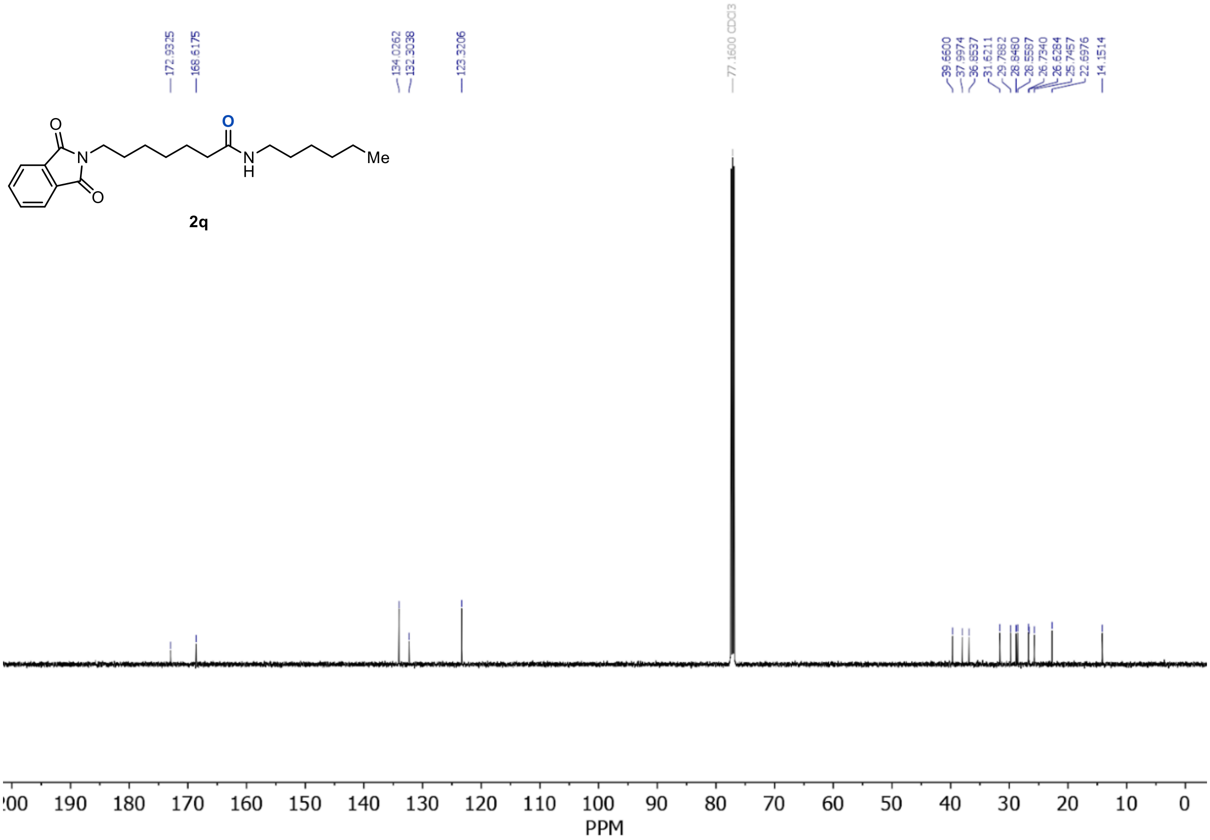

^{1}H NMR (500 MHz, CDCl$_3$) of 2-benzoyl-*N*-hexylpentanamide (2r)

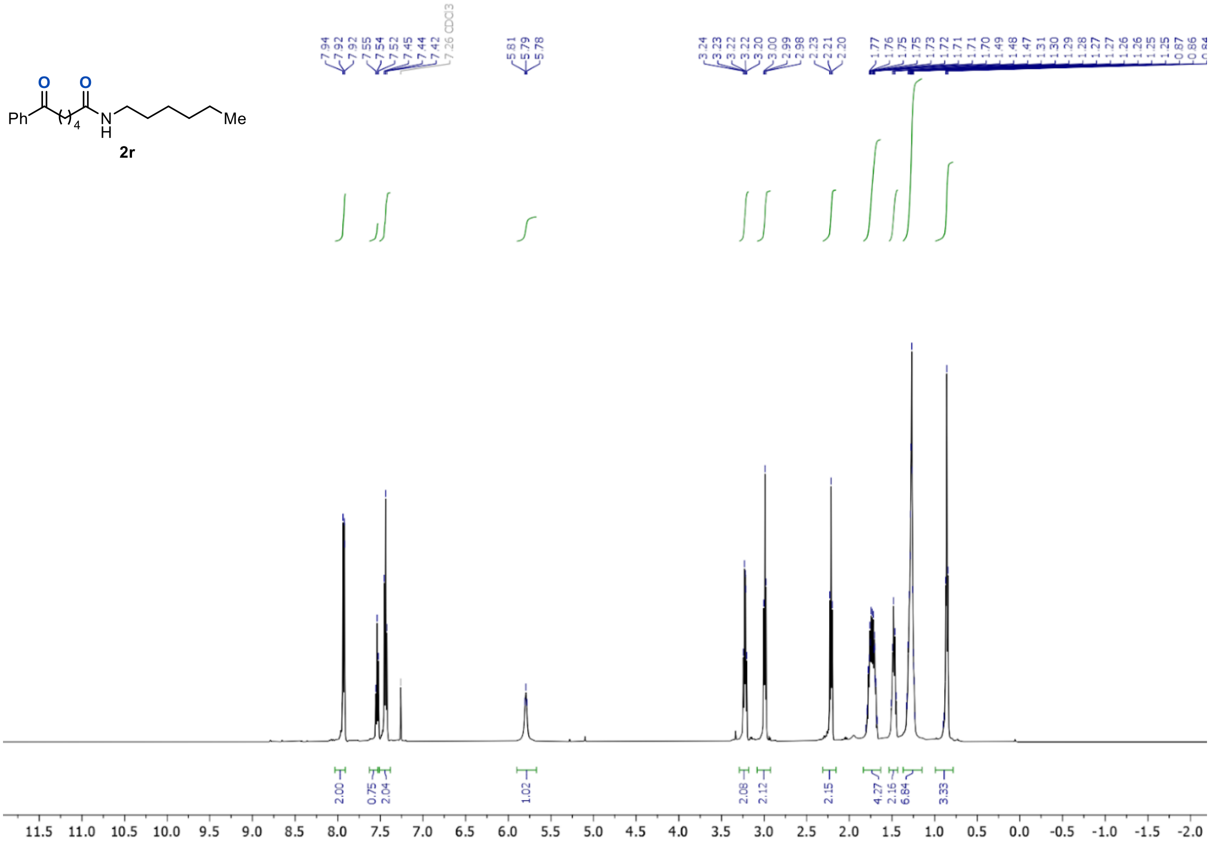

^{13}C{^{1}H} NMR (126 MHz, CDCl$_3$) of 2-benzoyl-*N*-hexylpentanamide (2r)

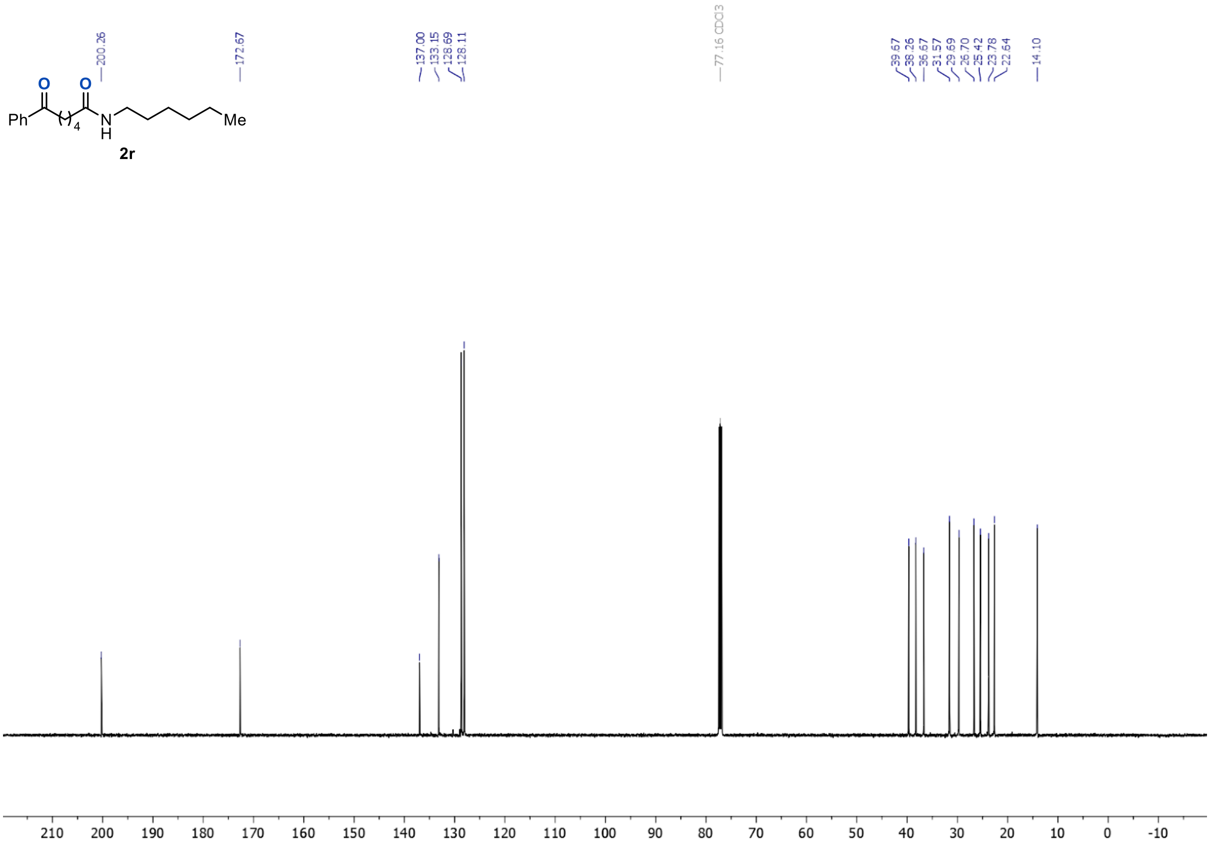

^{1}H NMR (500 MHz, CDCl$_3$) of N-hexyl-2,2-dimethyl-3-phenylpropanamide (2s)

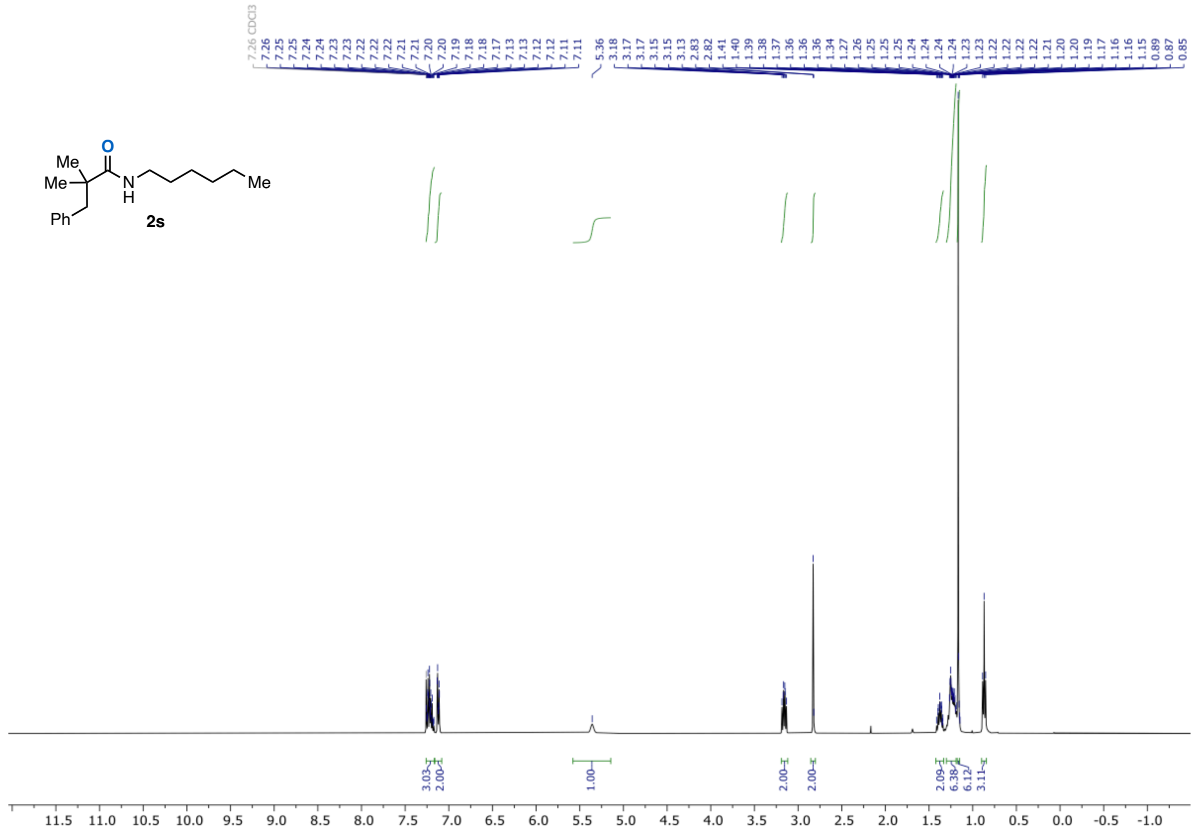

^{13}C{^{1}H} NMR (126 MHz, CDCl$_3$) of N-hexyl-2,2-dimethyl-3-phenylpropanamide (2s)

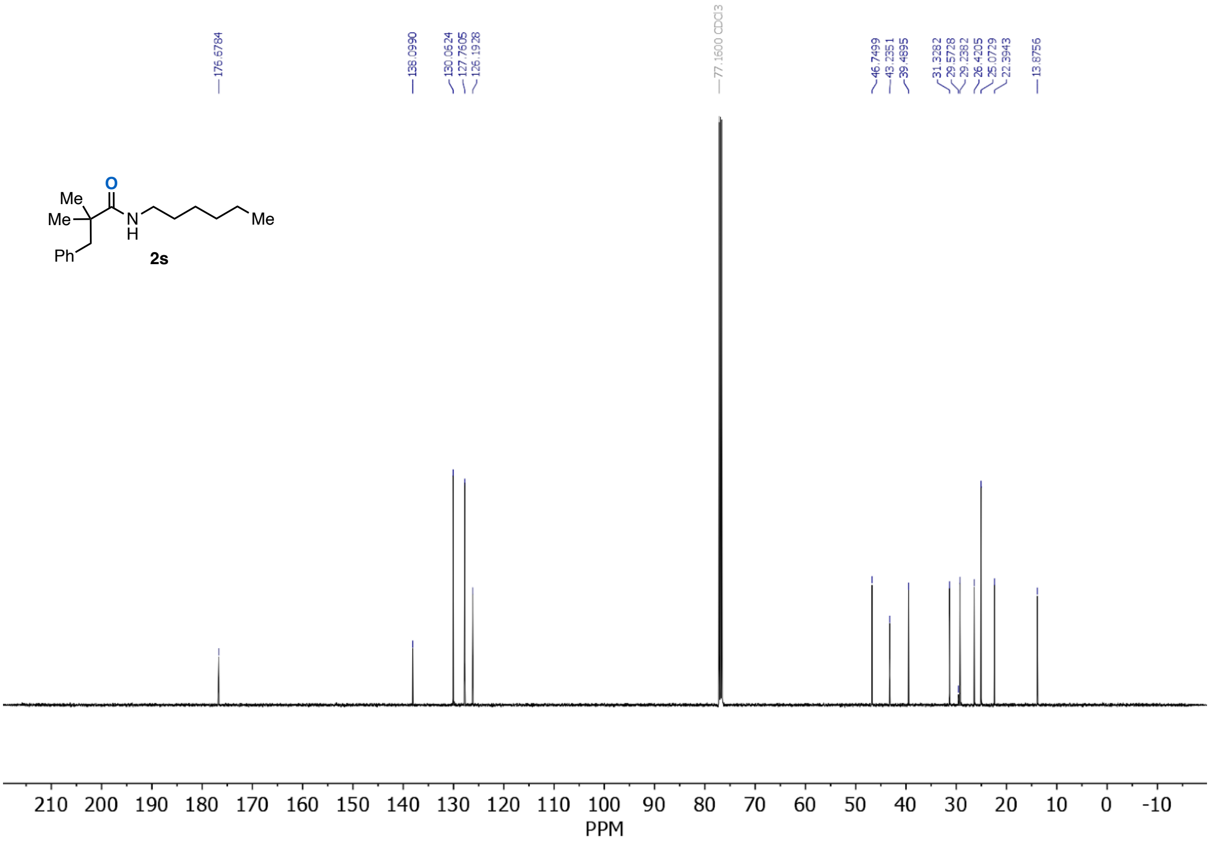

^{1}H NMR (500 MHz, CDCl$_3$) of *N*-hexylcyclohexanecarboxamide (2t)

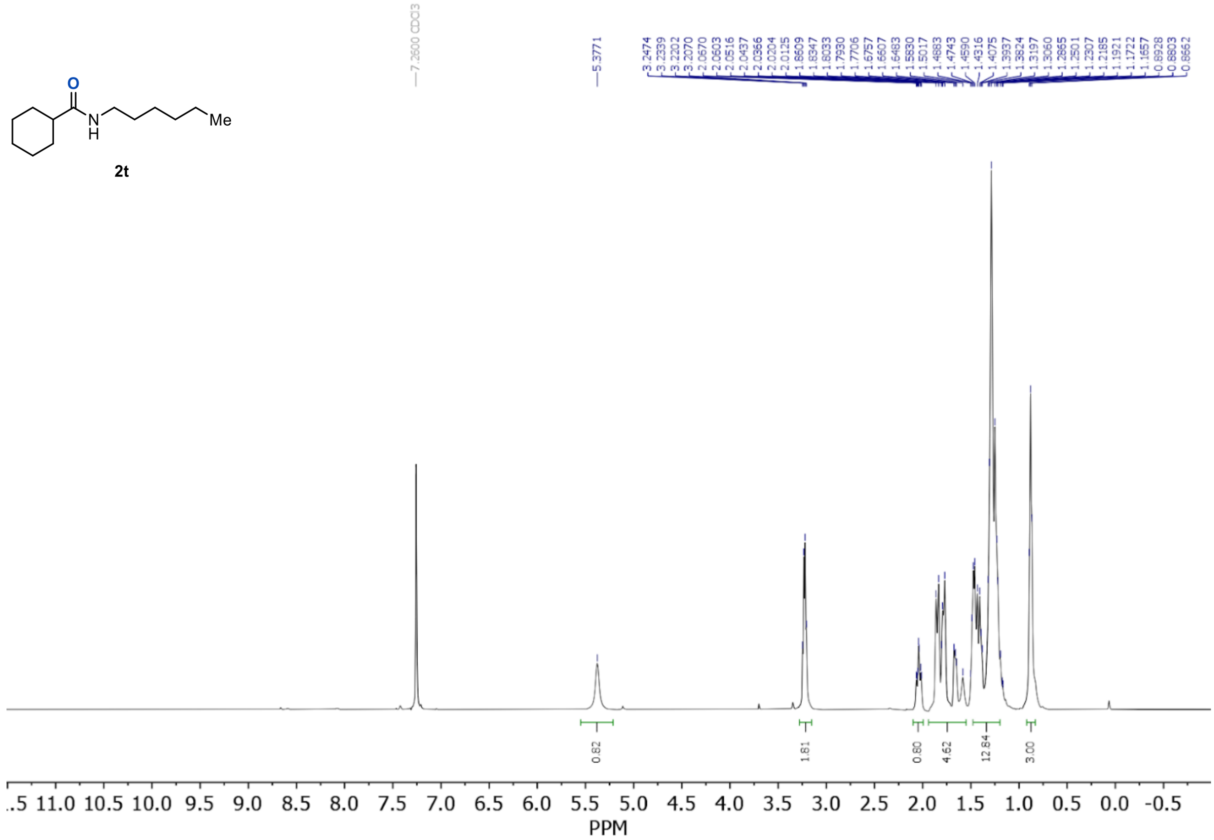

^{13}C{^{1}H} NMR (126 MHz, CDCl$_3$) of *N*-hexylcyclohexanecarboxamide (2t)

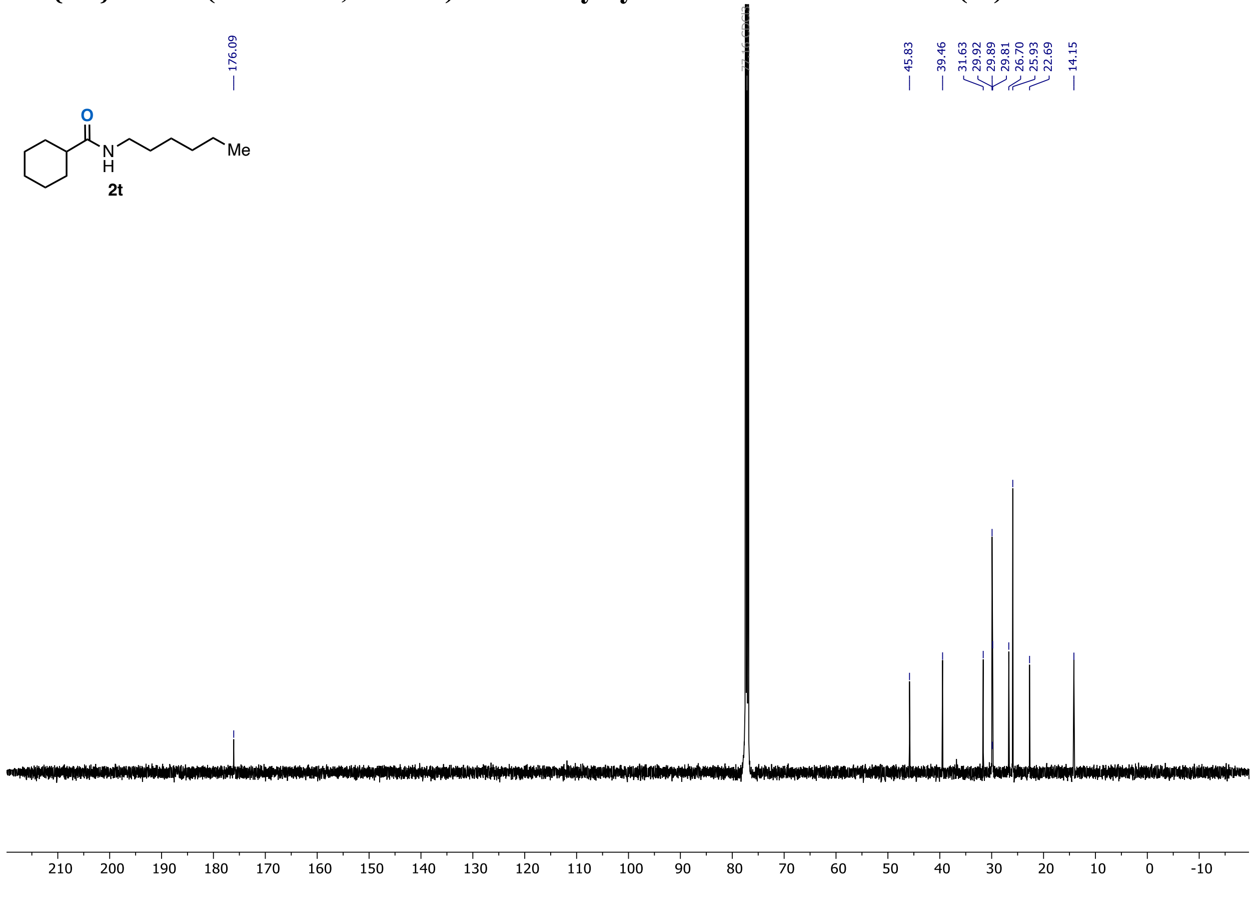

^{1}H NMR (500 MHz, $CDCl_3$) of 5-cyclopentyl-2-((ethylperoxy)-λ^2-methyl)-N-hexyl-2-methylpent-3-ynamide (2u)

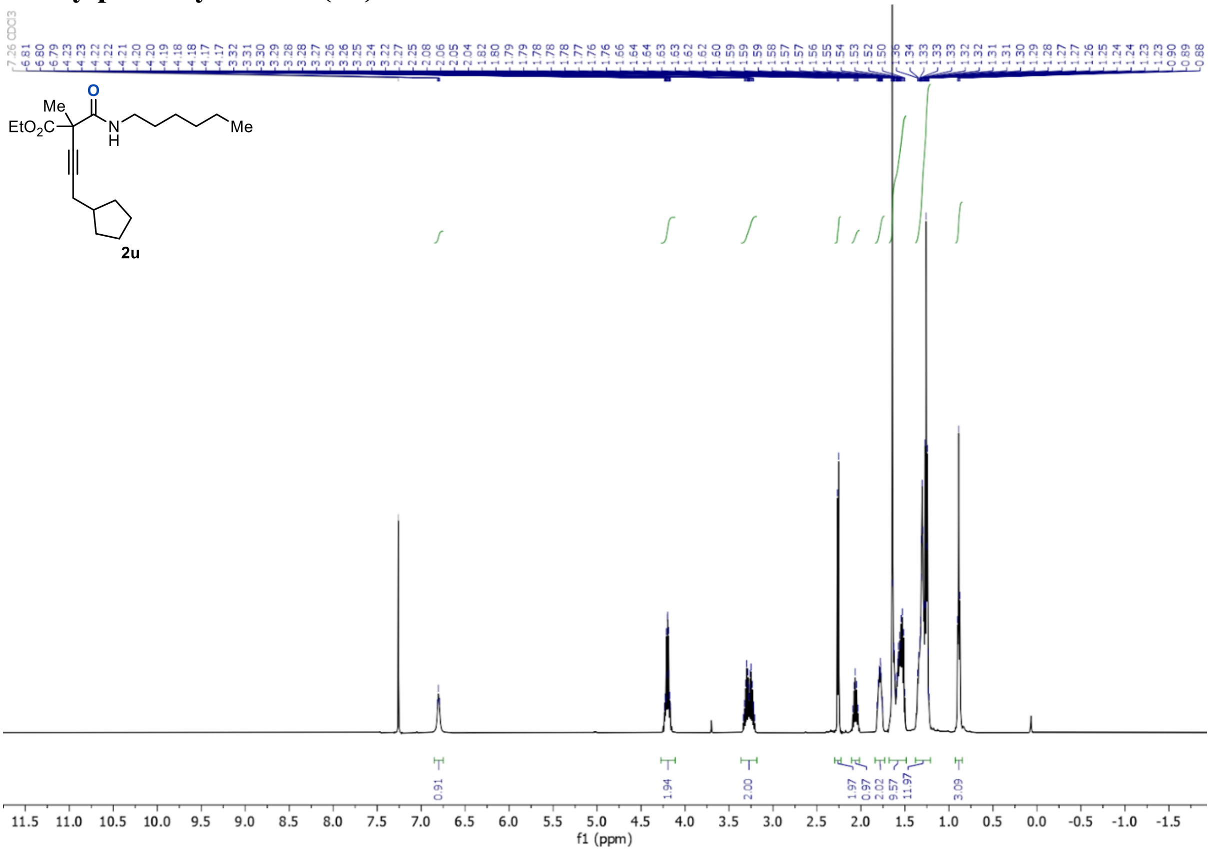

^{13}C{^{1}H} NMR (126 MHz, $CDCl_3$) of 5-cyclopentyl-2-((ethylperoxy)-λ^2-methyl)-N-hexyl-2-methylpent-3-ynamide (2u)

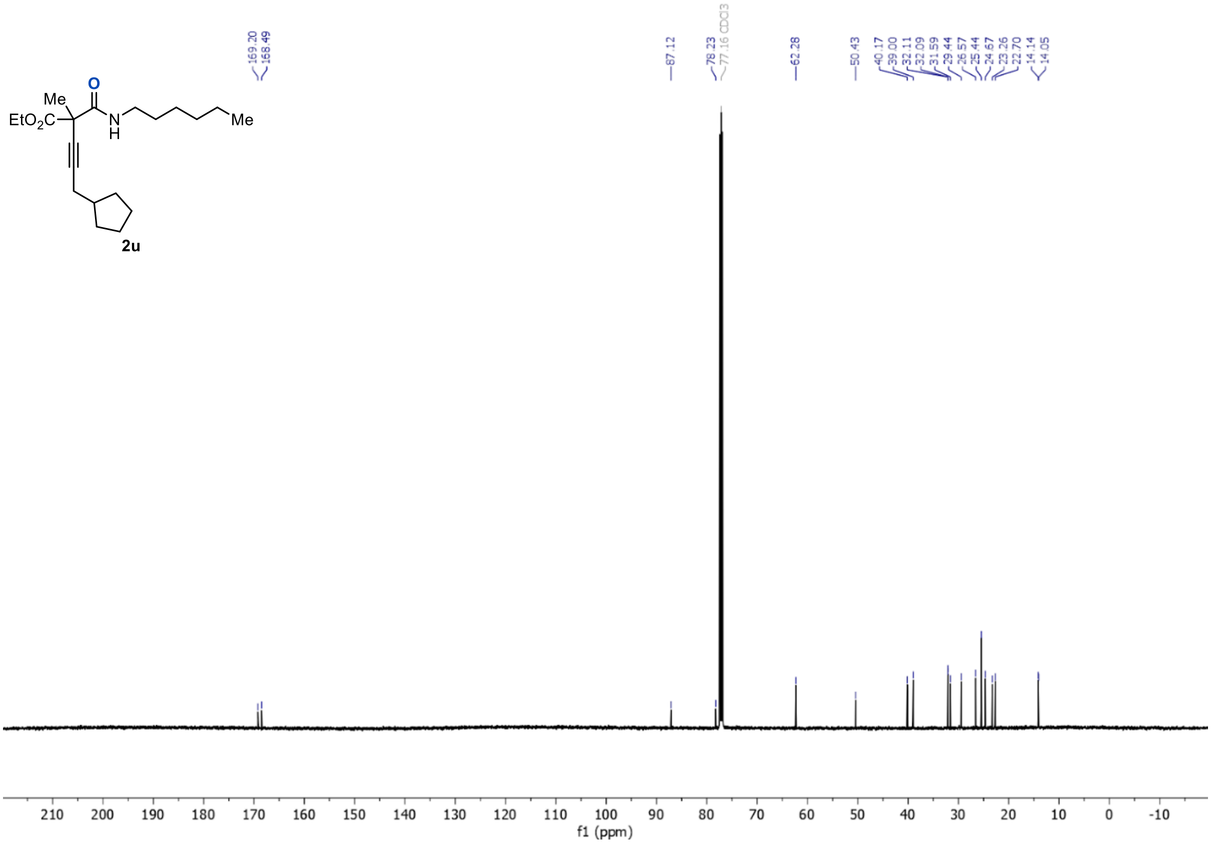

^{1}H NMR (500 MHz, $CDCl_3$) of 2-(1,3-dioxoisoindolin-2-yl)-*N*-hexylacetamide (2v)

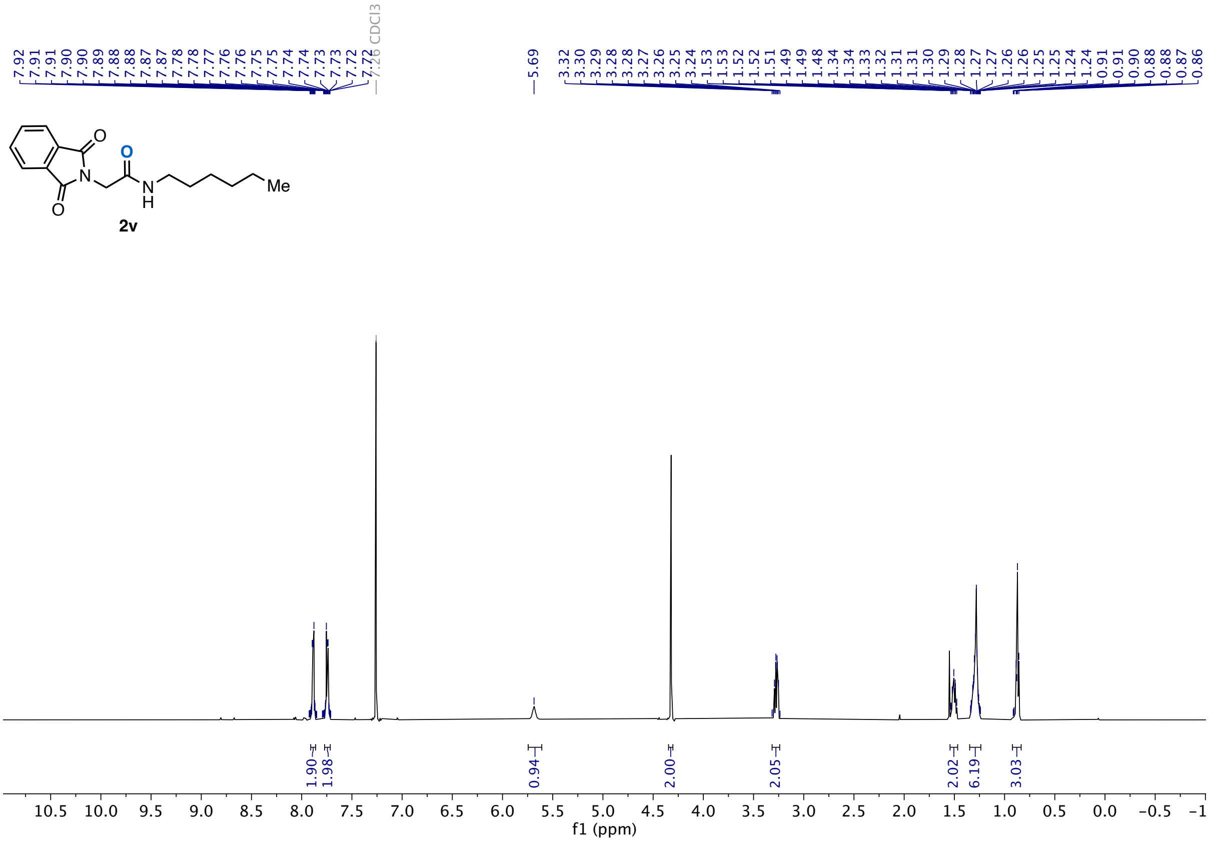

$^{13}C\{^1H\}$ NMR (126 MHz, $CDCl_3$) of 2-(1,3-dioxoisoindolin-2-yl)-*N*-hexylacetamide (2v)

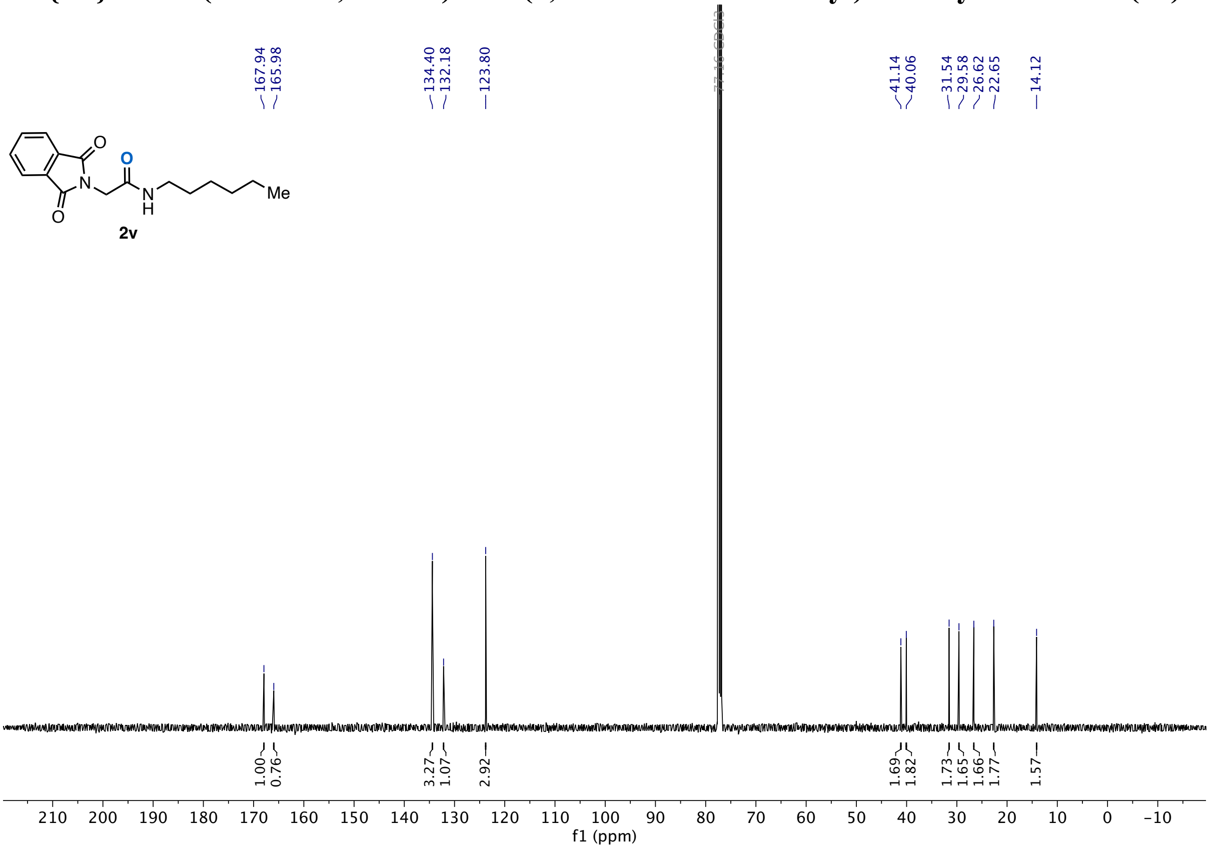

^{1}H NMR (500 MHz, CDCl$_3$) of 4-methyl-*N*-(1,2,3,4-tetrahydronaphthalen-1-yl)benzamide (3a)

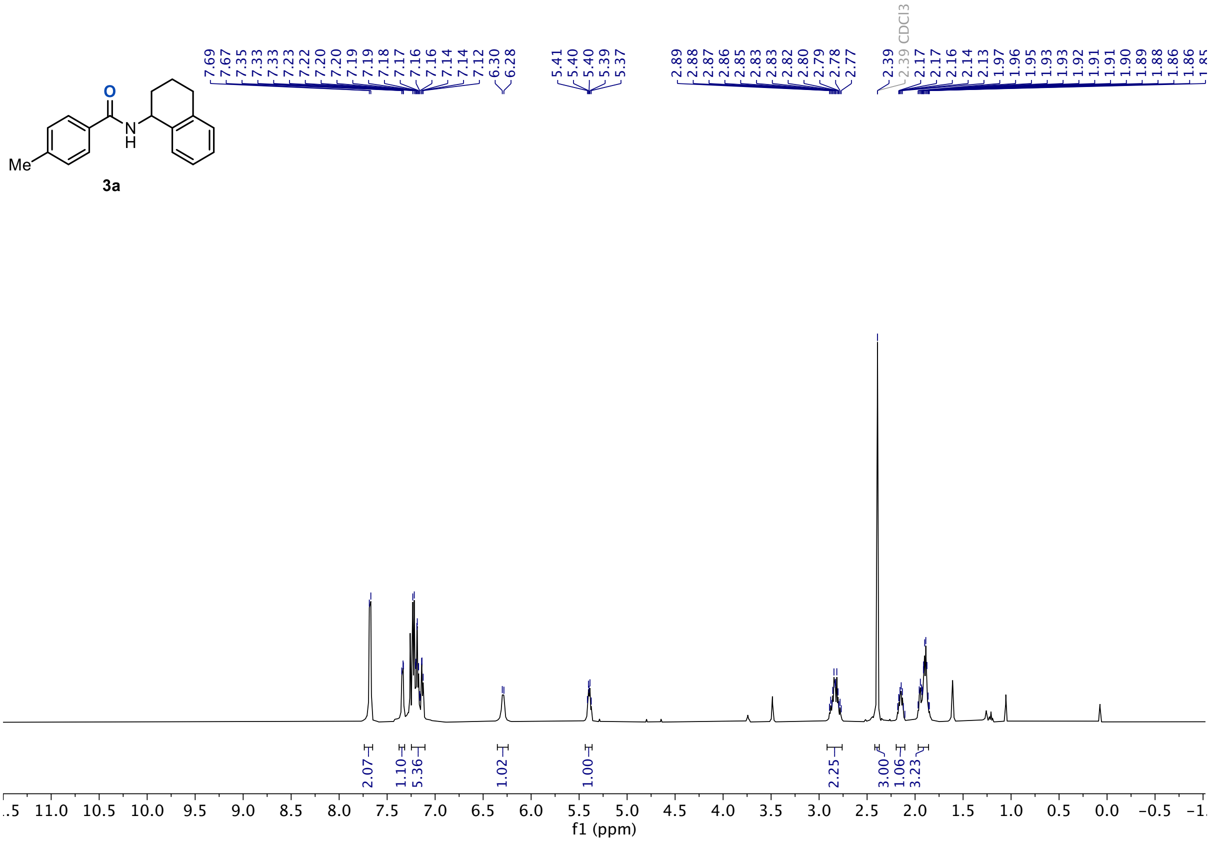

^{13}C{^{1}H} NMR (126 MHz, CDCl$_3$) of 4-methyl-*N*-(1,2,3,4-tetrahydronaphthalen-1-yl)benzamide (3a)

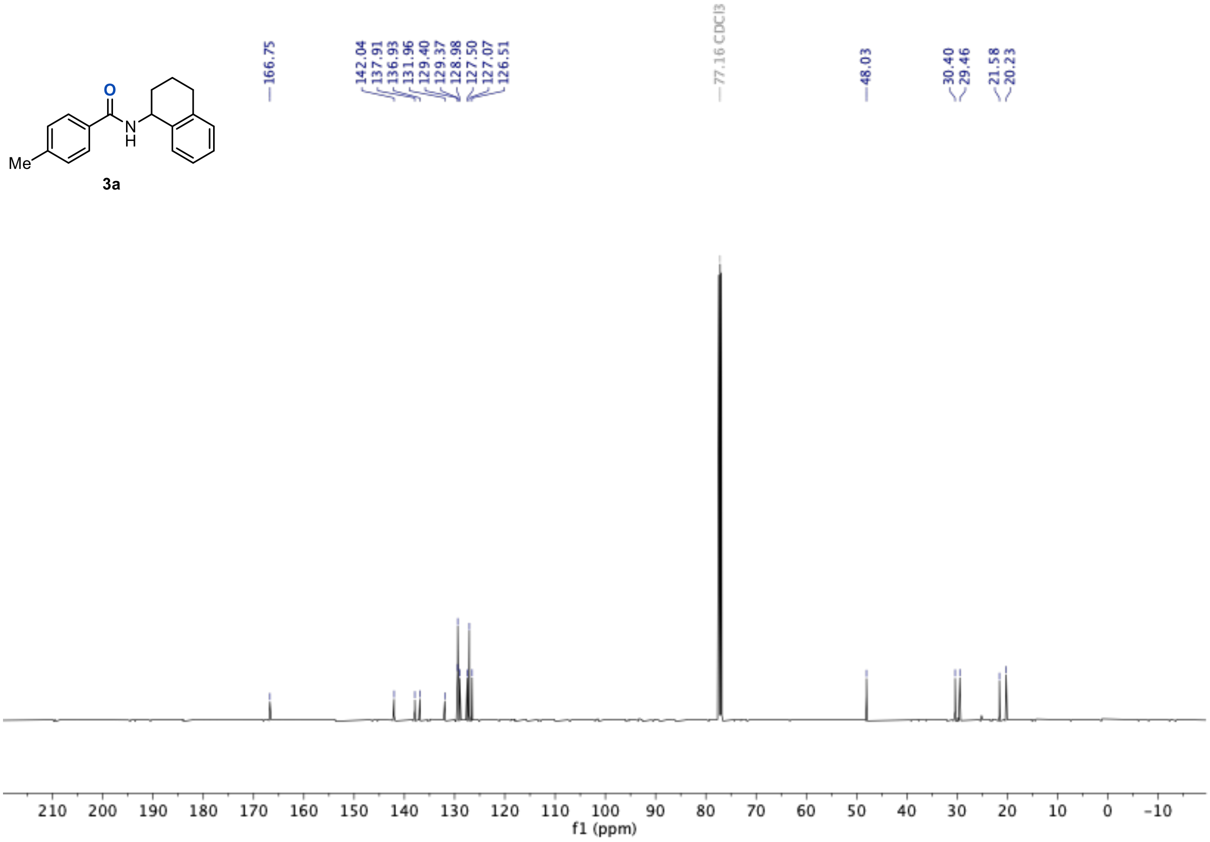

^{1}H NMR (500 MHz, $CDCl_3$) of *N*-(4-(dimethylamino)phenyl)-4-methylbenzamide (3b)

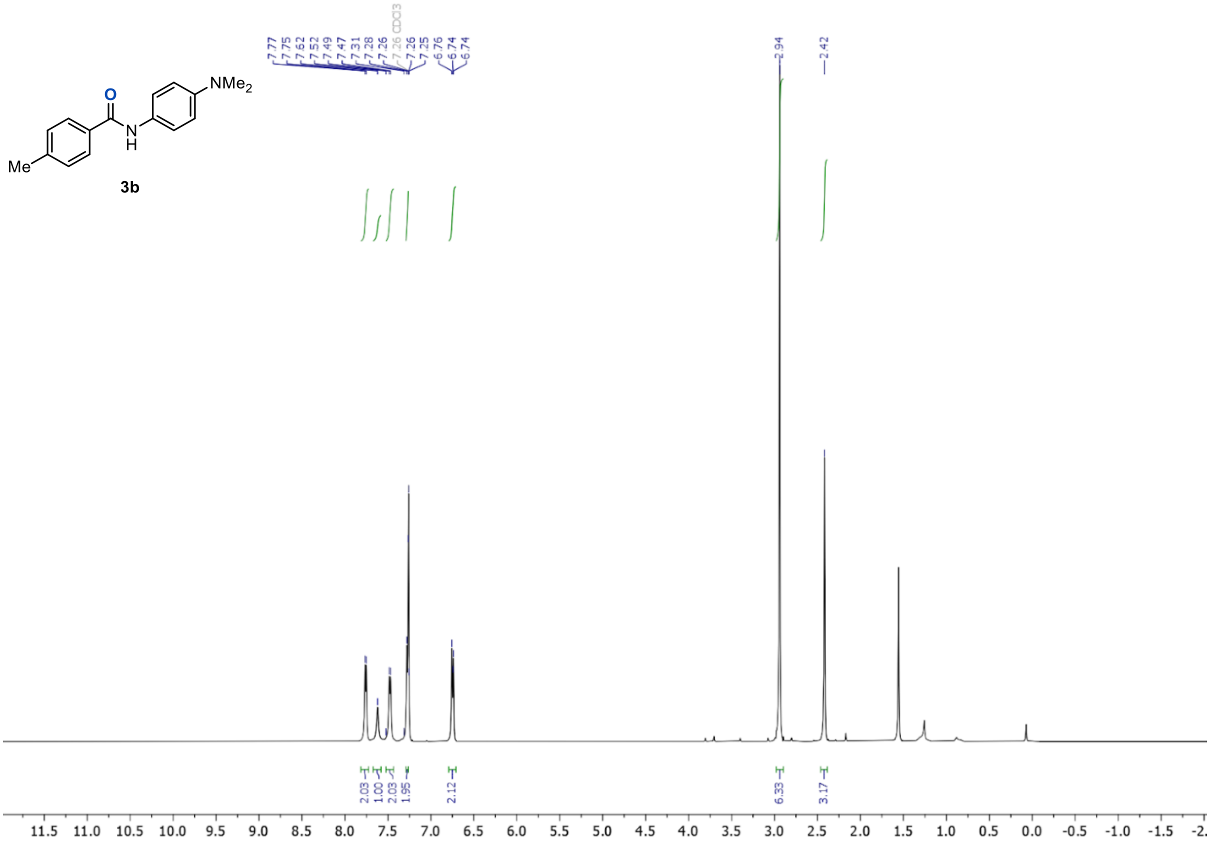

$^{13}C\{^{1}H\}$ NMR (126 MHz, $CDCl_3$) of *N*-(4-(dimethylamino)phenyl)-4-methylbenzamide (3b)

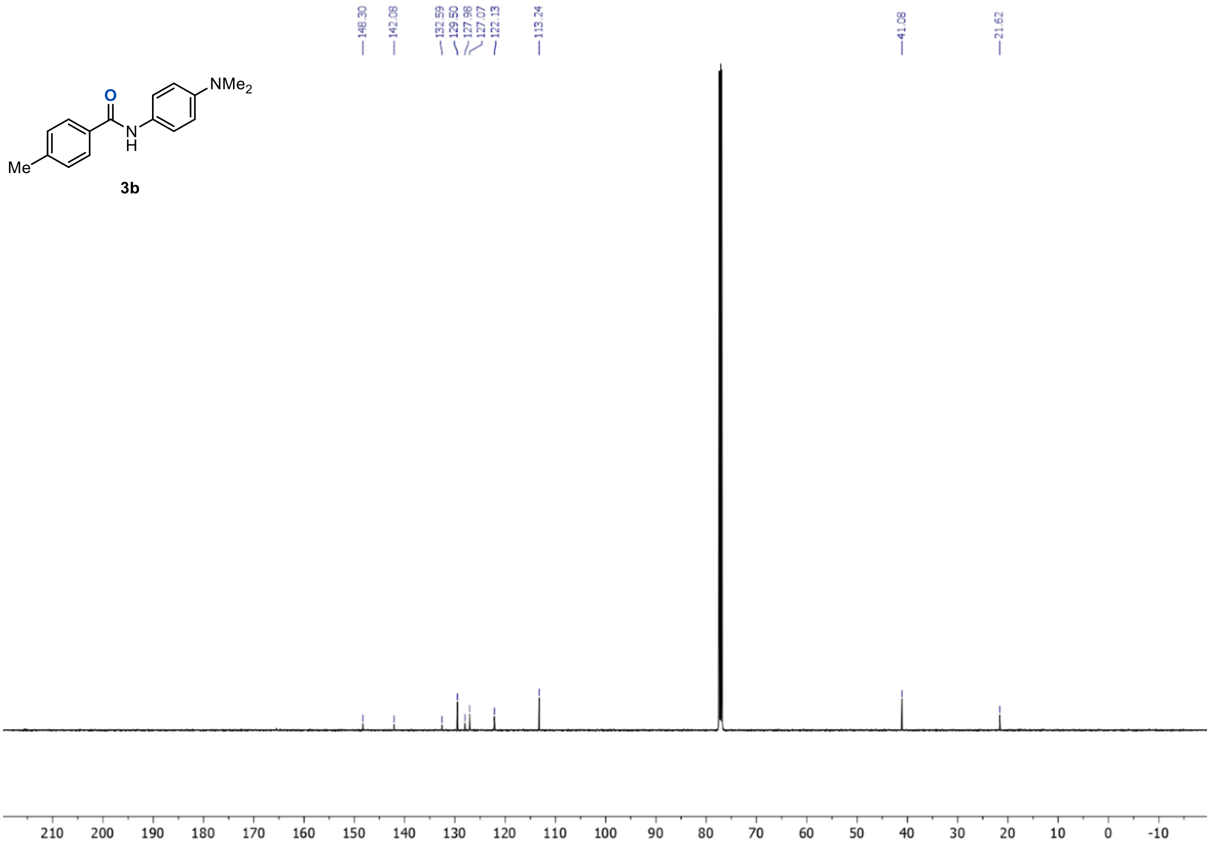

^{1}H NMR (500 MHz, CDCl$_3$) of 4-methyl-*N*-(pyridin-2-yl)benzamide (3c)

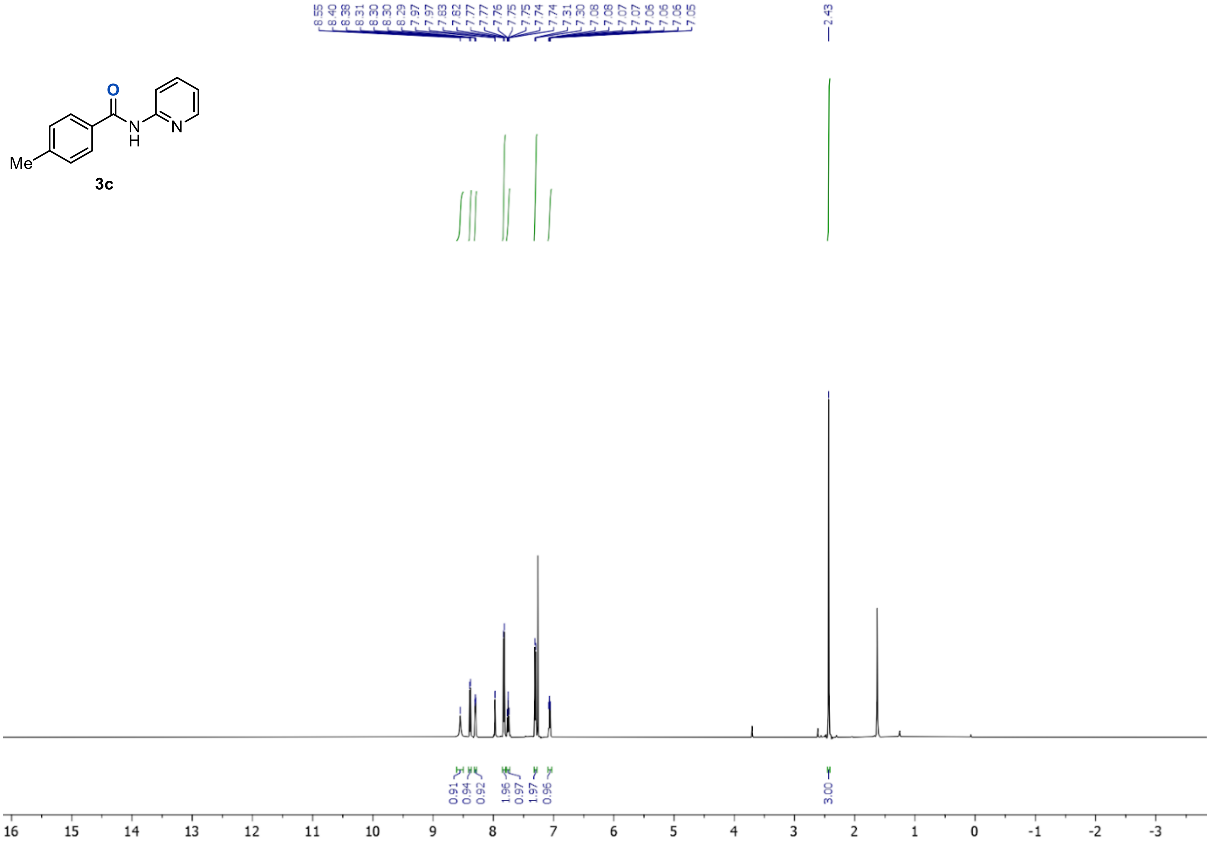

^{13}C{^{1}H} NMR (126 MHz, CDCl$_3$) of 4-methyl-*N*-(pyridin-2-yl)benzamide (3c)

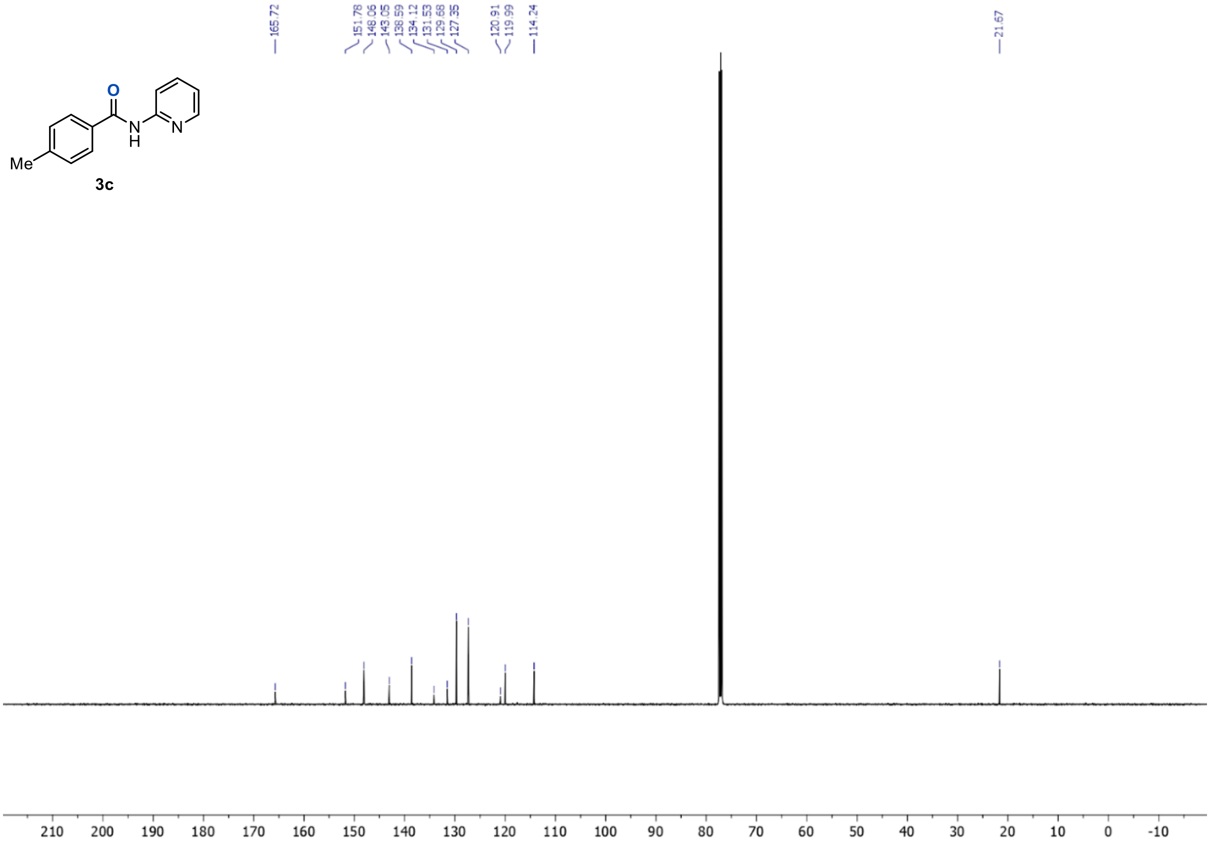

^{1}H NMR (500 MHz, $CDCl_3$) of *N*-methoxy-*N*,4-dimethylbenzamide (3d)

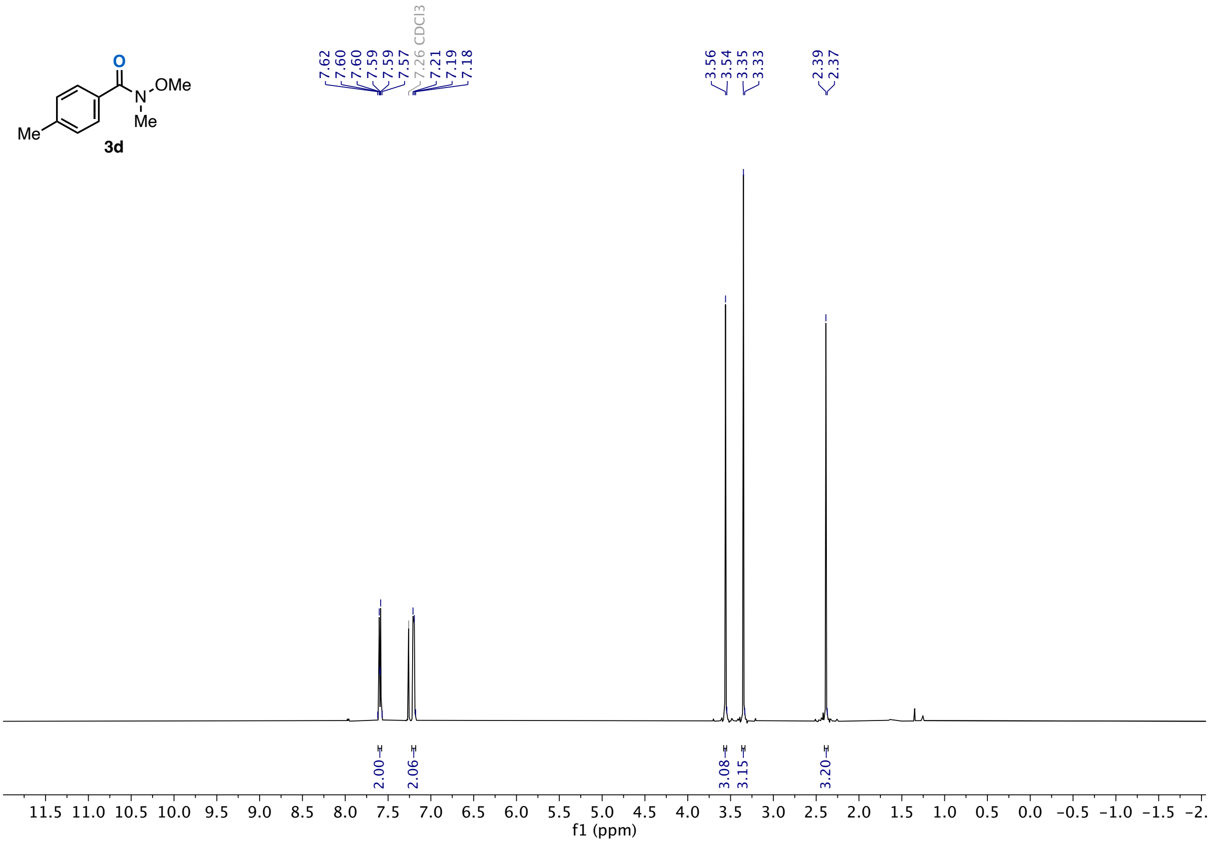

$^{13}C\{^{1}H\}$ NMR (126 MHz, $CDCl_3$) of *N*-methoxy-4-methylbenzamide (3d)

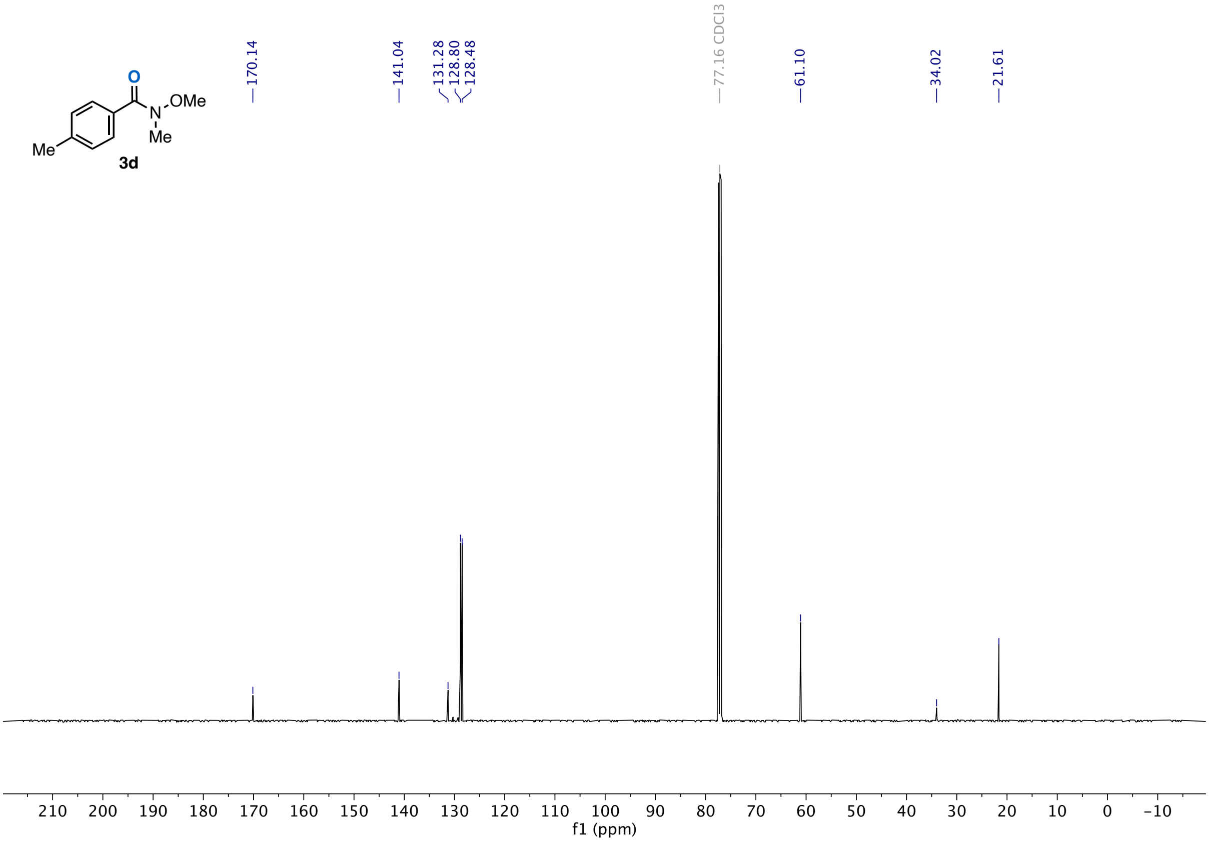

^{1}H NMR (500 MHz, $CDCl_3$) of *N*,*N*-bis(2-hydroxyethyl)-4-methylbenzamide (3e)

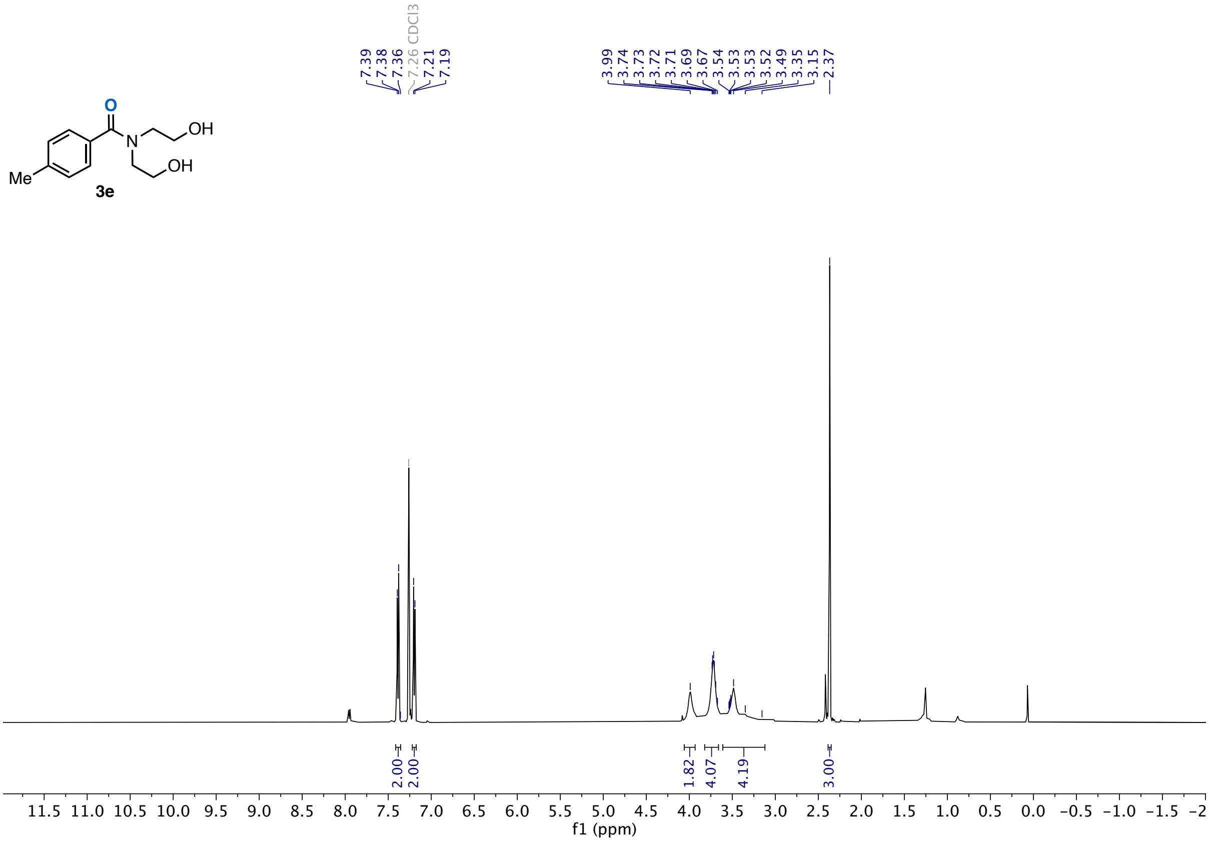

^{13}C{^{1}H} NMR (101 MHz, $CDCl_3$) of *N*,*N*-bis(2-hydroxyethyl)-4-methylbenzamide (3e)

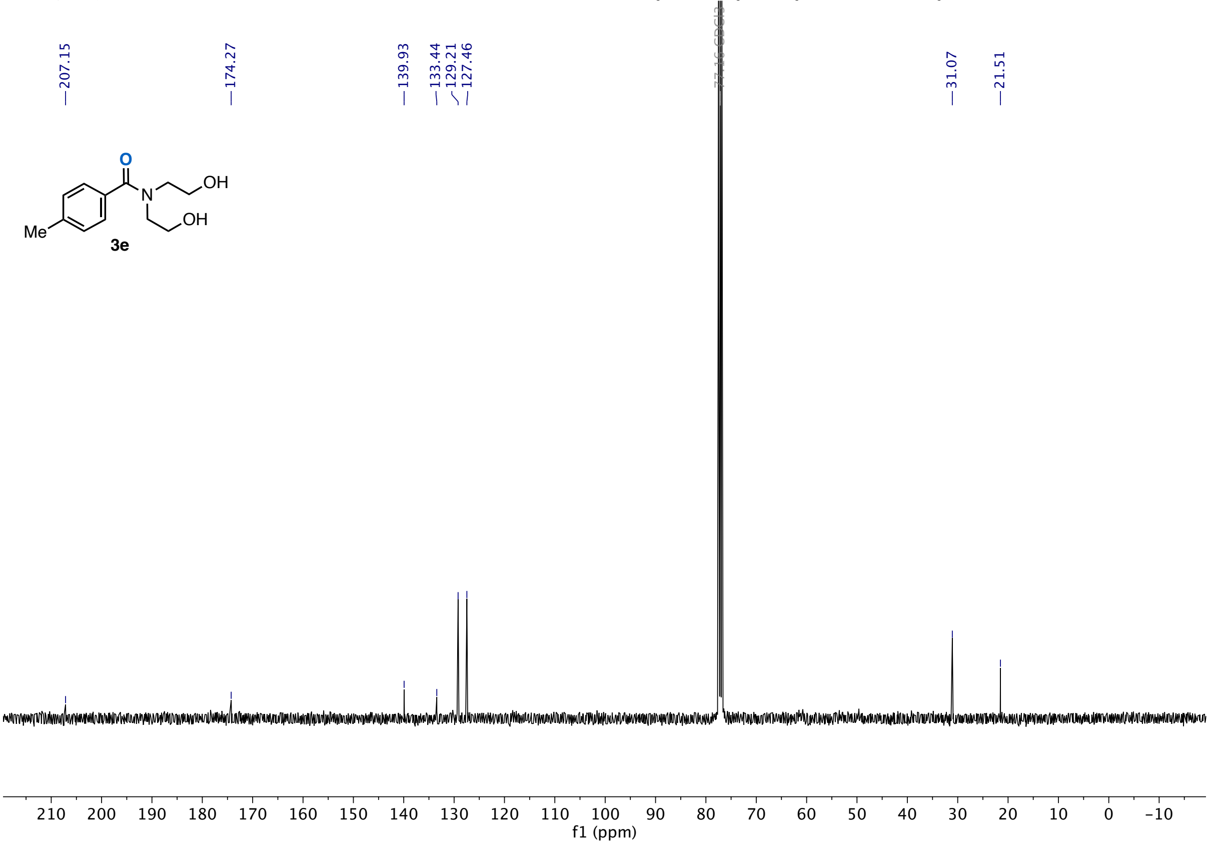

^{1}H NMR (500 MHz, CDCl$_3$) of (4-(8-chloro-5,6-dihydro-11H-benzo[5,6]cyclohepta[1,2-b]pyridin-11-ylidene)piperidin-1-yl)(p-tolyl)methanone (3f)

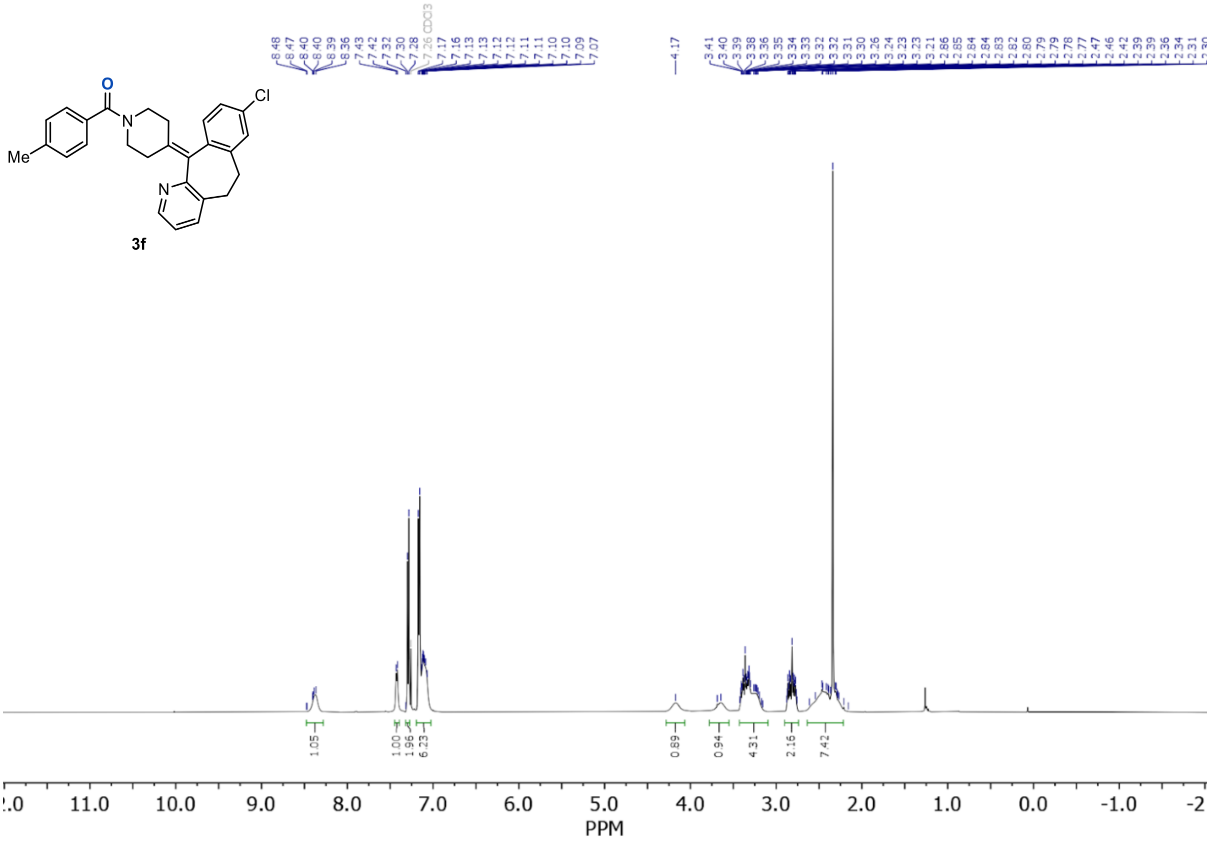

^{13}C{^{1}H} NMR (126 MHz, CDCl$_3$) of (4-(8-chloro-5,6-dihydro-11H-benzo[5,6]cyclohepta[1,2-b]pyridin-11-ylidene)piperidin-1-yl)(p-tolyl)methanone (3f)

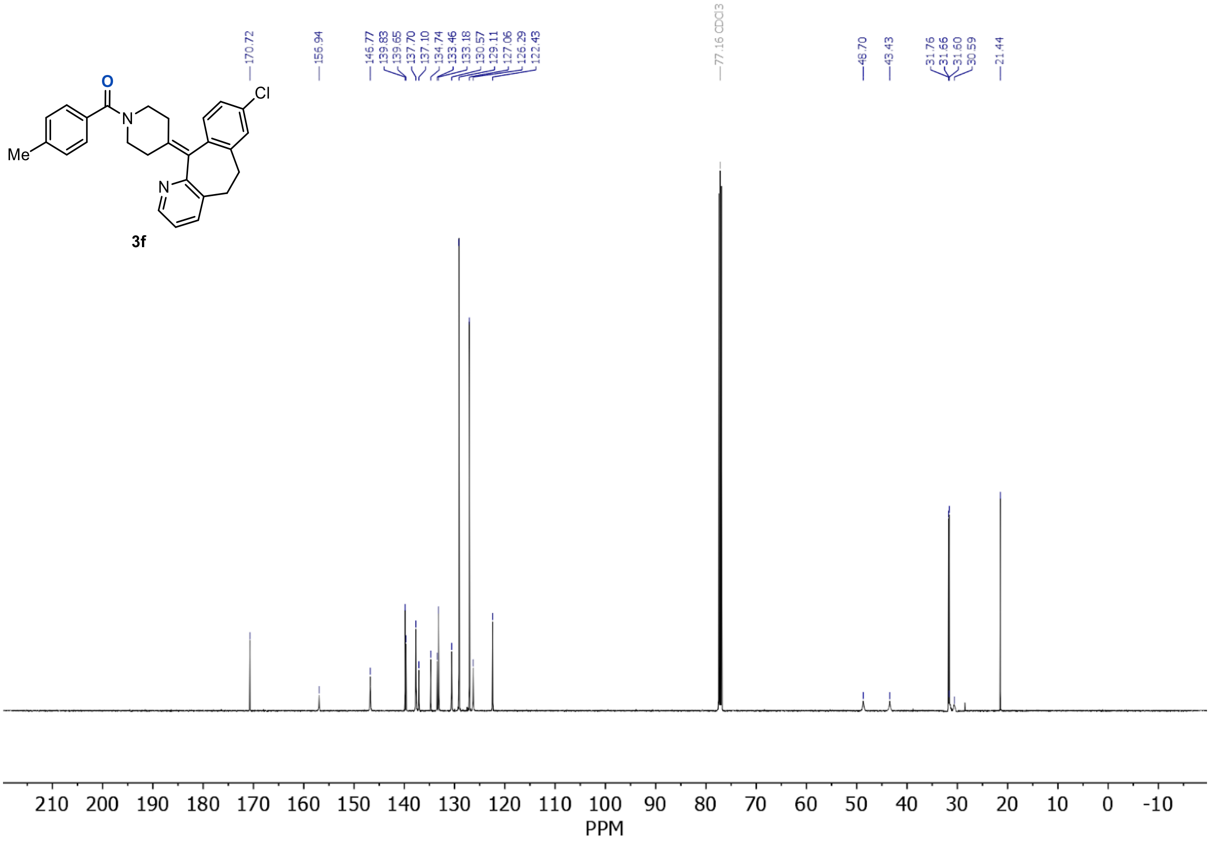

^{1}H NMR (500 MHz, $CDCl_3$) of 4-formyl-2-methoxyphenyl 4-methylbenzoate (3g)

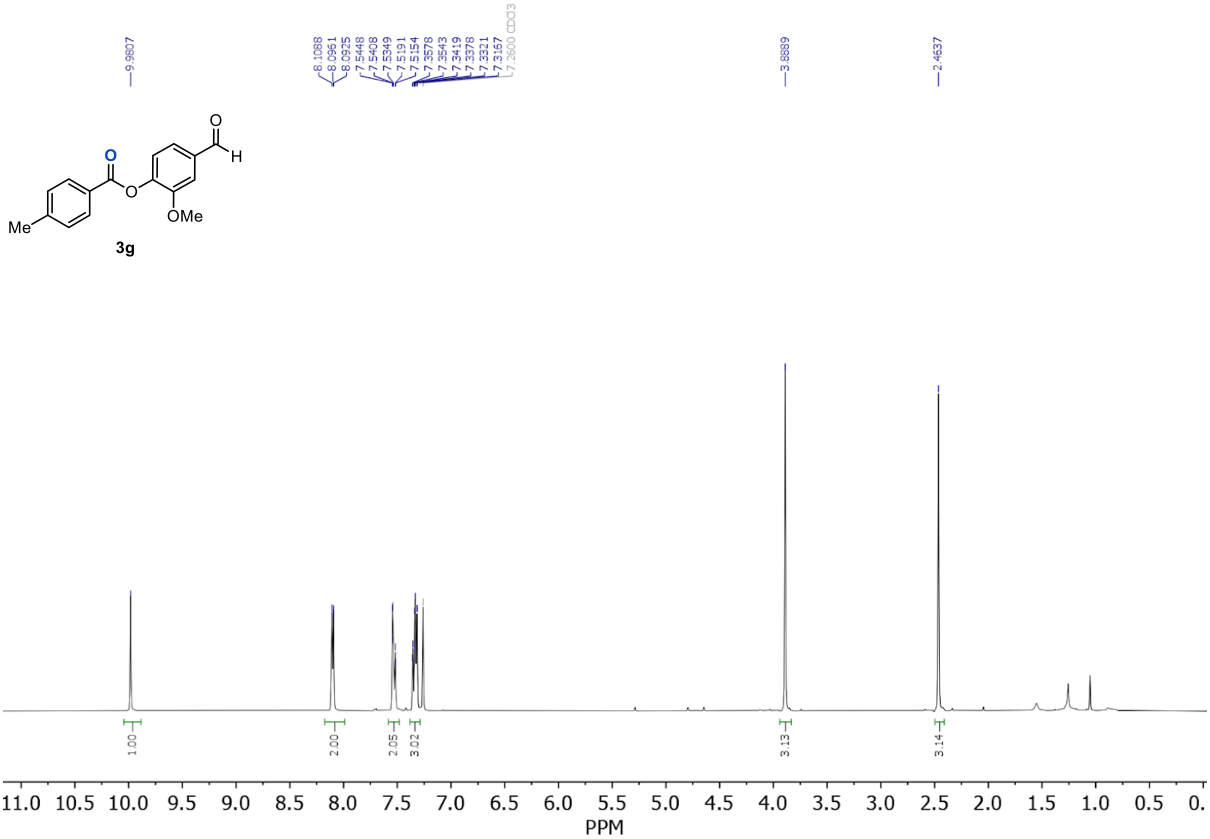

$^{13}C\{^{1}H\}$ NMR (126 MHz, $CDCl_3$) of 4-formyl-2-methoxyphenyl 4-methylbenzoate (3g)

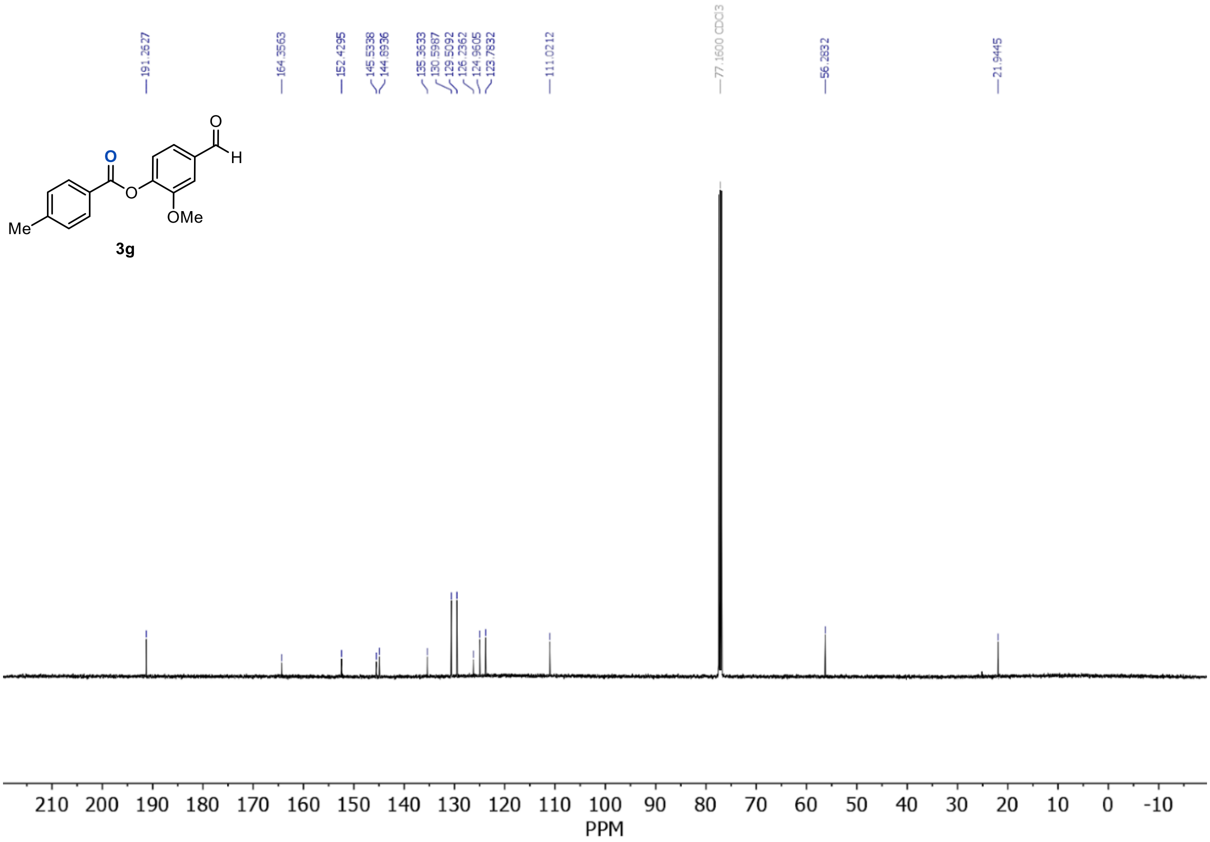

^{1}H NMR (600 MHz, $CDCl_3$) of (*R*)-(6-methoxyquinolin-4-yl)((1*S*,2*S*,4*S*,5*R*)-5-vinylquinuclidin-2-yl)methyl 4-methylbenzoate (3h)

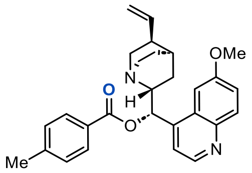

3h

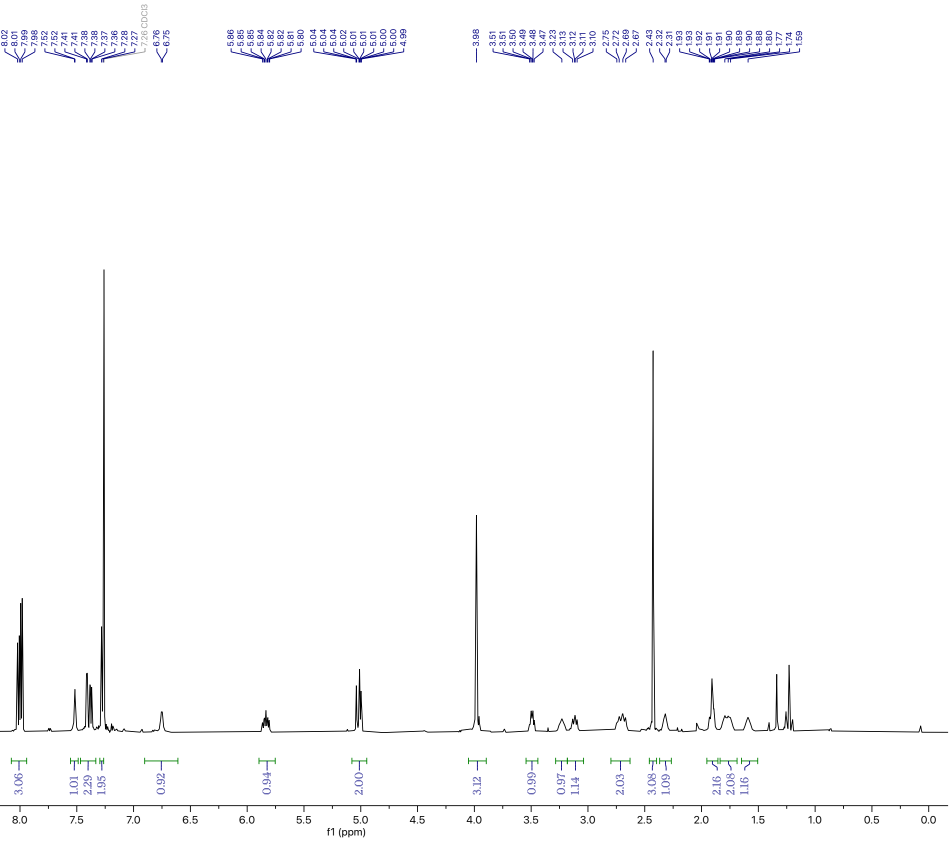

$^{13}C\{^{1}H\}$ NMR (151 MHz, $CDCl_3$) of (*R*)-(6-methoxyquinolin-4-yl)((1*S*,2*S*,4*S*,5*R*)-5-vinylquinuclidin-2-yl)methyl 4-methylbenzoate (3h)

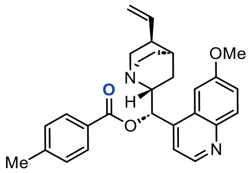

3h

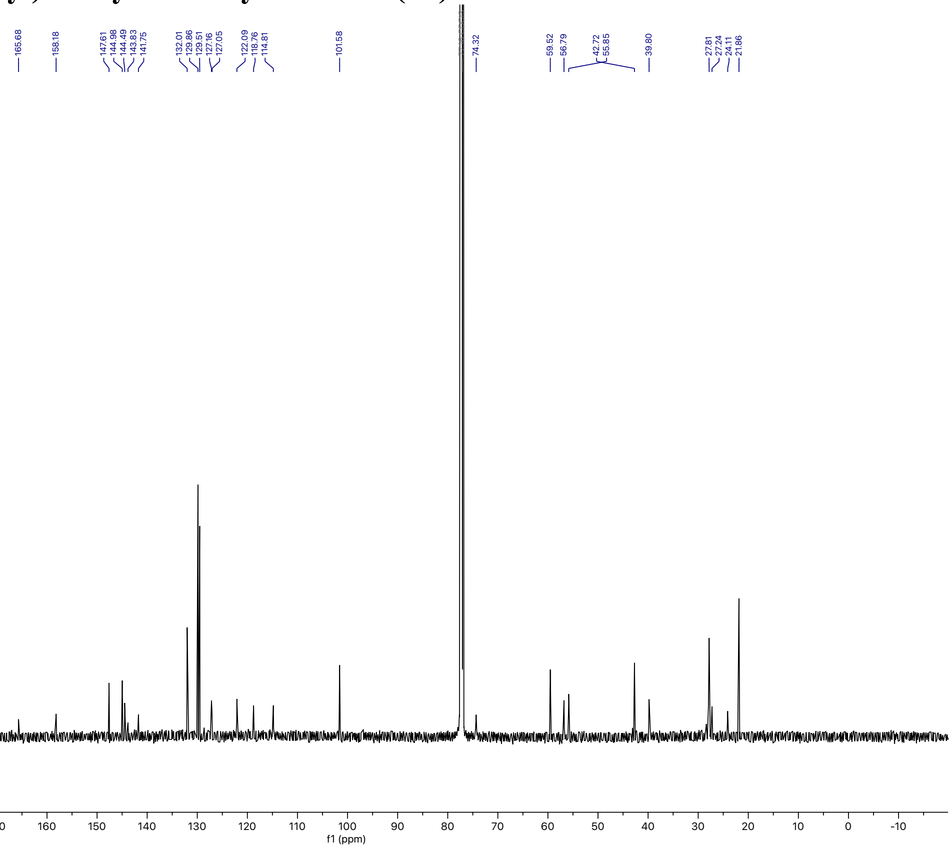

^{1}H NMR (500 MHz, $CDCl_3$) of *S*-hexyl 4-methylbenzothioate (3i)

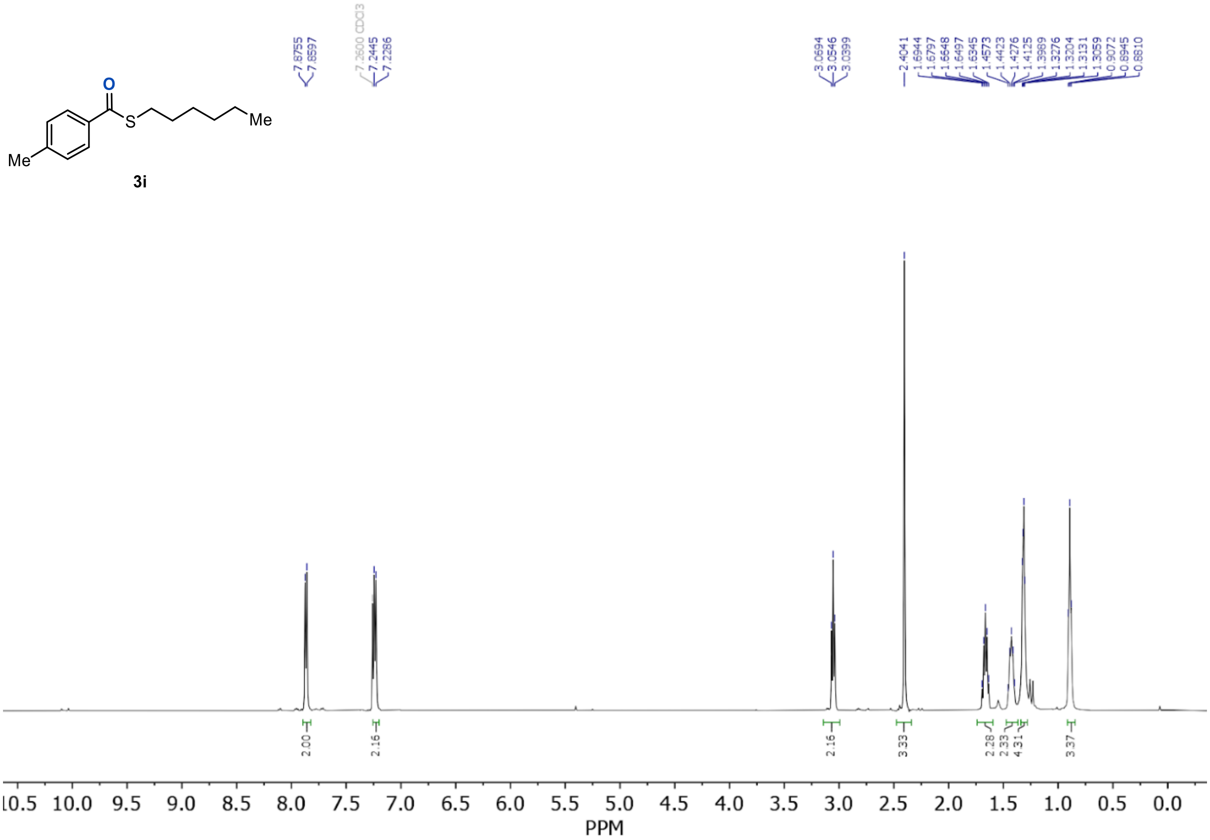

$^{13}C\{^1H\}$ NMR (126 MHz, $CDCl_3$) of *S*-hexyl 4-methylbenzothioate (3i)

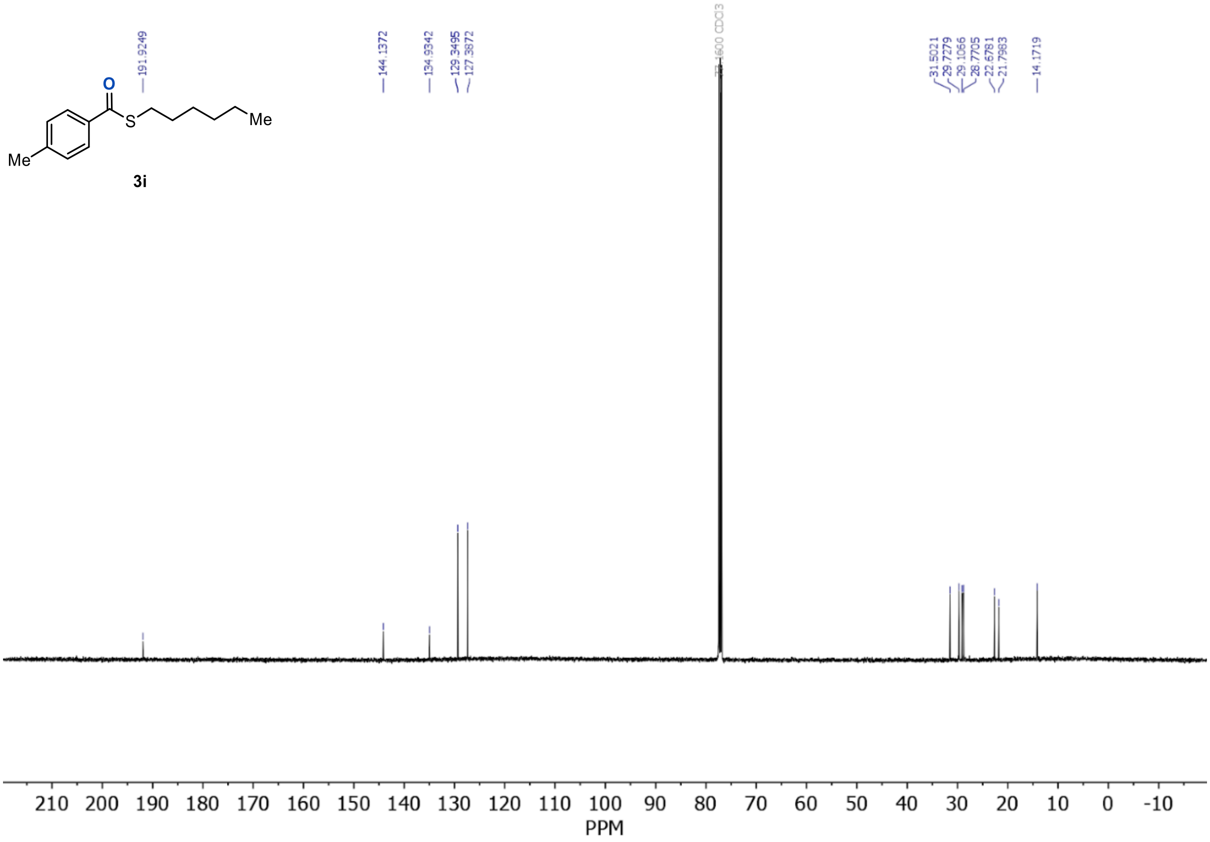

^{1}H NMR (400 MHz, CDCl$_3$) of *S*-((3*R*,5*S*)-adamantan-1-yl) 4-methylbenzothioate (3j)

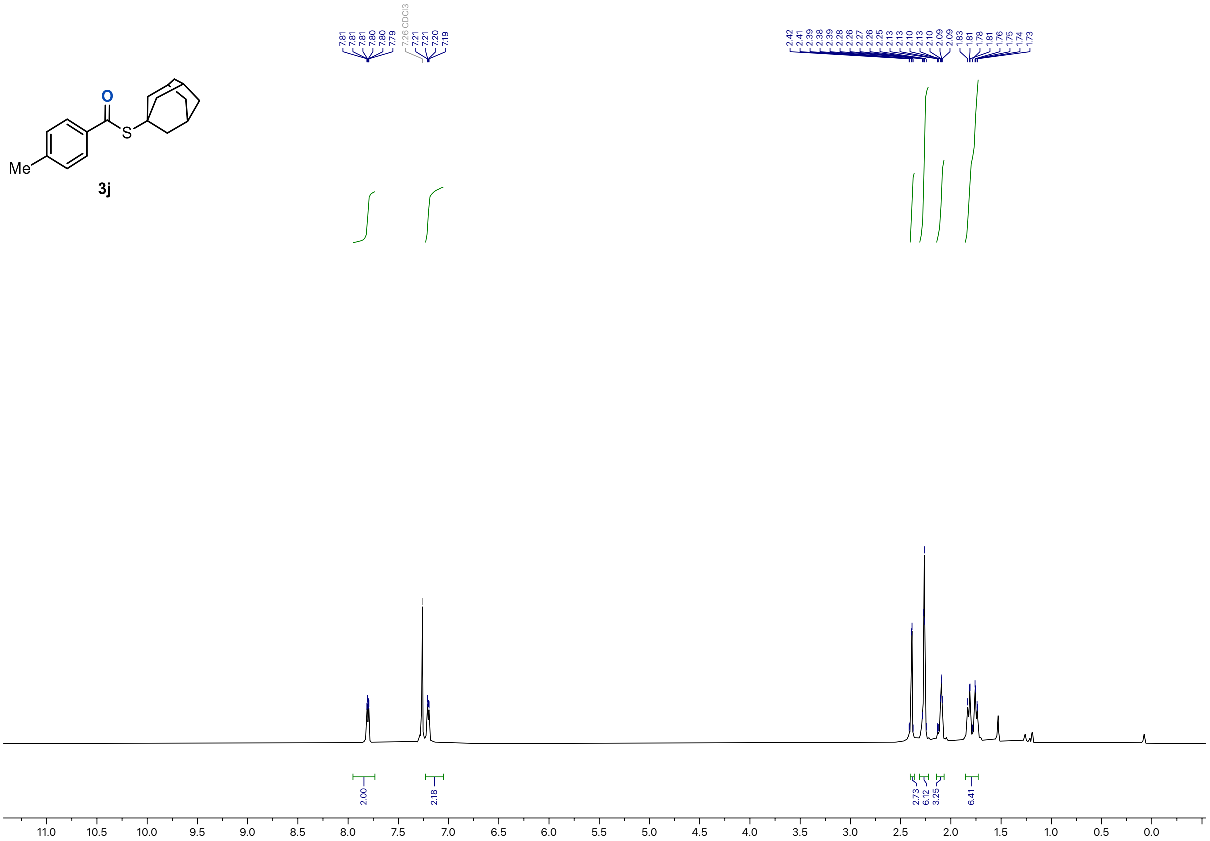

^{13}C{^{1}H} NMR (101 MHz, CDCl$_3$) of *S*-((3*R*,5*S*)-adamantan-1-yl) 4-methylbenzothioate (3j)

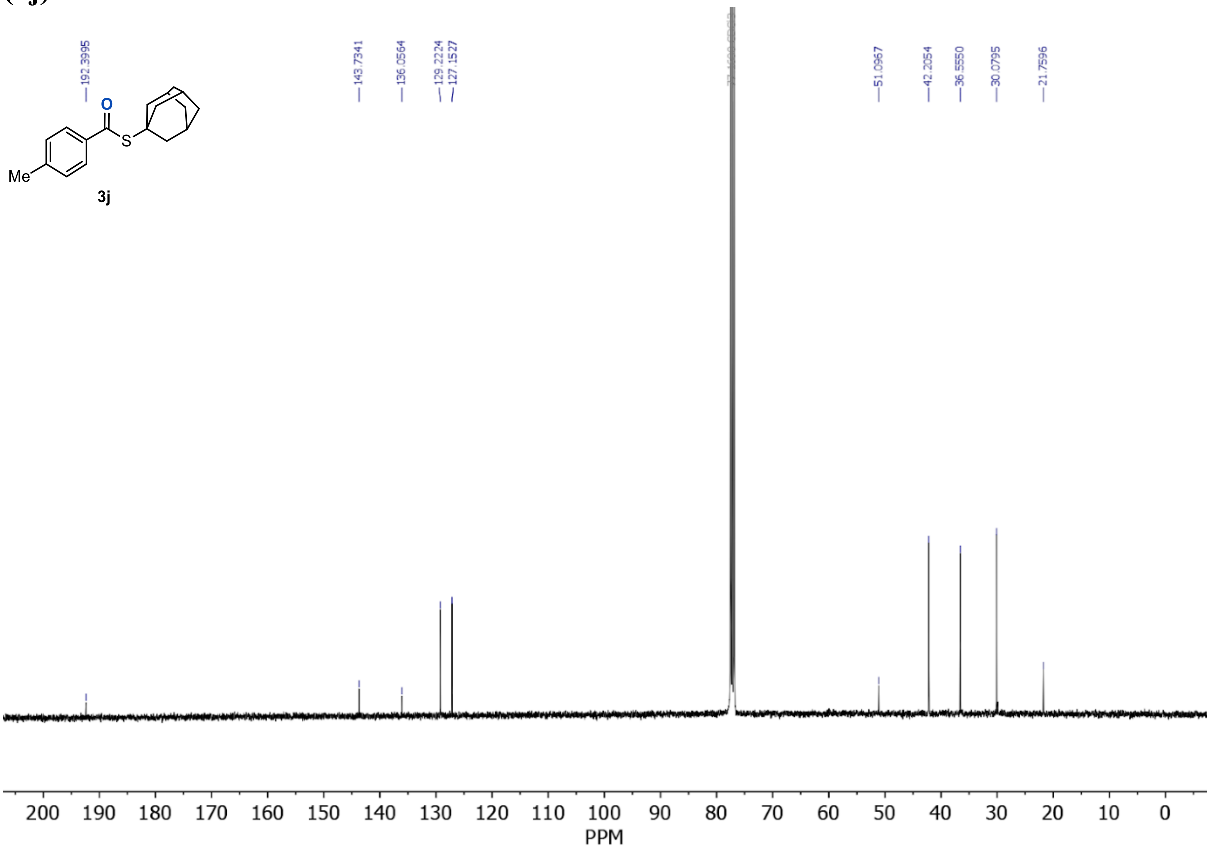

^{1}H NMR (500 MHz, CDCl$_3$) of methyl (*S*)-3,3-dimethyl-2-(4-methylbenzamido)butanoate (3k)

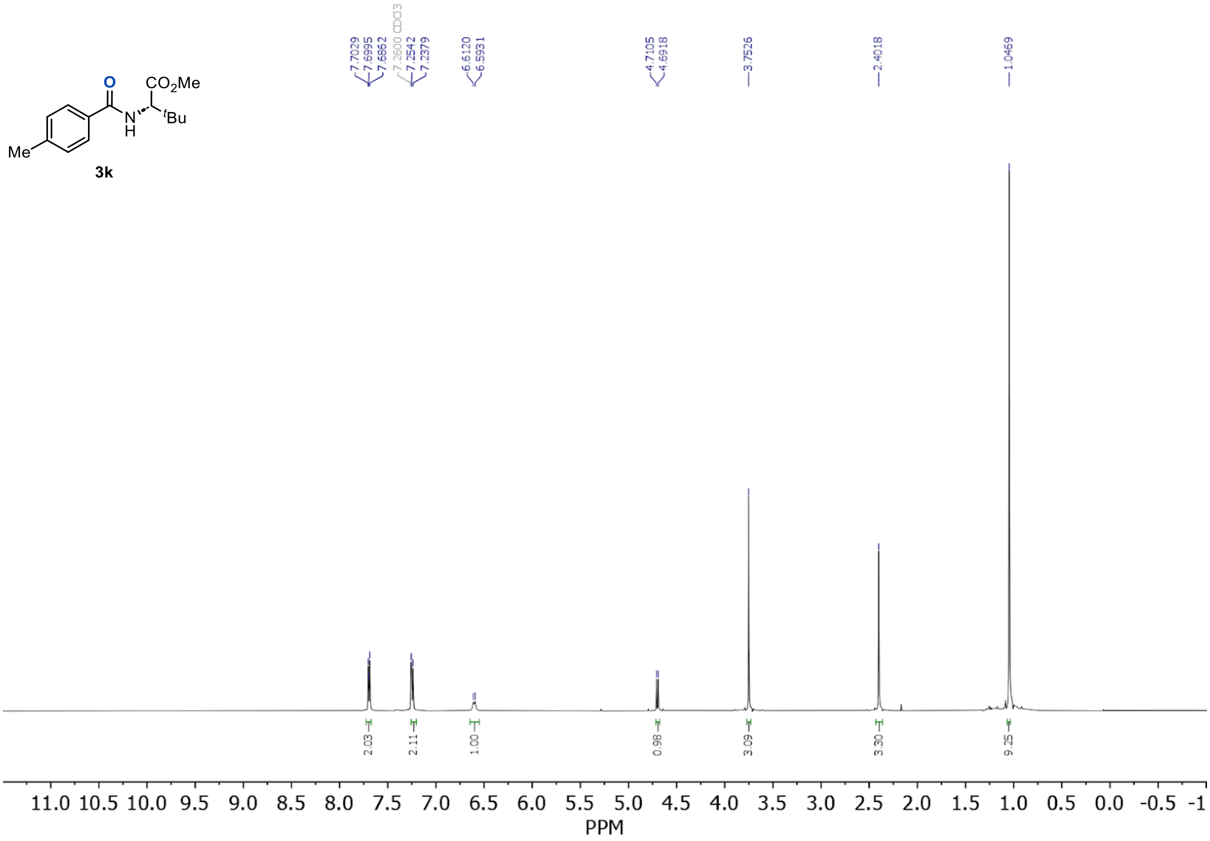

^{13}C{^{1}H} NMR (126 MHz, CDCl$_3$) of methyl (*S*)-3,3-dimethyl-2-(4-methylbenzamido)butanoate (3k)

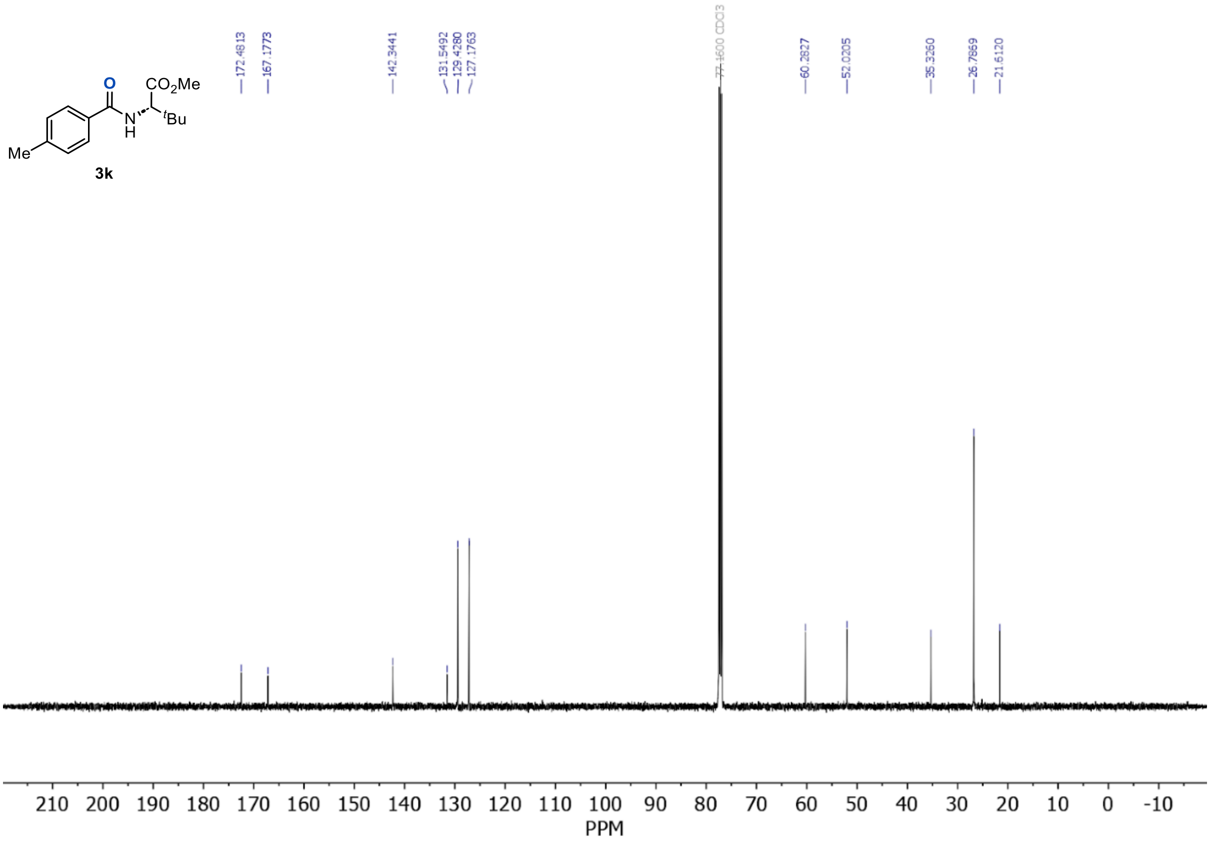

^1H NMR (600 MHz, $CDCl_3$) of (R)-2-((tert-butoxycarbonyl)amino)-3-methoxy-3-oxopropyl 4-methylbenzoate (3l)

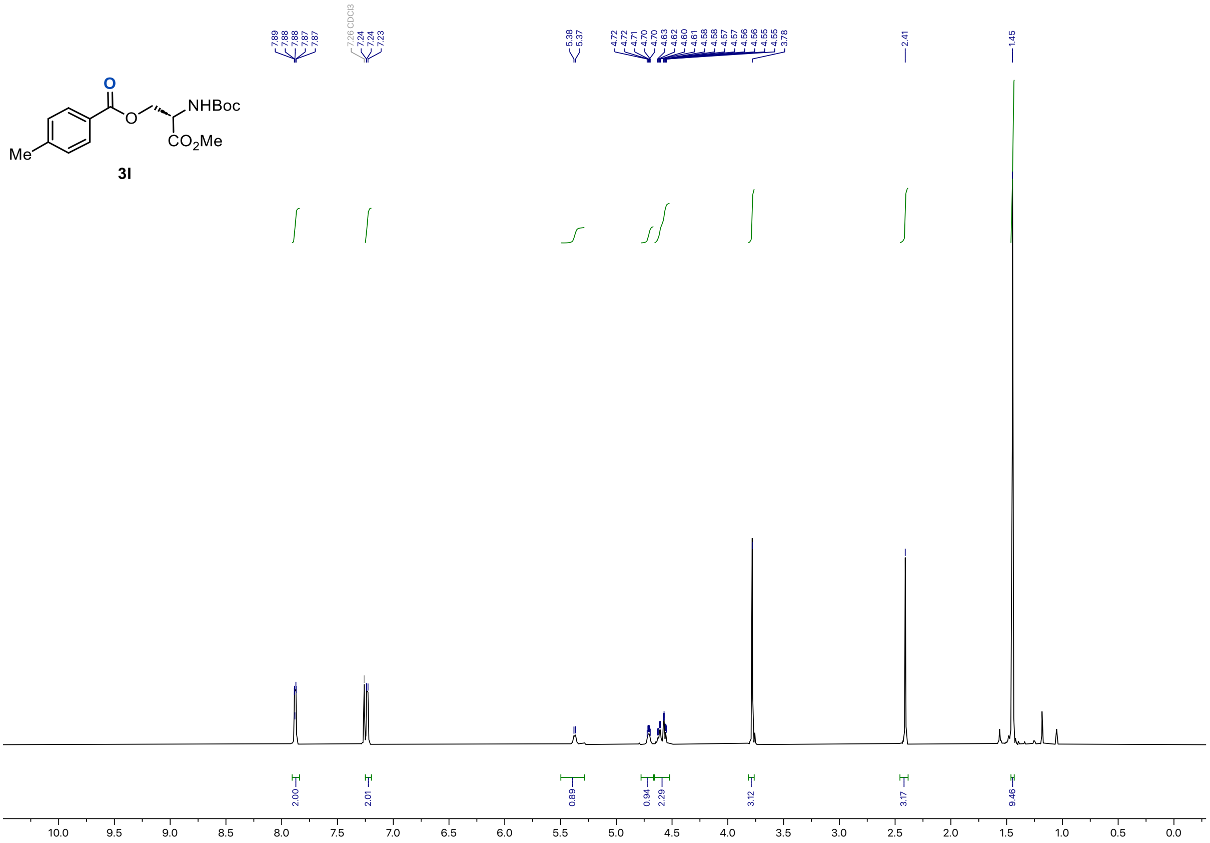

$^{13}C\{^1H\}$ NMR (151 MHz, $CDCl_3$) of (R)-2-((tert-butoxycarbonyl)amino)-3-methoxy-3-oxopropyl 4-methylbenzoate (3l)

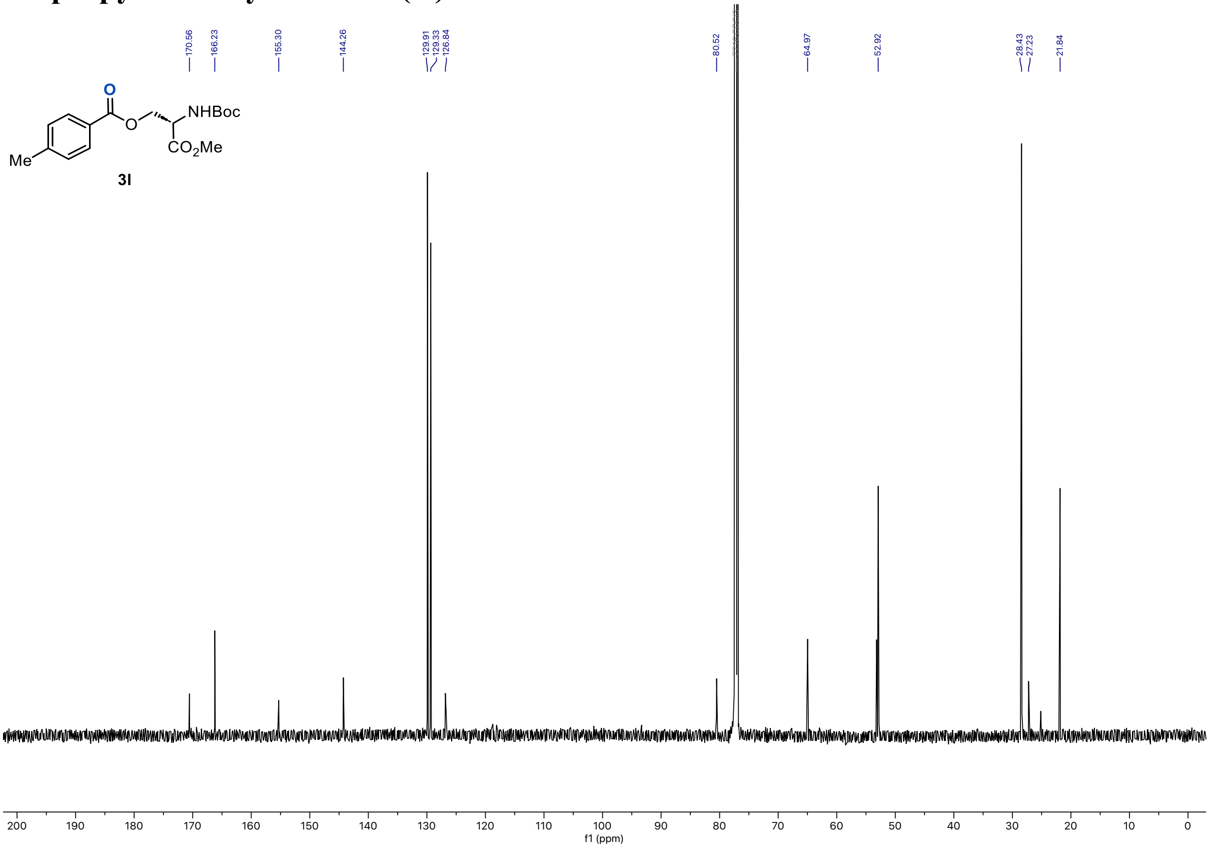

^{1}H NMR (500 MHz, CDCl$_3$) of ethyl N-acetyl-S-(4-methylbenzoyl)-L-cysteinate (3m)

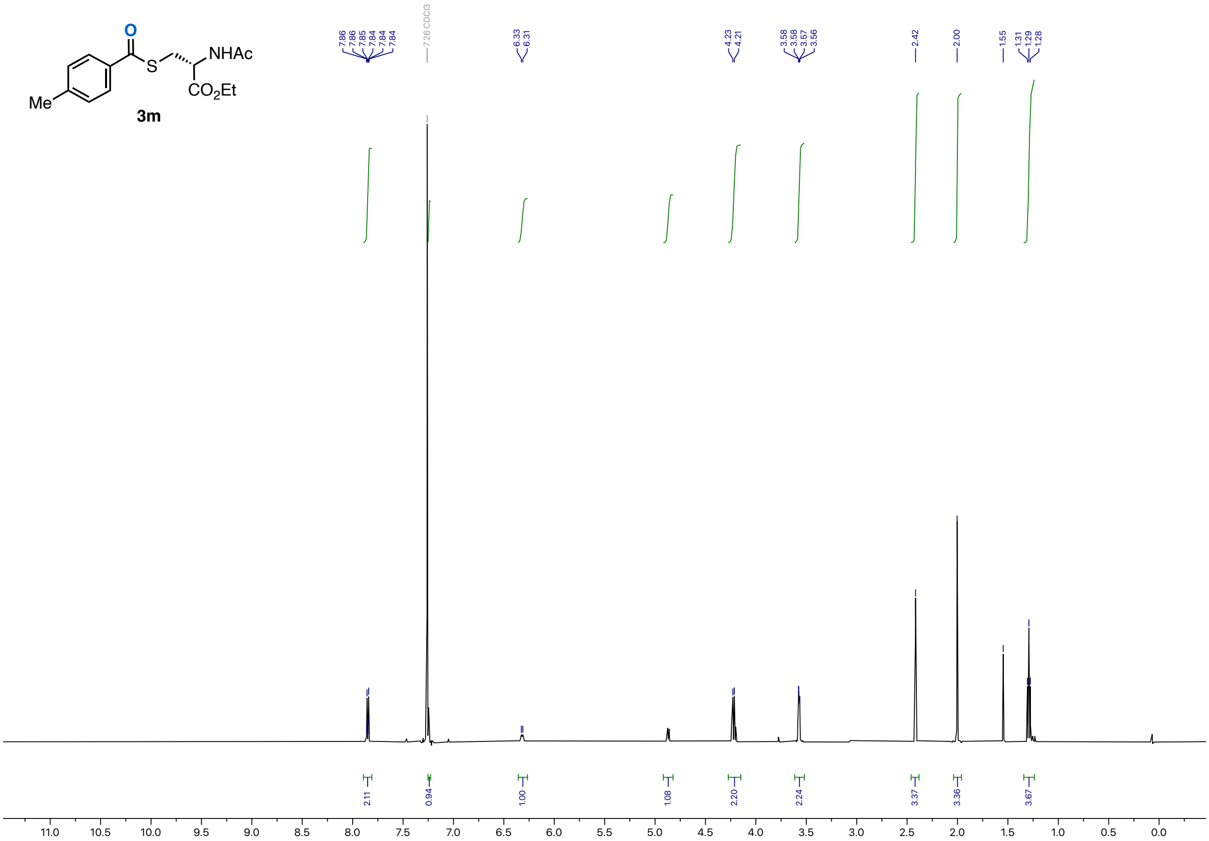

^{13}C{^{1}H} NMR (126 MHz, CDCl$_3$) of ethyl N-acetyl-S-(4-methylbenzoyl)-L-cysteinate (3m)

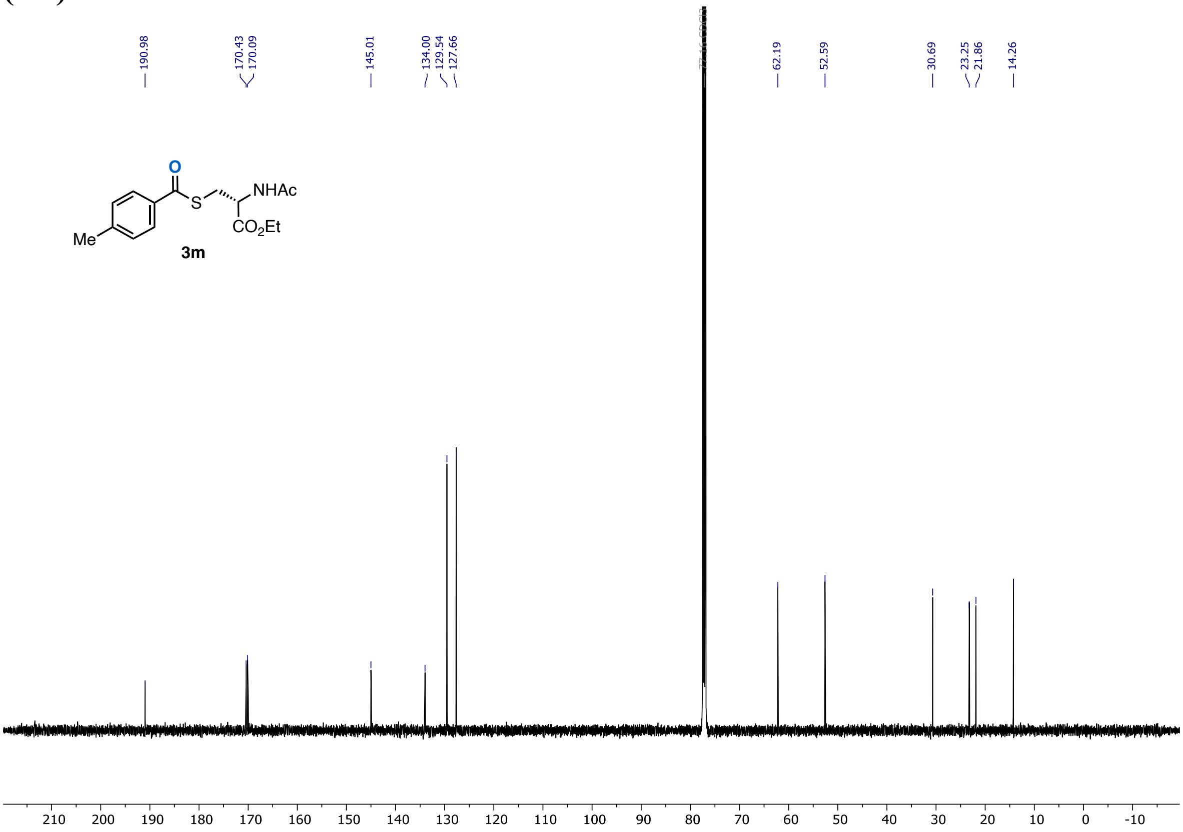

^{1}H NMR (500 MHz, $CDCl_3$) of methyl (S)-3-(4-(tert-butoxy)phenyl)-2-(2-(1,3-dioxoisoindolin-2-yl)acetamido)propanoate (5a)

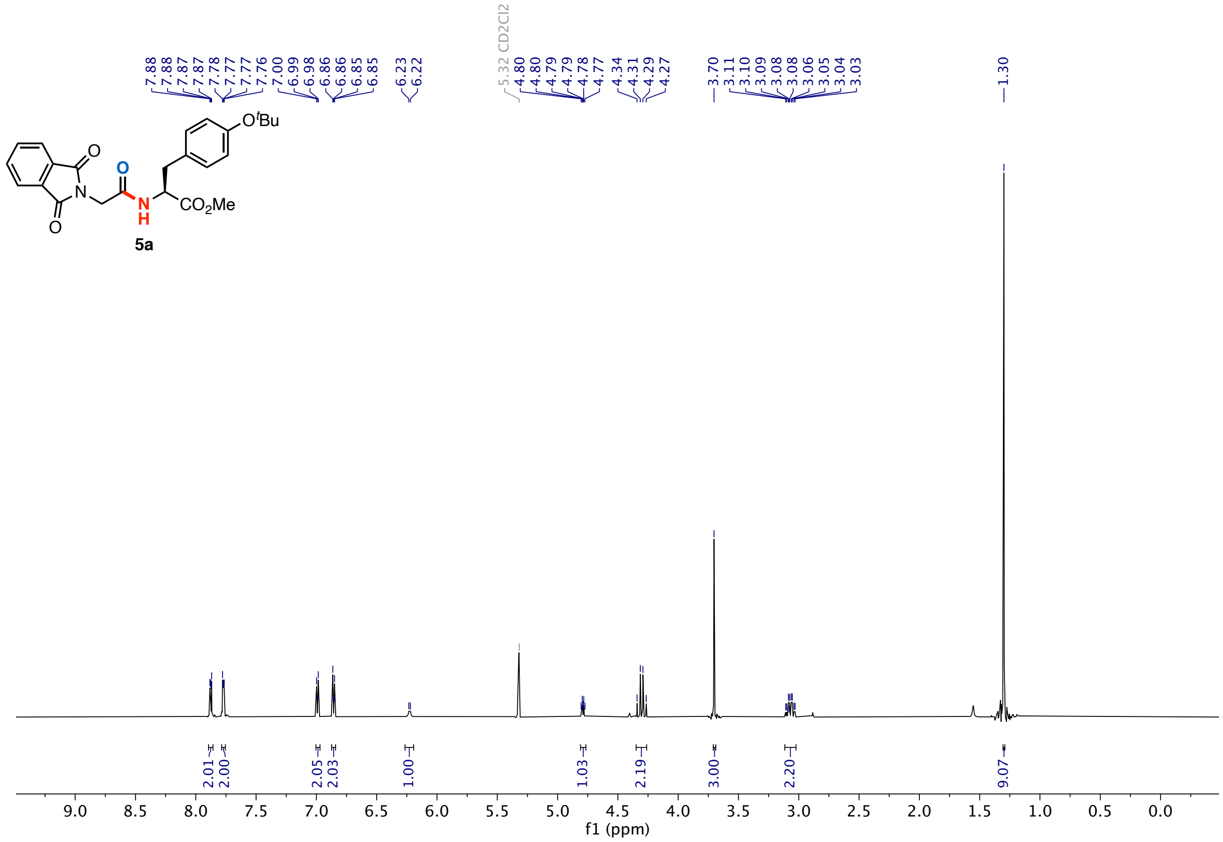

$^{13}C\{^{1}H\}$ NMR (126 MHz, $CDCl_3$) of methyl (S)-3-(4-(tert-butoxy)phenyl)-2-(2-(1,3-dioxoisoindolin-2-yl)acetamido)propanoate (5a)

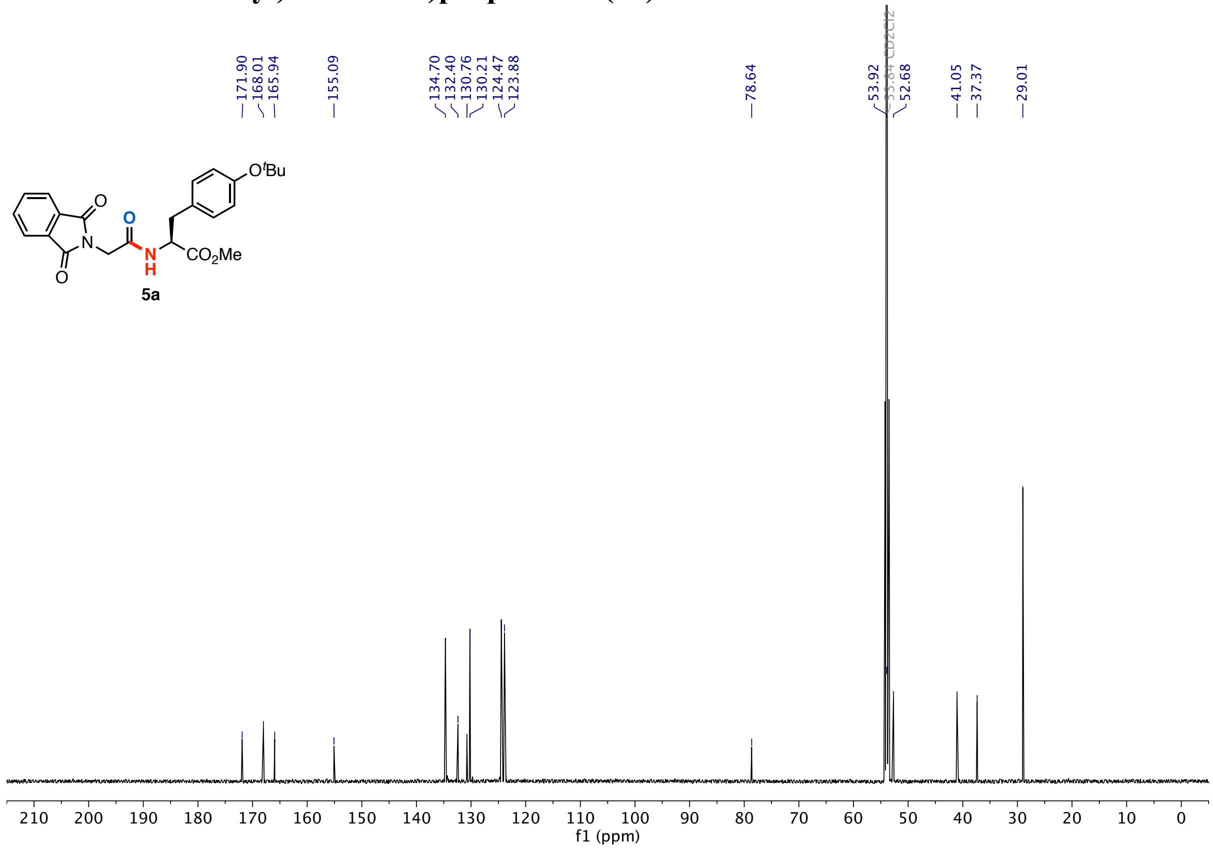

^{1}H NMR (500 MHz, $CDCl_3$) of methyl (*tert*-butoxycarbonyl)-*L*-phenylalanylglycyl-*L*-tryptophanate (5b)

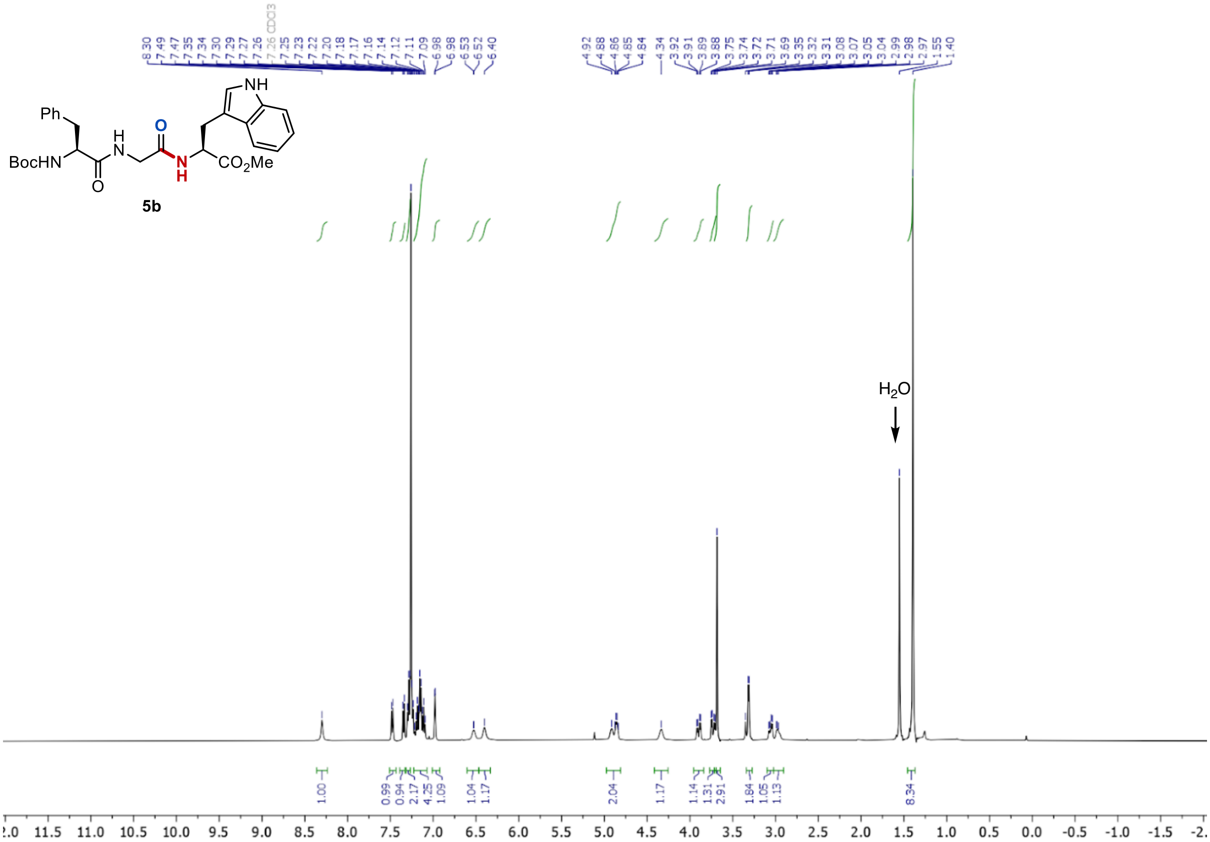

$^{13}C\{^{1}H\}$ NMR (126 MHz, $CDCl_3$) of methyl (*tert*-butoxycarbonyl)-*L*-phenylalanylglycyl-*L*-tryptophanate (5b)

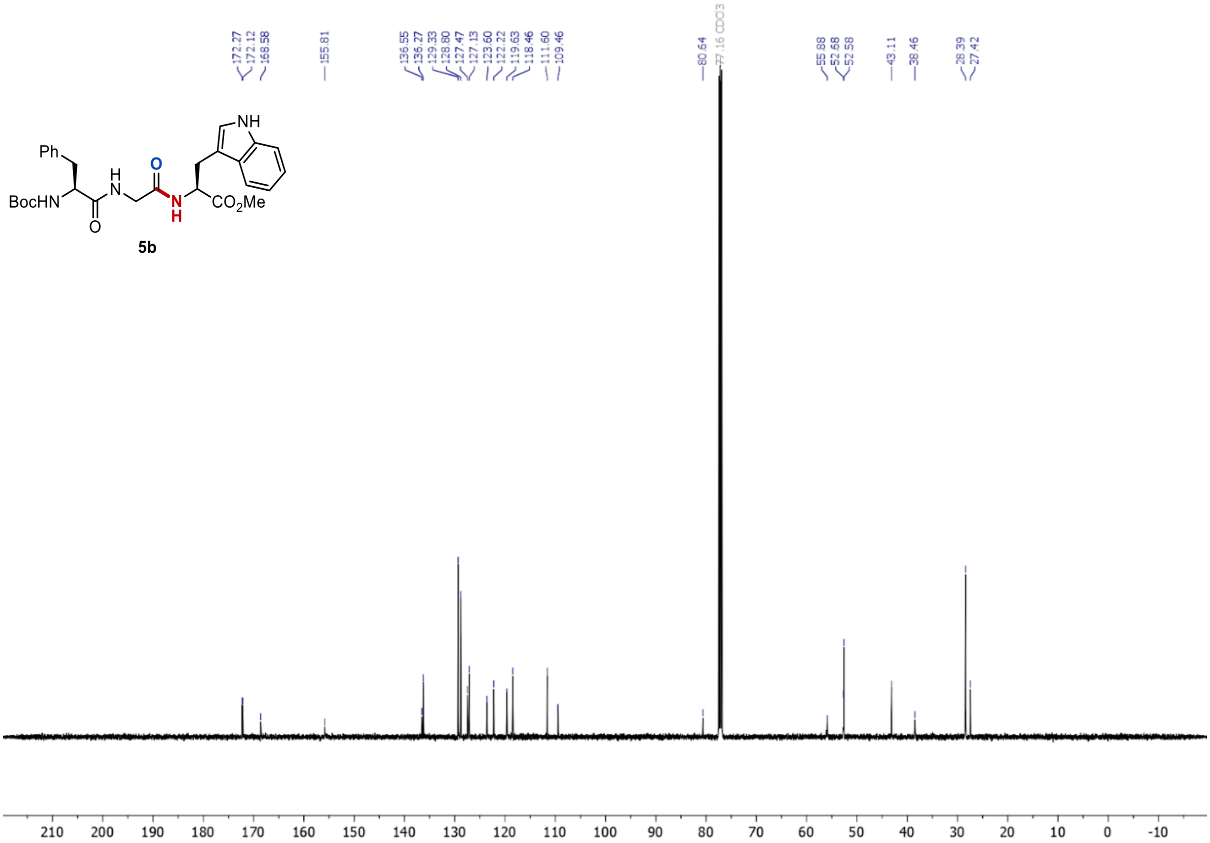

^{1}H NMR (400 MHz, CD_3OD) of BocHN-Gly-Trp(boc)-Pro-*D*-Ala-Thr(*t*Bu)-Gly-Tyr(*t*Bu)-OMe (5c) (pivaldehyde as external standard, 1.14 equiv.)

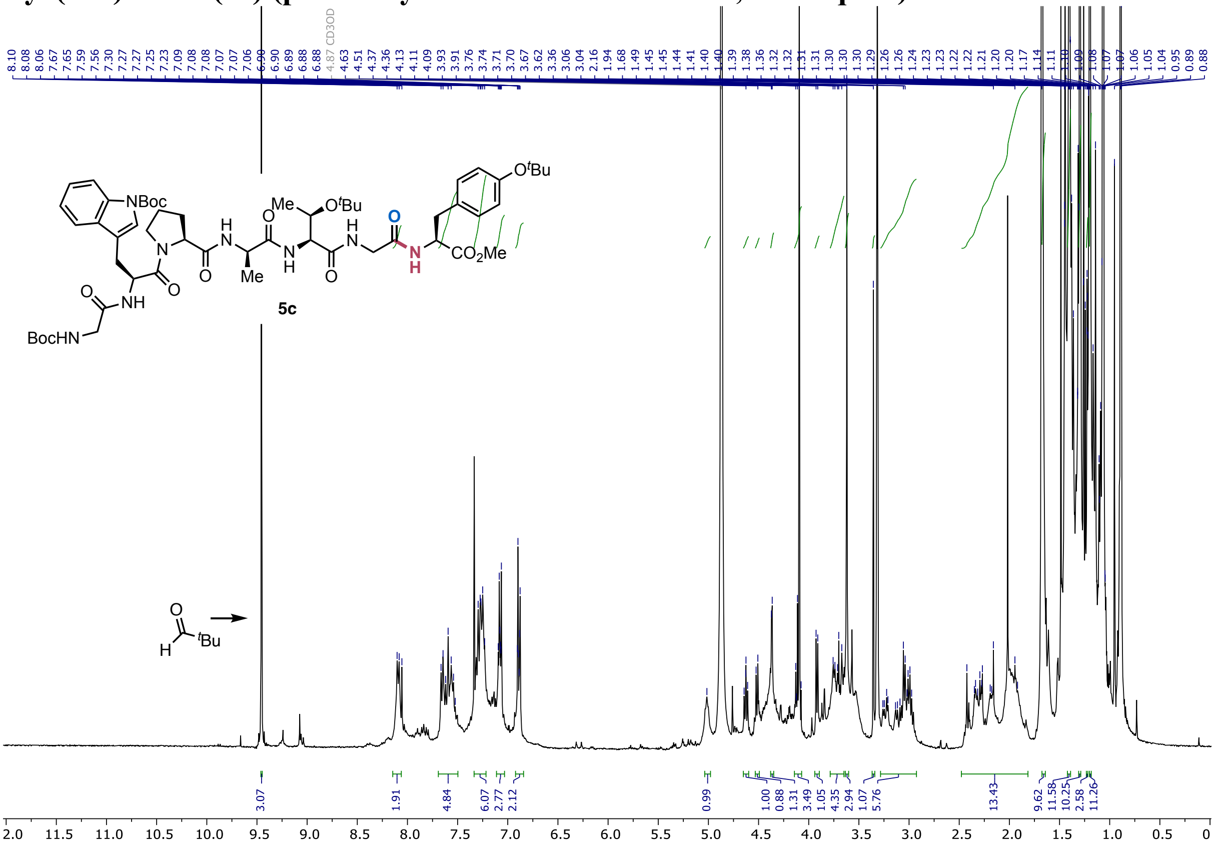

^{1}H NMR (800 MHz, CD_3OD) of BocHN-Gly-Trp(boc)-Pro-*D*-Ala-Thr(*t*Bu)-Gly-Tyr(*t*Bu)-OMe (5c)

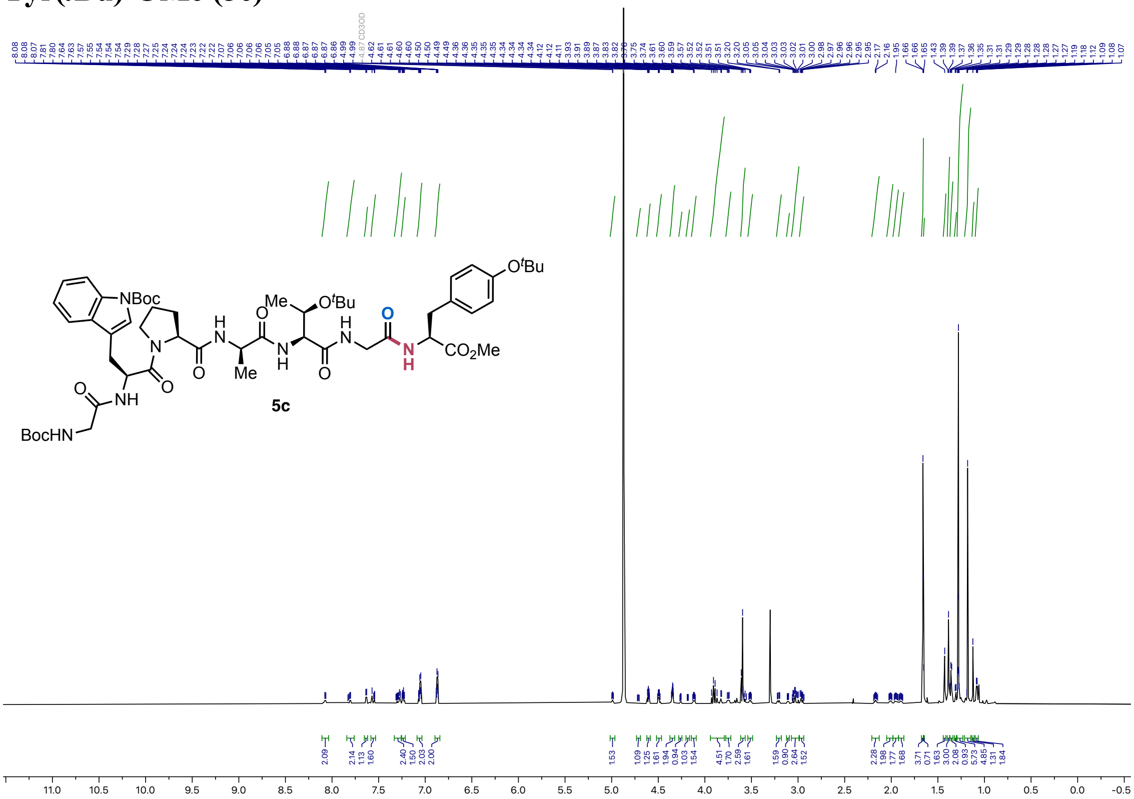

$^{13}C\{^1H\}$ NMR (201 MHz, CD_3OD) of BocHN-Gly-Trp(boc)-Pro-*D*-Ala-Thr(*t*Bu)-Gly-Tyr(*t*Bu)-OMe (5c)

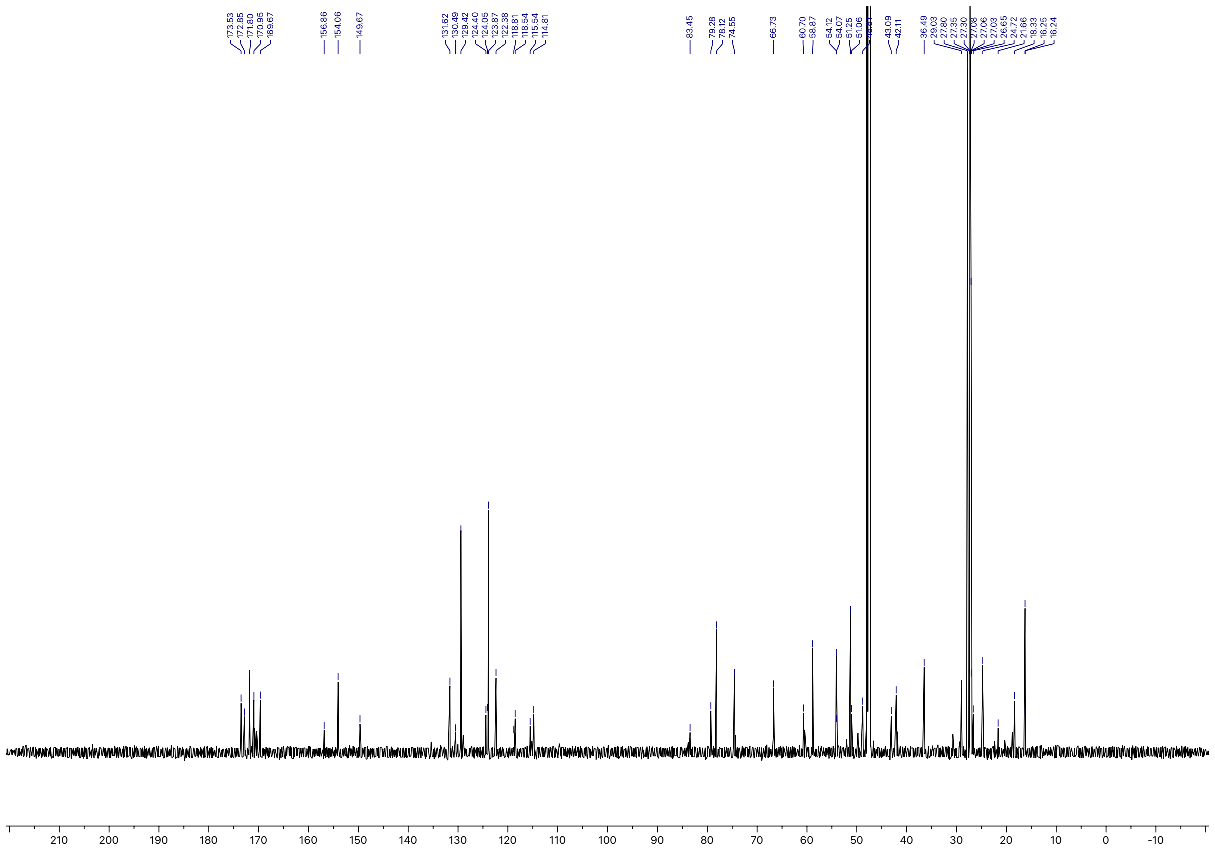

^{1}H NMR Yield (500 MHz, $CDCl_3$) of (2*R*,3*R*,4*S*,5*R*)-2-(acetoxymethyl)-6-(((*tert*-butoxycarbonyl)-*L*-phenylalanylglycyl)oxy)tetrahydro-2*H*-pyran-3,4,5-triyl triacetate (5d) (CH_2Br_2 as external standard, 1.0 equiv)

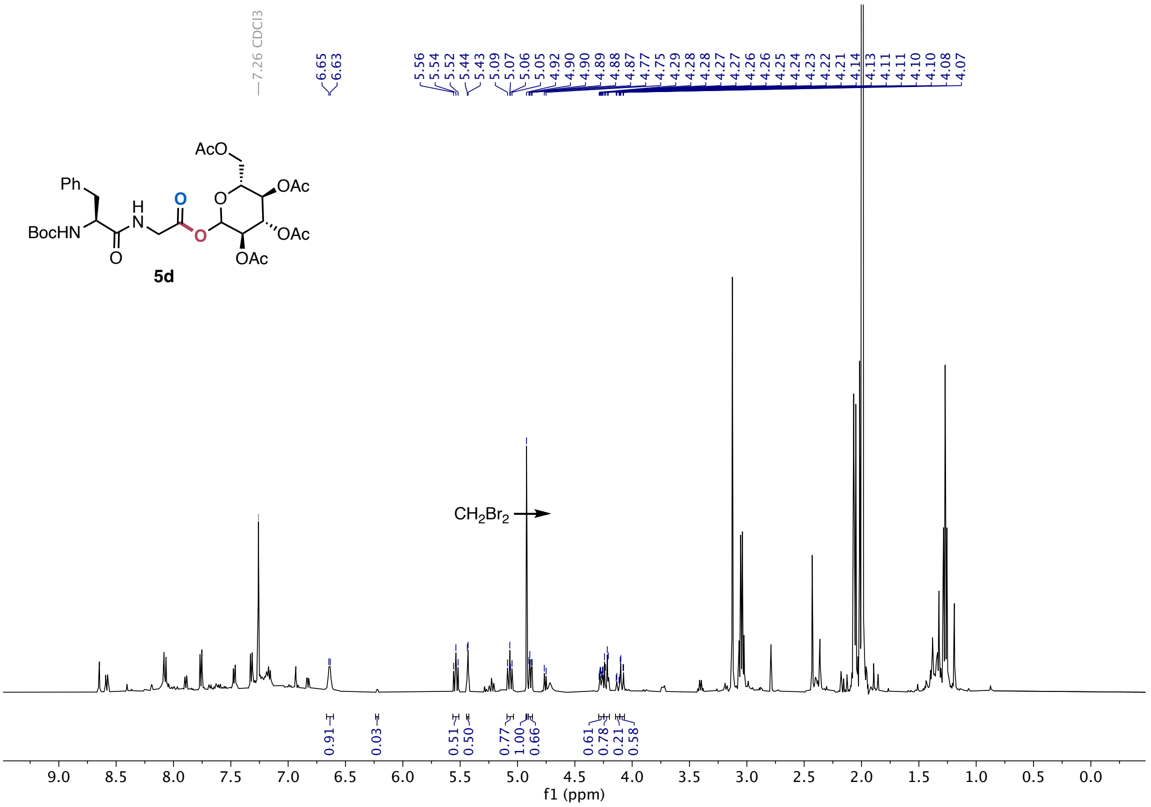

^{1}H NMR (500 MHz, CDCl$_3$) of (2*R*,3*R*,4*S*,5*R*)-2-(acetoxymethyl)-6-(((*tert*-butoxycarbonyl)-*L* phenylalanylglycyl)oxy)tetrahydro-2*H*-pyran-3,4,5-triyl triacetate

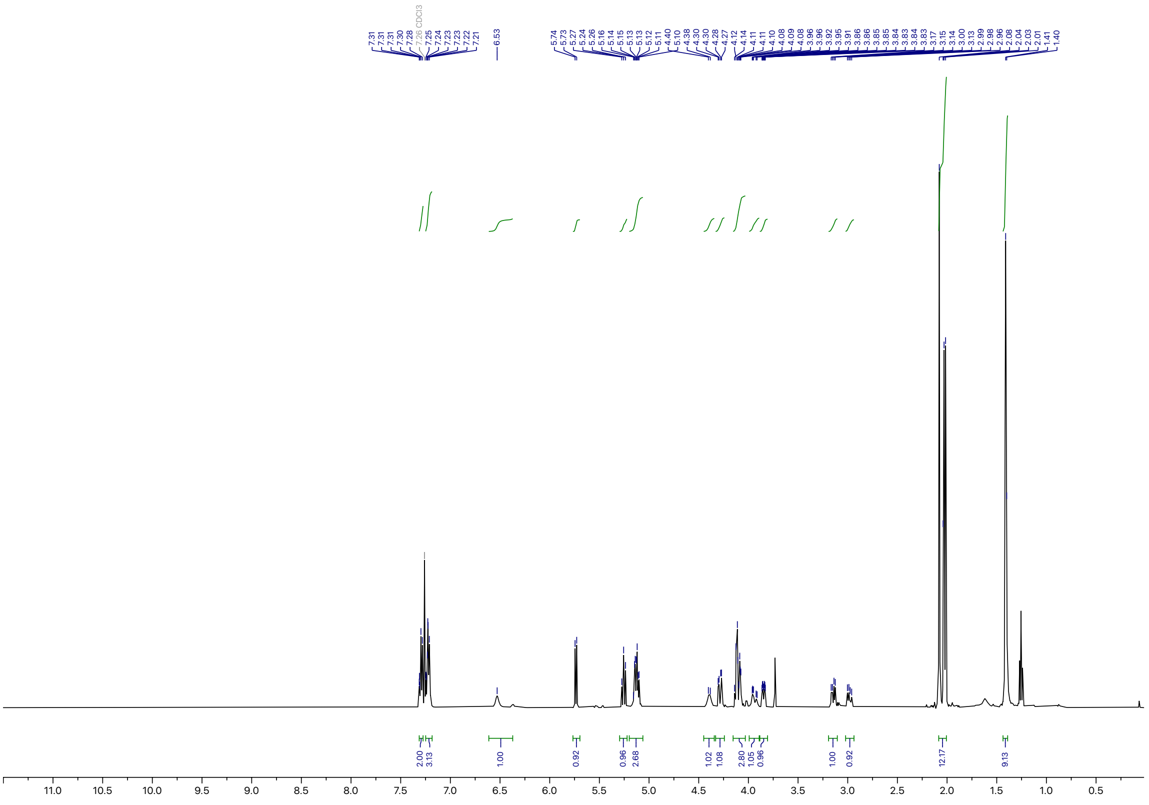

$^{13}C\{^1H\}$ NMR (126 MHz, $CDCl_3$) of (2*R*,3*R*,4*S*,5*R*)-2-(acetoxymethyl)-6-(((*tert*-butoxycarbonyl)-*L* phenylalanylglycyl)oxy)tetrahydro-2*H*-pyran-3,4,5-triyl triacetate

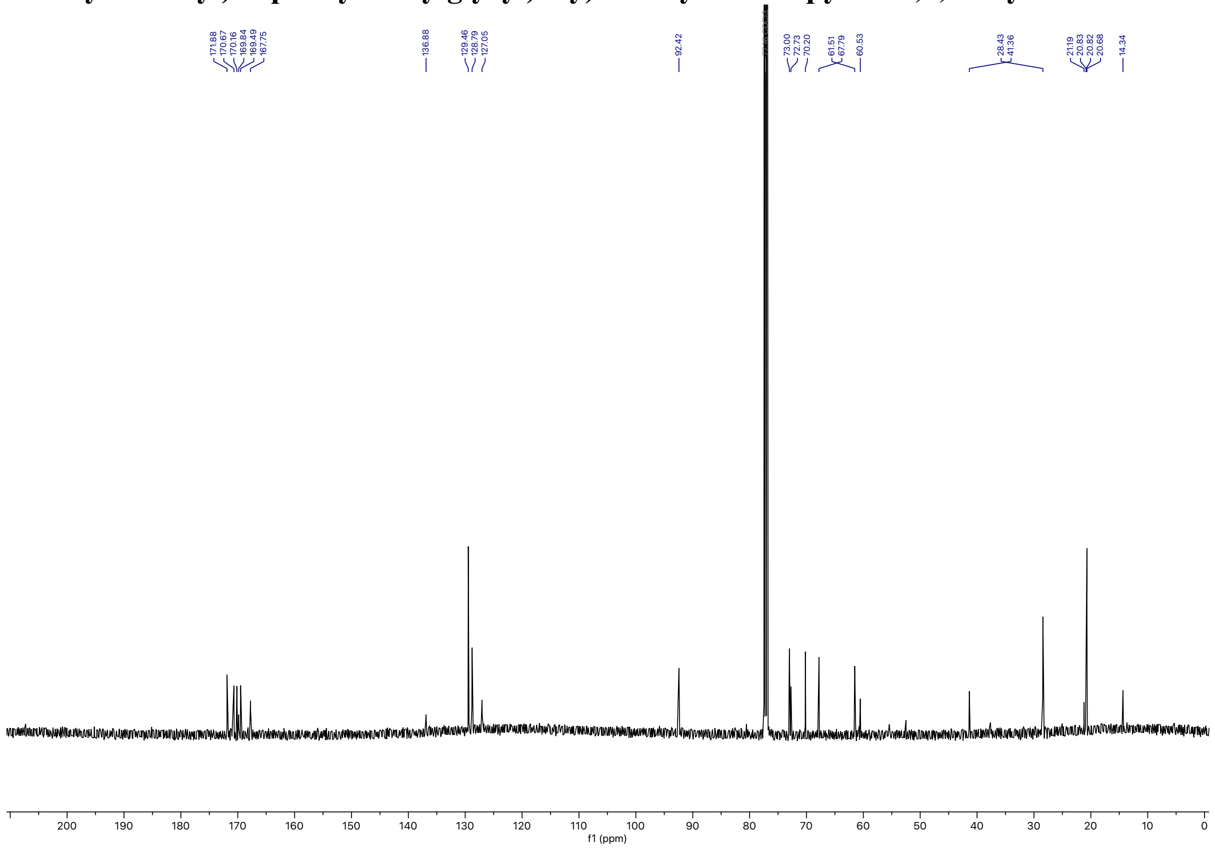

References

1. Sedikides, A. T.; Lennox, A. J. J. Silver-Catalyzed (Z)-β-Fluoro-Vinyl Iodonium Salts from Alkynes: Efficient and Selective Syntheses of Z-Monofluoroalkenes. *J. Am. Chem. Soc.* **2024**, *146*, 15672–15680.
2. Dong, X.; Jiang, W.; Hua, D.; Wang, X.; Xu, L.; Wu, X. Radical-Mediated Vicinal Addition of Alkoxysulfonyl/fluorosulfonyl and Trifluoromethyl Groups to Aryl Alkyl Alkynes. *Chem. Sci.* **2021**, *12*, 11762–11768.
3. Du, D.; Ji, C.; Zheng, S.; Chen, Y.; Cai, H.; Li, Z.; Yan, D.; Teng, H. Synthesis of Difluorocyclopropanes via Visible-Light-Mediated [1+2] Cycloaddition of Diazo Esters with gem-Difluoroalkenes and Evaluation of their Antifungal Activity. *Adv. Synth. Catal.* **2024**, *366*, 1738–1743.
4. Tian, H.; Yang, S.; Wang, X.; Xu, W.; Liu, Y.; Li, Y.; Wang, Q. Dehalogenative Cross-Coupling of gem-Difluoroalkenes with Alkyl Halides via a Silyl Radical–Mediated Process. *J. Org. Chem.* **2021**, *86*, 12772–12782.
5. Wang, W.; Xu, B.; Hammond, G. B. Synthesis of Functionalized α,α-Disubstituted β-Alkynyl Esters from Allenoates through an Alkynylenolate Intermediate. *Org. Lett.* **2008**, *10*, 3713–3716.
6. Garg, A.; Gerwien, N. J.; Fasting, C.; Charlton, A.; Hopkinson, M. N. Formal Insertion of Alkenes into C(sp)−F Bonds Mediated by Fluorine-Hydrogen Bonding. *Angew. Chem. Int. Ed.* **2023**, *62*, e202302860.
7. Yang, Y.; Hammond, G. B.; Umemoto, T. Self-Sustaining Fluorination of Active Methylene Compounds and High-Yielding Fluorination of Highly Basic Aryl and Alkenyl Lithium Species with a Sterically Hindered N-Fluorosulfonamide Reagent. *Angew. Chem. Int. Ed*. **2022**, *61*, e202211688.
8. Yang, R.-Y.; Xu, B. Chemo-, Regio- and Stereoselective Synthesis of Monofluoroalkenes via a Tandem Fluorination-Desulfonation Sequence. *ChemComm* **2021,** *57*, 7802–7805.
9. Zhang, G.; McCorvy, J. D.; Shen, S.; Cheng, J.; Roth, B. L.; Kozikowski, A. P. Design of Fluorinated Cyclopropane Derivatives of 2-Phenylcyclopropylmethylamine Leading to Identification of a Selective Serotonin 2C (5-HT_{2C}) Receptor Agonist Without 5-HT_{2B} Agonism. *Eur. J. Med. Chem.* **2019,** *182*, 111626.
10. Zeng, Y.; Jiang, Z.-T.; Zhu, Y.; Chen, J.; Zhang, H.; Xia, Y. Carbofluorination of Alkenes with *gem*-Difluorinated Cyclopropanes as Bifunctional Reagents Enabled by Well-Define Rhodium Catalysts. *ChemRxiv* **2023**, DOI: 10.26434/chemrxiv-2023-8mj8n.
11. Okoromoba, O. E.; Han, J.; Hammond, G. B.; Xu, B. Designer HF-Based Fluorination Reagent: Highly Regioselective Synthesis of Fluoroalkenes and *gem*-Difluoromethylene Compounds from Alkynes. *J. Am. Chem. Soc.* **2014,** *136*, 14381–14384.
12. Seath, C. P.; Vogt, D. B.; Xu, Z.; Boyington, A. J.; Jui, N. T. Radical Hydroarylation of Functionalized Olefins and Mechanistic Investigation of Photocatalytic Pyridyl Radical Reactions. *J. Am. Chem. Soc*. **2018**, *140*, 15525–15534.
13. Yang, M.-H.; Matikonda, S. S.; Altman, R. A. Preparation of Fluoroalkenes via the Shapiro Reaction: Direct Access to Fluorinated Peptidomimetics. *Org. Lett.* **2013,** *15*, 3894–3897.
14. Zhang, Q.-Q.; Chen, S.-Y.; Lin, E.; Wang, H.; Li, Q. Regio- and Stereoselective Alkenylation of Allenoates with *gem*-Difluoroalkenes: Facile Access to Fluorinated 1,4-Enynes Bearing an All-Carbon Quaternary Center. *Org. Lett.* **2019,** *21*, 3123–3126.
15. Pfeifer, L.; Gouverneur, V. Controlled Single and Double Iodofluorination of Alkynes with DIH- and HF-Based Reagents. *Org. Lett*. **2018**, *20*, 1576–1579.

16. Wang, Y. M.; Bruno, N. C.; Placeres, Á. L.; Zhu, S.; Buchwald, S. L. Enantioselective Synthesis of Carbo- and Heterocycles through a CuH-Catalyzed Hydroalkylation Approach. *J. Am.Chem. Soc*. **2015**, *137*, 10524–10527.
17. Merchant, R. R.; Edwards, J. T.; Qin, T.; Kruszyk, M. M.; Bi, C.; Che, G.; Bao, D.-H.; Qiao, W.; Sun, L.; Collins, M. R.; Fadeyi, O. O.; Gallego, G. M.; Mousseau, J. J.; Nuhant, P.; Baran, P. S. Modular Radical Cross-Coupling with Sulfones Enables Access to sp^3-rich (Fluoro)alkylated Scaffolds. *Science* **2018**, *360*, 75–80.
18. Nambo, M.; Ghosh, K.; Yim, J. C. H.; Tahara, Y.; Inai, N.; Yanai, T.; Crudden, C. M. Desulfonylative Coupling of Alkylsulfones with *gem*-Difluoroalkenes by Visible-Light Photoredox Catalysis. *ACS Catal.* **2022**, *12*, 9526–9532.
19. Ohshima, T.; Hayashi, Y.; Agura, K.; Fujii, Y.; Yoshiyama, A.; Mashima, K. Sodium Methoxide: a Simple but Highly Efficient Catalyst for the Direct Amidation of Esters. *Chem.Commun.* **2012**, *48*, 5434–5436.
20. Wu, W.; Yi, J.; Xu, H.; Li, S.; Yuan, R. An Efficient, One-Pot Transamidation of 8-Aminoquinoline Amides Activated by Tertiary-Butyloxycarbonyl. *Molecules* **2019**, *24*, 1234.
21. Wang, C.; Tan, C.; Zhu, Y.; Liu, G.; Huang, Y.; Liu, R. A Site-Selective $C(sp^3)$-H Chlorination Boosted by an Imidazolium-Functionalized Cage in Water. *ACS Catal.* **2023**, *13*, 13896–13901.
22. Karimi, F.; Kihlberg, T.; Långström, B. $[^{11}C]/(^{13}C)$Carbon Monoxide in Palladium-Mediated Synthesis of Imides. *J. Chem. Soc., Perkin Trans. 1.* **2001**, 1528–1531.
23. Langton, M. J.; Duckworth, L. C.; Beer, P. D. Nitrate Anion Templated Assembly of a [2]Rotaxane for Selective Nitrate Recognition in Aqueous Solvent Mixtures. *Chem. Commun.* **2013**, *49*, 8608–8610.
24. Xu, D.; Wang, L.; Yao, F.; Shi, M.; Xie, M.; Li, T.; Nie, R.; Gou, H.; Zhao, G.; Sun, W. Direct Use of Hydroxyl Ions as an Oxygen Source for Oxidation of Isoquinolinium Salts to Isoquinolinones in Water Solution. *New J. Chem*. **2023**, *47*, 3921–3927.
25. Ishitani, H.; Takeno, K.; Sasaya, M.; Kobayashi, S. Continuous-Flow Dehydrative Amidation Between Carboxylic Acids and Amines Using Modified Mixed Metal Oxides as Solid Acid Catalysts. *Catal. Sci. Technol.* **2023**, *13*, 5536–5542.
26. Pan, B.; Huang, D. M.; Sun, H. T.; Song, S. N.; Su, X. B. Heterocyclic Boron Acid Catalyzed Dehydrative Amidation of Aliphatic/Aromatic Carboxylic Acids with Amines. *J. Org. Chem.* **2023**, *88*, 2832–2840.
27. Petchey, T. H. M.; Comerford, J. W.; Farmer, T. J.; Macquarrie, D. J.; Sherwood, J.; Clark, J. H. Optimization of Amidation Reactions Using Predictive Tools for the Replacement of Regulated Solvents with Safer Biobased Alternatives. *ACS Sustainable Chem. Eng.* **2018**, *6*, 1550–1554.
28. Thanh Dang, T.; Chen, A.; Majeed Seayad, A. An Efficient Synthesis of Weinreb Amides and Ketones via Palladium Nanoparticles on ZIF-8 Catalysed Carbonylative Coupling. *RSC Adv*. **2014**, *4*, 30019–30027.
29. Che, Z.; Yang, J.; Sun, D.; Tian, Y.; Shengming, L.; Lin, X.; Jiang, J.; Chen, G. Synthesis of Novel (9*S*)-Acyloxy Derivatives of Quinidine and Dihydroquinidine as Insecticidal Agents. *C&B* **2020**, *17*, e1900696.
30. Yi, C. L.; Huang, Y. T.; Lee, C. F. Synthesis of Thioesters Through Copper-Catalyzed Coupling of Aldehydes with Thiols in Water. *Green Chem*. **2013**, *15*, 2476–2484.
31. Wise, D. E.; Gogarnoiu, E. S.; Duke, A. D.; Paolillo, J. M.; Vacala, T. L.; Hussain, W. A.; Parasram, M. Photoinduced Oxygen Transfer Using Nitroarenes for the Anaerobic Cleavage of Alkenes. *J. Am. Chem. Soc.* **2022**, *144*, 15437–15442.
32. Boreux, A.; Indukuri, K.; Gagosz, F.; Riant, O. Acyl Fluorides as Efficient Electrophiles for the Copper-Catalyzed Boroacylation of Allenes. *ACS Catal.* **2017**, *7*, 8200–8204.

33. Cohen, O.; Sasson, R.; Rozen, S. A New Method for Making Acyl Fluorides Using BrF_3. *J. Fluor. Chem* **2006**, *127*, 433 – 436
34. S. J. McCarver, J. X. Qiao, J. Carpenter, R. M. Borzilleri, M. A. Poss, M. D. Eastgate, M. M. Miller, D. W. C. MacMillan. Decarboxylative Peptide Macrocyclization through Photoredox Catalysis. *Angew. Chem. Int. Ed.* **2017**, *56*, 728.
35. Gerhardt, W. W.; Weck, M. Investigations of Metal-Coordinated Peptides as Supramolecular Synthons. *J.Org. Chem.* **2006**, *71*, 6333–6341.
36. Das, M.; Senapati, K.; Panda, S. S.; Bhattacharya, P.; Jana, S.; Mandal, S. M.; Basak, A. Pi-Stacking Assisted Redox Active Peptide-Gallol Conjugate: Synthesis of a New Generation of Low-Toxicity Antimicrobial Silver Nanoparticles. *RSC Adv.* **2016**, *6*, 85254–85260.
